# Tenascin‐C immobilizes infiltrating T lymphocytes through CXCL12 promoting breast cancer progression

**DOI:** 10.15252/emmm.202013270

**Published:** 2021-05-14

**Authors:** Devadarssen Murdamoothoo, Zhen Sun, Alev Yilmaz, Gilles Riegel, Chérine Abou‐Faycal, Claire Deligne, Ines Velazquez‐Quesada, William Erne, Marine Nascimento, Matthias Mörgelin, Gérard Cremel, Nicodème Paul, Raphael Carapito, Romain Veber, Hélène Dumortier, Jingping Yuan, Kim S Midwood, Thomas Loustau, Gertraud Orend

**Affiliations:** ^1^ The Tumor Microenvironment Laboratory INSERM UMR_S 1109 Faculté de Médecine Hopital Civil Institut d'Hématologie et d'Immunologie Strasbourg France; ^2^ The Microenvironmental Niche in Tumorigenesis and Targeted Therapy (MN3T) INSERM UMR_S 1109 Faculté de Médecine Hautepierre France; ^3^ Université Strasbourg Strasbourg France; ^4^ Fédération de Médecine Translationnelle de Strasbourg (FMTS) Strasbourg France; ^5^ Department of Gastrointestinal Surgery Tongji Hospital Tongji Medical College in Huazhong University of Science and Technology Wuhan China; ^6^ Tongji Cancer Research Institute Tongji Hospital Tongji Medical College in Huazhong University of Science and Technology Wuhan China; ^7^ Kennedy Institute of Rheumatology University of Oxford Oxford UK; ^8^ Colzyx AB Lund Sweden; ^9^ GENOMAX platform INSERM UMR_S 1109 Institut thématique interdisciplinaire (ITI) de Médecine de Précision de Strasbourg Transplantex NG Faculté de Médecine Fédération Hospitalo‐Universitaire OMICARE, LabEx TRANSPLANTEX Strasbourg France; ^10^ Institut de Biologie Moléculaire et Cellulaire CNRS, UPR3572 Immunologie Immunopathologie et Chimie Thérapeutique Institut de Biologie Moléculaire et Cellulaire Strasbourg France; ^11^ Department of Pathology Renmin Hospital of Wuhan University Wuhan China

**Keywords:** CD8 tumor infiltrating lymphocytes, CXCL12, extracellular matrix, tenascin‐C, Tumor immune microenvironment, Cancer, Immunology

## Abstract

Immune checkpoint therapy, where CD8 tumor infiltrating T lymphocytes (TIL) are reactivated, is a promising anti‐cancer treatment approach, yet with low response rates. The extracellular matrix, in particular tenascin‐C, may generate barriers for TIL. To investigate this possibility, we used a MMTV‐NeuNT and syngeneic mammary gland grafting model derived thereof with engineered tenascin‐C levels and observed accumulation of CD8 TIL in tenascin‐C‐rich stroma. Inhibition studies revealed that tenascin‐C induced CXCL12 through TLR4. By binding CXCL12, tenascin‐C retained CD8 TIL in the stroma. Blockade of CXCR4, the receptor of CXCL12, enhanced macrophage and CD8 TIL infiltration and reduced tumor growth and subsequent metastasis. Retention of CD8 TIL by tenascin‐C/CXCL12 was also observed in human breast cancer by tissue staining. Moreover, whereas high CD8 TIL numbers correlated with longer metastasis‐free survival, this was not the case when also tenascin‐C and CXCL12 levels were high. Altogether, these results may be useful for improving tumor immunity as diagnostic tool and to formulate a future “TIL‐matrix‐release‐and‐reactivate” strategy.

## Graphical abstract

Matrix molecule Tenascin‐C influences immune exclusion by retaining CD8 TILs in the stroma. The release of CD8 TILs from the matrix restores immune defense, resulting in reduced tumor growth and subsequent metastasis of human breast cancer.

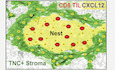

**The paper explained**

**Problem**

Immune checkpoint therapy (ICT), where CD8 tumor infiltrating T lymphocytes (TIL) get reactivated, is a promising anti‐cancer treatment approach, yet with low response rates in breast cancer. Immune exclusion by the matrix, in particular by the highly abundant matrix molecule tenascin‐C, forming spatial niches separating tumor cell nests, may generate barriers for TIL.

**Results**

We combine evidence from clinical material and experimental models to reveal that through induction (involving TLR4) and binding of CXCL12, tenascin‐C impairs macrophages and CD8 TIL. We report that a tenascin‐C/CXCL12 substratum retains CD8 TIL in the matrix impairing tumor cell killing. This molecular axis is important as inhibition of CXCR4 (CXCL12 receptor) restores anti‐tumor immunity by activating and releasing CD8 TIL from the matrix, promoting CD8 TIL tumor infiltration and tumor cell apoptosis, and causing reduced tumor growth and subsequent lung metastasis. Notably, in human breast cancer patients, we document an immune exclusion phenotype correlating with shorter survival characterized by CD8 TIL retention in tenascin‐C‐rich stroma, together with high CXCL12.

**Impact**

This study provides novel insight into how matrix retains CD8 TIL contributing to the immune exclusion phenotype, information that could be exploited to formulate a novel “TIL‐matrix‐release‐and‐reactivate” strategy. Moreover, our results provide rationale for targeting matrix plus CXCR4 for improving ICT in particular in tumors with high tenascin‐C, high CXCL12, and high CD8 TIL as this combination correlates with short breast cancer patient survival.

## Introduction

Immune checkpoint therapy (ICT) is a promising approach to activate the body’s immune system to fight cancer. Yet, in most breast cancer patients, ICT is only efficient in up to 20% of patients (Duraiswamy *et al,* 2013). Inactive CD8 TIL can get reactivated with antibodies targeting PD1, PD‐L1, or CTLA (Duraiswamy *et al,* 2013; Huang *et al,* 2017); however, CD8 TIL are often either scarce or located inside the tumor stroma not reaching the tumor cells (Joyce & Fearon, 2015). The “immune contexture” and tumor microenvironment (TME) have been identified as potential obstacle in ICT (Fridman *et al,* 2012; Zemek *et al,* 2020). In particular, the so‐called “immunoscore” takes into account the exclusion of CD8 TIL from tumor cell clusters (Galon & Bruni, 2019) which are often organized as nests surrounded by stroma, that is rich in extracellular matrix (ECM) (Pickup *et al,* 2014; Spenlé *et al,* 2015). Yet, the roles of the ECM in controlling CD8 TIL infiltration are not well understood (Erdag *et al,* 2012; Galon *et al,* 2012; Joyce & Fearon, 2015).

The ECM molecule tenascin‐C (TNC) is highly expressed in the tumor stroma in regions called tumor matrix tracks (TMT), that are composed of several matrix molecules and are enriched with fibroblasts and leukocytes (Spenlé *et al,* 2015; Spenlé *et al,* 2020). TNC is also expressed in regions with high collagen‐rich tumor‐associated collagen signatures (TACS) that correlate with poor breast cancer patient survival (Tomko *et al,* 2018). TNC has many context‐dependent functions (Midwood *et al,* 2016). TNC can impair adhesion, proliferation, and function of cytotoxic T lymphocytes (CTL) and can be degraded by autophagy that is defective in triple‐negative breast cancer causing CTL impairment (Rüegg *et al,* 1989; Hemesath *et al,* 1994; Hauzenberger *et al,* 1999; Parekh *et al,* 2005; Huang *et al,* 2010; Li *et al,* 2020). TNC skewed macrophages toward a M2 phenotype through TLR4 promoting lung metastasis in the syngeneic NT193 breast cancer model, that has been used in the current study (Deligne *et al,* 2020). Moreover, in an oral squamous cell carcinoma model, TNC promoted an immune suppressive TME through CCR7/CCL21 (Spenlé *et al,* 2020). Altogether, TNC may contribute to evasion from immune surveillance in breast cancer by regulating CTL function but how is largely unknown.

Here, by investigating two breast cancer progression models with engineered high and low TNC levels, we demonstrated that TNC contributes to the escape from anti‐tumor immunity by regulating CD8 TIL. TNC impaired CD8 TIL function through induction of CXCL12. TNC bound CXCL12, thus immobilizing CD8 TIL in the matrix. Subsequently, these lymphocytes were impaired in reaching and killing the tumor cells. Upon inhibition of CXCR4, CD8 TIL were released from the TNC‐rich stroma, now accessing and killing the tumor cells causing reduced tumor growth and subsequent metastasis. This mechanism may be relevant in human breast cancer where CD8 TIL accumulation in the TNC‐rich stroma in context of high CXCL12 correlated with shortest survival of breast cancer patients.

## Results

### TNC regulates expression of immunity‐associated genes in the MMTV‐NeuNT model

**Figure 1:**
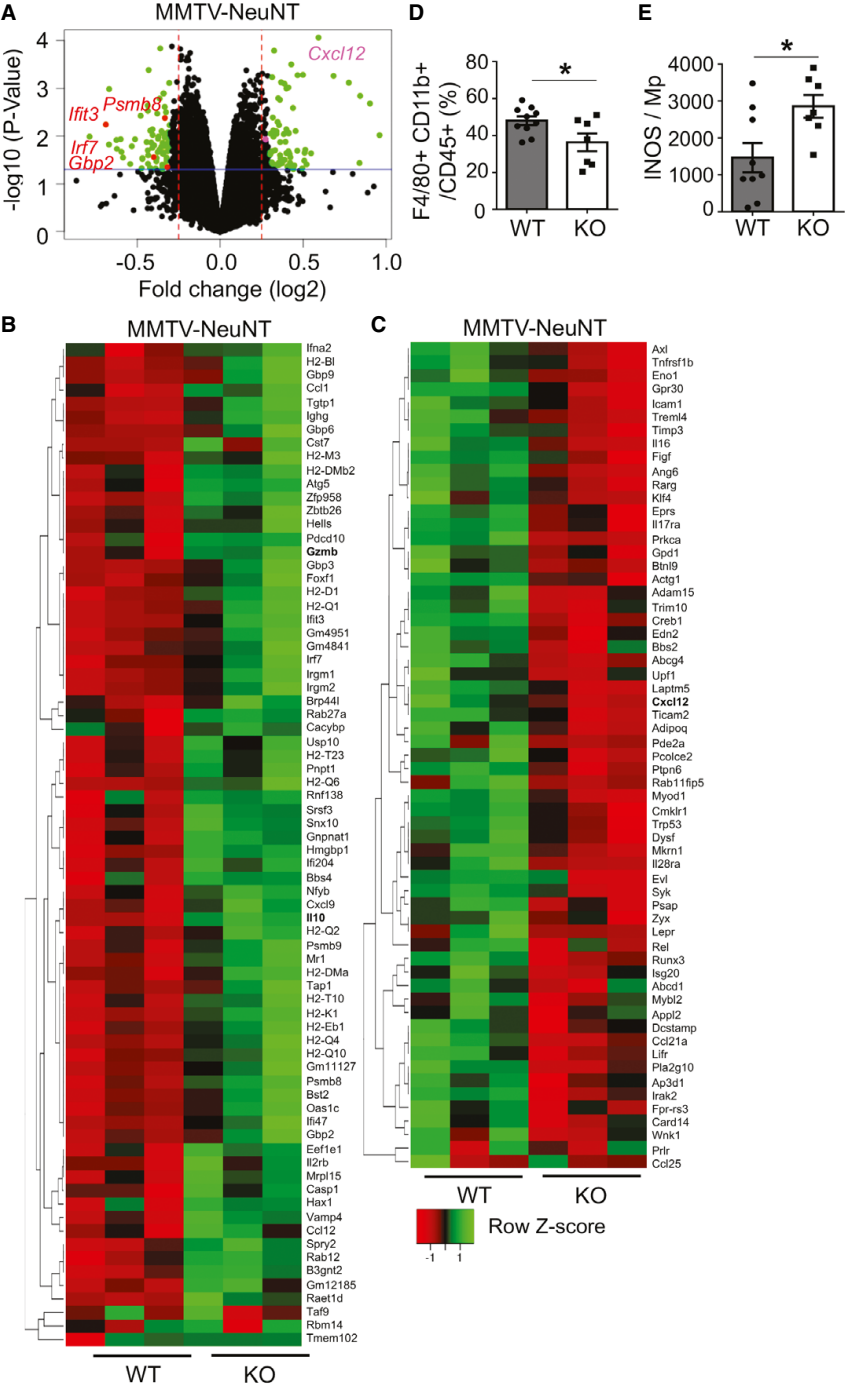
Loss of the TNC protein in NeuNT tumors impacts gene expression and the immune cell infiltrate A–CDisplay of differentially expressed genes in NeuNT tumors (WT, TNCKO) as volcano plot (A) and heatmaps (B, C) representing all (A) and immunity‐associated genes to be low (B) or high (C) in WT tumors. *N* = 3 tumors per genotype. (A) Log_2_ fold change (x‐axis) and the negative log_10_ (*P*.Value) (y‐axis) is displayed, the blue line indicating *P* = 0.05 (y‐axis), points above the line, *P* < 0.05. The dashed red lines indicate the log_2_ fold change cutoff of −0.25 and 0.25, respectively (x‐axis). Green dots represent genes that display both statistical significance (*P* < 0.05) and fold changes −0.25 and 0.25, respectively. Selected genes regulated by TNC are marked in red (type I interferon response) or pink (*Cxcl12*) dot.D, EInfiltration of F4/80^+^ macrophages determined by flow cytometry. (WT, *N* = 10, TNCKO, *N* = 7 tumors), *P = 0.0304, unpaired *t*‐test and iNOS^+^ macrophages (WT, *N* = 9, TNCKO, *N* = 7 tumors) *P = 0.0197, unpaired *t*‐test. Mean ± SEM. Source data are available online for this figure.

**Figure EV1:**
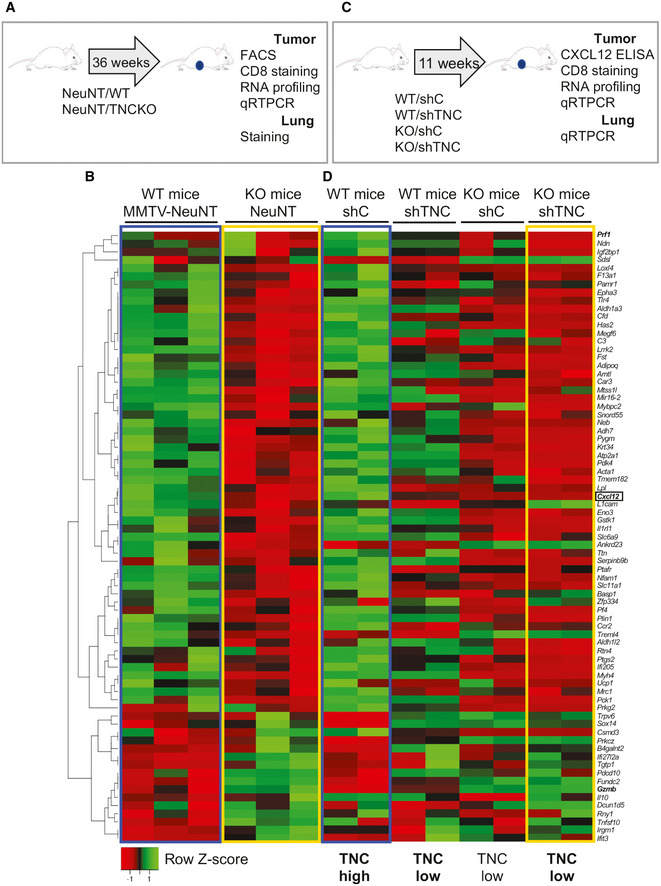
Loss of the TNC protein in both cancer and stromal cells impacts tumor gene expression Schematic depiction of the experimental setup in NeuNT mice with a WT or TNCKO background.Juxtaposed heatmaps representing RNA chip analysis data of MMTV‐NeuNT tumors (WT, TNCKO; *N* = 3).Schematic depiction of the experimental setup in the NT193 grafting model followed by analysis of the tumor and the lungs as indicated. In the grafting experiments, four conditions were used where shC or shTNC tumor cells (sh1, sh2) were engrafted into the mammary gland of a WT or TNCKO (KO) host giving rise to TNC‐high (WT/shC) and TNC‐low tumors (WT/shTNC, KO/shC, KO/shTNC).Juxtaposed heatmaps representing the RNA sequencing results from NT193 tumors (WT and TNCKO mice injected with shC or sh2TNC cells; *N* = 2). Conditions further used in the study are shown in bold as TNC high and TNC low, respectively. Note, juxtaposition shows similarities in the pattern of gene expression between TNC‐high (MMTV‐NeuNT/WT, WT/shC) tumors (blue squares) (B) and TNC‐low (MMTV‐NeuNT/KO, WT/shTNC, KO/shTNC) tumors (yellow squares) (D).

To investigate how TNC impacts tumor growth, we used the MMTV‐NeuNT (NeuNT) model on a wild‐type (WT) and TNC knockout (KO) background. In this model, we have previously shown that TNC increased tumor onset and lung metastasis (Sun *et al,* 2019). We compared overall gene expression in tumors isolated from WT and TNCKO mice by an Affymetrix chip array. This analysis revealed 2,260 genes to be differently regulated between tumors of both genotypes (*P* < 0.05) (Fig 1A–C, Appendix Table S1). Gene ontology analysis showed differential expression of several genes including 136 immune‐related genes (75 downregulated (Fig 1B) and 61 upregulated (Fig 1C)) and reduced expression of type I interferon (IFN) responsive genes in WT compared to TNCKO tumors (Figs 1A and EV1A, B, Appendix Fig S1A and B, Appendix Table S1). We also noticed that more than 10 *Mhc type II* molecules and chemokines such as *Cxcl12* were higher and lower in the absence of TNC, respectively, suggesting an impact of TNC on tumor immunity (Fig 1B and C). By flow cytometry, we investigated the immune cell infiltrate and observed that F4/80^+^ and iNOS^+^ macrophages were more and less abundant in WT than TNCKO tumors, respectively (Fig 1D and E). Moreover, T cell subtype numbers and in particular CD8 T cells (CD44^+^ and PD‐1^+^) were in general very low in these late‐stage tumors with no difference between genotypes (Appendix Fig S1C–F). Interestingly, the CD8 T cell activation marker *Perforin* and several T cell‐regulating genes were higher in TNCKO tumors, indicating that TNC impacts CTL function (Figs 1B and EV1B, Appendix Fig S1G).

### TNC promotes stromal CD8 TIL enrichment and enhances survival of tumor cells in the syngeneic NT193 breast cancer grafting model

**Figure EV2:**
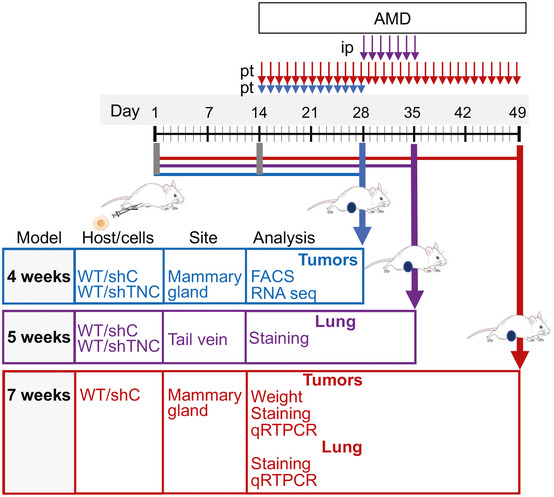
Experimental setup of the CXCR4 inhibition experiments in the NT193 grafting model Experimental setup for the 4, 5, and 7 weeks protocols. 4‐week model: WT mice were engrafted with shC or shTNC tumor cells in the mammary gland followed by AMD treatment (5 mg/kg/day, peritumoral (pt)) for 2 weeks (week 2–4) before sacrifice and investigation of the primary tumor by flow cytometry and RNA‐seq (WT/shC and WT/shTNC). 5‐week model: WT mice were iv engrafted with shC or shTNC tumor cells for 4 weeks followed by AMD treatment (7.5 mg/kg/day, ip) for 1 week (week 5) before sacrifice and analysis of the lungs by tissue staining. 7‐week model: WT mice were engrafted with shC tumor cells into the mammary gland followed by AMD treatment (5 mg/kg/day, pt) for 5 weeks (week 2–7) before sacrifice and investigation of the tumor (weight, tissue staining, qRT–PCR) and the lungs (tissue staining, qRT–PCR).

Due to poorly abundant CD8 TIL in late‐stage NeuNT tumors (confirmed by immunofluorescence staining (IF), Appendix Fig S1C and S2A), we decided to use our novel syngeneic orthotopic NT193 grafting model with more numerous CD8 TIL (Deligne *et al,* 2020). In the previous study, the TNC‐low condition had some residual TNC that was provided by the host. Here, we engrafted *Tnc* knockdown (shTNC) NT193 tumor cells into a TNCKO host to obtain a close to TNC‐negative condition where indeed TNC is almost undetectable (Sun *et al,* 2019). For some experiments, it is relevant to use a TNCKO host to mimic the TNC‐negative condition as host TNC plays a role in promoting lung metastasis by impacting tumor cell survival and plasticity in the blood vessel invasions (BVI), an important precursor of metastasis (Siegel *et al,* 2003; Sun *et al,* 2019). As illustrated in Fig EV1C and D, we compared tumors with high and low TNC levels. In the TNC‐high condition control (shC), tumor cells were engrafted into a WT host (WT/shC). For TNC‐low tumors, a WT or TNCKO host was engrafted with shTNC cells, giving rise to WT/shTNC or KO/shTNC tumors, respectively. We used different time points (4, 5, 7, and 11 weeks) and grafting methods (iv, orthotopic) as indicated and outlined in Fig EV2. We investigated gene expression in all grafted tumors by RNA‐seq analysis and compared gene expression levels to that in NeuNT tumors (Fig EV1B–D). This comparison revealed clear differences between tumors and showed that in tumors with lowered TNC, gene expression was clearly different to tumors with abundant TNC. Moreover, similarities in gene expression were seen in the TNC‐high group (NeuNT‐WT, WT/shC) and TNC no/low group (NeuNT‐KO, WT/shTNC, KO/shTNC), respectively (Fig EV1B and D, Appendix Table S2).

**Figure 2:**
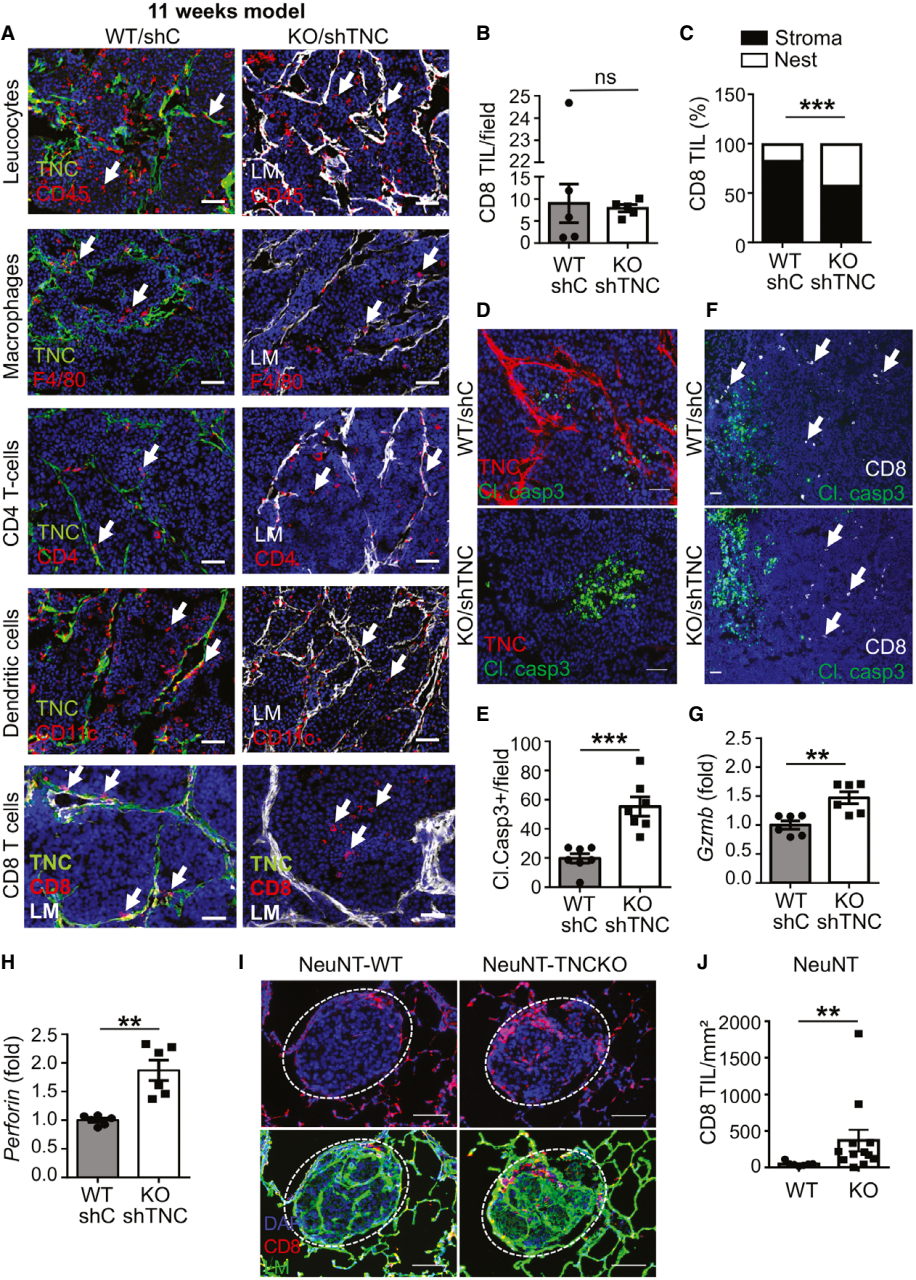
Impact of TNC on CD8 TIL in the syngeneic NT193 breast cancer model ARepresentative staining images (*N* = 5) of tissue from TNC‐high (WT/shC) and TNC‐low (KO/shTNC) tumors (*N* = 5 per condition) with the indicated antibodies. Arrows point at the respective immune subtype. Scale bar, 50 µm.B, CQuantification of CD8 TIL upon tissue staining as total number per field (B) and in the stroma or tumor cell nest (C), expressed as relative ratio, ***P = 0.0002, Fisher’s exact test and in TNC‐high (*N* = 5) and TNC‐low tumors (*N* = 5). 3 sections per tumor and 8 random fields per section. Mean ± SEM.D, EApoptosis measurement upon tumor staining for cl. casp3 (D) and signal quantification per field (E) in TNC‐high and TNC‐low tumors (*N* = 7 per condition), 2 sections per tumor, 5 random fields. ***P = 0.0006, Mann–Whitney test, mean ± SEM. Scale bar, 50 µm.FRepresentative staining images (*N* = 5 tumors, 3 sections per tumor) for the indicated molecules. Scale bar, 50 µm. Arrows point at CD8 TIL.G, H
*Gzmb* and *Perforin* expression as determined by qRT–PCR. *N* = 6 tumors per condition. **P = 0.0022 for both graphs, Mann–Whitney test. Mean ± SEM.I, JDetection (I) and quantification (J) of CD8 TIL in lung metastasis of NeuNT‐WT and TNCKO mice (*N* = 3 mice, *n* = 7 and 12 lung metastases, respectively). Scale bar, 100 µm. **P = 0.0097, Mann–Whitney test. Mean ± SEM. Source data are available online for this figure.

We previously noticed that macrophages were abundant in the stroma of TNC‐high (WT/shC) tumors (Deligne *et al,* 2020). Now we wondered whether TNC influenced the localization of macrophages and other immune subtypes which we addressed by tissue staining in the 11‐week model (Fig EV2) and observed that CD45^+^ leukocytes were present inside the stroma as well as in the tumor cell nests. As previously seen for macrophages (F4/80) (Deligne *et al,* 2020), also CD4 T cells and CD11c myeloid/dendritic cells were present inside the tumor nests and the stroma with no apparent difference between genotypes (Fig 2A). This was different for CD8 TIL. Whereas CD8 TIL were similarly abundant in TNC‐high and TNC‐low (KO/shTNC) tumors (Fig 2B), they resided predominantly inside the stroma of TNC‐high tumors, in contrast to TNC‐low (KO/shTNC) tumors where CD8 TIL more frequently invaded the tumor cell nests (Fig 2A and C). As CD8 TIL were found in the stroma, we asked what contribution tumor and stromal TNC has on this retention. Therefore, we quantified CD8 TIL in WT/shTNC tumors and compared results to KO/shTNC and WT/shC tumors which revealed a tendency of more CD8 TIL infiltration when tumor cell‐derived TNC was lowered (WT/shTNC). However, tumor nest infiltration of CD8 TIL was only significantly higher in the absence of host TNC (KO/shTNC). This result clearly shows that host TNC is important in retaining CD8 TIL (Appendix Fig S2B). To address whether CD8 TIL in the tumor nests had an impact on survival, we stained for cleaved caspase 3 (Cl. casp3) and saw more apoptosis in TNC‐low (KO/shTNC) than in TNC‐high tumors (Fig 2D and E). Costaining for CD8 and Cl. casp3 revealed no colocalization, but vicinity, suggesting a paracrine mechanism (Fig 2F). In support, we saw higher expression of *Granzyme B* (*Gzmb)* and *Perforin* (secreted by activated CD8 TIL (Kelso *et al,* 2002)) in TNC‐low (KO/shTNC) than TNC‐high tumors (Fig 2G and H). As TNC is expressed in the lungs of NT193 tumor mice (Sun *et al,* 2019), we addressed whether TNC also impacted CD8 TIL in lung metastasis by determining the abundance of CD8 TIL by tissue staining. We noticed a higher infiltration of CD8 TIL in lung metastases from WT than TNCKO mice (Fig 2I and J) correlating with higher metastasis in WT mice (Sun *et al,* 2019). Altogether, our results point at CD8 TIL localization and function to be regulated by TNC.

### Tenascin‐C upregulates and binds CXCL12

**Figure 3:**
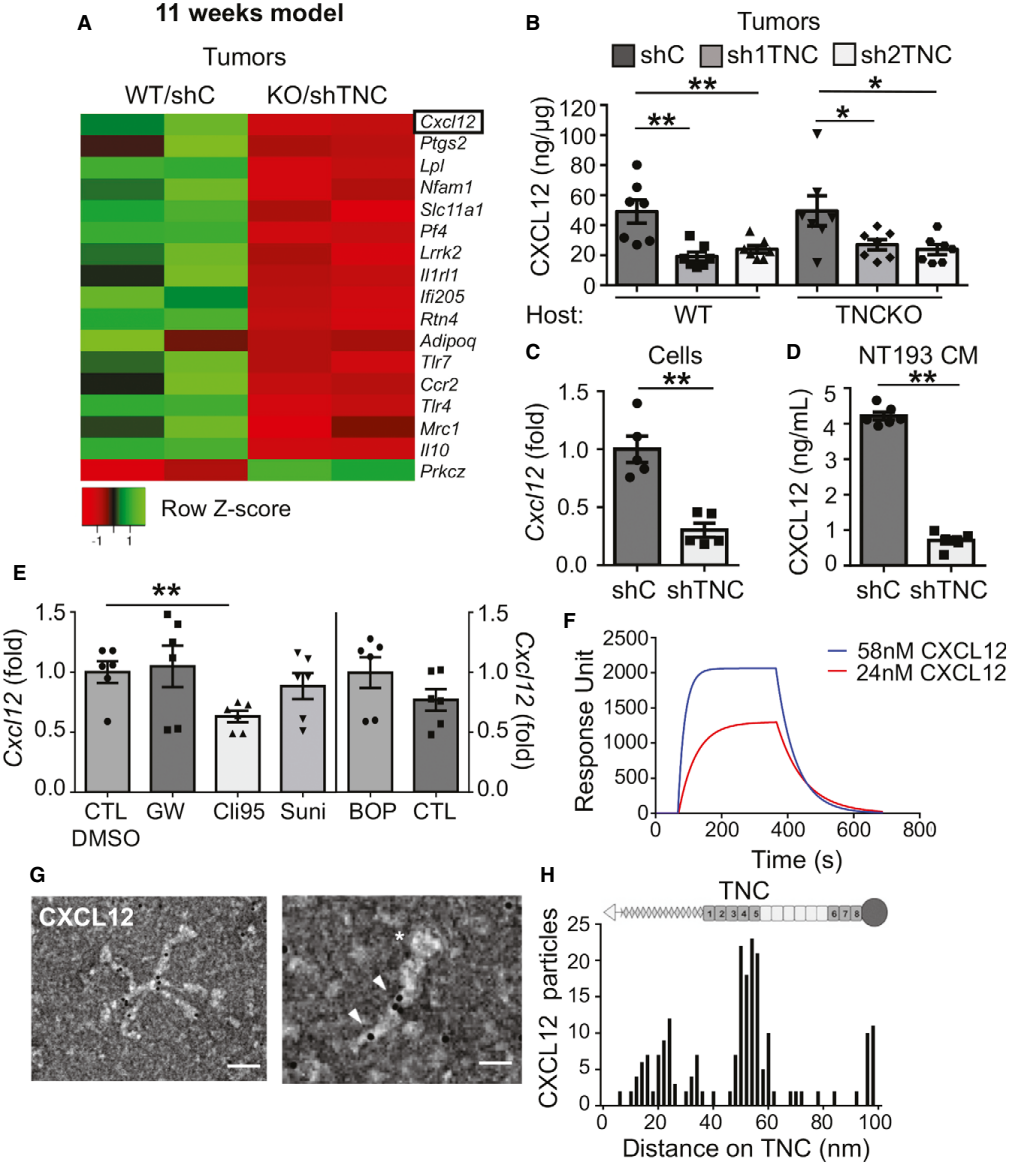
CXCL12 expression and binding to TNC AHeatmap representation of the most 17 deregulated Panther identified immune‐related genes in TNC‐high (WT/shC) and TNC‐low (KO/shTNC) tumors (*N* = 2 tumors each).BCXCL12 protein levels in NT193 tumors (ELISA). *N* = 7 tumors per group, **P = 0.0035 (WT host, shC versus sh1TNC), **P = 0.0070 (WT host, shC versus sh2TNC), *P = 0.0379 (TNCKO host, shC versus sh1TNC), *P = 0.0262 (TNCKO host, shC versus sh2TNC), Mann–Whitney test. Mean ± SEM.C
*Cxcl12* levels in cultured tumor cells (qRT–PCR). *N* = 5 independent experiments, **P = 0.0079, Mann–Whitney test. Mean ± SEM.DCXCL12 protein levels (ELISA) in conditioned medium (CM) from cultured tumor cells, *N* = 6 independent experiments, **P = 0.0022, Mann–Whitney test. Mean ± SEM.EAnalysis of *Cxcl12* (qRT–PCR) in tumor cells upon pretreatment with the indicated inhibitors for 45 min (GW, BOP, DMSO, and PBS as control, respectively) or 60 min (Sunitinib) and 6 h (Cli95) (6 h DMSO as control) before incubation with TNC for 24 h. *N* = 6 independent experiments, **P = 0.0022, Mann–Whitney test. Mean ± SEM.F–HCXCL12 binding to TNC as determined by SPR measurement (F) and negative EM imaging (G), followed by quantification (H). (G) scale bars, 100 nm (left), 50 nm (right), the arrowheads point at gold labeled CXCL12 and the asterisk points at the fibrinogen globe of the murine TNC monomer. (H) Representation of TNC monomer, oligomerization domain (triangle) to form hexamers as seen in (G), FNIII repeats (gray boxes, constant domains, white boxes, alternative domains), fibrinogen like domain (circle). Representative result (3 independent experiments). Source data are available online for this figure.

**Figure EV3:**
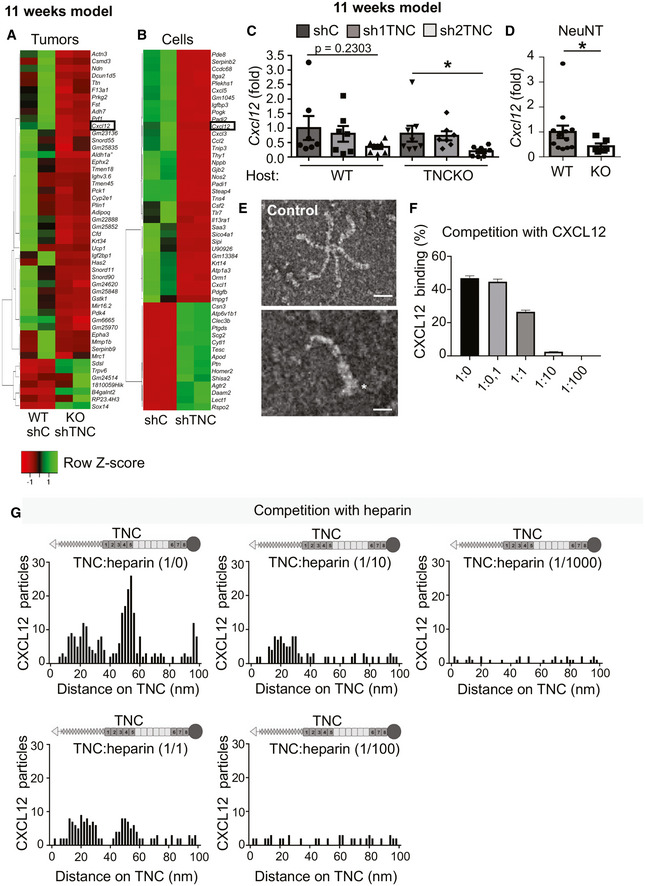
CXCL12 expression and binding to TNC A, BRNA‐seq gene expression results represented as heatmap for the fifty most deregulated genes in TNC‐high (WT/shC) and TNC‐low (KO/shTNC) tumors (*N* = 2 tumors per condition), 11‐week model (A) and in shC and shTNC cells (*N* = 2) (B).CCXCL12 mRNA levels in NT193 tumors, 11‐week model (*N* = 7 tumors for WT/shC, WT/sh1TNC, TNCKO/sh1TNC, and TNCKO/sh2TNC conditions, *N* = 8 tumors for WT/sh2TNC and TNCKO/shC conditions), *P = 0.0205, Mann–Whitney test. Mean ± SEM.DCXCL12 mRNA levels in MMTV‐NeuNT/WT compared to TNCKO tumors as determined by qRT–PCR (*N* = 13 and 6 tumors, respectively), *P = 0.0123, Mann–Whitney test. Mean ± SEM.E–GNegative EM analysis of binding of unlabeled beads to TNC (E) and upon binding of CXCL12‐adsorbed gold beads upon incubation with unlabeled CXCL12 (F) and heparin (G) at the indicated molar ratios to TNC. Asterisk points at fibrinogen globe. Scale bars, 100 nm (top), 50 nm (bottom), (E). Representation of TNC monomer, oligomerization domain (triangle) to form hexamers as seen in (E) FNIII repeats (gray boxes, constant domains, white boxes, alternative domains), fibrinogen like domain (circle). Representative result (3 independent experiments, *n* = 500 TNC molecules each). Mean ± SEM.

To gain insight into the underlying mechanism, we consulted our RNA‐seq results which revealed 1,502 genes to be differently expressed, including *Cxcl12*. Here, we focused on CXCL12, a known regulator of tumor immunity (Okabe *et al,* 2005; Zhang *et al,* 2019) that was more expressed in TNC‐high in comparison to TNC‐low (WT/shTNC, KO/shTNC) tumors and in cultured tumor cells which we confirmed by qRT–PCR and ELISA (Figs 3A–C and EV3A and B, Appendix Table S2). Tumors derived from shC‐engrafted tumor cells had the highest CXCL12 levels irrespective of the host, and shTNC‐engrafted tumor cells expressed less CXCL12 altogether pointing at the tumor cells as major source of CXCL12 (Figs 3B and ). We confirmed higher *Cxcl12* levels also in NeuNT‐WT than TNCKO tumors (qRT–PCR) and in the supernatant (conditioned medium, CM) of shC in comparison to shTNC cells (qRT–PCR, ELISA) (Figs 3C and D, and EV3D). Next, we addressed how TNC induced *Cxcl12* by using inhibitors according to established protocols for tyrosine kinase inhibitors (sunitinib), integrin α4β1 and α9β1 (BOP), TGFβ receptor 1 (TGFβRI, GW788388), and Toll‐like receptor 4 (TLR4, Cli95) on tumor cells stimulated with TNC. In contrast to BOP, GW, and sunitinib that did not affect *Cxcl12* expression, Cli95 reduced TNC‐induced *Cxcl12* demonstrating that TNC regulates CXCL12 expression through the known TNC receptor TLR4 like other molecules (Fig 3E; Midwood *et al,* 2009; Spenlé *et al,* 2020).

Next, we used tissue staining to assess the relative distribution of CXCL12 within the tumor. We noticed higher CXCL12 expression in the tumor nests than in the stroma as also documented in human tumors (Toullec *et al,* 2010; Lefort *et al,* 2017). In contrast, TNC was highly expressed in the stroma but not in the tumor nest which we confirmed by using 3 different antibodies (Appendix Fig S2B and C). We cannot explain how CXCL12 expression is regulated in the tumor nests. TNC may play a role by inducing CXCL12 in the stroma to either bind to TNC and/or diffuse into the tissue. CXCL12 gradients in tumors have been described but how they are established is not well understood (Spinosa *et al,* 2017).

TNC interacts with many molecules (De Laporte *et al,* 2013; Midwood *et al,* 2016; Spenlé *et al,* 2020). Therefore, we asked whether TNC potentially binds CXCL12. Indeed, by surface plasmon resonance measurement, CXCL12 was found to bind TNC in a range of 790 nM (35‐fold lower than binding to CXCR4; Richter *et al,* 2014; Fig 3F). Moreover, by negative staining electron microscopy, we confirmed CXCL12 binding to TNC. CXCL12 labeled with colloidal gold particles bound to TNC predominantly inside the fibronectin type III (FNIII) domains, including the 5^th^ FNIII repeat that was previously shown to bind several other molecules (De Laporte *et al,* 2013; Spenlé *et al,* 2020). Additional but much lower binding was also seen in the EGFL repeats and the fibrinogen like globe (FBG) whereas control gold particles (not chemokine coupled), or EGF‐ or BSA‐coupled gold particles (Spenlé *et al,* 2020), did not bind TNC (Figs 3G and H, and EV3E). Moreover, binding of CXCL12‐coupled beads to TNC was competed with free CXCL12 and with increasing concentrations of heparin implying interactions via charged moieties (Fig  EV3F and G).

### CXCL12 immobilizes CD8 T leukocytes in a CXCR4‐dependent manner

We investigated whether TNC influenced expression of the CXCL12 receptors CXCR4 and/or CXCR7 (Bleul *et al,* 1996; Balabanian *et al,* 2005) and observed that *Cxcr4* levels were higher in TNC‐low (WT/shTNC, KO/shTNC) tumors and in cultured tumor cells upon TNC knockdown, revealing an opposite regulation than *Cxcl12* by TNC (Appendix Fig S3A). No difference in *Cxcr7* mRNA levels was seen in cultured tumor cells in dependence of TNC (Appendix Fig S3B).

**Figure 4:**
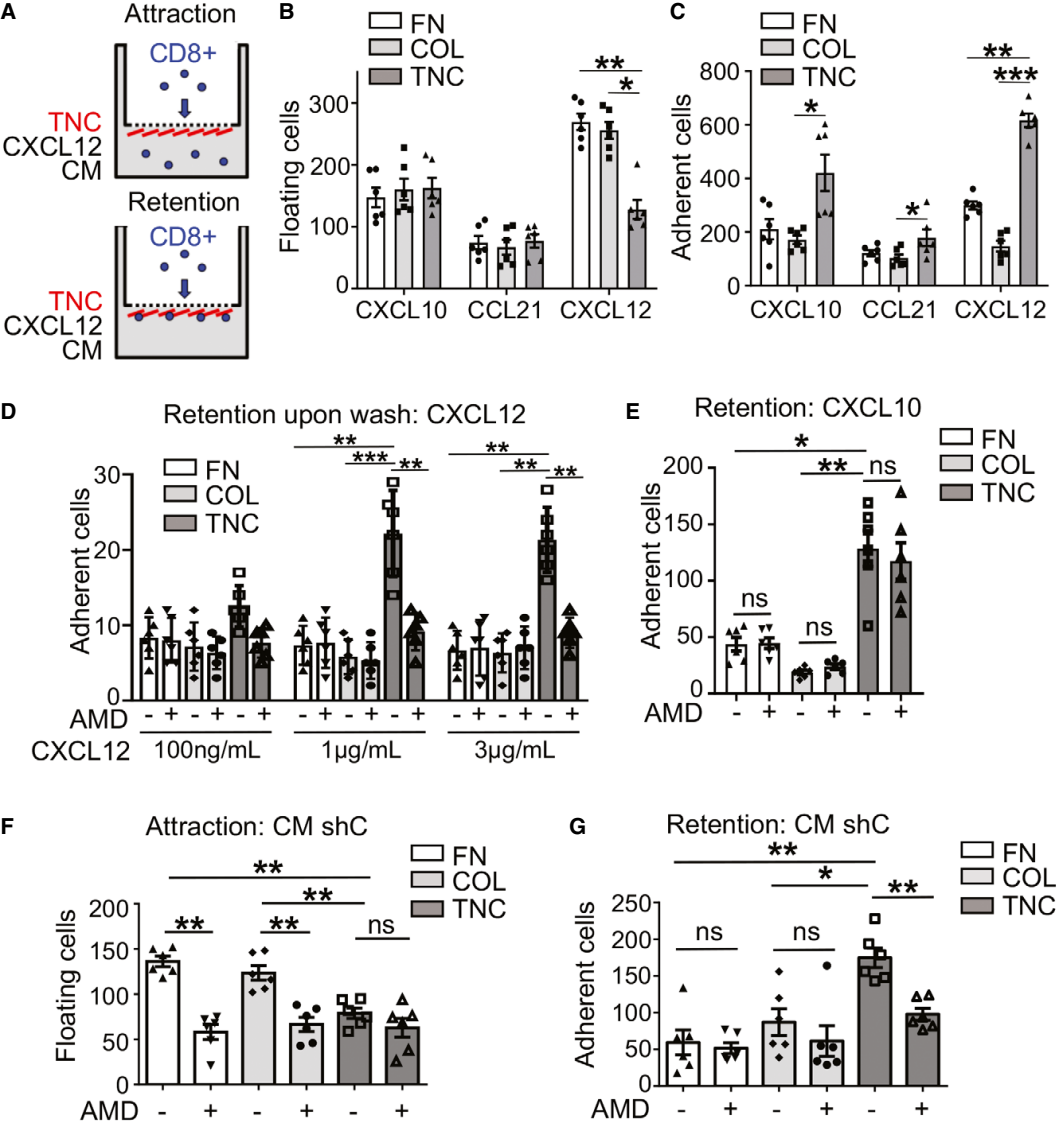
Chemoattraction of CD8 T leukocytes by TNC and CXCL12 ABoyden chamber experiment to measure attraction and retention of CD8 T cells toward factors in the lower chamber. Cells in the lower chamber were measured by flow cytometry (attraction) or on the matrix coatings (retention) by counting Dapi‐stained nuclei 5 h after plating.B–GAttraction (B, F) and retention (C, D, E, G) were determined toward coatings with collagen I (COL), fibronectin (FN), or TNC in the presence of CXCL10, CCL21, CXCL12 (B, C), CXCL12 (D), CXCL10 (E), or CM from shC cells (CM:shC) and upon AMD3100 (AMD, 5 µg/ml) (D–G). (D) CXCL12 (indicated concentrations) was added overnight at 4°C during the coating followed by washing (2 times with PBS) and plating CD8 T cells for 5 h before fixation and counting Dapi‐stained nuclei. Three independent experiments in duplicates, Mann–Whitney test, ns > 0.05; *P < 0.05; **P < 0.01; ***P < 0.005. Mean ± SEM. Source data are available online for this figure.

To address whether CXCL12 regulates CD8 T cell function, we compared transwell migration (attraction or retention) of CD8 T cells toward defined substrata, collagen I (COL), fibronectin (FN), or TNC together with CXCL12 and CXCL10 or CCL21, respectively, as previously described (Fig 4A, Spenlé *et al,* 2020). CXCL12 attracted more CD8 T cells than CXCL10 or CCL21. In contrast to FN or COL, CD8 T cell attraction by CXCL12 was surprisingly largely reduced by TNC (Fig 4B). However, we found more CD8 T cells to be immobilized on TNC than on the other substrata, with a more potent effect of CXCL12 than CXCL10 or CCL21 (Fig 4C). As TNC binds CXCL12, potentially generating an adhesive substratum, we washed the coating after incubation with CXCL12 and observed that TNC plus CXCL12 indeed retained CD8 T cells whereas the other substrata did not. This effect was CXCR4 dependent as AMD3100 (AMD), a CXCR4 inhibitor, blocked CXCL12‐ but not CXCL10‐dependent CD8 T cell retention (Fig 4D and E).

To address whether CXCL12 is the major chemoattractant by which TNC immobilizes CD8 T cells, we used conditioned medium (CM) from cultured tumor cells in the migration and retention assay, respectively. In contrast to CM from shTNC cells (low CXCL12) that was poorly cell attracting, CM from shC cells (high CXCL12) strongly attracted CD8 T cells toward the FN and COL but less to the TNC coating (Fig 4F). As for CXCL12, also with the CM from shC cells more CD8 T cells adhered on TNC than on the other substrata which were not seen with CM from shTNC cells (Fig 4G, Appendix Fig S3C and D). Finally, the TNC retention effect of the CM was abolished with AMD, supporting a role of CXCR4 signaling in CD8 T cell attraction and retention by the secretome from the tumor cells (Fig 4F and G, Appendix Fig S3C and D).

To rule out other potential effects of AMD on CD8 T cells, we investigated CD8 T cell survival, proliferation, and function upon activation by an anti‐CD3 antibody and inhibition of CXCR4. In contrast to *Bax* and *Bcl‐w* that were unchanged (determined by qRT–PCR), expression of apoptosis inhibitory *Bcl‐2* was increased by anti‐CD3, yet not affected by AMD (Appendix Fig S3E–G). With an MTS assay, proliferation was found to be increased with anti‐CD3, which was not changed by AMD (Appendix Fig S3H). Moreover, upon incubation with the anti‐CD3 antibody, CD8 T cells upregulated expression of *Ifnγ*, *Perforin,* and *Gzmb* which again was not altered by AMD (Appendix Fig S3I–K).

Finally, by higher resolution tissue staining of TNC‐high tumors, we noticed a punctate CXCL12 signal in the stroma, importantly colocalizing with TNC and CD8 TIL, altogether suggesting that the described chemoretention by TNC/CXCL12 might be relevant for tethering CD8 TIL in the tumor stroma (Appendix Fig S3L).

### Inhibition of CXCR4 restores CD8 TIL tumor nest infiltration triggering tumor cell death

**Figure EV4:**
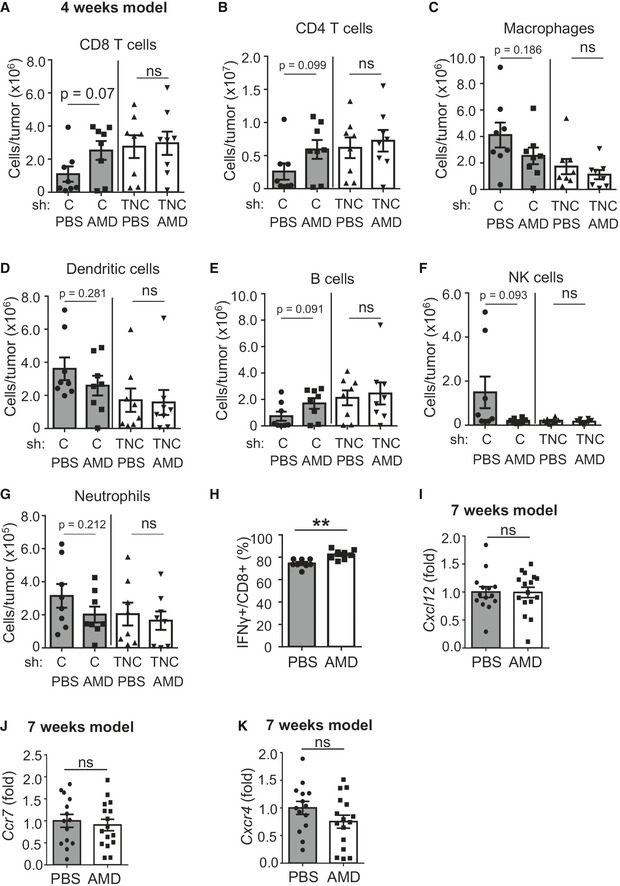
Impact of CXCR4 on the tumor immune cell infiltrate and gene expression in the tumors A–G4‐week model. Abundance of the indicated immune cell subtypes per tumor as determined by flow cytometry. *N* = 8 tumors per condition. ns: *P* > 0.05, Mann–Whitney test. Mean ± SEM.H4‐week model. IFNγ expression in MACS isolated CD8 T leukocytes as determined by flow cytometry. *N* = 8 tumors per group, **P = 0.0015, unpaired *t*‐test. Mean ± SEM.I–K7‐week model. Expression of the indicated molecules in tumors as determined by qRT–PCR. *N* = 14 (PBS), *N* = 16 (AMD) tumors. ns: *P* > 0.05, unpaired *t*‐test. Mean ± SEM.

We investigated by flow cytometry whether TNC influenced CD8 TIL function *in vivo* through CXCL12, by blocking CXCR4 with AMD (for 2 weeks) in tumor‐bearing mice in the 4‐week model (Figs EV2 and EV4A–H). We observed a tendency toward more CD8 TIL in TNC‐high tumors upon AMD treatment (*P* = 0.07). This was different to TNC‐low (WT/shTNC) tumors with no change in CD8 TIL levels (Fig EV4A). Whereas a tendency toward more CD4 and B cells and less macrophages, DC, NK cells, and neutrophils was seen in the TNC‐high tumors upon CXCR4 inhibition, respectively, there was clearly no difference in TNC‐low tumors suggesting that CXCR4 inhibition may have an impact on the immune cell infiltrate in the presence but not in the absence of TNC (Fig EV4A–G).

**Figure 5:**
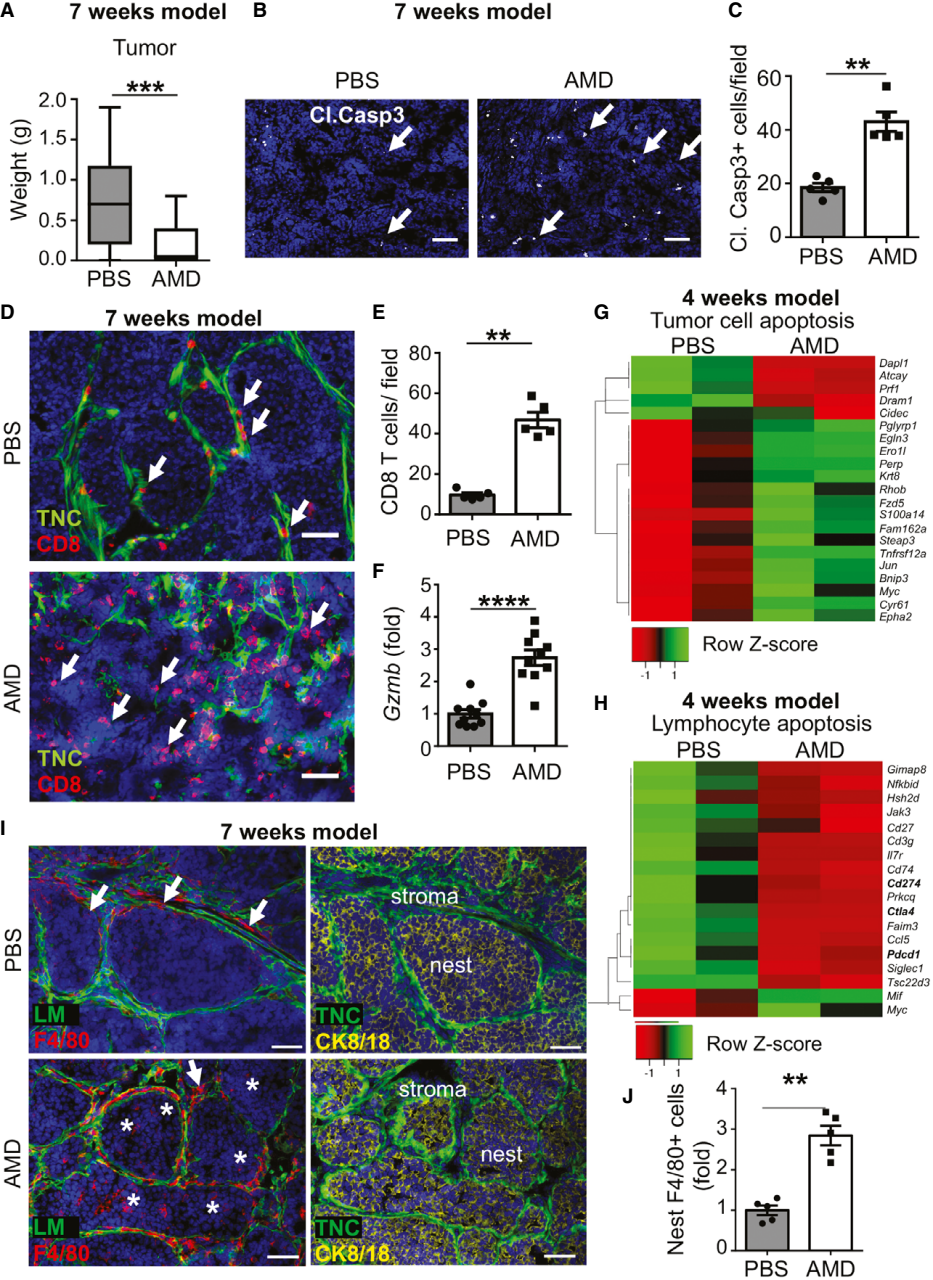
Inhibition of CXCR4 induces infiltration of CD8 leukocytes and macrophages and reduces tumor cell death and tumor growth (7‐week model) AGrowth of TNC‐high (WT/shC) tumors upon cell engraftment and 5 weeks AMD treatment. *N* = 20 tumors per condition, ***P = 0.0007, unpaired *t*‐test. The central band represents the median, the ends of the box the first and the third quartiles, and the whiskers reach from each quartile to the minimum or maximum.B, CImaging and signal quantification upon staining for cl. casp3 and DAPI in PBS‐ and AMD‐treated tumors, *N* = 5 tumors per condition, 2 sections per tumor, 5 random fields, **P = 0.0079, Mann–Whitney test. Mean ± SEM. Scale bar, 50 µm, arrows point at cl. Casp3.D, ECD8 TIL abundance and localization in PBS‐ and AMD‐treated tumors, arrow points at CD8 TIL, scale bar, 50 µm (D). (E) Quantification of CD8 T cells per randomly chosen fields. *N* = 5 tumors per condition, 2 sections per tumor, 5 random fields, **P = 0.0079, Mann–Whitney test. Mean ± SEM.F
*Gzmb* levels in PBS‐ and AMD‐treated tumors. *N* = 10 tumors, ****P < 0.0001, unpaired *t*‐test. Mean ± SEM.G, HHeatmap of tumor cell apoptosis‐associated (G) and lymphocyte apoptosis‐associated (H) genes differentially expressed in PBS‐ and AMD‐treated TNC‐high (WT/shC) tumors, *N* = 2.I, JAbundance and quantification of F4/80^+^ macrophages in PBS‐ and AMD‐treated tumors, (I) staining for the indicated molecules, (arrow, macrophages in the stroma; asterisk, macrophages in the tumor nest), (J) quantification of F4/80^+^ cells inside the tumor nest. *N* = 5 tumors per condition, 2 sections per tumor, 5 random fields, **P = 0.0079, Mann–Whitney test. Mean ± SEM. Scale bar, 50 µm. Source data are available online for this figure.

We considered that in the 4‐week model, the full effect of AMD may not have developed yet; therefore, we used other AMD treatment protocols (Azab *et al*, 2009, Domanska *et al,* 2012; Fig EV2). In the 7‐week model, we observed a reduced tumor weight in AMD‐treated mice, indicating that inhibition of CXCR4 reduced tumor growth (Fig 5A). In accordance, we observed more apoptosis (quantification of the cl. casp3 staining signal) in the tumors upon CXCR4 inhibition (Fig 5B and C). Next, we addressed CD8 TIL tumor infiltration by tissue staining and observed more CD8 TIL in AMD‐treated tumors (Fig 5D and E) that likely were active as *Gzmb* and *IFNγ* levels were higher upon CXCR4 inhibition (Figs 5F and EV4H). To rule out other factors, by qRT–PCR, we determined *Cxcl12*, *Cxcr4,* and *Cxcr7* expression and did not see differences between PBS‐ and AMD‐treated groups (Fig EV4I–K).

**Figure EV5:**
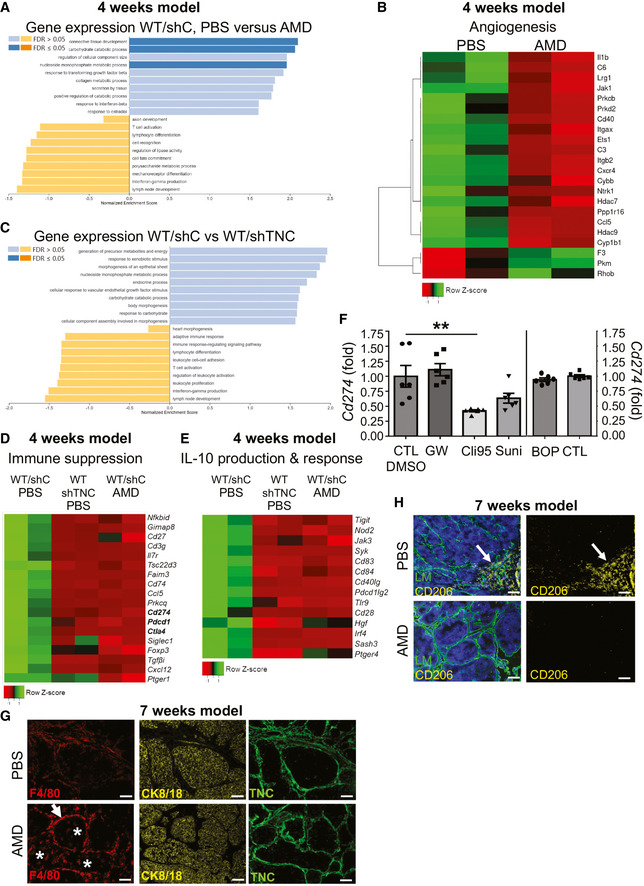
Impact of CXCR4 on gene expression and abundance of macrophages A–E4‐week model. Gene set enrichment analysis of differentially expressed genes in TNC‐high (WT/shC) tumors treated with PBS or AMD (A, B, D, E) and in WT/shC and WT/shTNC tumors treated with PBS (C, D, E). (A, C) False discovery rate (FDR) > 0.05 and ≤ 0.05. *N* = 2 tumors per group. RNA‐seq gene expression results represented as heatmap for genes that belong to the angiogenesis pathways (*P* = 2.31 × 10^−9^) (B), immune suppression (*P* = 7.87 × 10^−7^) (D), IL‐10 production and response signaling (*P* = 8.12 × 10^−8^) (E) according to the Panther software and Geneontology functional database.FAnalysis of *Cd274* levels by qRT–PCR in shC tumor cells upon treatment with the indicated inhibitors, *N* = 6 independent experiments, **P = 0.0022, Mann–Whitney test. Mean ± SEM.G, H7‐week model. Staining of tumor tissue (WT/shC) for the indicated molecules. Scale bar, 50 µm. *N* = 5 tumors, *n* = 2 slides per condition. Arrows point at F4/80^+^ (G) and CD206^+^ cells (H). Asterisks point at the F4/80^+^ cells localized in the tumor cell nest (G).

To gain more insight into the underlying mechanisms, we compared gene expression in PBS with AMD‐treated conditions (TNC‐high) in the 4‐week model, by RNA‐seq analysis, and noticed profound differences between the groups. 1,287 genes were deregulated with 269 up‐ and 1,018 downregulated upon CXCR4 inhibition (Appendix Table S3). The large majority of genes in a signature associated with tumor cell apoptosis were upregulated upon AMD treatment (Fig 5G). In contrast, signatures associated with angiogenesis, lymphocyte apoptosis, immune suppression, and IL10 production and response were downregulated upon CXCR4 inhibition in comparison to the PBS control (Figs 5H and EV5A–E). Also, CD8 T cell inhibitory *Pdcd‐1, Cd274,* and *Ctla4* were reduced upon AMD treatment (Figs 5H and EV5D). As TNC impacted expression of *Cd274,* we investigated gene expression with inhibitors as described (Fig 3E) and found that *Cd274* was reduced in the tumor cells with the TLR4 inhibitor Cli95 yet not with the other inhibitors (Fig EV5F). Moreover, upon CXCR4 inhibition, several genes regulating lymphocyte differentiation, T cell activation, and IFNγ production were deregulated mimicking gene expression of tumors with low TNC (WT/shTNC) (1,753 deregulated genes, 470 up‐ and 1,283 downregulated), altogether suggesting that immunity is largely regulated by CXCR4 signaling in these tumors in a TNC‐dependent manner (Fig EV5D and E, Appendix Table S4).

As we had previously seen that TNC promoted an M2 macrophage phenotype (Deligne *et al,* 2020), we wondered whether CXCR4 inhibition potentially impacted macrophages. By staining of TNC‐high tumor tissue from the 7‐week model for F4/80, we observed more activated macrophages upon AMD treatment compared to PBS. More importantly, largely present in control tumors, macrophages were even more abundant upon CXCR4 inhibition, now clearly infiltrating the tumor nests (Figs 5I and J, and EV5G). By tissue staining for CD206, we confirmed M2 macrophage infiltration of TNC‐high tumors (Deligne *et al,* 2020) which we noticed to be abolished with AMD as no M2 macrophages were detectable anymore (Fig EV5H).

Thus, we conclude that TNC plays an important role in regulating macrophages and CD8 TIL through CXCL12/CXCR4 signaling thereby impairing tumor cell killing and promoting tumor growth.

### Impact of CXCR4 inhibition on lung metastasis

**Figure 6:**
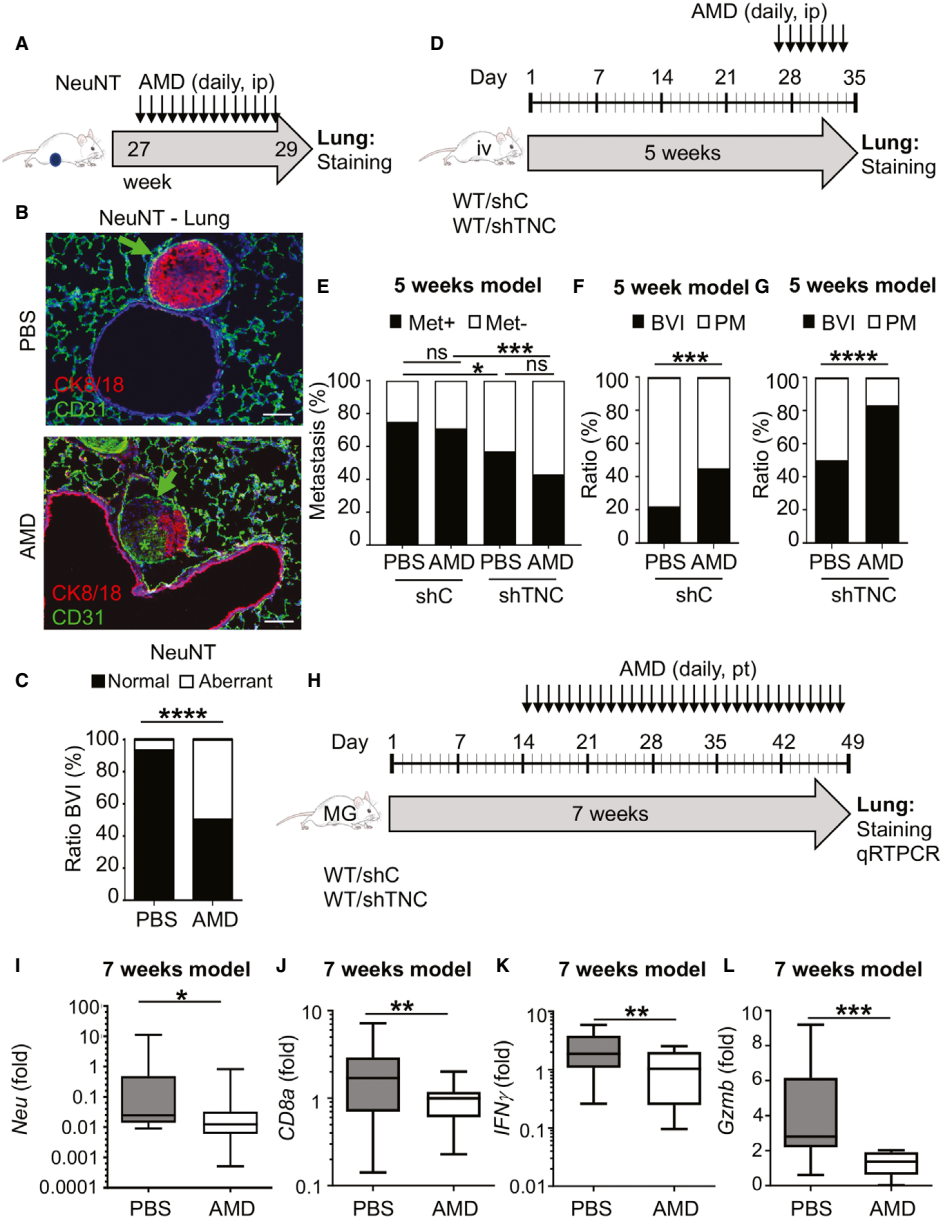
Impact of CXCR4 inhibition on lung metastasis ASchematic depiction of the AMD treatment protocol before sacrifice and analysis of the lungs. Intraperitoneal (ip) injection of 5 mg/kg/day AMD into NeuNT mice (daily for 2 weeks, week 27–29).BIF tissue staining identifying blood vessel invasions (BVI), surrounded by an EC monolayer (arrow) and expanded EC (asterisk), scale bar, 50 µm.CQuantification of BVI with normal (EC monolayer) and aberrant (expanded EC) phenotype expressed as ratio. *N* = 5 mice (PBS), *n* = 3 sections, *n* = 15 BVI, *n* = 7 PM (parenchymal metastasis); *N* = 5 mice (AMD), *n* = 3 sections, *n* = 12 BVI, *n* = 2 PM, ****P < 0.0001, Fisher’s exact test.DSchematic depiction of the AMD treatment protocol before sacrifice and analysis of the lungs. Tail vein (iv) tumor cell‐engrafted mice (WT/shC, WT/shTNC) were ip injected (daily for one week, week 4–5) with AMD 7.5 mg/kg/day. Metastasis assessment in the 5 weeks iv orthotopic grafting model (D–G).ERatio of mice with and without lung metastasis determined by H&E tissue staining. *N* = 8 mice (shC, PBS), *N* = 7 mice (shC, AMD), *N* = 7 mice (shTNC, PBS), *N* = 7 mice (shTNC, AMD). *P* < 0.0001, chi‐square test. Fisher’s exact test was used to compare the conditions pairwise *P < 0.05, ***P < 0.001. *P* = 0.0109 (shC/PBS versus shTNC/PBS), *P* = 0.0001 (shC/AMD versus shTNC/AMD).F, GRatio of BVI and PM in lungs from shC‐engrafted (F) and shTNC‐engrafted (G) mice. *N* = 8 mice (shC, PBS), *n* = 4 BVI, *n* = 13 PM; *N* = 7 mice (shC, AMD), *n* = 15 BVI, *n* = 21 PM; *N* = 7 mice (shTNC, PBS), *n* = 8 BVI, *n* = 9 PM; *N* = 7 mice (shTNC, AMD). *N* = 5 BVI, *n* = 1 PM, ***P < 0.001, ****P < 0.0001. *P* = 0.0009 (shC/PBS versus shC/AMD) and *P* = 0.0001 (shTNC/PBS versus shTNC/AMD), Fisher’s exact test.HSchematic depiction of the AMD treatment protocol before sacrifice and analysis of the lungs. Mice with mammary gland (MG)‐engrafted tumor cells (WT/shC) were daily injected peritumorally (pt) for 5 weeks with 5 mg/kg/day (week 2–7). Metastasis assessment in the 7 weeks orthotopic grafting model (H‐L).I–LqRT–PCR assessment of *Neu* (I), *Cd8a* (J), *Ifnγ* (K), and *Gzmb* (L) by qRT–PCR. PBS‐treated (*N* = 16) and AMD‐treated (*N* = 19) mice, *P = 0.0364, Mann–Whitney test (I), **P = 0.0086 unpaired *t*‐test (J), **P = 0.0055, unpaired *t*‐test (K), ***P = 0.0003 unpaired *t*‐test (L), respectively. The central band represents the median, the ends of the box the first and the third quartiles, and the whiskers reach from each quartile to the minimum or maximum. Source data are available online for this figure.

As CXCR4 signaling is known to promote cell migration and metastasis (Chatterjee *et al,* 2014), we investigated whether CXCR4 inhibition by AMD had an effect on lung metastasis in the spontaneous NeuNT and grafting NT193 models, respectively, as detailed in Fig 6A, D, and H. Due to variable latency and multi‐focal tumor onset, the NeuNT model is poorly suitable for general metastasis assessment. However, upon AMD treatment, we noticed differences in the phenotype of blood vessel invasions (BVI), important precursors of parenchymal metastasis in this model (Fig 6B; Siegel *et al,* 2003; Sun *et al,* 2019). Whereas tumor cells were surrounded by an endothelial cell (EC) monolayer in BVI of PBS‐treated mice (like in untreated mice; Sun *et al,* 2019), we noticed a prominent EC expansion in the BVI of AMD‐treated mice which may impact progression into parenchymal metastasis (Fig 6B and C, Appendix Fig S4A and B; Sun *et al,* 2019).

To address a potential role of CXCR4 signaling on homing and tumor outgrowth in the NT193 model, we engrafted shC or shTNC cells via tail vein (iv) injection into a WT host followed by a daily AMD treatment for one week as previously described (Yang *et al,* 2019) (5‐week model, Fig 6D). By a stereological approach, we assessed lung metastasis (Saupe *et al,* 2013) and observed that the majority of shC‐engrafted mice got metastases, however with no impact of AMD. In contrast, engrafted shTNC cells caused less metastases which again was not affected by AMD, supporting a potential role of TNC in tumor cell homing to the lung as previously described for another model (Fig 6E; Oskarsson *et al,* 2011). We further found that the number and size of metastases was not different in the TNC‐high condition (Appendix Fig S4C and D). Moreover, we noticed prominent round structures with flat nuclei at the rim (representing EC), reminiscent of blood vessel invasions (BVI) in the lungs of iv tumor cell‐engrafted mice (similar to the orthotopic grafting condition (Sun *et al,* 2019)) (Appendix Fig S4E). Upon CXCR4 inhibition, BVI were more abundant in TNC‐high conditions in expense of parenchymal metastasis (PM) (in comparison to PBS controls) (Fig 6F). The PM/BVI ratio was even further reduced upon AMD treatment in the TNC‐low (WT/shTNC) condition suggesting a stronger effect of CXCR4 inhibition in the absence of TNC (Fig 6G). These results show that CXCR4 inhibition impacts BVI potentially delaying parenchymal metastasis.

Next, we used the 7‐week model where mice with shC orthotopically engrafted tumor cells were treated with AMD for 5 weeks according to established protocols (Azab *et al*, 2009, Domanska *et al,* 2012; Fig 6H). We had observed a clear effect of AMD on tumor growth inhibition that may have an effect on lung metastasis; therefore, we examined the abundance of tumor cells in the lungs. As we could not detect tumor cells by tissue staining at this early time point, we used qRT–PCR for *Neu* which revealed expression in PBS controls that was lower upon CXCR4 inhibition, indicative of less tumor cells in the lung (Fig 6I). We considered that tumor cells invading the lung may elicit an immune response as seen (Fig 2I); therefore, we used qRT–PCR to determine expression of *Cd8*, *Ifnγ*, and *Gzmb* that was indeed seen in PBS conditions, however with lower levels upon CXCR4 inhibition potentially reflecting less tumor cells in the lungs (Fig 6J–L).

Altogether, these results suggest that reduced metastasis is likely due to a major impact of CXCR4 inhibition on tumor growth.

### Combined abundance of CD8 TIL and high TNC and CXCL12 expression correlate with worsened breast cancer patient survival

**Figure 7:**
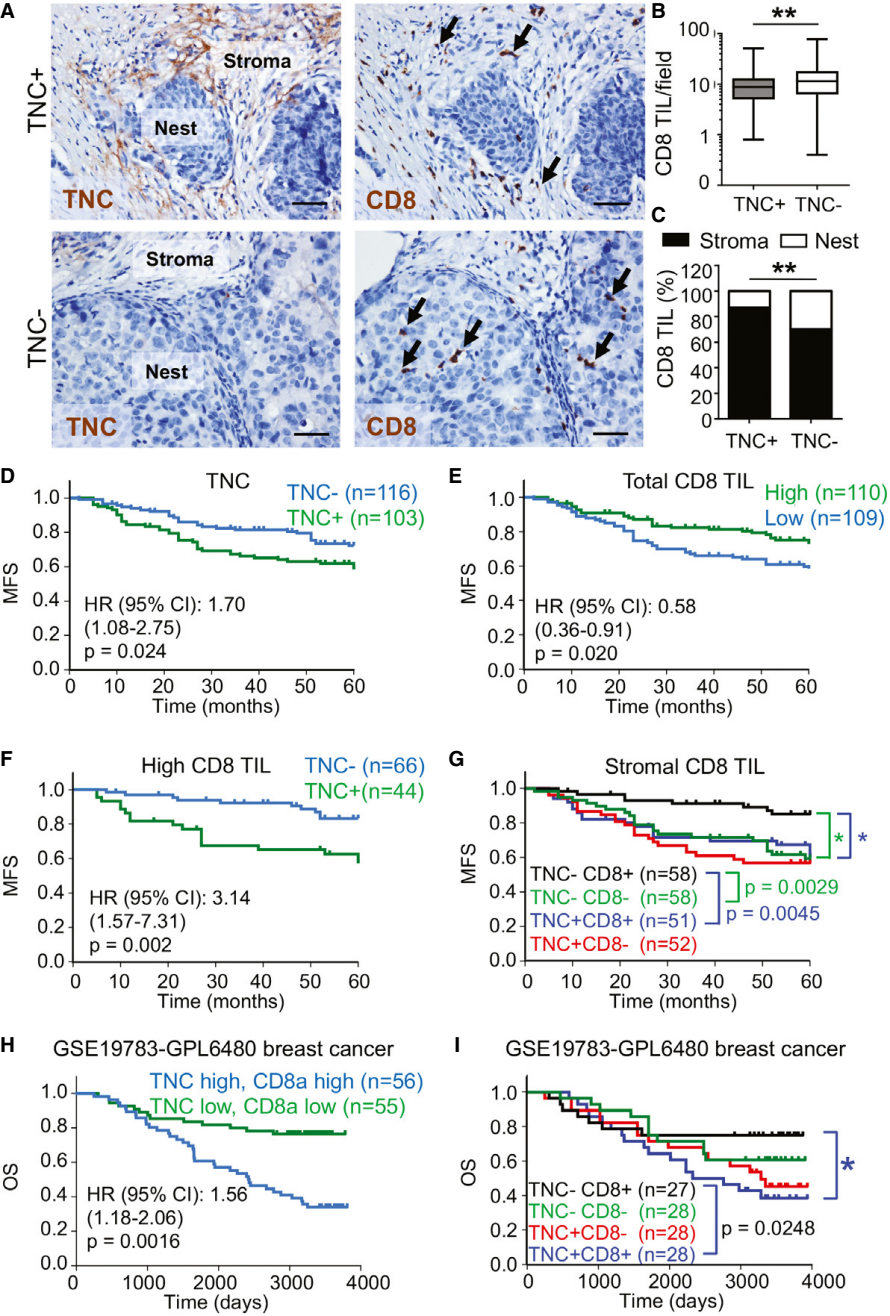
TNC expression, abundance, and localization of CD8 TIL in breast tumors and survival of human breast cancer patients A–CAdjacent human breast cancer tissue microarray (TMA) sections were stained with the indicated antibodies, scale bar, 50 μm, arrows point at CD8 TIL (A), and CD8 TIL were counted per field (B), or in the stroma and nest, respectively (C), *N* = 103 TNC^+^ tumors, *N* = 116 TNC‐ tumors. (B) **P = 0.0055, Fisher’s exact test. The central band represents the median, the ends of the box the first and the third quartiles, and the whiskers reach from each quartile to the minimum or maximum. (C) **P = 0.0055, Mann–Whitney test.D–GKaplan–Meier analysis to address correlation of TNC expression (D), total numbers of CD8 TIL (E), combined high (above median) CD8 TIL and high or low (above/below median) TNC (F) and stromal CD8 cells (G) with metastasis‐free survival (MFS) of breast cancer patients. Stromal CD8^−^ or CD8^+^ is defined as below or above median stromal CD8 TIL per area. Log‐rank test.H, ICorrelation of *TNC* and *CD8a* levels with overall survival (OS) using survival analysis on breast cancer patient cohort GSE 19783‐GPL6580. Hazard ratio (HR) and p values are indicated in Appendix Fig S5N. Log‐rank test. Source data are available online for this figure.

To address whether the results from the murine tumor models are relevant in human breast cancer, we stained a human breast cancer tissue microarray (Chen *et al,* 2010, 2011) for TNC and CD8. We used a limited dilution approach for TNC that allowed to discriminate between tumors expressing high (TNC^+^) and low TNC (TNC^−^) levels, respectively (Fig 7A, Appendix Fig S5A and B). TNC^−^ and TNC^+^ groups almost equally distributed between breast cancer subtypes except for Her2^+^ breast cancer specimens where the TNC^+^ phenotype was more prominent (41%) than the TNC^−^ phenotype (29%) (Table 1). Moreover, CD8 TIL were more abundant in TNC^−^ tumors which was pronounced in basal‐like cases (Fig 7B, Appendix Fig S5C). A closer investigation of CD8 TIL distribution showed that in both tumors (TNC^−^ and TNC^+^) CD8 TIL were more abundant in the stroma than in the nests, which applied to all three (Her2^+^, basal, and luminal) subtypes (Appendix Fig S5D–G, Table 2). However, the ratio of nest‐to‐stroma CD8 TIL was lower in TNC^+^ than in TNC^−^ tumors (Fig 7C). Also in TNC^+^ tumors, nest infiltration of CD8 TIL was lower than in TNC^−^ tumors supporting that TNC may play a role in retaining CD8 TIL in the stroma (Appendix Fig S5H and I).

**Table 1:** Comparison of TNC expression with clinicopathological characteristics in breast cancer patients.

Parameter	TNC^−^ patients (*n* = 116) (%)	TNC^+^ patients (*n* = 103) (%)	*P* value
MFS (months), mean (95% CI)	51.5 (48.5–54.5)	45.1 (41.0–49.1)	0.024^∗^
Age (year), median (range)	47 (31–75)	48 (29–76)	
< 50	56 (60.2)	80 (63.5)	0.570
≥ 50	37 (39.8)	46 (36.5)	
Molecular type: *n* (%)			
Luminal	55 (47.4)	40 (38.8)	0.202
Her‐2^+^	34 (29.3)	42 (40.8)	
Basal‐like	27 (23.3)	21 (20.4)	
TNM stage: *n* (%)			
TNM 1	7 (6.1)	5 (4.9)	0.738
TNM 2	76 (65.5)	64 (62.1)	
TNM 3	33 (28.4)	34 (33.0)	
Histological grade: *n* (%)			
Well‐differentiated	16 (13.8)	18 (17.5)	0.571
Moderately differentiated	71 (61.2)	56 (54.4)	
Poorly/undifferentiated	29 (25.0)	29 (28.2)	
Pathological type: *n* (%)			
Invasive ductal cancer	78 (67.2)	83 (80.6)	0.052
Invasive lobular cancer	16 (13.8)	7 (6.8)	
Mixture	16 (13.8)	4 (3.9)	
Others	6 (5.2)	9 (8.8)	
Neoadjuvant chemotherapy: *n* (%)			
Yes	68 (58.6)	54 (52.4)	0.357
No	48 (41.4)	49 (47.6)	

Comparisons of variables in the TMA cohort (Chen *et al,* 2010, 2011) were performed by using the chi‐square test.

^∗^ MFS (metastasis‐free survival) was analyzed by log‐rank test.

**Table 2:** Comparison of TNC expression with CD8 and CXCL12 expression in breast cancer patients.

Parameter	TNC^−^ patients (*n* = 116) (%)	TNC^+^ patients (*n* = 103) (%)	*P* value
CD8^+^ Leukocytes: *n* (%)			
< 9.4	50 (43.1)	59 (57.3)	0.036
≥ 9.4	66 (56.9)	44 (42.7)	
Nest CD8^+^ Leukocytes: *n* (%)			
< 1.7	36 (31.0)	74 (71.8)	< 0.001
≥ 1.7	80 (69.0)	29 (28.2)	
Stromal CD8^+^ Leukocytes: *n* (%)			
< 7.6	58 (50.0)	52 (50.5)	0.943
≥ 7.6	58 (50.0)	51 (49.5)	
CXCL12: *n* (%)			
Low	63 (54.3)	46 (44.7)	0.154
High	53 (45.7)	57 (55.3)	

Numbers of CD8 T leukocytes and expression of CXCL12 in the TMA cohort (Chen *et al,* 2010, 2011) was determined on two separate sections per tumor using the median value. Comparison of variables by using the chi‐square test.

Next, we asked whether TNC expression and CD8 TIL abundance potentially had an impact on patient survival. By Kaplan–Meier analysis of the TMA cohort, we first observed that high TNC levels (above the median) correlated with shorter metastasis‐free survival (MFS) as previously seen in other patient cohorts (Fig 7D; Minn *et al,* 2005; Oskarsson *et al,* 2011). Importantly, higher CD8 TIL levels were seen in patients with longer MFS (Fig 7E). However, the survival benefit of high CD8 TIL seemed to be abolished when TNC levels were also high (Fig 7F). In CD8^−^ tumors, TNC levels did not correlate with survival (Appendix Fig S5J). High or low abundance of CD8 TIL in the tumor nest also showed no difference in MFS (Appendix Fig S5K). In contrast, stromal CD8 TIL abundance and TNC levels, CD8^+^/TNC^−^, correlated with longest MFS whereas CD8^+^/TNC^+^ and CD8‐ conditions correlated with shorter MFS (Fig 7G, Appendix Fig S5J and K).

In the well‐studied breast cancer patient cohort GSE 19783‐GPL6580 representing all subtypes (Enerly *et al,* 2011), high *TNC* levels (above the median) and combined high TNC and high CD8 TIL correlated with shorter overall survival (Fig 7H and I, Appendix Fig S5L). Moreover, a combined high expression of *TNC* together with either high *CD8α* (HR 1,56) or *CD8β* (HR 1,57), *GZMB* (HR 1,52), *IFNγ* (HR 1,76), and *Perforin* (HR 1,75), respectively, correlated with a shorter overall survival and stronger power than *TNC* alone (HR 1,37) suggesting that stromal CD8 TIL tethering by TNC may be more important than CD8 TIL activation which does not seem to be linked to TNC (Appendix Fig S5N–U).

**Figure 8:**
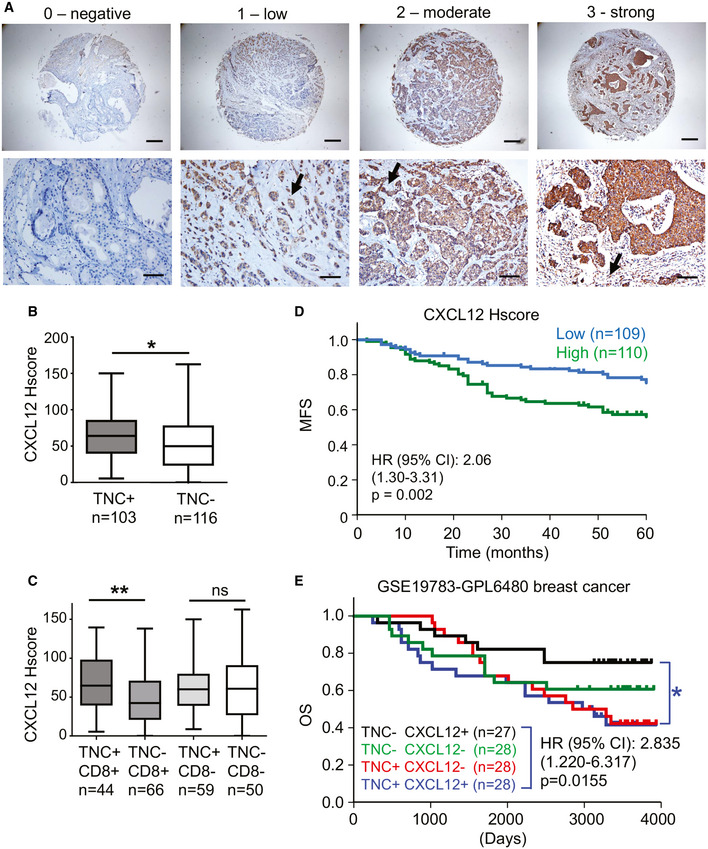
CXCL12 expression in human breast cancer and patient survival ARepresentative IHC images of CXCL12 intensity and staining pattern in the TMA. *N* = 219 tumors. Upper panel, scale bar, 400 μm; lower panel, scale bar, 100 μm. Arrows point at punctate staining in the stroma.B, CHistological score of CXCL12 staining in TNC^+^ and TNC^−^ tumors in combination with CD8^+^ (above median) and CD8^−^ (below the median). *P = 0.0206, **P = 0.0040, unpaired *t*‐test. The central band represents the median, the ends of the box the first and the third quartiles, and the whiskers reach from each quartile to the minimum or maximum.DKaplan–Meier survival analysis of breast cancer patients upon stratification into tumors with a CXCL12 Hscore below or above the median, *P* = 0.002. Mann–Whitney test.ESurvival analysis of breast cancer patients in the GSE 19783‐GPL6580 cohort upon stratification into tumors with high or low expression of TNC and CXCL12. Log‐rank test. HR and *P* values are indicated, see also Appendix Fig S6B. Source data are available online for this figure.

Next we stained the breast cancer TMA for CXCL12 and observed similar staining patterns as reported (Toullec *et al,* 2010; Lefort *et al,* 2017). We discriminated 4 staining intensities with no or low CXCL12 and high CXCL12 in tumor nests only, or combined high expression in tumor nest and stroma (Fig 8A). As in the grafting model, CXCL12 showed a punctate expression in the stroma (Fig 8A). As previously done, we combined intensity and abundance of CXCL12 expression in the so‐called Hscore (Lefort *et al,* 2017) that we then used to assess whether CXCL12 expression is correlated with TNC levels. Indeed, TNC^+^ tumors had a higher CXCL12 Hscore than TNC^−^ tumors (Fig 8B). The CXCL12 Hscore was similar in the different tumor subtypes (Appendix Fig S6A, Table 2). More detailed analysis revealed that the CXCL12 Hscore was higher when TNC and CD8 TIL were abundant (Fig 8C). Moreover, high TNC correlated with shorter overall survival when CXCL12 levels were high or the CXCL12 Hscore was above the median, whereas the TNC level was not prognostic in patients with tumors that had CXCL12 levels below the median (Fig 8D and E, Appendix Fig S6B).

**Figure 9:**
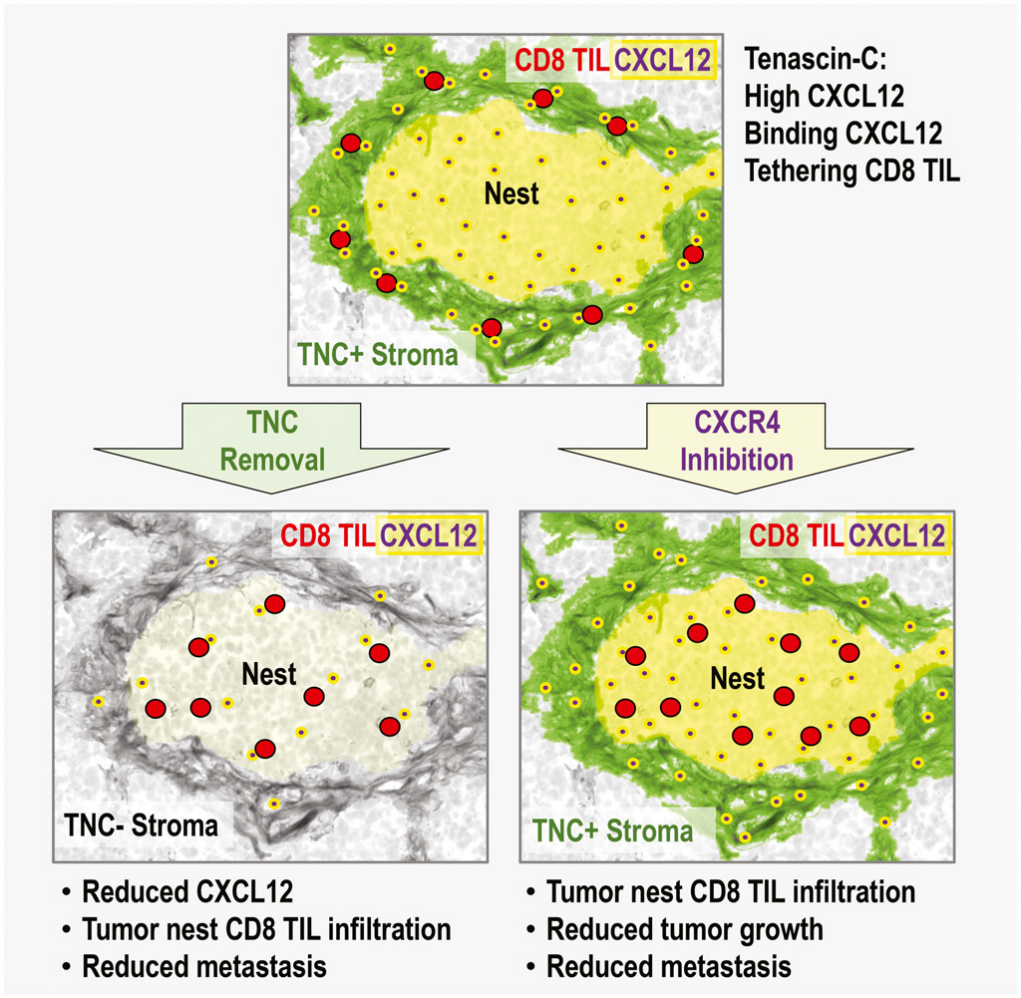
“TIL‐matrix‐release‐and‐reactivate” strategy through targeting TNC and CXCR4 TNC contributes to an immune suppressive TME in breast cancer by retaining CD8 TIL in the stroma. TNC induces CXCL12 in a TLR4‐dependent manner. The TNC protein binds CXCL12 generating a substratum that tethers CD8 TIL in the stroma thus preventing infiltration of CD8 TIL into the tumor nests and subsequent tumor cell killing. This mechanism could be relevant in human breast cancer as patients with tumors of high TNC expression show high CD8 TIL in the (TNC‐rich) stroma which correlates with shorter MFS. (Lower left) Upon removal of TNC anti‐tumor immunity is activated and characterized by lower CXCL12 expression, higher CD8 TIL nest infiltration, and a reduced metastasis rate as previously shown (Sun *et al,* 2019). In human breast cancer, low TNC and high CD8 TIL correlate with longest MFS. (Lower right) TNC impacts CD8 TIL through CXCL12/CXCR4. Inhibition of CXCR4 led to increased CD8 TIL infiltration, higher tumor cell apoptosis, and reduced tumor growth and subsequent lung metastasis. Also macrophages, loosing their M2 phenotype, infiltrate the tumor nests more numerously contributing to the observed activated CD8 TIL phenotype (e.g., less PD‐L1, CTLA4). Targeting TNC and CXCL12 could direct a novel “TIL‐matrix‐release‐and‐reactivate” strategy to improve ICT in breast cancer.

Altogether, our results suggest that TNC regulates macrophages and CD8 TIL involving their positioning inside the stroma. By upregulating CXCL12 through TLR4 and binding to CXCL12, we showed that TNC generates stromal niches that attract and immobilize CD8 TIL. Subsequently, CD8 TIL may be impaired in reaching and killing the tumor cells, contributing to the well‐known immune exclusion phenotype (Fig 9, Galon & Bruni, 2019). Here we showed that high CD8 TIL in the TNC stroma and combined high TNC and CXCL12 expression are parameters that define the immune exclusion phenotype which correlates with shorter survival of breast cancer patients that could have therapeutic value as seen in the preclinical models upon CXCR4 inhibition (Fig 9).

## Discussion

Tumors are complex ecosystems where the matrix is an instrumental component not only providing architectural but also signaling cues to cells (Lu *et al,* 2012; Pickup *et al,* 2014). In particular, the matrix molecule TNC that is highly abundant in (breast) cancers plays an instructive role in many steps during cancer progression including regulation of tumor immunity (Jachetti *et al,* 2015; Midwood *et al,* 2016; Deligne *et al,* 2020; Spenlé *et al,* 2020).

We revealed a novel mechanism of CD8 T cell impairment by TNC, retaining CD8 TIL inside the tumor stroma. We showed that TNC regulates CXCL12 expression through TLR4. We further showed that as in the murine model also in human breast cancer CXCL12 is predominantly expressed by the tumor cells. However, CXCL12 was also but less expressed by stromal cells presumably fibroblasts as seen in a murine pancreatic cancer model (Feig *et al,* 2013). Most importantly, we described that CXCL12 binds to TNC and turns the TNC matrix normally poorly adhesive for T cells (Hauzenberger *et al,* 1999; Huang *et al,* 2001), into a substratum that retains CD8 TIL in the TNC‐rich stroma. This mechanism seems to be relevant in human breast cancer as high TNC and high stromal CD8 TIL correlate with shorter MFS. High CXCL12 levels, potentially acting as protective shield toward CD8 TIL (by an unknown mechanism) (Vianello *et al,* 2006; Guyon, 2014), may even further potentiate CD8 TIL exclusion from the tumor nest. We have shown that inhibition of CXCR4 releases the matrix‐retained, now active CD8 TIL (as *Ifnγ* and *Gzmb* expression was elevated) to infiltrate the tumor tissue thus increasing tumor cell death and reducing tumor growth and subsequently metastasis. This mechanism could be clinically relevant as combined high TNC and high CXCL12 correlated with shortest MFS.

CXCR4 signaling is active in many cell types (Döring *et al,* 2014); thus, it is possible that CXCR4 inhibition also affects, for example, other immune subtypes or the vasculature. In support by RNA‐seq analysis, we observed a reduced angiogenesis‐associated gene signature and an aberrant EC phenotype in BVI upon AMD treatment. CXCR4 inhibition also increased the number and infiltration of activated macrophages whereas tumor‐promoting M2 macrophages disappeared. Moreover, CXCR4 inhibition reduced expression of immune suppressive IL10‐production/IL‐10 response‐associated genes and CD8 T cell inhibitory CTLA4, PD‐L1, and PD1, altogether presumably boosting CD8 TIL. However, our results propose that tumor cell‐directed cytotoxicity may only be accomplished upon release of the CD8 TIL from the matrix where TNC binding CXCL12 could be crucial and exploitable for therapy. It is conceivable that targeting matrix potentiates ICT. This is supported by published work showing that blocking the TLR4/TNC interaction increased the anti‐PD‐L1 effects on metastasis inhibition (Deligne *et al,* 2020) and that combined treatment of anti‐PD‐L1 with AMD increased TIL infiltration reducing tumor growth in a pancreatic tumor model (Feig *et al,* 2013).

Previously, the so‐called immunoscore, reflecting the abundance of CTL and memory T cells in the tumor cell nest or in the stroma, was identified as a better predictive tool for survival of colorectal carcinoma patients than the TNM classification (Galon *et al,* 2006; Galon *et al,* 2012). No information about a potential link to TNC or any other matrix was provided. It was also not known whether the immunoscore would also be a predictive tool in breast cancer. Here, we have shown that CD8 TIL immune exclusion in TNC‐rich stroma correlates with worsened prognosis and identified novel parameters for stratification of breast cancer patients, irrespective of subtype, likely to benefit from ICT. Our results predict that patients with tumors of high levels of CD8 TIL, TNC, and CXCL12 (detectable at mRNA and protein tissue level), correlating with short MFS, may benefit from ICT in combination with CXCR4 inhibition.

Indeed, clinical trials targeting CXCR4 in cancer patients with different drugs alone or in combination with ICT are ongoing (NCT02104427, NCT01359657). A combination of the CXCR4 antagonist X4P‐001 in combination with Pembrolizumab reactivating CD8 TIL (Emancipator, 2020) demonstrated a clinical response in patients with advanced melanoma (phase 1b trial, NCT02823405). Interestingly, the improved response was associated with an increased CD8 TIL density in the tumor center.

Altogether, our results propose that releasing CD8 TIL from the tumor matrix may be an important factor to empower ICT, where targeting matrix‐regulated CXCR4 signaling is worth to be further explored. RNA gene expression results from our novel immune competent breast cancer model provides several molecular indicators such as TNC and CXCL12 to direct a future combined “TIL‐matrix‐release‐and‐reactivate” strategy for improving ICT in breast cancer.

## Material and Methods

The experiments conformed to the principles set out in the WMA Declaration of Helsinki and the Department of Health and Human Services Belmont Report. More detailed information can be found in the Appendix Supplementary Information section.

### Immunohistochemical analysis of human breast tumors

Paraffin‐embedded tissue microarrays (TMA with 219 specimens with invasive breast cancer, Hubei Cancer Hospital, China, Chen *et al,* 2010; Chen *et al,* 2011) were used for hematoxylin/eosin (H&E) and antibody staining for TNC (Abcam, ab2074, 1/500), CXCL12 (Abcam, ab9797,1/200) and CD8 (DAKO, C8/144B) (Tables 1 and 2). Informed consent was obtained from each patient and the study was approved by the hospital's Ethics Committee. Slides were independently investigated blinded by two investigators. Duplicates were counted and the average signal count per slide was used for statistical analysis. The expression of TNC was scored according to the staining intensity and the proportion of positive stromal cells in the whole slide per tissue sample (Yang *et al,* 2017). CXCL12 expression is expressed as histological score (Hscore) (Lefort *et al,* 2017). CD8‐stained cells were counted in five representative pictures (20 fields) according to placement in tumor nest or stroma. Numbers were determined as high or low using the median value as cutoff.

### Patient survival analysis

The survival data for GSE19783‐GPL6480 (Enerly *et al,* 2011) were obtained from and analyzed by a web application (http://genomics.jefferson.edu/proggene/index.php) as described elsewhere (Goswami & Nakshatri, 2013). Hazard ratio above or below 1 within a confidence interval of 95% (HR, 95% CI), and *P* values were determined using the log‐rank test.

### Experimental mice and study design

MMTV‐NeuNT female mice in FVB/NCrl (WT or TNCKO background; more than 10 backcrosses into FVB (Sun *et al,* 2019)) were bred in parallel from heterozygous parents. Mice were housed and used according to the guidelines of INSERM and the ethical committee of Alsace, France (CREMEAS), and agreement number D 67‐482‐033 at the animal facilities of INSERM U682 and U1109 under pathogen‐free conditions in cages providing disposable homes and nesting paper, together with food and water at discretion. Before begin of the experiment, female mice (8–10 weeks of age) were acclimatized in the procedure room for at least two weeks and were daily checked for good health and welfare throughout the whole experiment (including posture and activity). Experimental groups consisted of at least 5 mice per time point and per genotype. Cages with experimental mice were placed in random order, which was then used throughout the experiment. Upon sedation with 4% isoflurane (Isoflurin® 1,000 mg/g, Axience), female WT or TNCKO FVB mice were grafted with 1 × 10^7^ NT193 cells (shC, *Tnc* knockdown (sh1TNC, sh2TNC; Sun *et al,* 2019) in the surgically opened left fourth mammary gland that was surgically closed afterward. For most experiments, sh2TNC cells were used and defined as “shTNC” if not further specified. Tumor growth was assessed by measuring the tumor size, and tumor volume was determined using the following calculation V = (width)^2^ × length/2. Tumor‐bearing mice (or mice with iv engrafted tumor cells) were daily treated with AMD3100 (Sigma, A5602) at 5 mg/kg or PBS as control by peritumoral (pt) injection (4‐ and 7‐week model), intraperitoneal injection (ip) (NeuNT model), or at 7.5 mg/kg ip injection (5‐week model). Mice were euthanized by cervical dislocation. Tumors and lungs were processed for freezing in liquid nitrogen (protein and mRNA analysis), or embedding in OCT (Sakura Finetek) or paraffin (Leica, 39601006) for immunostaining. The experimental setup is explained in the text and in Figs 6 and EV1, and EV2.

### Flow cytometry

Tumor tissue was digested in RPMI medium, 5% fetal bovine serum, penicillin (10,000 U/ml), streptomycin (10 mg/ml), Liberase TM (500 μg/ml), and DNAse (Thermo Fischer Scientific, 18047019, 100 μg/ml). Cells were separated through a 70‐µm cell strainer (BD Falcon, 352350) and counted. Flow cytometry with the indicated antibodies (Appendix Table S5) was performed with a LSR Fortessa machine (BD Biosciences) or a Beckman Coulter Gallios flow cytometer. FlowJo was used for the data analysis.

### Immunofluorescence (IF) and H&E staining

OCT‐embedded tissue sections were incubated with a blocking solution before incubation with the indicated primary antibody, washed, incubated with the secondary antibodies (Appendix Table S6), and DAPI (Sigma, catalog number D9542) followed by embedding with FluorSave^TM^ Reagent (Calbiochem, 345789). Images were analyzed with a Zeiss Axio Imager Z2 microscope with constant acquisition setting (microscope, magnification, light intensity, exposure time) and quantified by the ImageJ (National Institutes of Health) software using a constant threshold.

### Real‐time quantitative–PCR analysis

Total RNA was extracted from frozen tumors, lungs, and cultured cells with TRIzol (Invitrogen, 12044977). cDNAs (synthesized using random primers and Moloney murine leukemia virus reverse transcriptase (MultiScribe, Applied Biosystems, 10117254)) were used for qRT–PCR in an Mx3005P Real‐Time PCR System (Thermo Fisher Scientific) with Sybr Green Master mix (Thermo Fisher Scientific, 4344463) or Fast Taqman mix (Thermo Fisher Scientific, 4444557). Expression of mouse *Gapdh* mRNA (Life Technology, 433764T) was used as endogenous control in the comparative cycle threshold method (2‐ΔΔ*C*_t_) with the listed primers (Appendix Table S7).

### Cell culture

NT193 tumor cells (Sun *et al,* 2019) were cultured in DMEM‐glucose (Dutscher) complemented with 10% of fetal bovine serum (FBS, Dutscher). Silencing of TNC was done by short hairpin (sh)‐mediated gene expression knock down and was confirmed by qRT–PCR and Western blot (Sun *et al,* 2019). Tumor cells were pretreated with inhibitors for TGFβRI (GW788388, 10 μM, 45 min, Selleckchem, S2750), TLR4 (Cli95, 1 μg/ml, 6 h, InvivoGen, tlrl‐cli95), receptor tyrosine kinases (SU6668, 30 μM, 60 min, Tocris bioscience, 3335), and α4β1/α9β1 (BOP, 1 μM, 45 min, Tocris bioscience, 6047) according to the manufacturer’s instructions and as published (Sun *et al,* 2019; Deligne *et al,* 2020; Spenlé *et al,* 2020). Inhibitor‐treated tumor cells were then incubated with TNC (10 µg/ml) (Huang *et al,* 2001) for 24 h before RNA extraction. Purification of recombinant his‐tagged human TNC was done as described (Huang *et al,* 2001). The absence of endotoxins was determined with the PyroGene Recombinant Factor C Endpoint Fluorescent Assay (Lonza 50‐658 U) according to the manufacturer`s instruction.

### CD8 T leukocyte isolation and attraction and retention assay

CD8 T leukocytes were sorted by using the murine CD8a^+^ T Cell Isolation Kit (Miltenyi Biotec, 130‐104‐075) from spleens of FVB mice as described (Deligne *et al,* 2020). The lower surface of polycarbonate membrane transwells (Costar, 5 µm‐pore size, 3421) was coated with matrix molecules, washed with PBS, and blocked with 1% BSA overnight at 4°C. The lower chambers of the transwells were filled with TexMACS medium (Miltenyi Biotec, 130‐097‐196) containing CXCL12 (1 µg/ml, R&D Systems, 460‐SD‐050) or conditioned medium (CM) from shC or shTNC cells. AMD3100 (Sigma, A5602) at 5 µg/ml was added to the CD8 T cells for 1 h at 37°C before seeding cells in the upper chamber for 5 h at 37°C followed by collection of the medium in the lower chamber for cell counting by flow cytometry (Appendix Table S5). Cells on the surface of the lower side of the insert were fixed with 4% PFA (Santa Cruz, sc‐281692), stained with DAPI and imaged. Pictures were analyzed with the ImageJ software.

### Surface plasmon resonance (SPR) analysis

In SPR binding experiments (Biacore 2000 instrument (Biacore Inc.), recombinant human TNC was immobilized (Spenlé *et al,* 2020). CXCL12 (R&D Systems, 460‐SD‐050) (from 0.6 × 10^−7^ M to 6 × 10^−7^ M) was added to the chip at pH 7.4 (10 mM HEPES, 150 mM sodium chloride, 0.005% (v/v) surfactant P20), at a flow rate of 10 μL/min. A steady‐state condition was used to determine the affinity of CXCL12 for TNC. The dissociation constant (Kd) was determined using the 1:1 Langmuir association model.

### Negative staining, transmission electron microscopy, and CXCL12 binding assay

The interaction of murine TNC (Spenlé *et al,* 2020) with CXCL12 (R&D Systems, 460‐SD‐050) was visualized by negative staining and transmission electron microscopy as described (Bober *et al,* 2010; Spenlé *et al,* 2020). For inhibition experiments, TNC samples were pre‐incubated with the indicated concentrations of heparin dp10 (AMS Biotechnology, AMS.HO10). Specimens were examined in a Philips/FEI CM 100 TWIN transmission electron microscope operated at 60 kV accelerating voltage. Images were recorded with a side‐mounted Olympus Veleta camera and the ITEM acquisitions software. Binding of CXCL12 to TNC was determined by counting the number of colloidal gold particles along the length of the TNC monomer. Numbers of molecules from 500 randomly picked distinct TNC molecules were determined. Competition was done with increasing concentration of CXCL12 in TBS (50 mM Tris, 150 mM Nacl, pH 7.9).

### Gene expression analysis

The sequencing library was prepared from mRNA derived from tumors, shC, and shTNC cells, respectively, with the Ion Total RNA‐Seq Kit v2 (Thermo Fisher Scientific, 4475936). Sequencing was performed on an Ion Proton sequencer with the Ion PI™ Hi‐Q™ Sequencing 200 Kit (Thermo Fisher Scientific A26433). The transcriptome data were processed by the RNASeqAnalysis plugin from the Torrent Suite Software 5.06 (Thermo Fisher Scientific). RNA from MMTV‐NeuNT‐WT and TNCKO mammary tumors were used for the Affymetrix Microarray experiments, performed at the IGBMC Sequencing Core Facility (Illkirch, France).

### Statistical analysis

Gaussian distribution was tested by the D’Agostino‐Pearson normality test. When data followed a Gaussian distribution, statistical differences were analyzed by unpaired *t*‐test (with Welch’s correction in case of unequal variance) or ANOVA one‐way with Tukey post‐test. Otherwise, the Mann–Whitney test or a non‐parametric ANOVA followed by Dunn’s post‐test were used to verify significance of the observed differences. All statistical analyses were performed using the GraphPad Prism software. All results were compared to the respective control by the indicated statistical test, and only *P* < 0.05 are mentioned in the figure legends. Mean ± SEM. *P* < 0.05 were considered as statistically significant, *P < 0.05; **P < 0.01; ***P < 0.001.

## Author contributions

DM, ZS, KM, TL, and GO conceptualized the study. DM, ZS, AY, GR, CAF, CD, IVQ, WE, MN, MM, GC, NP, RV, JY, and TL performed experiments, analyzed, and interpreted the data. RC and HD supervised the RNA profiling and flow cytometry experiments. Experiments were performed as follows: SRP by GC, negative EM imaging by MM, RNA‐seq analysis by NP, IVQ, TL, and RC, human tissue microarray staining and patient survival analysis by ZS and JY, analysis and treatment of mice with AMD by DM, AY, GR, CAF, flow cytometry by DM, CD, RV, staining by DM, ZS, WE, AY, MN, CAF, preparation of human TNC by DM, WE, AY, CAF, TL, cell culture experiments by DM, AY, TL. DM, TL. GO wrote the manuscript, DM, ZS, AY, CAF, KM edited the manuscript. GO supervised the study. All authors read and discussed the manuscript.

## Conflict of interest

The authors declare that they have no conflict of interest.

## Supporting information



Appendix

Expanded View Figures PDF

Review Process File

Source Data for Figure 1

Source Data for Figure 2

Source Data for Figure 3

Source Data for Figure 4

Source Data for Figure 5

Source Data for Figure 6

Source Data for Figure 7

Source Data for Figure 8

## Data Availability

Microarray data generated for this study have been submitted to ArrayExpress under the accession numbers https://www.ebi.ac.uk/arrayexpress/experiments/E‐MTAB‐10149 and https://www.ebi.ac.uk/arrayexpress/experiments/E‐MTAB‐10135.
