# Supplementary material for: Tenascin‐C immobilizes infiltrating T lymphocytes through CXCL12 promoting breast cancer progression
Source: EMBO Mol Med. 2021 May 14;13(6):e13270. doi: 10.15252/emmm.202013270 (PMC8185552; doi:10.15252/emmm.202013270)
Supplement: Supplementary file 1 — Appendix [file EMMM-13-e13270-s007.docx]

**Appendix Supplementary Information for Murdamoothoo, Sun, Yilmaz et al., “Tenascin-C immobilizes infiltrating T lymphocytes through CXCL12 promoting breast cancer progression”**

**Appendix Supplementary Material, Methods and References**

**Appendix Figures S1-6**

**Appendix Table S1 Differential gene expression in MMTV-NeuNT tumors (WT versus TNCKO)**

**Appendix Table S2 Differential gene expression in TNC high (WT host/shC) and TNC low (TNCKO host/shTNC) tumors (11 weeks model)**

**Appendix Table S3 Differential gene expression in TNC high (WT host/shC) tumors upon AMD and PBS treatment (4 weeks model)**

**Appendix Table S4 Differential gene expression in TNC high (WT host/shC) and TNC low tumors (WT host/shTNC) upon PBS treatment (4 weeks model)**

**Appendix Table S5 List of antibodies for flow cytometry**

**Appendix Table S6 List of antibodies for tissue staining**

**Appendix Table S7 List of primers**

**Appendix Supplemental Material, Methods and References**

**Immunohistochemical analysis of human breast tumors**

Paraffin-embedded tissue microarrays (TMA with 219 specimens with invasive breast cancer (Hubei Cancer Hospital, China, Chen et al, 2010; Chen et al, 2011) were used for hematoxylin/eosin (H&E) and antibody staining for TNC (Abcam, ab2074, 1/500), CXCL12 (Abcam, ab9797,1/200) and CD8 (DAKO, C8/144B) **(Table 1 and 2)** for 50 minutes at room temperature. Tissue was deparaffinized, subjected to heat-induced epitope retrieval and then immersed in 3% H_2_O_2_ in methanol to quench endogenous peroxidase activity before incubation with the primary and followed by the secondary antibody (anti- mouse/rabbit, Envision plus, Dako) for 30 minutes at room temperature followed by exposure to the 3,3′-diaminobenzidine (DAB) reagent. The slides were stained with hematoxylin, dehydrated, and protected with a cover slip. Patient characteristics are summarized in **Table 1**. Informed consent was obtained from each patient and the study was approved by the Ethics Committee. Slides were independently investigated blinded by two investigators. Duplicates were counted and the average signal count per slide was used for statistical analysis. The expression of TNC was scored according to the staining intensity and the proportion of positive stromal cells in the whole slide per tissue sample (Yang et al, 2017). The immunohistochemical scores were determined as: 1- weak staining <50% or moderate staining < 20% of stromal cells; 2- weak staining in ≥ 50%, moderate staining in 20–50% or strong staining < 20%; 3- moderate staining in ≥ 50% or strong staining in ≥ 20%. Cases with score 2 and 3 were regarded as TNC high expression (Yang et al, 2017). For quantification of CXCL12 expression a histological score (Hscore) was given as a function of the percentage of positive cell area multiplied by the staining intensity (ranging from 0 to 3) in the whole section per tissue sample (Lefort et al, 2017). For CD8 quantification, positively stained cells that had morphological features compatible with lymphocytes were counted in five representative pictures (20 fields) according to two places within the tumor: (1) intraepithelial infiltrates (nest CD8 TIL), defined as CD8 TIL within cancer cell nests or in direct contact with cancer cells; and (2) stromal infiltrates (stromal CD8 TIL), defined as CD8 TIL within stromal areas that lack direct contact with epithelial cells. The total number of CD8 TIL was calculated as the sum of both and, average cell count per core was used for statistical analysis (Webb et al, 2014). Numbers of CD8 TIL and the Hscore of CXCL12 were determined as high or low using the median value as cut-off (**Table 2**).

**Patient survival analysis**

The survival data for GSE19783-GPL6480 (Enerly E et al., 2011) were obtained from and analyzed by a web application (<http://genomics.jefferson.edu/proggene/index.php)> as described elsewhere (Goswami CP et al, 2013). The cohort was split by median of corresponding gene expression (“High” and “Low,” respectively). Analysis was performed for overall survival and metastasis-free survival in the cohorts of breast cancer patients (Chen et al, 2010; Chen et al, 2011, and cohort GSE19783-GPL6480). Hazard ratio above or below 1 within a confidence interval of 95% (HR, 95% CI) and, p values were determined using the log-rank test.

**Experimental mice and study design**

MMTV-NeuNT female mice in FVB/NCrl (WT or TNCKO background; more than 10 backcrosses into FVB (Sun et al., 2019)) were bred in parallel from heterozygous parents. Mice were housed and used according to the guidelines of INSERM and the ethical committee of Alsace, France (CREMEAS) and agreement number D 67-482-033 at the animal facilities of INSERM U682 and U1109 under pathogen free conditions in cages providing disposable homes and nesting paper, together with food and water at discretion. Before begin of the experiment female mice (8-10 weeks of age) were acclimatized in the procedure room for at least two weeks and were daily checked for good health and welfare (including posture and activity). Experimental groups consisted of at least 5 mice per time point and per genotype. Cages with experimental mice were placed in random order, which was then used throughout the experiment. Upon sedation with 4% isoflurane (Isoflurin® 1000mg/g, Axience) female WT or TNCKO FVB mice were grafted with 1 x10^7^ NT193 cells (shC, *Tnc* knockdown (sh1TNC, sh2TNC) (Sun et al., 2019) in the surgically opened left fourth mammary gland that was closed afterwards. For most experiments sh2TNC cells were used and defined as “shTNC” if not further specified. Tumor growth was assessed by measuring the tumor size and tumor volume was determined using the following calculation V= (width)^2^ x length/2. Tumor-bearing mice (or mice with iv engrafted tumor cells) were daily treated with AMD3100 (Sigma, A5602) at 5 mg/kg or PBS as control by peritumoral (pt) injection (4 and 7 weeks model), intraperitoneal injection (ip) (NeuNT model), or at 7,5 mg/kg ip injection (5 weeks model). Mice were euthanized by cervical dislocation. Tumors and lungs were processed for freezing in liquid nitrogen (protein and mRNA analysis), or embedding in OCT (Sakura Finetek) or paraffin (Leica, 39601006) for immunostaining. The experimental setup is explained in the text and in **Fig. 6, EV1** and **EV2**

**Metastasis analysis**

Lungs from the different protocols were collected and embedded in OCT for IF tissue analysis (NeuNT, 7 weeks model) or in paraffin for stereological analysis. Pieces (2 mm) of paraffin embedded lung tissue was embedded again into paraffin and cut into 7 μm thick slices as previously described (Saupe et al., 2013). Two sections 21 μm apart were used representing 2 to 8 distinct areas of the lung. Parenchymal metastasis (PM) and Blood Vessel Invasions (BVI) were imaged and quantified (number and size) as described (Sun et al., 2019). In the 7 weeks tumor group sections from the biggest lung lobe of the 3 biggest tumor bearing mice were prepared. Every 50 µm a section was collected to give rise to 15 distinct areas (5 slides with 3 tissue sections) followed by CK8/18 and TNC staining. No tumor cells were detected, therefore qRTPCR for *Neu*, *Cd8a*, *Ifnγ* and *Gzmb* was performed (see below).

**Flow cytometry**

Tumor tissue was cut into small pieces (< 5mm^3^) and digested in PRMI medium supplemented with 5 % of inactivated fetal bovine serum, penicillin (10 000 U/ml), streptomycin (10 mg/ml), Liberase TM (500ug/ml) and DNAse (ThermoFischer Scientific, 18047019,100ug/ml) for 30 minutes at 37°C under agitation. Cells were then separated through a 70 µm cell strainer (BD Falcon, 352350) and counted. Cells were stained with Dead viability dye-efluor (Thermo Fisher, 65-0863-18) according the manufacturer’s instructions. Cells were incubated with 2% FcBlock CD16/32 solution (Thermo Fisher, 14-0161-85) and then stained with a standard panel of immunophenotyping antibodies **(Appendix Table S5**). Data were acquired with a LSR Fortessa machine (BD Biosciences, San Jose, CA, USA) or a Beckman Coulter Gallios flow cytometer. Adjustments and data analysis were performed by using FlowJo software.

**Hematoxylin-Eosin staining (HE)**

The OCT embedded tissue sections (8μm thick) were incubated in ddH20 before staining with hematoxylin (Surgipath, catalog number 3801560) for 30 seconds and eosin (Sigma, catalog number HT110132) for 10 seconds, spaced by 1 minute of dd H20 washes. After the last wash, tissue sections were dehydrated 5 minutes in increasing percentage baths of ethanol (from 70 to 100%) and toluene and then covered with the Eukitt solution (Sigma, catalog number 03989).

**Immunofluorescence staining (IF)**

OCT embedded tissue sections (7 µm-thick) were incubated with a blocking solution (5% normal goat serum in PBS) for 1 hour at room temperature before incubation with the indicated primary antibody (**Appendix Table S6**) overnight at 4°C, washed, incubated with the secondary antibodies (**Appendix Table S6**), and DAPI (Sigma) for 10 min at room temperature followed by embedding with FluorSave^TM^ Reagent (Calbiochem, 345789). Images were analyzed with a Zeiss Axio Imager Z2 microscope with constant acquisition setting (microscope, magnification, light intensity, exposure time) and quantified by the ImageJ (National Institutes of Health) software using a constant threshold.

**Real Time quantitative PCR (qPCR) analysis**

Total RNA was extracted from frozen tumors, lungs and cultured cells with TRIzol (Invitrogen, 12044977). RNA quality was confirmed by optical density measurement (OD 260 nm). cDNAs (synthesized using random primers and Moloney murine leukemia virus reverse transcriptase (MultiScribe, Applied Biosystems, 10117254)) were used for qRTPCR in an Mx3005P Real-Time PCR System (ThermoFisher Scientific). Reactions were carried out in duplicate for all conditions using a Sybr Green Master mix (ThermoFisher Scientific, catalog number 4344463) or Fast Taqman mix (ThermoFisher Scientific, catalog number 4444557) and expression of mouse or human *Gapdh* mRNA (Life Technology, catalog number 433764T) was used as endogenous control in the comparative cycle threshold method (2-ΔΔCt) with the listed primers (**Appendix Table S7).**

**Analysis of CXCL12 protein expression by ELISA**

CXCL12 expression was determined in tissue and protein lysates by using the mouse CXCL12 Quantikine ELISA Kit (R&D systems, MCX120) according to the manufacturer’s instructions.

**Cell culture**

NT193 tumor cells (Sun et al., 2019) were cultured in DMEM-glucose (Dutscher) complemented with 10% of fetal bovine serum (FBS, Dutscher), 100 U/mL penicillin, 100 μg/mL streptomycin (PenStrep, Dutscher), 40 U/mL Gentamicin (ThermoFisher Scientific). Cells were maintained at 37°C in a humidified atmosphere of 5 % CO_2_. Silencing of TNC was done by short hairpin (sh) mediated gene expression knock down and was confirmed by qRTPCR and western blot (Sun et al., 2019). Tumor cells were pretreated with inhibitors for TGFβRI (GW788388, 10μM, 45 minutes, Selleckchem, S2750), TLR4 (Cli95, 1 μg/mL, 6 hours, InvivoGen, tlrl-cli95), receptor tyrosine kinases (SU6668, 30 μM, 60 minutes, Tocris bioscience, 3335), and α4β1/α9β1 (BOP, 1 μM, 45 minutes, Tocris bioscience, 6047) according to the manufacturer`s instructions and as published (Sun et al., 2019, Deligne et al.,2020, Spenle et al., 2020). Inhibitor treated tumor cells were then incubated with TNC (10 µg/ml) (Huang et al., 2001) for 24 hours before RNA extraction. Purification of recombinant his-tagged human TNC was done as described (Huang et al., 2001). The absence of LPS was determined with the endotoxin detection assay (PyroGene Recombinant Tactor C Endpoint Fluorscent Assay, Lonza 50-658U) according to the manufacturer`s instruction.

**CD8 T leukocyte isolation**

Spleens of FVB mice were isolated and cut into small pieces (70 µm filter with a syringe piston), washed with PBS, and incubated with potassium ammonium chloride lysing buffer (ThermoFisher Scientific, 10492) for 30 minutes at room temperature for erythrocyte lysis as described (Deligne et al., 2020). CD8 T leukocytes were sorted by using the murine CD8a^+^ T Cell Isolation Kit (Miltenyi Biotec, 130-104-075) from spleens of FVB mice as described (Deligne et al., 2020).

**CD8 T cell attraction, retention and functional assays**

The lower surface of polycarbonate membrane transwells (Costar, 5 µm-pore size, 3421) were coated with fibronectin (FN, TNC or collagen I (COL) (Huang et al., 2001)) at 1 µg/cm^2^ for 1 hour at 37°C, washed with PBS and blocked with 1% BSA overnight at 4°C. The lower chambers of the transwells were filled with TexMACS medium (Miltenyi Biotec, 130-097-196) containing CXCL12 (1 µg/mL, R&D Systems, 460-SD-050) or conditioned medium (CM) from shC or shTNC NT193 cells (collected 24 hours after incubation in serum free DMEM). AMD3100 (Sigma, A5602) at 5 µg/mL was added to the CD8 T cells for 1 hour at 37°C before seeding cells in the upper chamber for 5 hours at 37°C followed by collection of the medium in the lower chamber for cell counting by flow cytometry (**Appendix Table S5**). For assessment of CD8 T cell retention, cells on the surface of the lower side of the insert were fixed with 4% PFA (Santa Cruz, sc-281692) and stained with DAPI. Pictures were analyzed with the ImageJ software. CD8 T cells were treated *in vitro* with a CD3 mouse antibody (anti-mouse CD3e, clone 145-2C11, Armenian hamster IgG, BD Pharmingen, ref: 553057, 1µg/mL) for 48 hours followed by 45 minutes incubation with AMD (5 µg/mL) before RNA extraction and qRTPCR. Cell proliferation was assessed by using the CellTiter 96® AQueous Non-Radioactive Cell Proliferation Assay (Promega G5421) according to the manufacturer’s instructions. In brief, cell expansion was measured after 24 hours by incubating CellTiter 96® reagent for 2 hours at 37°C. The optical density (OD: 490 nm) was measured with a Varioskan LUX Multimode Microplate Reader (Thermo Fischer).

**Surface Plasmon Resonance analysis**

In SPR binding experiments (Biacore 2000 instrument (Biacore Inc.) recombinant human TNC was immobilized (Spenle et al., 2020). CXCL12 (R&D Systems, 460-SD-050) (from 0.6x10^-7^ M to 6x10^-7^ M) was added to the chip at pH 7.4 (10 mM HEPES, 150 mM sodium chloride, 0.005% (v/v) surfactant P20), at a flow rate of 10 μL/min. A blank CM5 chip was used for background correction. A steady state condition was used to determine the affinity of CXCL12 for TNC. The Dissociation constant (Kd) was determined using the 1:1 Langmuir association model as described by the manufacturer (https://www.biacore.com/lifesciences/help/kinetic_model_1_1_binding/index.html).

**Negative staining, transmission electron microscopy and CXCL12 binding assay**

The interaction of murine TNC (Spenle et al., 2020) with CXCL12 (R&D Systems, 460-SD-050) was visualized by negative staining and transmission electron microscopy as described (Bober et al, 2010; Spenle et al., 2020). Briefly, TNC samples (20 nM) were incubated with a 3-molar excess of CXCL12 (R&D Systems, 460-SD-050) for one hour at 37°C in tris buffered saline (TBS), pH 7.4. For visualization in the electron microscope CXCL12 was conjugated with 5 nm colloidal gold (Baschong et al., 1990). For inhibition experiments, TNC samples were pre-incubated with the indicated amounts of heparin dp10 (AMS Biotechnology, AMS.HO10) for one hour at 37°C. Specimens were examined in a Philips/FEI CM 100 TWIN transmission electron microscope operated at 60 kV accelerating voltage. Images were recorded with a side-mounted Olympus Veleta camera with a resolution of 2048 x 2048 pixels (2k x 2K) and the ITEM acquisitions software. Binding of CXCL12 particles to TNC was determined by counting the number of colloidal gold particles along the length of the TNC monomer. Number of molecules from 500 randomly picked distinct TNC molecules were determined. Competition was done with increasing concentration of CXCL12 in TBS (50 mM Tris, 150 mM Nacl, pH 7.9).

**Gene expression analysis**

Integrity of mRNA from NT193 shC and shTNC cells (2 samples per group) was assessed with the Agilent total RNA Pico Kit on a 2100 Bioanalyzer instrument (Agilent Technologies). The sequencing library was prepared with the Ion Total RNA-seq kit v2 (ThermoFisher Scientific, 4475936) according to the manufacturer's instructions. The libraries were loaded two by two at a concentration of 20 pM on an Ion PI™ Chip using the Ion Chef Instrument (ThermoFisher Scientific, 4484177). Finally, the sequencing was performed on an Ion Proton sequencer with the Ion PI™ Hi-Q™ Sequencing 200 Kit (ThermoFisher Scientific A26433). The transcriptome data were processed by the RNASeqAnalysis plugin from the Torrent Suite Software 5.06 (ThermoFisher Scientific). The reads were mapped by a two-step alignment scheme (Dobin et al, 2013), Langmead et al, 2012). RNA from NT193 tumors (TNC-high and TNC-low, PBS and AMD treated) (2 samples per condition) was isolated using the RNeasy Mini Kit (Qiagen, 74104) and RNA integrity was determined with an Agilent Bioanalyzer 2100 (Pico Kit, Agilent Technologies). Total RNA Sequencing libraries were prepared with SMARTer Stranded Total RNA-Seq Kit v2 - Pico Input Mammalian (TaKaRa, 634411) according to the manufacturer’s protocol. Libraries were pooled and sequenced (paired-end 2*75bp) on a NextSeq500 using the NextSeq 500/550 High Output Kit v2 according to the manufacturer’s instructions (Illumina, 20024907). Quality control of every sample was assessed with the NGS Core Tools FastQC and sequence reads were mapped using STAR and Bowtie2. The total mapped reads were finally available in BAM format for raw read counts extraction. Read counts were determined with the htseq-count tool of the Python package HTSeq (Anders et al, 2015). Differential analyses were performed by the DESEQ2 package of the Bioconductor framework (Love et al, 2014) (**Appendix Tables S1 – S4**). RNA from MMTV-NeuNT WT and TNCKO mammary tumors (3 samples per group) were used for the Microarray experiments, performed at the IGBMC Affymetrix Core Facility (Illkirch, France). Tumors were collected 3 months after tumor palpation which was around 8 months for mice in a WT background and (due to a delay in tumor appearance) at 9 – 13 months in the TNCKO background (Sun et al., 2019). Due to a high heterogeneity simple and adjusted p values < 0.05 were considered. Biotinylated single strand cDNA targets were prepared by using the Affymetrix GeneChip WT Terminal Labeling Kit (Affymetrix, 900670) according to manufacturer`s recommendations, and 2 μg of cDNAs were hybridized for 16 hours at 45°C on GeneChip Human Gene 1.0 ST arrays (Affymetrix, 901086). The chips were washed and stained in the GeneChip® Fluidics Station 450 (Affymetrix, 00-0079) and scanned with the GeneChip Scanner 3000 7G (Affymetrix, 00-0210). Raw data (.CEL Intensity files) were extracted from the scanned images using the Affymetrix GeneChip Command Console (AGCC) version 3.1. CEL files were further processed with Affymetrix Expression Console software version 1.1. to calculate probe set signal intensities using Robust Multi-array Average (RMA) algorithms with default settings. Normalized data were analyzed with Bioconductor's LIMMA-package ([http://www.bioconductor.org](http://www.bioconductor.org/)) (1)  using R (version 3.1.3) (2) (<http://www.r-project.org>) to identify differentially expressed genes (DEG) (**Appendix Table S1**).

For all gene expression data, up-regulated and down-regulated genes were selected based on the p-value (p < 0,05) or adjusted p-value cutoff of 10% and a minimum fold-change of +/- 0.8 **(Appendix Tables S1 – S4)**. Gene Ontology (GO) term enrichment (GO_Biological process_2018) was performed using Enrichr tool (Ma’ayan laboratory) (Ferris, 2015; Seiwert et al., 2016). Deregulated gene expression analysis was performed by using the PANTHER version 11 and REACTOME software. GSEA analysis was performed by using WEB-based GEne SeT AnaLysis Toolkit (WebGestalt) based on Geneontology functional database.

**Statistical analysis**

Gaussian distribution was tested by the D’Agostino-Pearson normality test. When data followed a Gaussian distribution, statistical differences were analyzed by unpaired t-test (with Welch’s correction in case of unequal variance) or ANOVA one-way with Tukey post-test. Otherwise, the Mann Whitney test or a non-parametric ANOVA followed by Dunn’s post-test were used to verify significance of the observed differences. All statistical analyses were performed using the GraphPad Prism software. All results were compared to the respective control by the indicated statistical test, and only p values < 0,05 are mentioned in the figure legends. Mean ± SEM. p values < 0.05 were considered as statistically significant, * p < 0.05; ** p < 0.01; *** p < 0.001.

**References**

Anders S, Pyl PT, Huber W (2015) HTSeq--a Python framework to work with high-throughput sequencing data. Bioinformatics 31: 166–169.

Arcangelis AD, Neuville P, Boukamel R, Lefebvre O, Kedinger M, Simon-Assmann P (1996) Inhibition of Laminin od-Chain Expression Leads to Alteration of Basement Membrane Assembly and Cell Differentiation. *The Journal of Cell Biology* 133: 14.

Arpel A, Sawma P, Spenlé C, Fritz J, Meyer L, Garnier N, Velázquez-Quesada I, Hussenet T, Aci-Sèche S, Baumlin N, et al. (2014) Transmembrane Domain Targeting Peptide Antagonizing ErbB2/Neu Inhibits Breast Tumor Growth and Metastasis. Cell Reports 8: 1714–1721.

Aufderheide E and Ekblom P. (1988) Tenascin During Gut Development: Appearance in the Mesenchyme, Shift in Molecular Forms, and Dependence on Epithelial-Mesenchymal Interactions. J Cell Biol. 9.

Baschong W, Wrigley NG (1990) Small colloidal gold conjugated to fab fragments or to immunoglobulin g as high-resolution labels for electron microscopy: A technical overview. J Elec Microsc Tech 14: 313–323.

Bober M, Enochsson C, Collin M, Mörgelin M (2010) Collagen VI is a subepithelial adhesive target for human respiratory tract pathogens. J Innate Immun 2: 160–166.

Chen C, Xia H-S, Gong Y-P, Peng J, Peng C-W, Hu M-B, Zhu X-B, Pang D-W, Sun S-R, Li Y (2010) The quantitative detection of total HER2 load by quantum dots and the identification of a new subtype of breast cancer with different 5-year prognosis. Biomaterials 31: 8818–8825.

Chen C, Sun S-R, Gong Y-P, Qi C-B, Peng C-W, Yang X-Q, Liu S-P, Peng J, Zhu S, Hu M-B, et al. (2011) Quantum dots-based molecular classification of breast cancer by quantitative spectro analysis of hormone receptors and HER2. Biomaterials 32: 7592–7599.

Deligne C, Murdamoothoo D, Gammage AN, Gschwandtner M, Erne W, Loustau T, Marzeda AM, Carapito R, Paul N, Velazquez-Quesada I, et al. (2020) Matrix-Targeting Immunotherapy Controls Tumor Growth and Spread by Switching Macrophage Phenotype. Cancer Immunol Res 8: 368–382.

Dobin A, Davis CA, Schlesinger F, Drenkow J, Zaleski C, Jha S, Batut P, Chaisson M, Gingeras TR (2013) STAR: ultrafast universal RNA-seq aligner. Bioinformatics 29: 15–21.

Enerly E, Steinfeld I, Kleivi K, Leivonen S-K, Aure MR, Russnes HG, Rønneberg JA, Johnsen H, Navon R, Rødland E, et al. (2011) miRNA-mRNA Integrated Analysis Reveals Roles for miRNAs in Primary Breast Tumors. PLoS ONE 6: e16915.

Ferris RL (2015). Immunology and Immunotherapy of Head and Neck Cancer. J Clin Oncol.33:3293–304

Goswami CP, Nakshatri H (2013) PROgene: gene expression based survival analysis web application for multiple cancer. J Clin Bioinforma 22: 3-22.

Györffy B, Lanczky A, Eklund AC, Denkert C, Budczies J, Li Q, Szallasi Z (2010) An online survival analysis tool to rapidly assess the effect of 22,277 genes on breast cancer prognosis using microarray data of 1,809 patients. Breast Cancer Res Treat 123: 725–731.

Huang W, Chiquet-Ehrismann R, Moyano JV, Garcia-Pardo A, Orend G (2001) Interference of tenascin-C with syndecan-4 binding to fibronectin blocks cell adhesion and stimulates tumor cell proliferation. Cancer Res 61: 8586–8594.

Langmead B, Salzberg SL (2012) Fast gapped-read alignment with Bowtie 2. Nat Methods 9: 357–359.

Lefort S, Thuleau A, Kieffer Y, Sirven P, Bieche I, Marangoni E, Vincent-Salomon A, Mechta-Grigoriou F (2017) CXCR4 inhibitors could benefit to HER2 but not to triple-negative breast cancer patients. Oncogene 36: 1211–1222.

Love MI, Huber W, Anders S (2014) Moderated estimation of fold change and dispersion for RNA-seq data with DESeq2. Genome Biol 15: 550.

Muller WJ, Sinn E, Pattengale PK, Wallace R, Leder P (1988) Single-step induction of mammary adenocarcinoma in transgenic mice bearing the activated c-neu oncogene. Cell 54: 105–115.

Seiwert TY, Burtness B, Mehra R, Weiss J, Berger R, Eder JP, et al. Safety and clinical activity of pembrolizumab for treatment of recurrent or metastatic squamous cell carcinoma of the head and neck (KEYNOTE-012): an open-label, multicentre, phase 1b trial (2016). Lancet Oncol. 17:956–65.

Simo P, Simon-Assmann P, Arnold C, Kedinger M. (1992) Mesenchyme-mediated effect of dexamethasone on laminin in cocultures of embryonic gut epithelial cells and mesenchyme-derived cells. JCS. 161-171.

Spenlé C, Loustau T, Murdamoothoo D, Erne W, Beghelli-de la Forest Divonne S, Veber R, et al. (2020) Tenascin-C Orchestrates an Immune-Suppressive Tumor Microenvironment in Oral Squamous Cell Carcinoma. Cancer Immunol Res.

Sun Z, Velázquez-Quesada I, Murdamoothoo D, Ahowesso C, Yilmaz A, Spenlé C, Averous G, Erne W, Oberndorfer F, Oszwald A, et al. (2019) Tenascin-C increases lung metastasis by impacting blood vessel invasions. Matrix Biology 83: 26–47.

Talts JF, Wirl G, Dictor M, Muller WJ, Fässler R (1999) Tenascin-C modulates tumor stroma and monocyte/macrophage recruitment but not tumor growth or metastasis in a mouse strain with spontaneous mammary cancer. J Cell Sci 112 ( Pt 12): 1855–1864.

Webb JR, Milne K, Watson P, Deleeuw RJ, Nelson BH (2014) Tumor-infiltrating lymphocytes

expressing the tissue resident memory marker CD103 are associated with increased survival in high-grade serous ovarian cancer. Clin Cancer Res 20: 434–444.

Yang Z, Ni W, Cui C, Fang L, Xuan Y (2017) Tenascin C is a prognostic determinant and potential cancer-associated fibroblasts marker for breast ductal carcinoma. Exp Mol Pathol 102: 262–267.

Yang F, Takagaki Y, Yoshitomi Y, Ikeda T, Li J, Kitada M, Kumagai A, Kawakita E, Shi S, Kanasaki K, Koya D (2019) Inhibition of Dipeptidyl Peptidase-4 Accelerates Epithelial–Mesenchymal Transition and Breast Cancer Metastasis via the CXCL12/CXCR4/mTOR Axis. Cancer Res 79:735-746.

**Appendix Figures S1-6**

**Appendix Fig. S1**

**
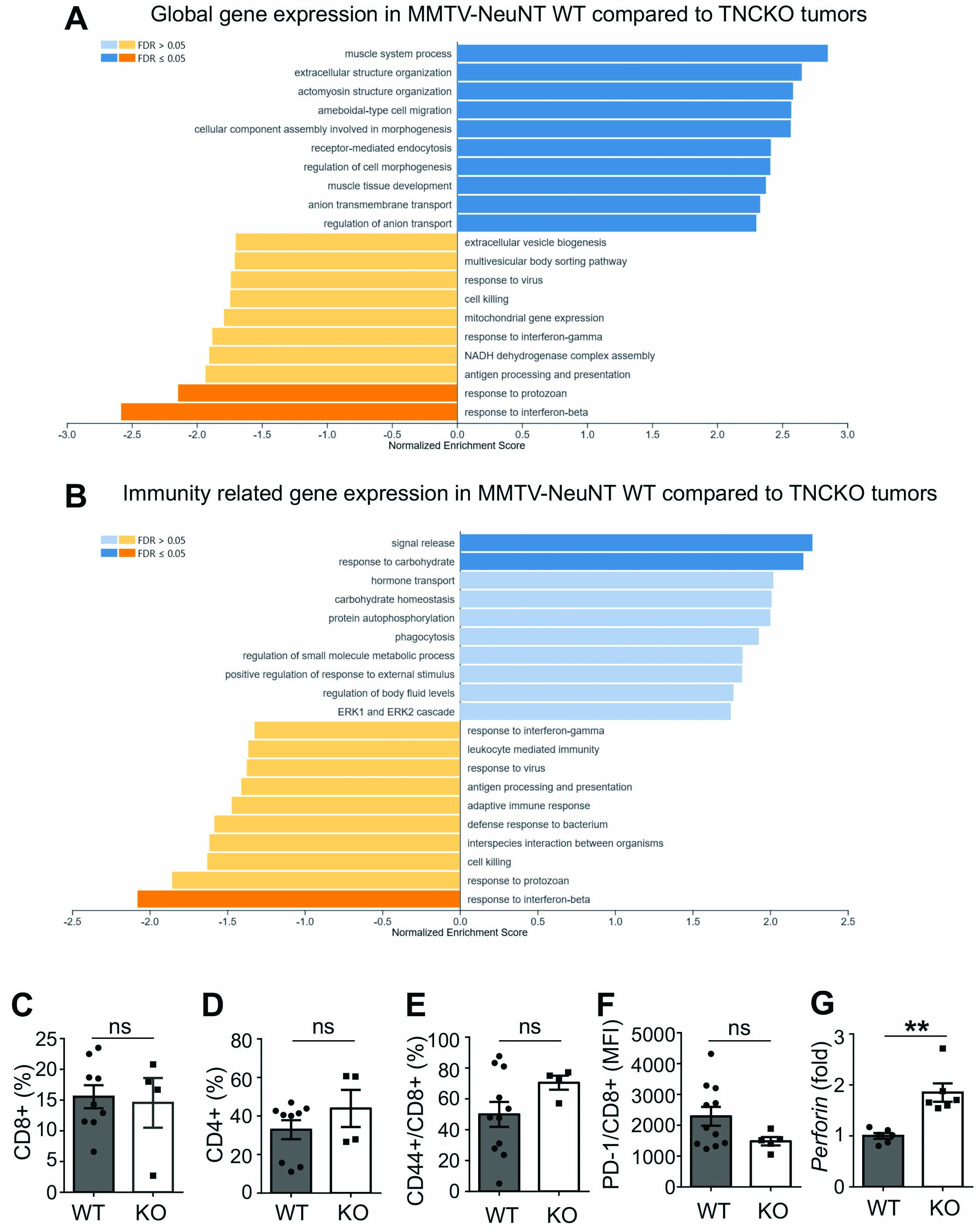
**

**Appendix Fig. S1 Gene ontology representation and immune cell infiltrate in MMTV-NeuNT tumors**

**(A)** Gene set enrichment analysis (GSEA) of the 2260 differentially expressed genes in MMTV-NeuNT tumors from TNC WT vs. KO mice. Up-regulated pathways are those enriched in the over-represented gene sets (Geneontology database) and are represented on the right. Down-regulated pathways are those enriched in the negatively regulated gene sets and represented on the left. The analysis showed that in the TNC WT tumors muscle extracellular and actomyosin structure organization were upregulated, while genes involved in interferon responses were down-regulated. False discovery rate (FDR) > 0,05 and ≤ 0,05. **(B)** Gene set enrichment analysis of differentially expressed immune-related genes in MMTV-NeuNT WT versus TNCKO tumors. False discovery rate (FDR) > 0,05 and ≤ 0,05. **(C - F)** Tumor infiltrating immune cells determined by flow cytometry for the indicated markers expressed as % of T cells. N = 9 WT, N = 4 TNCKO **(C, D)** and % of CD8 T cells, N = 11 WT, N = 4, **(E, F)**. p > 0.05, Mann-Whitney test **(C - F). (G)** Differential expression of *Perforin* as determined by qRTPCR. N = 6 WT, N = 6 TNCKO, p = 0.0011, unpaired t-test.

**Appendix Fig. S2**

**
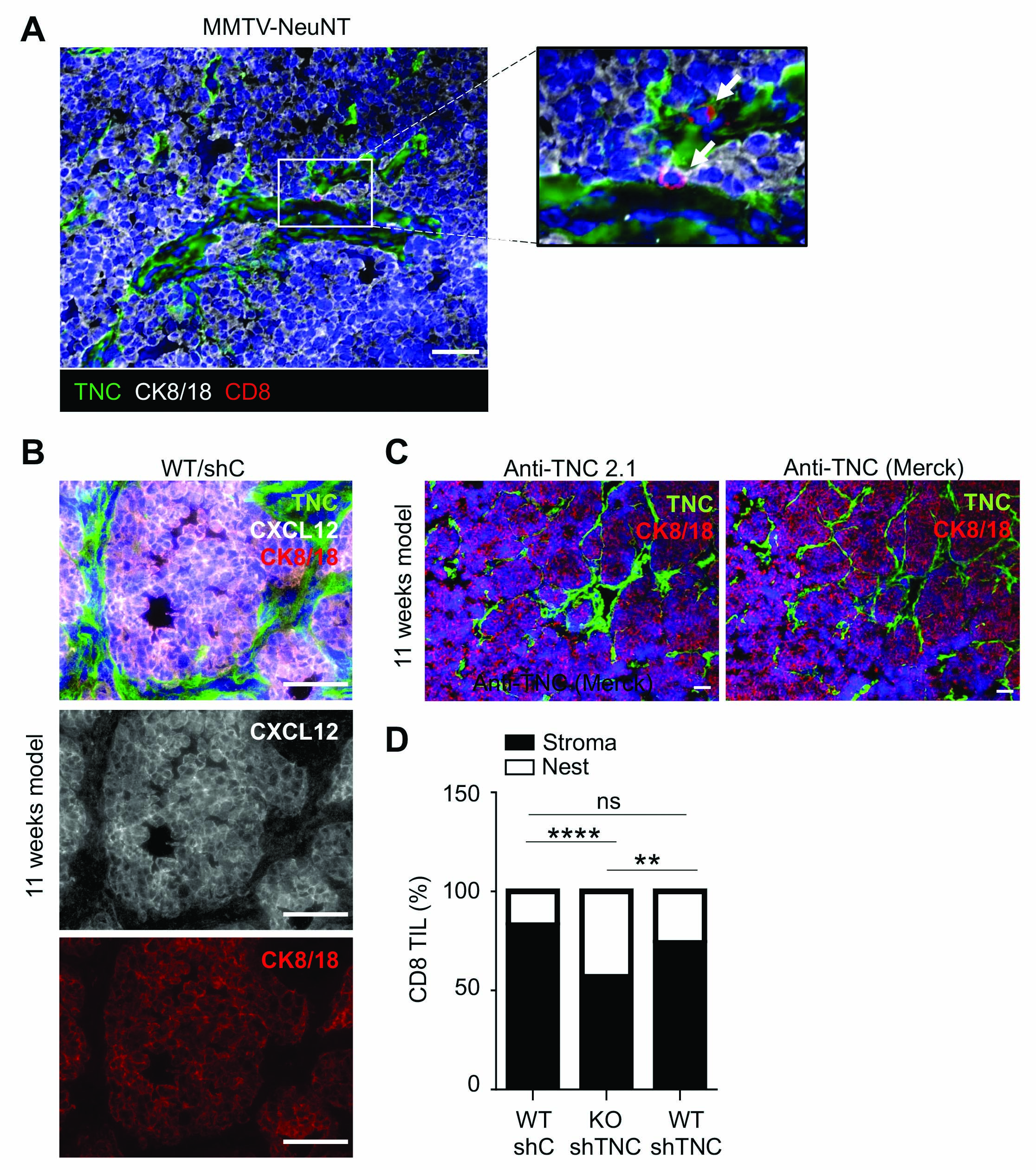
**

**Appendix Fig. S2 Expression of TNC and localisation of CD8 TIL in NeuNT and NT193 tumors (A)** Tissue staining of a NeuNT tumor for the indicated molecules, revealing very low abundance of CD8 TIL. Scael bar, x μm. **(B, C)** Staining of a TNC high (WT/shC) tumor for TNC with the rat monoclonal MTn12 **(B)** and rabbit polyclonal TNC2.1 and Merck antibodies **(C)** revealing expression of the TNC protein in the stroma but not in the tumor cell nests (CK8/18).

**Appendix Fig. S3**

**
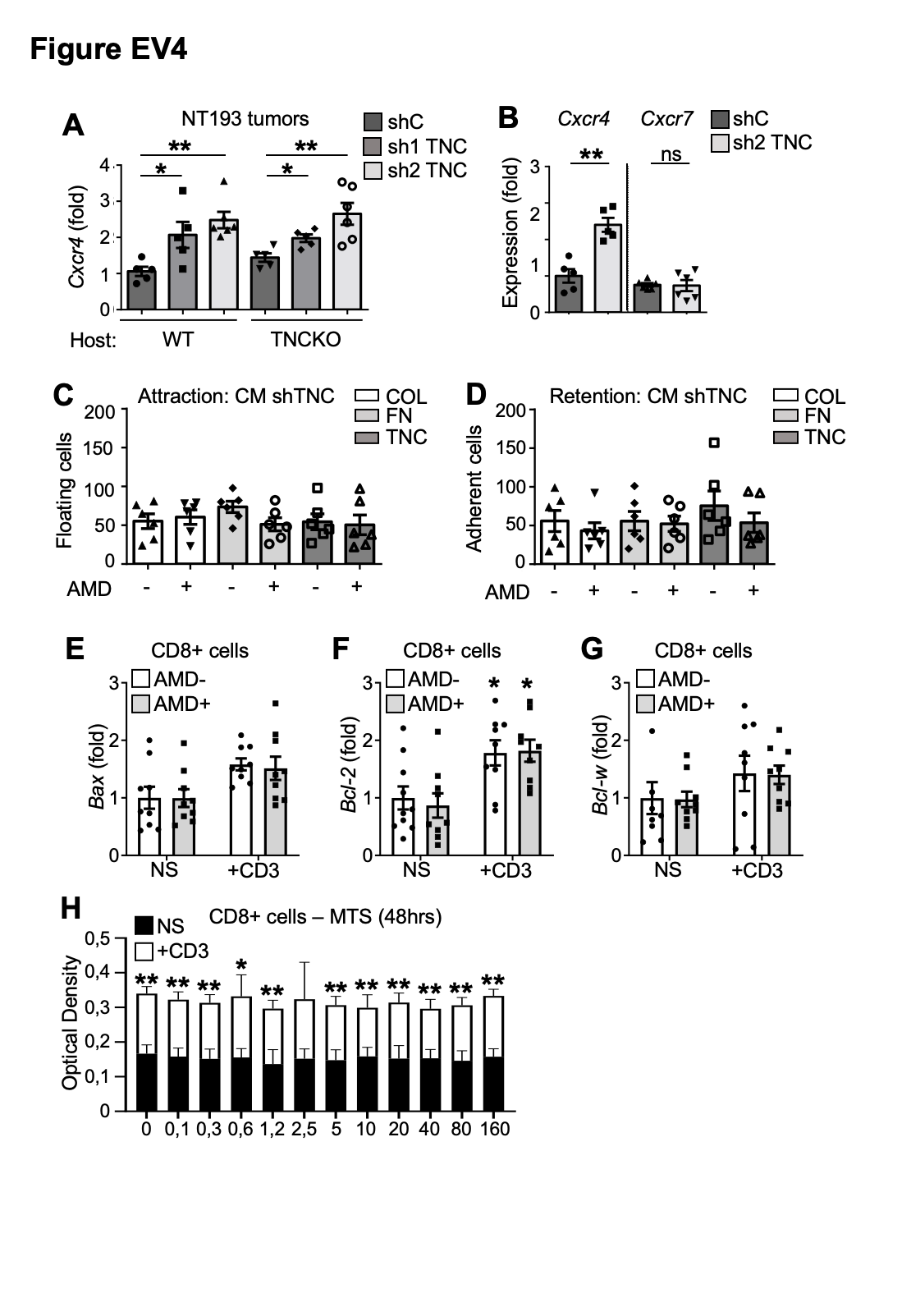
**

**
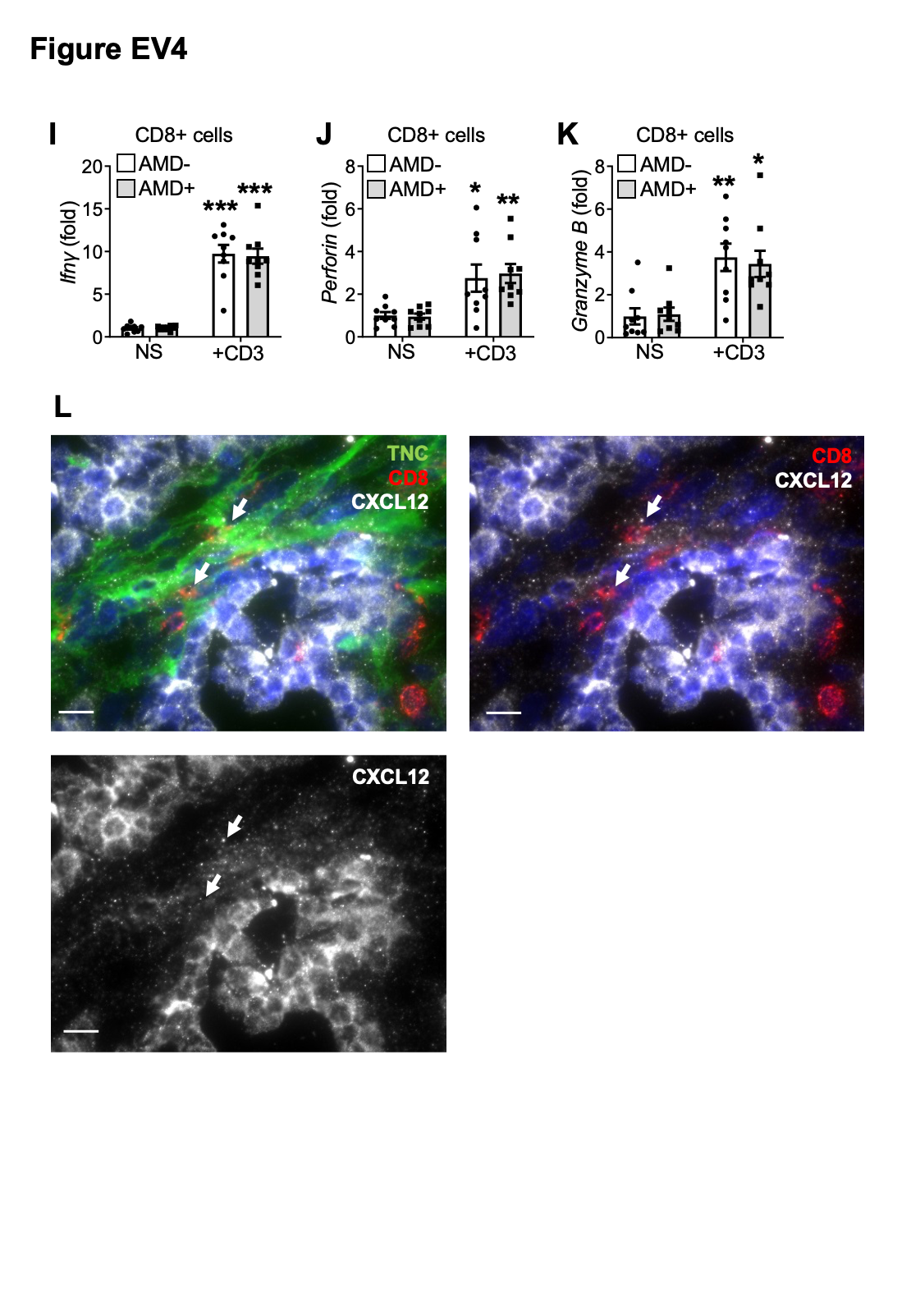
Appendix Figure S3 CXCL12 receptor expression and CD8 T leukocyte migration and function upon CXCR4 inhibition**

**(A, B)** mRNA levels of CXCR4 and CXCR7 **(B)** in NT193 tumors **(A)** and cultured NT193 cells **(B)**. **(A)** N = at least 5 tumors per group, ** p = 0.0043 (WT host, shC versus sh2TNC), * p = 0.0159 (WT host, shC versus sh1TNC), ** p = 0.0087 (TNCKO host, shC versus sh2TNC), * p = 0.0159 (TNCKO host, shC versus sh1TNC), Mann-Whitney test. **(B)** N = at least 5 tumors per group, ** p = 0.0079 **(C)** Migration and **(D)** retention of CD8 T leukocytes with CM from shTNC cells upon coating of the lower insert surface with the indicated matrix molecules. Cells were quantified upon addition of AMD3100 (AMD), 1 hour pretreatment (5 μg/ml AMD) for 5 hours, **(C)** N = 6 per group, t-test, ns > 0.05. **(D)** N = 6 per group, Mann-Whitney test, ns > 0.05. **(E – G, I - K)** Gene expression (qRTCPR) of *Bax* **(E),** *Bcl-2* **(F),** *Bcl-w* **(G),** *Ifnγ* **(I)**, *Perforin* **(J),** *Granzyme B* **(K)** in CD8 T cells treated *in vitro* with a CD3 mouse antibody (1µg/mL; +CD3) or not (Non-stimulated; NS) for 24 hours and incubation for 45 min with AMD (5 µg/mL; AMD+) or not (AMD-). N = 9 (3 independent experiments in triplicate). Values are normalized to *Gapdh* and are expressed as relative ratio to control CD8 T cells (NS, AMD-). Mean ± SEM, Two way-AVOVA and Tukey’s post-test. * relative to NS condition (* p <0,05; **p < 0.01; ***p < 0.005). **(H)** MTS cell proliferation of CD8 T cells after CD3+ stimulation or not and 48 hours of incubation with the indicated AMD concentrations (µg/mL, n = 2 experiments, n = 8 replicates). Two way-AVOVA and Tukey’s post-test, mean ± SEM, * relative to corresponding NS condition (* p < 0,05; **p < 0.01). **(L)** Representative staining image (N = 5 tumors) of a TNC+ tumor for the indicated molecules. Scale bars, 50 µm. Note punctate CXCL12 expression in the tumor cells and at lower level in the stroma colocalizing with CD8 and TNC (arrows).

**Appendix Fig. S4**

**
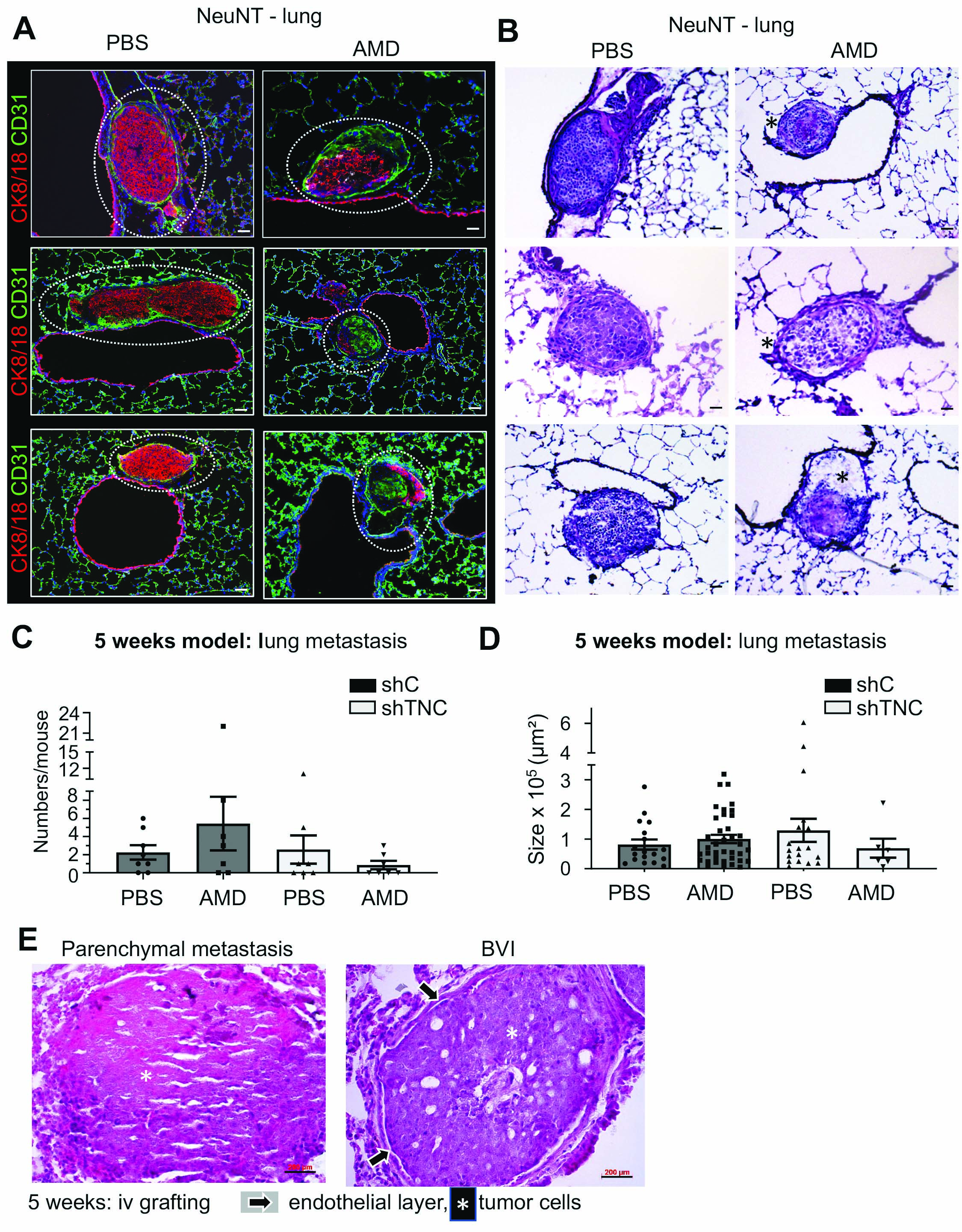
Appendix Figure S4 Impact of CXCR4 inhibition on BVI in NeuNT mice**

Metastasis assessment in lungs from NeuNT mice by IF **(A)** and H&E staining **(B).** BVI are encircled with a broken white line. Representative images of N = 5 mice, PBS and N = 5 mice, AMD, scale bar, 50µm. **(C-E)** 5 weeks model. Number **(C)** and size **(D)** of lung metastases upon iv grafting of shC or shTNC cells followed by stereological analysis by H&E staining. N = 8 mice (shC, PBS), N = 7 mice (shC, AMD), N = 7 mice (shTNC, PBS), N = 7 mice (shTNC, AMD). No statistical difference was observed by Kruskal Wallis statistical test, p > 0.05. **(E)** Representative image of parenchymal metastasis and BVI. Scale bar, 200µm.

**Appendix Fig. S5**

**C cc
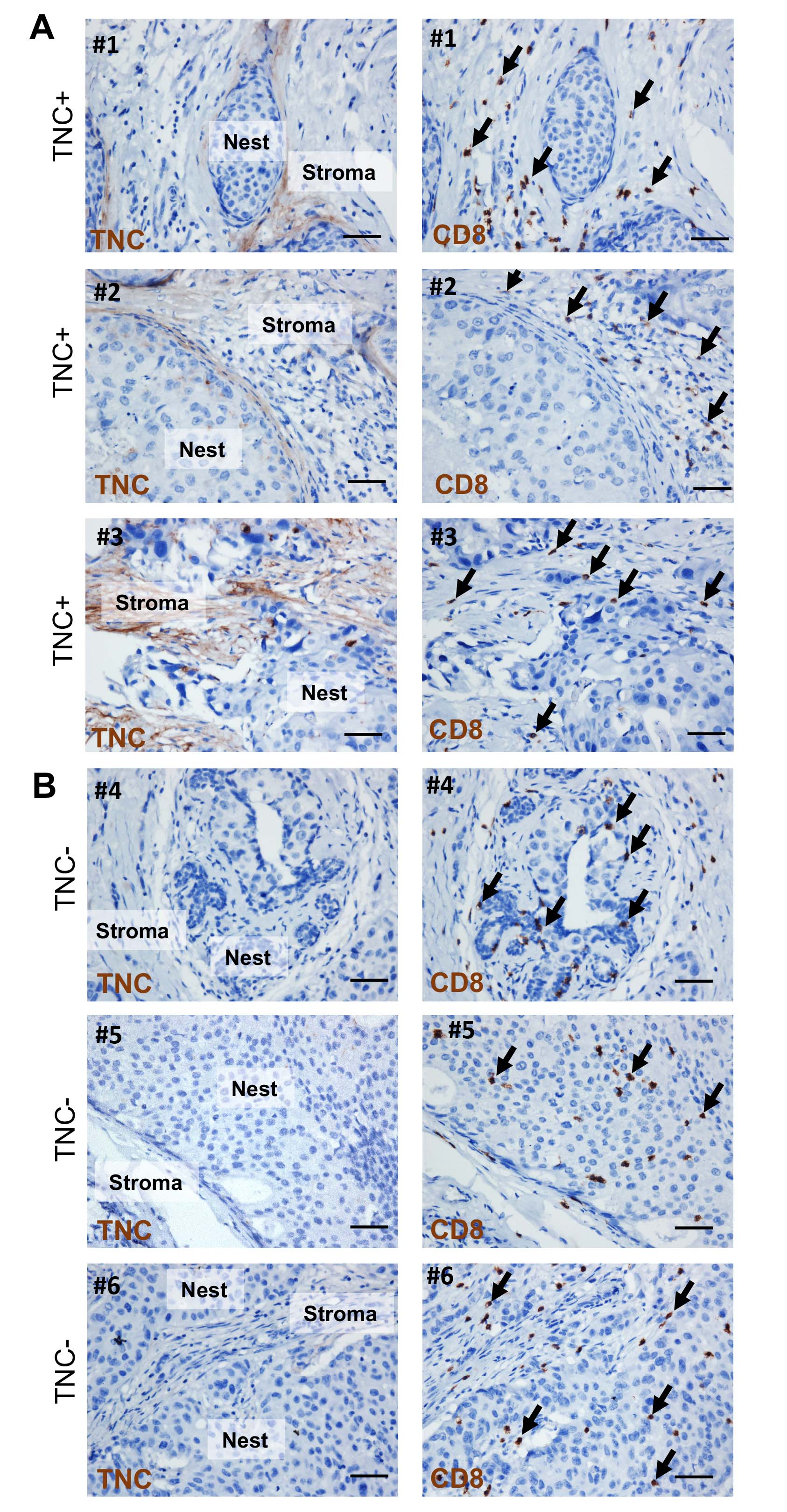
**

**
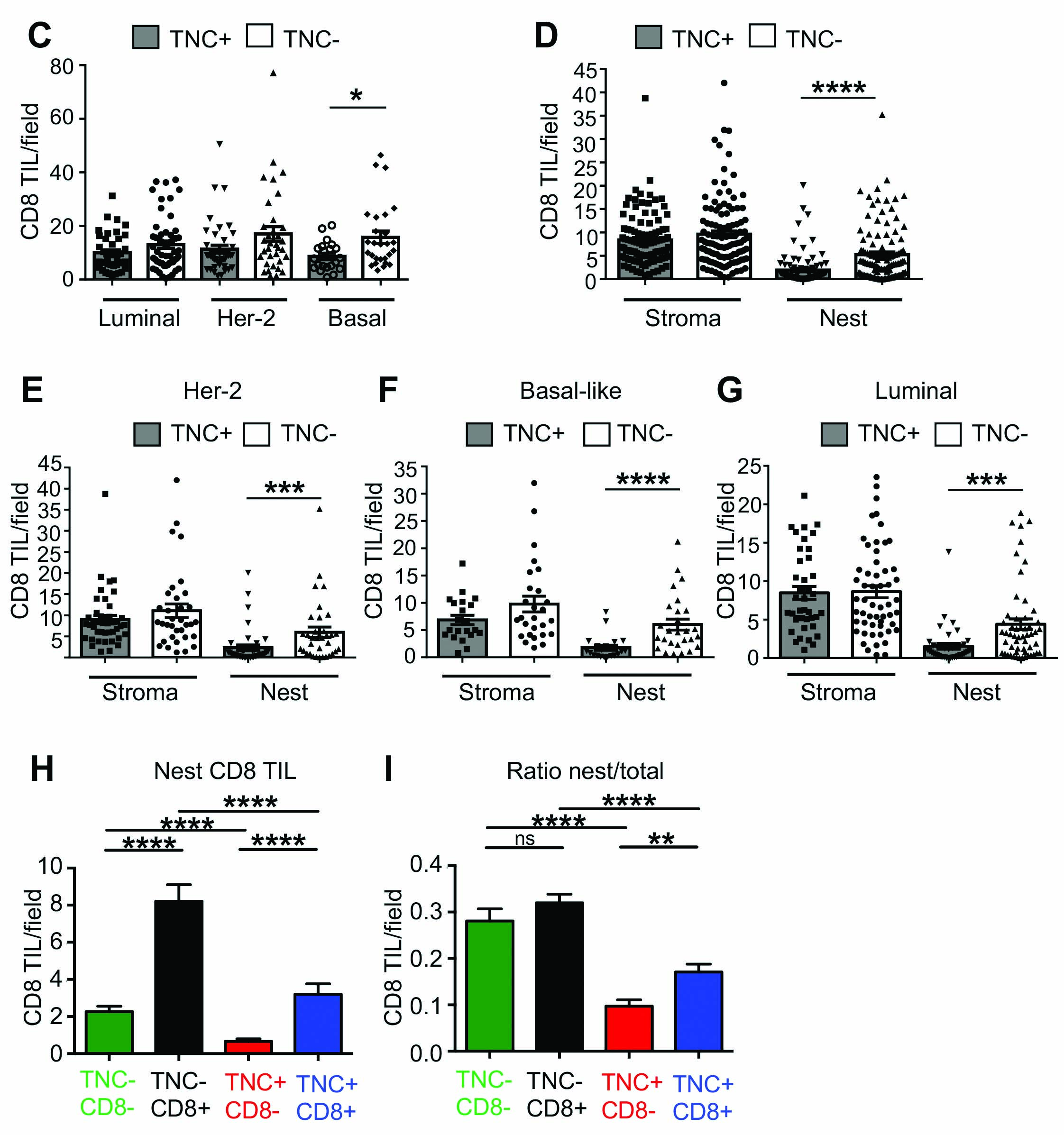
**

**
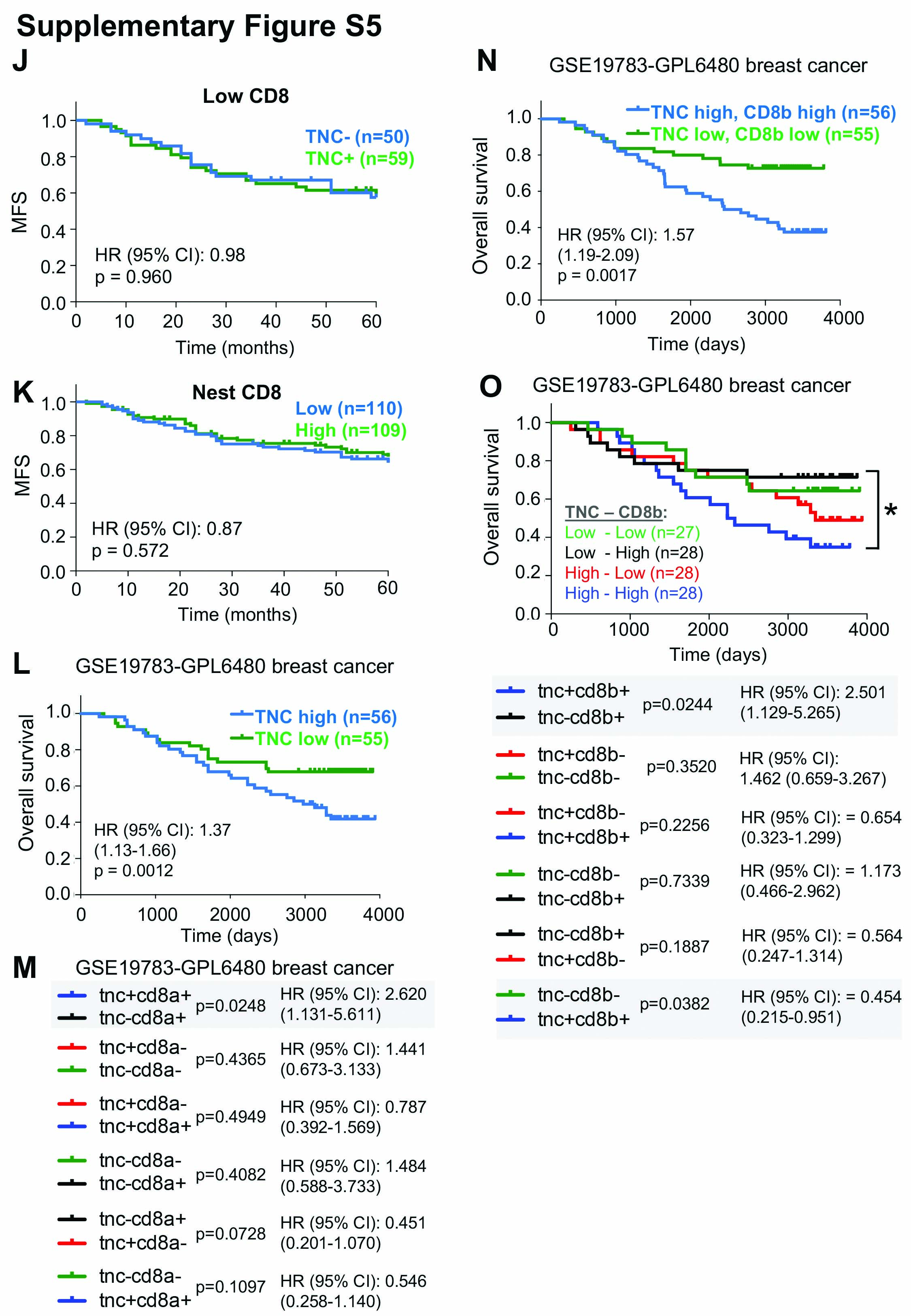

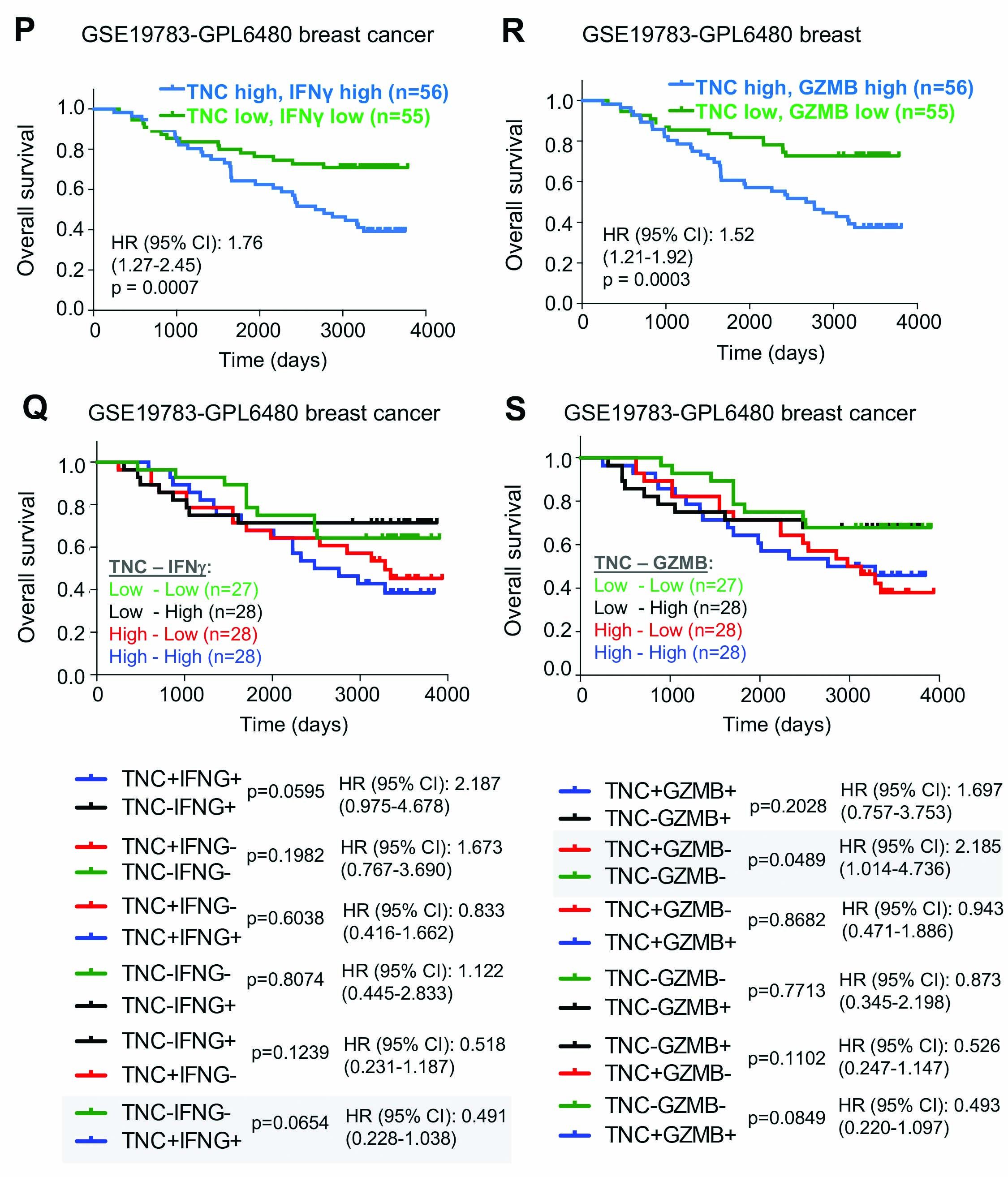

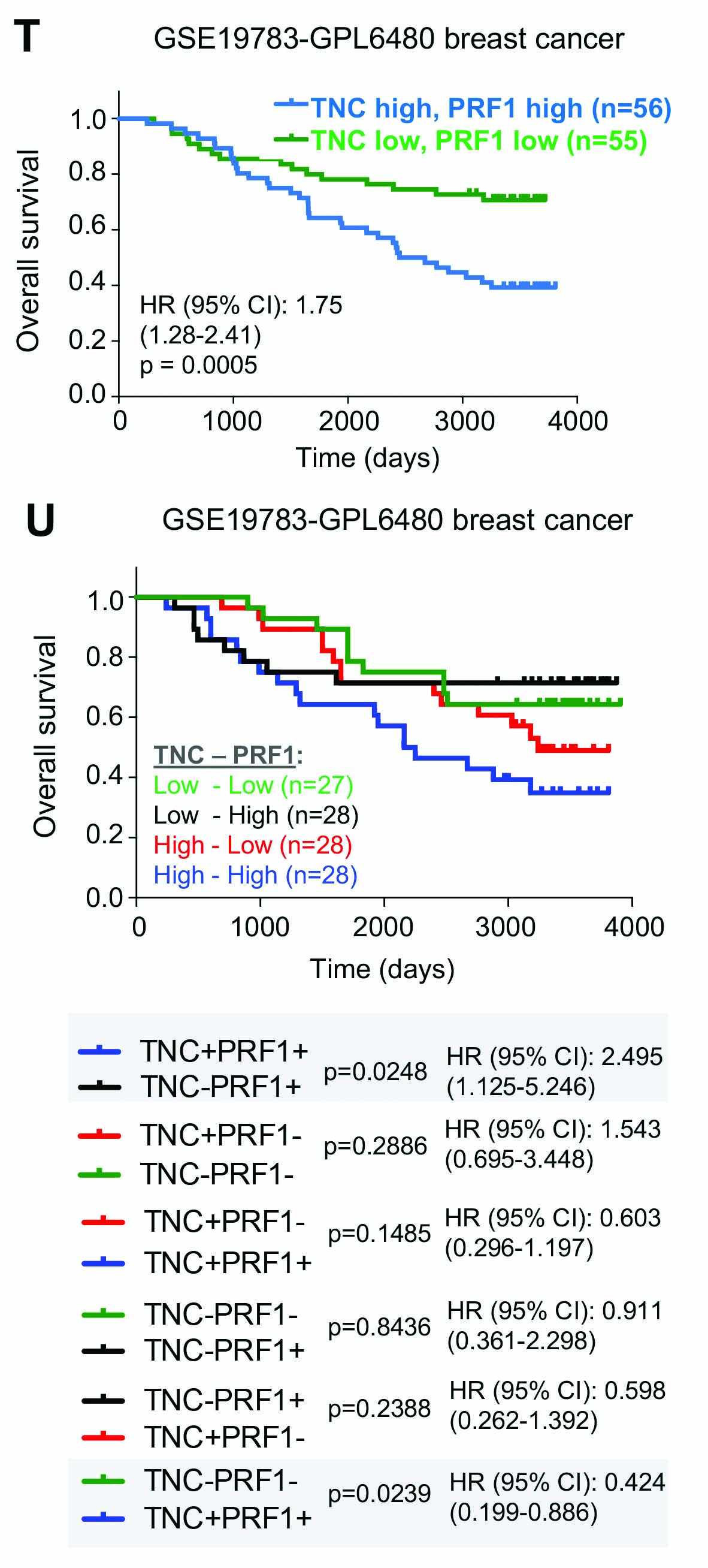
**

**Appendix Figure S5 Correlation of TNC expression and CD8 TIL abundance with breast cancer patient survival**

**(A, B)** Representative adjacent sections of human breast cancer tissue (TMA, N = 219) stained with antibodies specific for TNC or CD8. Scale bar = 50 µm. **(C - G)**Determination of CD8 TIL in TNC+ and TNC- tumors of the different molecular subtypes. **(C)** Total and **(D - I)** nest or stromal numbers of CD8 TIL. Nest CD8- or CD8+ or stromal CD8- or CD8+ is defined as below/above median of nest or stromal CD8 TIL per area. **(C)*** p = 0.0161**(D)****** p < 0.0001**(E)***** p = 0.0007**(F)****** p < 0.0001**(G)***** p = 0.0003, **(H, I)** **** p < 0.0001**,**** p < 0.01, Mann-Whitney test. **(J - U)**Survival analysis for the indicated molecules in patient cohort of the TMA **(J, K)**and cohort GSE19783 **(L - U)**. Values are shown above or below the median, HR and p values are indicated for the different comparisons. Log-rank test.

**Appendix Fig. S6**

**
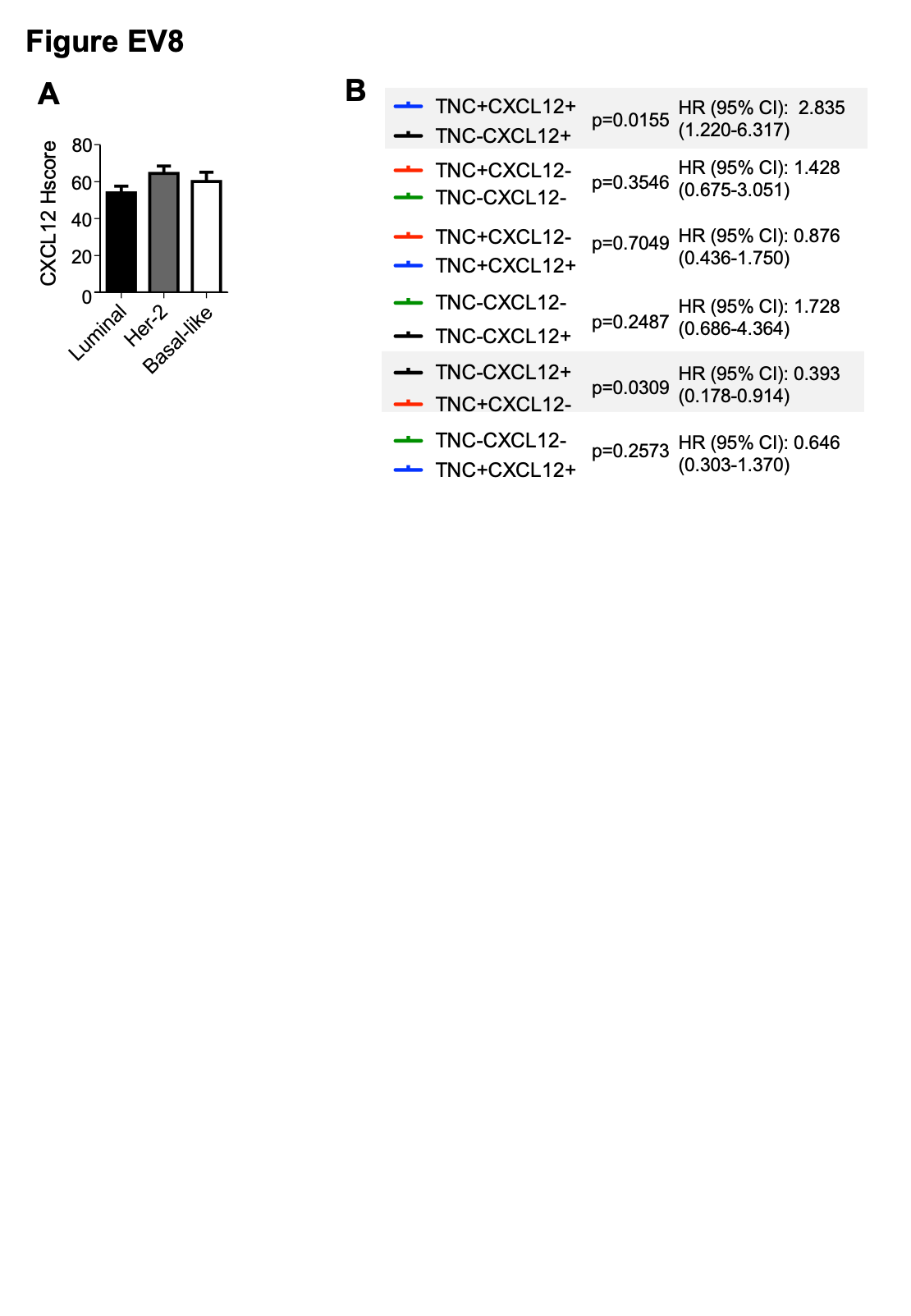
Appendix Figure S6** **Correlation of TNC and CXCL12 expression with breast cancer patient survival**

**(A)** Histological score of CXCL12 staining in different molecular subtypes of breast cancer patients, TMA cohort, one way Anova. p > 0.05. **(B)** HR and p values are indicated for the different comparisons of TNC and CXCL12 expression with survival used in **Fig. 8E**. Log-rank test.

**Appendix Table S1 Gene expression in MMTV-NeuNT tumors (WT versus TNCKO)**

RNA Affymetrix chip profiling data, p-value < 0.05, N = 2.

| **Gene Symbol** | **logFC** | **P.Value** |
| --- | --- | --- |
| 3110052M02Rik | -0.290697732 | 0.000132405 |
| Gm10509 | -0.360943619 | 0.000146001 |
| Cd59a | -0.240173247 | 0.000149773 |
| Slc6a9 | 0.325117146 | 0.000162493 |
| Zfp932 | -0.180907473 | 0.000181417 |
| Cetn2 | -0.233331174 | 0.000209572 |
| N6amt1 | -0.196585647 | 0.000223957 |
| Sumo1 | -0.237856847 | 0.000253319 |
| Zfp932 | -0.190879734 | 0.000258638 |
| Sumo1 | -0.20370483 | 0.000262108 |
| Myh1 | 0.428682715 | 0.000316298 |
| Nsun3 | -0.188036921 | 0.000391737 |
| Fundc2 | -0.430702378 | 0.000413596 |
| Ncapd2 | 0.157754298 | 0.00042026 |
| Megf6 | 0.25419361 | 0.00049259 |
| Trdn | 0.498926832 | 0.000522269 |
| Lgals7 | 0.466635571 | 0.000531149 |
| Itga2 | 0.23068653 | 0.000534866 |
| Mybpc2 | 0.285608645 | 0.00054718 |
| Ckm | 0.68340863 | 0.000549072 |
| Myoz1 | 0.313805753 | 0.000584811 |
| Guf1 | -0.193429281 | 0.000599944 |
| Gm17764 | -0.155729652 | 0.000602199 |
| Pdgfd | -0.193655644 | 0.000660832 |
| Megf6 | 0.219460668 | 0.000663301 |
| Fahd1 | -0.130886765 | 0.000673825 |
| Atoh8 | 0.180740712 | 0.000706005 |
| Gm6581 | -0.260061554 | 0.000734548 |
| Ly6d | 0.367356254 | 0.000748025 |
| Khnyn | 0.242926462 | 0.000752731 |
| Atp2a1 | 0.775398842 | 0.000762891 |
| Pcdhb7 | -0.30662622 | 0.000773267 |
| Zfp958 | -0.194396418 | 0.000775439 |
| Actn3 | 0.38771265 | 0.000803168 |
| Mpzl3 | -0.162500573 | 0.000829965 |
| Megf6 | 0.203050954 | 0.000849855 |
| Gyltl1b | 0.145290658 | 0.000854912 |
| Paip1 | -0.171132729 | 0.000867415 |
| Apoo | -0.206881201 | 0.000946445 |
| Neb | 0.411498826 | 0.000961841 |
| Basp1 | 0.399186238 | 0.001005419 |
| Ifi27l2a | -0.668887005 | 0.001035957 |
| Cldn10 | -0.202924644 | 0.001069053 |
| Apobec2 | 0.216980214 | 0.001080554 |
| Mthfd2 | 0.249907877 | 0.001111509 |
| Ddx23 | 0.150874979 | 0.001119675 |
| Troap | 0.153309525 | 0.001157455 |
| Sepsecs | -0.14027969 | 0.001197093 |
| Megf6 | 0.251997715 | 0.00127148 |
| Fem1c | 0.169168951 | 0.001273494 |
| Gm13235 | -0.340870478 | 0.001278799 |
| Slc33a1 | -0.198896289 | 0.001343561 |
| Ttll7 | 0.12422265 | 0.001367448 |
| Sarnp | -0.13837587 | 0.001377787 |
| Spa17 | -0.213575251 | 0.001380735 |
| Fbxl5 | -0.19791829 | 0.001380893 |
| Arrb1 | 0.19313149 | 0.001400062 |
| 3110082I17Rik | -0.174200881 | 0.001406755 |
| Zfp54 | -0.219303277 | 0.001416123 |
| Mtus1 | 0.211378544 | 0.001429715 |
| Acta1 | 0.843714088 | 0.001444262 |
| Megf6 | 0.163316527 | 0.001479235 |
| Ube2v2 | -0.18091188 | 0.001504858 |
| Sarnp | -0.134604415 | 0.001510723 |
| Rras2 | -0.158909635 | 0.001525449 |
| Tcp11l1 | 0.143407678 | 0.001528365 |
| Mtss1l | 0.349746306 | 0.00161101 |
| Ccdc12 | -0.177541019 | 0.001618604 |
| Gm10509 | -0.364937345 | 0.001639561 |
| Chac1 | 0.243455288 | 0.001658337 |
| Btc | -0.239773669 | 0.001663554 |
| Qdpr | -0.233678853 | 0.001666908 |
| Wdr81 | 0.150638714 | 0.001676744 |
| Zfp160 | -0.177819188 | 0.001708976 |
| Mrps18c | -0.191272703 | 0.001713199 |
| Tnnt3 | 0.360434145 | 0.001743335 |
| Ap1s3 | -0.146361507 | 0.001790113 |
| Smtn | 0.271439296 | 0.001808219 |
| Lypla1 | -0.277039639 | 0.001859575 |
| Runx3 | 0.149573172 | 0.001872701 |
| Pak6 | 0.241385202 | 0.001882342 |
| Cldn10 | -0.22076144 | 0.001930279 |
| Klhl21 | 0.246734613 | 0.001962738 |
| Megf6 | 0.241063543 | 0.002009163 |
| Slc30a5 | -0.170319926 | 0.00201849 |
| Zfp932 | -0.168912529 | 0.002020985 |
| Car3 | 0.902682201 | 0.00208449 |
| Ube2v2 | -0.17607406 | 0.002085063 |
| Gtpbp8 | -0.141660377 | 0.002102867 |
| Ndufa1 | -0.194150698 | 0.002110457 |
| Pnrc1 | 0.134804291 | 0.002153917 |
| Elovl1 | 0.126508862 | 0.002182256 |
| Tnnc2 | 0.207574678 | 0.002193253 |
| Ankrd11 | -0.186301728 | 0.002208561 |
| Msrb2 | -0.184477267 | 0.00221176 |
| Tgfb1 | 0.181422117 | 0.002222444 |
| Mir200c | 0.116516061 | 0.002244427 |
| Itga10 | 0.177228489 | 0.002262448 |
| Fam175a | -0.208067299 | 0.002277541 |
| Pdcd10 | -0.436265144 | 0.002286496 |
| Gpsm2 | 0.151570792 | 0.002329 |
| Rbm14 | 0.18351068 | 0.00233211 |
| Eln | 0.171497772 | 0.002338416 |
| Mrpl33 | -0.18300852 | 0.002349902 |
| P2rx7 | 0.134498154 | 0.002368719 |
| Fam36a | -0.131440803 | 0.002374417 |
| Riok2 | -0.157387056 | 0.002383768 |
| Trim41 | 0.141456706 | 0.002429833 |
| Bend6 | -0.333970625 | 0.002453661 |
| Tonsl | 0.219379323 | 0.002521155 |
| Espl1 | 0.12876869 | 0.002534258 |
| Gm10495 | -0.192706348 | 0.00253672 |
| Pvalb | 0.438939588 | 0.00258131 |
| Ccnl2 | 0.175537042 | 0.002597894 |
| Calcoco1 | 0.198488319 | 0.002610563 |
| Ccl21a | 0.184278099 | 0.002629758 |
| Trdmt1 | -0.23809325 | 0.0026694 |
| Hemk1 | -0.204123482 | 0.002671162 |
| Mki67ip | -0.171087876 | 0.002697834 |
| Mterfd1 | -0.131390173 | 0.002699469 |
| Arl6 | -0.172099174 | 0.002741394 |
| Atp5s | -0.144516331 | 0.002756487 |
| Megf6 | 0.283669204 | 0.002757687 |
| C1galt1c1 | -0.16458047 | 0.002768005 |
| Arhgap4 | 0.212181334 | 0.002772913 |
| Myom1 | 0.129230838 | 0.002813083 |
| Dcakd | 0.128245312 | 0.002820166 |
| Ube2w | -0.148486637 | 0.002827179 |
| Pstpip2 | -0.15298097 | 0.002885317 |
| Tmem177 | -0.217378048 | 0.002894662 |
| Itgax | 0.181362002 | 0.002953332 |
| Tnfsf10 | -0.435418254 | 0.002966278 |
| Apoo | -0.178552095 | 0.002973612 |
| Ch25h | -0.126158902 | 0.0030115 |
| Adamts3 | -0.259121077 | 0.003029123 |
| 4930455C21Rik | -0.175060484 | 0.00303966 |
| Hint3 | -0.188373433 | 0.003046452 |
| Kbtbd10 | 0.273613251 | 0.003055145 |
| Polr2m | -0.175049754 | 0.003070609 |
| Srsf4 | 0.133297006 | 0.003083848 |
| Aste1 | -0.15137986 | 0.003108606 |
| Aga | -0.153286955 | 0.003111854 |
| Orc5 | -0.261136209 | 0.003113542 |
| Tceal1 | -0.282517063 | 0.003114844 |
| H2-Ke6 | -0.233063552 | 0.003122509 |
| Wtap | -0.101996517 | 0.003157463 |
| BC026585 | -0.326337042 | 0.003165499 |
| Atg10 | -0.155584086 | 0.003205709 |
| Ccl25 | 0.158262475 | 0.003240401 |
| Uqcrb | -0.127407555 | 0.003241169 |
| Carf | -0.124113963 | 0.003324051 |
| Ptprf | 0.275917798 | 0.003335961 |
| Rraga | -0.117182358 | 0.003360312 |
| 4933404M02Rik | -0.127104248 | 0.003362635 |
| Cyp2j6 | -0.378926778 | 0.003386967 |
| 2510039O18Rik | 0.161678625 | 0.003389287 |
| Tmem33 | -0.134144597 | 0.003399014 |
| Tceb1 | -0.210246808 | 0.003402683 |
| Tmcc3 | 0.159267721 | 0.003420622 |
| Pter | -0.222568393 | 0.00343496 |
| Nfe2l1 | 0.165927116 | 0.00344273 |
| Hr | 0.27419489 | 0.00347944 |
| Szt2 | 0.126995149 | 0.003500153 |
| Szt2 | 0.123536789 | 0.003513705 |
| Thrap3 | 0.170971175 | 0.003516931 |
| Mki67ip | -0.178522698 | 0.003520714 |
| Tmem182 | 0.187027104 | 0.003522807 |
| Pck2 | 0.161839043 | 0.003555009 |
| 5430407P10Rik | 0.122969436 | 0.003562584 |
| 4933409K07Rik | 0.135622086 | 0.003580155 |
| 4933409K07Rik | 0.135622086 | 0.003580155 |
| 4933409K07Rik | 0.135622086 | 0.003580155 |
| 4933409K07Rik | 0.135622086 | 0.003580155 |
| Snx10 | -0.245059532 | 0.003618926 |
| Tmem60 | -0.271034897 | 0.003619686 |
| Zfp932 | -0.214969395 | 0.003635887 |
| Pex7 | -0.180408538 | 0.00364865 |
| Ttc30a2 | -0.147048606 | 0.003660917 |
| Ube2n | -0.205617938 | 0.003693949 |
| Zfp384 | 0.147062724 | 0.003699849 |
| Hmgb1 | -0.111318945 | 0.003737915 |
| 6720489N17Rik | -0.478987981 | 0.003763813 |
| AU019823 | -0.22613845 | 0.003797505 |
| Cnksr1 | 0.145269001 | 0.003841264 |
| Bcl11a | 0.204346984 | 0.003843947 |
| Ptpn6 | 0.098770626 | 0.003845714 |
| Ccdc125 | -0.207598077 | 0.003849791 |
| Tmem141 | -0.184037025 | 0.003902971 |
| Rsph9 | -0.215179652 | 0.003909497 |
| Prp2 | 0.230212488 | 0.003931913 |
| Plekha5 | 0.156204385 | 0.003985547 |
| Parl | -0.119642687 | 0.004023544 |
| Hmgb1 | -0.108688919 | 0.004028312 |
| Eno3 | 0.317404462 | 0.004100996 |
| Actn2 | 0.212622981 | 0.004102643 |
| Mrpl11 | -0.277455447 | 0.004132283 |
| Hmgb1 | -0.100675026 | 0.004142151 |
| Fmo1 | 0.16116194 | 0.004147751 |
| Slc1a5 | 0.210385719 | 0.004174346 |
| Psmb8 | -0.333041634 | 0.004202726 |
| Arpp19 | -0.272599792 | 0.004205674 |
| Mrpl30 | -0.184176792 | 0.00420936 |
| Mybpc1 | 0.194474835 | 0.004216431 |
| Hmgb1 | -0.119397317 | 0.004219909 |
| Ism1 | 0.143174284 | 0.004237529 |
| Arsg | 0.173944828 | 0.004255468 |
| Ndufaf1 | -0.105720704 | 0.00425565 |
| Nenf | -0.277889866 | 0.004257121 |
| Cpt1a | 0.184833935 | 0.004296782 |
| Eno1 | 0.101875631 | 0.004342723 |
| Alg5 | -0.277183376 | 0.004382189 |
| Cript | -0.151493112 | 0.004405554 |
| Rdh14 | -0.217715805 | 0.004420987 |
| Mtap6 | 0.2627016 | 0.00447556 |
| Lrp8 | 0.243251586 | 0.004517332 |
| Klhl7 | -0.168051921 | 0.004550338 |
| Pgrmc1 | -0.191272864 | 0.004599794 |
| Mrpl48 | -0.144946156 | 0.004607343 |
| Nol7 | -0.240927179 | 0.004620679 |
| Bsdc1 | 0.20748901 | 0.004649454 |
| Zfp947 | -0.179344374 | 0.004661851 |
| Cyp2r1 | -0.092772077 | 0.004697773 |
| Zfp119b | -0.284001267 | 0.004746345 |
| Ampd1 | 0.090135332 | 0.004750301 |
| Fam126b | -0.146861789 | 0.004762814 |
| Gm6548 | -0.193234265 | 0.004922209 |
| Ift52 | -0.156898818 | 0.00492288 |
| Gm12511 | 0.096505238 | 0.004935809 |
| Fam49a | 0.183383593 | 0.004941583 |
| Uqcrb | -0.09882308 | 0.004971202 |
| Wbp2 | 0.08498731 | 0.004998858 |
| Ptplad1 | -0.151840455 | 0.005046647 |
| Tmem167 | -0.213067862 | 0.00507357 |
| Hmgb1 | -0.090368468 | 0.005081835 |
| Ptafr | 0.346338566 | 0.005086151 |
| Laptm5 | 0.213909071 | 0.005090246 |
| Tmem14a | -0.097602222 | 0.005184735 |
| Gtpbp10 | -0.225169644 | 0.005187753 |
| Gm4802 | -0.157996641 | 0.005191222 |
| Hdac5 | 0.164088045 | 0.005213898 |
| Zfp119a | -0.094306079 | 0.005223288 |
| 1700084J12Rik | -0.242238416 | 0.005246812 |
| Hmgb1 | -0.116278225 | 0.005255192 |
| Coq2 | -0.276916357 | 0.005262459 |
| 1700034H14Rik | -0.235380606 | 0.005313993 |
| Ovgp1 | 0.166225165 | 0.005337089 |
| Vps26a | -0.133388143 | 0.005348156 |
| 1110032A03Rik | -0.197796454 | 0.005426307 |
| Gm10778 | -0.221695253 | 0.005435307 |
| Szt2 | 0.202243902 | 0.005454828 |
| 2010110P09Rik | 0.185108131 | 0.005483789 |
| Dnajc13 | -0.137959922 | 0.005503718 |
| Lhpp | -0.118398144 | 0.005507398 |
| Ndufs8 | -0.116402334 | 0.005512266 |
| Pcbp1 | 0.100037531 | 0.005531284 |
| Gtf3c3 | -0.150358884 | 0.005544271 |
| Gm10616 | 0.09762853 | 0.005551844 |
| Csrp1 | 0.129157131 | 0.005555342 |
| Vdr | 0.274046739 | 0.005560512 |
| Abcc3 | 0.203720684 | 0.005566528 |
| Tbc1d19 | -0.142280721 | 0.00558136 |
| 2700089E24Rik | -0.175094032 | 0.005587799 |
| Rsl1 | -0.289299419 | 0.00569638 |
| Ifit3 | -0.688680918 | 0.00573864 |
| Hfe2 | 0.139156218 | 0.005767272 |
| 1500011H22Rik | -0.148339264 | 0.005823562 |
| Sox11 | 0.101392655 | 0.005836251 |
| Psmc3ip | -0.148134718 | 0.005841802 |
| Zfp81 | -0.174309213 | 0.005878807 |
| Myot | 0.355136295 | 0.005901804 |
| Zfp511 | 0.110919681 | 0.005903026 |
| Usmg5 | -0.304864163 | 0.005904693 |
| Fam174a | -0.186542552 | 0.005913727 |
| Ppp1r36 | -0.281801239 | 0.005916631 |
| Klhl28 | 0.097541518 | 0.005930942 |
| Rfc3 | -0.277450458 | 0.005936051 |
| Zfp948 | -0.134845084 | 0.005940209 |
| Myom2 | 0.133816518 | 0.005955156 |
| Cetn3 | -0.195151001 | 0.005963634 |
| Ezh1 | 0.180530386 | 0.006008578 |
| Gtpbp10 | -0.226574389 | 0.006034431 |
| Brp44 | -0.20422454 | 0.006037329 |
| Eno1 | 0.095382254 | 0.006062067 |
| Dennd2d | -0.143120816 | 0.006107511 |
| Fancg | 0.137464364 | 0.006110858 |
| 2700081O15Rik | 0.132726255 | 0.006111953 |
| Mrps14 | -0.119612357 | 0.00611822 |
| Iqgap3 | 0.176359874 | 0.006145448 |
| Arhgap28 | 0.176897169 | 0.006145848 |
| Cib2 | -0.161345711 | 0.006146606 |
| Wwp2 | 0.203822679 | 0.006242339 |
| Cmya5 | 0.126336026 | 0.006265525 |
| Gpatch8 | 0.160525204 | 0.006298937 |
| Tle6 | 0.085515696 | 0.00633603 |
| Celsr2 | 0.265331048 | 0.006355867 |
| Hmgb1 | -0.091346859 | 0.006361548 |
| Slc38a1 | 0.185626992 | 0.006393861 |
| Apoe | 0.149454173 | 0.0064021 |
| Ube2n | -0.197486836 | 0.006425134 |
| Rnf139 | -0.147774931 | 0.006446444 |
| Tmem125 | 0.120551069 | 0.006450074 |
| Snrpc | -0.187611647 | 0.006452532 |
| Myo1d | 0.259670672 | 0.006509691 |
| Ccnh | -0.183612174 | 0.006533238 |
| Ftsjd1 | -0.232732084 | 0.006606703 |
| Zfp943 | -0.218260747 | 0.006618381 |
| Dnajc13 | -0.107844722 | 0.006626539 |
| 2310047B19Rik | -0.167957357 | 0.006638739 |
| Hiat1 | -0.156209474 | 0.006651464 |
| Hmgb1 | -0.099081106 | 0.006673927 |
| Hmgb1 | -0.099081106 | 0.006673927 |
| Axl | 0.124946155 | 0.006677617 |
| Atp5h | -0.190186366 | 0.006691565 |
| Fads6 | 0.24478605 | 0.006839643 |
| Hmgb1 | -0.084447868 | 0.006861106 |
| Tcf20 | 0.18732195 | 0.006879324 |
| Zfp141 | -0.18096264 | 0.006892177 |
| Hmgb1 | -0.112875213 | 0.006911316 |
| Capsl | -0.282340585 | 0.00693359 |
| Lifr | 0.18153409 | 0.006934576 |
| Gin1 | -0.098661583 | 0.006967091 |
| 4632419I22Rik | -0.091457431 | 0.006977419 |
| Sgpp1 | -0.105860009 | 0.006977744 |
| Mir713 | 0.131193988 | 0.006980512 |
| B3gnt2 | -0.156685865 | 0.007077463 |
| Gps2 | 0.108037731 | 0.007078133 |
| Iltifb | -0.088457501 | 0.007085071 |
| Ahcyl2 | 0.112177993 | 0.007097376 |
| Mr1 | -0.151798189 | 0.007100634 |
| Fbxl4 | -0.144650013 | 0.007102663 |
| 4632428N05Rik | 0.149835564 | 0.007103567 |
| Snord82 | -0.180251878 | 0.007144627 |
| Cdh19 | -0.241707417 | 0.007181985 |
| Adipoq | 0.398982806 | 0.007224779 |
| Casp4 | -0.407665572 | 0.007251394 |
| Tmco1 | -0.149165816 | 0.00727731 |
| Psmd9 | -0.132433727 | 0.007278005 |
| 2410042D21Rik | -0.151853622 | 0.007288164 |
| Hmgb1 | -0.08710479 | 0.007289776 |
| Zkscan5 | -0.086405126 | 0.007289917 |
| Txndc9 | -0.177137704 | 0.007301691 |
| Hmgb1 | -0.092678933 | 0.007340111 |
| Hmgb1 | -0.096671594 | 0.007341283 |
| Tnpo2 | 0.123840512 | 0.00738361 |
| Tob1 | 0.081994042 | 0.007434255 |
| Cdk7 | -0.100874201 | 0.007447848 |
| Foxj3 | 0.132807508 | 0.007449133 |
| Mir187 | 0.10456805 | 0.007474025 |
| Taldo1 | -0.169026614 | 0.007489021 |
| Rpl22l1 | -0.194438984 | 0.007503738 |
| Slc24a6 | 0.142759924 | 0.007514463 |
| Zfp1 | -0.138608019 | 0.007515909 |
| 1110004F10Rik | -0.234830231 | 0.007566185 |
| Ephx2 | -0.153733934 | 0.007636282 |
| Ankrd23 | 0.320184369 | 0.007645717 |
| Prickle3 | 0.133168184 | 0.007680651 |
| Mia1 | -0.421164964 | 0.007700439 |
| Chmp5 | -0.083393461 | 0.007744606 |
| Creb3l2 | 0.196645906 | 0.007771771 |
| Cd96 | -0.132572831 | 0.007775264 |
| Mertk | 0.140119457 | 0.007806684 |
| Bud31 | -0.239980133 | 0.007816264 |
| C3 | 0.454794573 | 0.007864863 |
| Ccdc122 | -0.353073679 | 0.007914616 |
| L1cam | 0.339439644 | 0.007933076 |
| Cited2 | 0.099691524 | 0.008007814 |
| Stc2 | 0.258780791 | 0.008016295 |
| Cops5 | -0.205807975 | 0.008036482 |
| Gm15421 | -0.201774236 | 0.008062183 |
| Mpv17 | -0.180330495 | 0.008063491 |
| Brp44l | -0.269740336 | 0.008069353 |
| Ube2v2 | -0.23446517 | 0.008071918 |
| 4930455F23Rik | -0.167498781 | 0.008077303 |
| Gtf3c6 | -0.263347308 | 0.008163731 |
| Vma21 | -0.187668176 | 0.008189794 |
| Slc25a4 | -0.118009044 | 0.008216049 |
| Tspan14 | 0.12648838 | 0.008240885 |
| Zfp763 | -0.184768257 | 0.00824336 |
| Atxn7l3b | -0.15173758 | 0.008250383 |
| 1810035L17Rik | -0.24403198 | 0.008263841 |
| Gpbp1 | -0.123312544 | 0.008295781 |
| Rnu2-10 | -0.313121803 | 0.008302576 |
| A730085E03Rik | 0.114966344 | 0.008334403 |
| Snord87 | -0.300137101 | 0.008362929 |
| Gnpnat1 | -0.194093035 | 0.00836377 |
| Stx7 | -0.146268066 | 0.008384688 |
| Hmgb1 | -0.082817358 | 0.008407315 |
| Tmsb15l | -0.257977342 | 0.008414027 |
| 1810013D10Rik | -0.256675848 | 0.008422096 |
| Pcdhb12 | -0.299338547 | 0.008433561 |
| 9430020K01Rik | 0.155720352 | 0.008436444 |
| Wbscr16 | -0.10226129 | 0.008440503 |
| Cisd1 | -0.136097845 | 0.008447214 |
| Ttn | 0.411827209 | 0.008462771 |
| Hspa8 | -0.092251283 | 0.008477435 |
| N4bp1 | 0.121249314 | 0.008496239 |
| Mospd4 | 0.161275195 | 0.008527327 |
| Hmgb1 | -0.084470461 | 0.008542075 |
| Sdr39u1 | -0.212313407 | 0.00857461 |
| Ifi204 | -0.111845774 | 0.008582382 |
| Zfp820 | -0.269360736 | 0.008675816 |
| Ggt6 | 0.210834254 | 0.008679296 |
| Tfam | -0.344663981 | 0.008684956 |
| Spsb1 | 0.138150345 | 0.008694365 |
| Xirp1 | 0.078865883 | 0.008700361 |
| Cnot4 | 0.113410908 | 0.00871325 |
| Aida | -0.211673784 | 0.008729555 |
| Recql4 | 0.083029026 | 0.0087571 |
| Arntl | 0.413045296 | 0.008809524 |
| Git1 | 0.116368767 | 0.00883154 |
| Tmem17 | -0.133459142 | 0.008839312 |
| Pla2g6 | 0.219337861 | 0.008871472 |
| Hmcn1 | 0.075523179 | 0.008888212 |
| Tapt1 | -0.134274795 | 0.008895639 |
| Steap4 | 0.272705425 | 0.008924498 |
| Zfp334 | 0.304283319 | 0.008927551 |
| Zmynd17 | 0.137494645 | 0.008929313 |
| Lig3 | 0.136170801 | 0.008954849 |
| Msl1 | 0.111507859 | 0.008956236 |
| 2410127L17Rik | -0.139317235 | 0.008976669 |
| Zfp719 | -0.245193152 | 0.00899051 |
| 2500003M10Rik | -0.083921948 | 0.008994704 |
| Pygm | 0.33925434 | 0.009040955 |
| Vmn2r29 | -0.134035533 | 0.009085744 |
| Cidec | 0.38347441 | 0.009138044 |
| 1110058L19Rik | -0.204708776 | 0.009148756 |
| Cox16 | -0.154827594 | 0.009167184 |
| Ppil3 | -0.217451928 | 0.009168744 |
| Tnni2 | 0.179492754 | 0.009215676 |
| 9830147E19Rik | -0.166853144 | 0.009229895 |
| Abcg1 | 0.158648692 | 0.009260368 |
| Fam92a | -0.223111131 | 0.009265957 |
| Slc15a2 | 0.228815731 | 0.009277027 |
| Pex1 | 0.152975969 | 0.009325158 |
| Atp5l | -0.276585111 | 0.009442181 |
| Cxcl9 | -0.406757652 | 0.009498206 |
| Snrpc | -0.298984735 | 0.009544414 |
| Mkrn1 | 0.127473399 | 0.009560273 |
| 1110002B05Rik | -0.23824676 | 0.00957494 |
| Myl1 | 0.960570232 | 0.009589922 |
| Ift74 | -0.098972857 | 0.009592361 |
| Setd5 | 0.167397261 | 0.009604517 |
| Actn1 | 0.126950251 | 0.009607575 |
| Eif2s2 | -0.154498861 | 0.009623683 |
| Ikzf5 | -0.240128424 | 0.009634119 |
| Vamp4 | -0.150927994 | 0.00967227 |
| Hmgb1 | -0.096956456 | 0.00968767 |
| Atp5f1 | -0.157380564 | 0.0096982 |
| Chm | -0.111061212 | 0.009707368 |
| Fam181b | -0.129077784 | 0.009720771 |
| Fam53b | 0.21218986 | 0.009758161 |
| Pigh | -0.1543494 | 0.009764587 |
| Sp1 | 0.116241126 | 0.0097985 |
| Ggcx | 0.190992342 | 0.009848683 |
| Gm20091 | -0.104339881 | 0.009863715 |
| Adam33 | 0.110191817 | 0.009874454 |
| 4930420K17Rik | -0.242154357 | 0.009879601 |
| Rad17 | -0.103446915 | 0.009894898 |
| Il16 | 0.127254122 | 0.00991909 |
| 4930528F23Rik | -0.124413088 | 0.009921195 |
| Flcn | 0.131646368 | 0.009924181 |
| Fam115a | 0.24133834 | 0.009926375 |
| Rpl5 | -0.15690145 | 0.00993322 |
| Mylpf | 0.138732681 | 0.009976071 |
| Hmgb1 | -0.117407297 | 0.009988897 |
| Rhot1 | -0.131601692 | 0.010008584 |
| Psma1 | -0.163747262 | 0.010082229 |
| Etohi1 | -0.332733316 | 0.010105769 |
| Gnrh1 | 0.133709876 | 0.010173766 |
| Auh | -0.186799851 | 0.010182196 |
| Romo1 | -0.170996121 | 0.010252323 |
| Cox7a1 | -0.371836895 | 0.010255591 |
| Unc50 | -0.156173736 | 0.010256585 |
| I830012O16Rik | -0.786875039 | 0.010260153 |
| H2-K1 | -0.13304954 | 0.010284361 |
| Ndufb4 | -0.178496512 | 0.010312098 |
| Pamr1 | 0.32140246 | 0.010316316 |
| A930004D18Rik | 0.093138847 | 0.010366899 |
| Timm9 | -0.153780315 | 0.010373175 |
| Cr1l | -0.247352968 | 0.010387732 |
| 4933407C03Rik | 0.221601435 | 0.010388942 |
| Gas2 | -0.111276415 | 0.010396989 |
| Echdc3 | 0.164011115 | 0.01040402 |
| B230307C23Rik | -0.2499718 | 0.010421712 |
| Hmgb1 | -0.093120625 | 0.010446528 |
| Arih2 | 0.090101098 | 0.010471497 |
| Timm17a | -0.116634578 | 0.010511849 |
| Zfp945 | -0.110378052 | 0.010529869 |
| Pcbp2 | 0.134737473 | 0.010595924 |
| Llph | -0.1456415 | 0.010599645 |
| Hist1h3h | 0.079563849 | 0.010612647 |
| 4632415L05Rik | -0.097566939 | 0.010613969 |
| Hmbs | -0.167893097 | 0.0106297 |
| Fgf7 | 0.154492586 | 0.010646573 |
| Snx2 | -0.176286334 | 0.010651302 |
| Lztfl1 | -0.200178923 | 0.010652629 |
| 4933407C03Rik | 0.221505155 | 0.010670116 |
| Clic3 | 0.1148519 | 0.010670306 |
| Hmgb1 | -0.082857194 | 0.010670944 |
| Eri2 | -0.11415475 | 0.010693572 |
| Zfp691 | 0.156826097 | 0.010709545 |
| Zfp229 | -0.33374998 | 0.010712808 |
| Hddc2 | -0.252788817 | 0.010735927 |
| Atad1 | -0.096880406 | 0.010737787 |
| Psmb9 | -0.327639339 | 0.010752209 |
| Hist1h3b | 0.082045903 | 0.010773727 |
| Hmgb1 | -0.083034177 | 0.010807016 |
| Rnf13 | -0.2374266 | 0.010851613 |
| Megf6 | 0.146490318 | 0.010871605 |
| H2-Q5 | -0.23133782 | 0.010876188 |
| Creb1 | 0.094755279 | 0.01091081 |
| Spry2 | -0.172610759 | 0.010911771 |
| Zfp141 | -0.165590583 | 0.010932043 |
| Usmg5 | -0.290531272 | 0.010932767 |
| Ndufs1 | -0.131739775 | 0.010947874 |
| Sidt2 | 0.12541482 | 0.010953377 |
| Fam53c | 0.094662866 | 0.010986905 |
| Ptpn13 | 0.147409192 | 0.011043864 |
| Kdm6b | 0.087505775 | 0.011048282 |
| Trappc9 | 0.159502205 | 0.011139681 |
| Snupn | -0.199213756 | 0.01116508 |
| Senp3 | -0.130898304 | 0.011188262 |
| Kif13b | 0.169245298 | 0.011192407 |
| Gm5068 | -0.217166675 | 0.011198496 |
| Pum1 | 0.160760706 | 0.01119927 |
| Hmgb1 | -0.091505357 | 0.011229386 |
| Mthfr | 0.169192798 | 0.01124704 |
| Avpi1 | -0.261782772 | 0.011248475 |
| Bbs10 | -0.224963153 | 0.011336094 |
| Tmed9 | -0.081404151 | 0.011344832 |
| Mrpl1 | -0.138915961 | 0.011350058 |
| Plcd3 | 0.129231438 | 0.011357287 |
| Ufm1 | -0.149450763 | 0.011359058 |
| Stk16 | -0.125567051 | 0.011363612 |
| Phyhd1 | -0.16274306 | 0.011377528 |
| Rnf34 | -0.183648314 | 0.011383895 |
| Gm7285 | -0.374337571 | 0.01140134 |
| Hist1h3c | 0.081983259 | 0.011419449 |
| Tagap1 | -0.150158772 | 0.011435374 |
| Gm5665 | -0.202505168 | 0.011441791 |
| Stk19 | -0.098466735 | 0.011442206 |
| Ndufb5 | -0.176759307 | 0.011465622 |
| Mrpl40 | -0.21152065 | 0.011545057 |
| Adam15 | 0.116639241 | 0.011569591 |
| Tlr6 | -0.177283284 | 0.011582059 |
| Sec22a | -0.230762806 | 0.011587192 |
| Atp5l | -0.30668794 | 0.011618967 |
| Ldb3 | 0.1425281 | 0.011619692 |
| Glmn | -0.14607491 | 0.011644432 |
| Rny1 | -0.599297979 | 0.011658175 |
| Rpl9 | -0.196174763 | 0.011661472 |
| Rere | 0.218087451 | 0.011689261 |
| Gzmb | -0.513006928 | 0.011689516 |
| Mpped2 | -0.183118261 | 0.011689878 |
| Ccdc32 | -0.205216757 | 0.011730255 |
| Cxcl12 | 0.274306633 | 0.011742335 |
| Timm8a1 | -0.197690206 | 0.011765679 |
| Morn2 | -0.210076022 | 0.01180383 |
| Zfp738 | -0.185879736 | 0.011838767 |
| Syk | 0.156875166 | 0.011844044 |
| Uqcr11 | -0.238736564 | 0.011850676 |
| Zfp40 | -0.134312264 | 0.012090588 |
| Cops4 | -0.170923574 | 0.012092518 |
| Lmf2 | 0.140294394 | 0.012100506 |
| Bex1 | -0.223844529 | 0.012121706 |
| Tgtp1 | -0.62502761 | 0.012144972 |
| Pacrgl | -0.105258646 | 0.012192564 |
| Atg5 | -0.20024739 | 0.012212839 |
| Hist2h3b | 0.078923882 | 0.01222184 |
| Trio | 0.170532849 | 0.012254064 |
| Olfr111 | 0.129417893 | 0.012259367 |
| Ldb1 | 0.094249866 | 0.012298327 |
| Pigy | -0.109502964 | 0.012301339 |
| Rnu2-10 | -0.278631311 | 0.012319046 |
| 2410015M20Rik | -0.239512622 | 0.012347641 |
| Zfp709 | -0.176072551 | 0.012423992 |
| Apip | -0.221326231 | 0.012436366 |
| Npepps | 0.155268332 | 0.012438659 |
| Atp5h | -0.181277355 | 0.012440896 |
| Ttll12 | 0.174418521 | 0.012453445 |
| Usp19 | 0.092682908 | 0.012459879 |
| Tmem70 | -0.102569279 | 0.012491576 |
| Tatdn3 | -0.128275594 | 0.012507979 |
| 2310030G06Rik | -0.168867815 | 0.012540126 |
| Isg20 | 0.133684902 | 0.012596916 |
| Pcsk6 | 0.214997198 | 0.012612251 |
| Jup | 0.164596821 | 0.012620088 |
| Spag1 | 0.12409046 | 0.01265023 |
| Arhgef10l | 0.179890496 | 0.012653485 |
| Pts | -0.284654469 | 0.012666103 |
| Cmpk1 | -0.20946941 | 0.012756777 |
| Zfp760 | -0.124868214 | 0.012760522 |
| Cox6b1 | -0.139811388 | 0.012797824 |
| Snrpe | -0.189547609 | 0.012814232 |
| Fam83h | 0.143589243 | 0.012837996 |
| Snx3 | -0.122979275 | 0.012870804 |
| Mrpl46 | -0.164996034 | 0.012904796 |
| Mosc2 | -0.251050834 | 0.012944274 |
| Crot | -0.285644203 | 0.012967088 |
| Acrbp | 0.188333494 | 0.012970968 |
| BC004004 | -0.132520881 | 0.013003884 |
| H2-T23 | -0.150570507 | 0.01300916 |
| Tyk2 | 0.097735706 | 0.013012998 |
| Rarg | 0.220145751 | 0.013083441 |
| Maob | 0.105325806 | 0.013098505 |
| Gas8 | 0.097574109 | 0.013103163 |
| Letmd1 | 0.170698868 | 0.013108333 |
| Efhd2 | 0.178016126 | 0.013114477 |
| Adam15 | 0.092768206 | 0.013152299 |
| Psph | -0.118481516 | 0.013191449 |
| Snapc3 | 0.184191285 | 0.013213319 |
| Aldh1l2 | 0.393809352 | 0.01322225 |
| Esco1 | -0.141097143 | 0.013228901 |
| Ahdc1 | 0.155842629 | 0.013231367 |
| Trappc2 | -0.178573071 | 0.01323907 |
| Siglec1 | 0.177037203 | 0.013251733 |
| Gpatch8 | 0.144872943 | 0.013272084 |
| Mtch2 | -0.170915122 | 0.013312792 |
| Ttc30b | -0.09960608 | 0.013315358 |
| Gpn1 | -0.142661468 | 0.013341811 |
| Ndufb4 | -0.178991132 | 0.013349684 |
| Mir134 | 0.154777311 | 0.013359742 |
| Tubgcp4 | 0.067273037 | 0.013378397 |
| Irgm1 | -0.442921563 | 0.013406673 |
| Hmgb1 | -0.074973874 | 0.01341601 |
| Extl2 | -0.111132168 | 0.01341727 |
| Gm10847 | 0.09505463 | 0.01342566 |
| Glipr2 | 0.119400901 | 0.01342761 |
| Treml4 | 0.361702314 | 0.01342929 |
| Tbce | -0.099706547 | 0.013450209 |
| Sepn1 | 0.135593021 | 0.01349302 |
| H47 | -0.200146163 | 0.013503813 |
| Cyb5r3 | 0.275416326 | 0.013519716 |
| Ubn2 | 0.215059786 | 0.013543956 |
| Fxyd1 | 0.15399956 | 0.01361515 |
| Szt2 | 0.183301817 | 0.013621633 |
| Oplah | 0.106525257 | 0.013640073 |
| Vamp2 | 0.101357309 | 0.013694577 |
| Tmem38b | -0.414947484 | 0.013733032 |
| Dpysl2 | 0.251519212 | 0.013748374 |
| Ints9 | 0.090014326 | 0.013755466 |
| Rasl12 | -0.115570516 | 0.013811892 |
| Mrpl27 | -0.136820296 | 0.013831382 |
| Arhgef17 | 0.263667928 | 0.013872802 |
| Zfp677 | -0.127460574 | 0.01387837 |
| Krt80 | 0.107595472 | 0.013908635 |
| Gm10767 | -0.152720467 | 0.013919447 |
| Zfp960 | -0.346795481 | 0.01395142 |
| Mir345 | 0.164598999 | 0.013954466 |
| Stk10 | 0.177175914 | 0.01396366 |
| Cox7c | -0.074903919 | 0.014008505 |
| Ticam2 | 0.118970213 | 0.014013473 |
| Pnkd | -0.164028579 | 0.014054609 |
| Mxi1 | 0.114511137 | 0.014058877 |
| Arhgef3 | 0.222020427 | 0.014143524 |
| Rnu2-10 | -0.287000356 | 0.014149274 |
| Fmo5 | -0.134423125 | 0.014153178 |
| Zfp11 | -0.121883213 | 0.014191297 |
| Zfp35 | -0.135361157 | 0.014193442 |
| Snord52 | -0.157251299 | 0.014205202 |
| H2-T10 | -0.317925725 | 0.014240235 |
| Mtrf1l | -0.072244881 | 0.014245372 |
| Cd37 | 0.136708916 | 0.014250834 |
| Tsg101 | -0.178837239 | 0.014270406 |
| Hist1h3e | 0.081180407 | 0.014273038 |
| Eef1e1 | -0.189306463 | 0.014282259 |
| Szt2 | 0.182248739 | 0.014286193 |
| Cblb | 0.23252285 | 0.014297632 |
| Pof1b | -0.137062255 | 0.014301506 |
| Pnpt1 | -0.162975746 | 0.014339167 |
| Arhgap8 | 0.127462697 | 0.014370637 |
| 4921524J17Rik | -0.085701808 | 0.014384111 |
| Tm6sf2 | 0.089969811 | 0.014404537 |
| Fis1 | -0.247285116 | 0.014432885 |
| Hist1h3g | 0.076907393 | 0.014461965 |
| Rheb | -0.157617069 | 0.014484377 |
| Ccnt1 | 0.161404131 | 0.014496396 |
| Fgfr1op | -0.116654496 | 0.014501898 |
| Cdkn2a | -0.082533195 | 0.014526396 |
| Rgma | 0.160619596 | 0.014529893 |
| H2-DMb2 | -0.254220509 | 0.014617095 |
| Pcdhb2 | -0.141084564 | 0.014620611 |
| Casc3 | 0.076901584 | 0.01464994 |
| D4Wsu53e | 0.267588183 | 0.014701766 |
| Loxl4 | 0.275796962 | 0.014733329 |
| Gimap9 | -0.150255243 | 0.014751855 |
| Lias | -0.173900362 | 0.014788569 |
| Nt5c3 | -0.283782768 | 0.014793169 |
| Ripply3 | -0.132749017 | 0.01483238 |
| Snd1 | 0.169487521 | 0.014883194 |
| Sun2 | 0.258800717 | 0.014886953 |
| 2410018L13Rik | -0.125110564 | 0.014891857 |
| Rasd1 | 0.165884583 | 0.014912664 |
| D6Wsu116e | 0.138726905 | 0.015007628 |
| Gemin7 | -0.141703703 | 0.015008589 |
| Pmpcb | -0.181094278 | 0.015016146 |
| Psmd14 | -0.1288564 | 0.015070414 |
| Extl3 | 0.072178107 | 0.015076156 |
| H2-Q6 | -0.274203511 | 0.015080195 |
| Gm12250 | -0.669933553 | 0.015083789 |
| Sprr1b | 0.12937874 | 0.015096635 |
| Szt2 | 0.157232154 | 0.015096727 |
| Mlxipl | 0.082841237 | 0.015104829 |
| Atp5l | -0.282895664 | 0.01510628 |
| Psma2 | -0.109650279 | 0.01512872 |
| Mrpl42 | -0.221421686 | 0.015135699 |
| Gpaa1 | 0.122456288 | 0.015143115 |
| 2410002F23Rik | -0.157826406 | 0.015178966 |
| 1810030N24Rik | -0.194637652 | 0.015189584 |
| Gkap1 | -0.126450219 | 0.01519034 |
| Vav2 | 0.121697216 | 0.015238535 |
| Atp5l | -0.271283468 | 0.015246354 |
| Atp2c2 | 0.139493903 | 0.015249399 |
| Hsbp1 | -0.090929194 | 0.015253531 |
| Acvr1b | 0.198545138 | 0.015266826 |
| 8430427H17Rik | 0.136098417 | 0.015284433 |
| Hist1h3a | 0.078757647 | 0.015318474 |
| Stard3nl | -0.268368839 | 0.015363337 |
| Pan2 | 0.126278682 | 0.015428146 |
| Usmg5 | -0.273889025 | 0.015456615 |
| Plxnc1 | 0.183399311 | 0.015456834 |
| Mylk2 | 0.100924411 | 0.015476945 |
| Ryr1 | 0.144713047 | 0.015531911 |
| Sptlc1 | -0.103551175 | 0.015543487 |
| Rasgrp4 | 0.144748002 | 0.015560838 |
| Uqcrb | -0.073567 | 0.015594729 |
| Hist1h3d | 0.078841159 | 0.015597967 |
| Ndufb7 | -0.216079591 | 0.01560739 |
| Ptgr2 | -0.17890129 | 0.015615079 |
| Ipo13 | 0.183822493 | 0.015626377 |
| C330018D20Rik | -0.230898208 | 0.015635607 |
| Arcn1 | -0.069816271 | 0.015648802 |
| Prkra | -0.137262601 | 0.015688692 |
| Srsf3 | -0.397553468 | 0.015688931 |
| Zfat | 0.109597214 | 0.015695443 |
| Atf7 | 0.218700495 | 0.015716567 |
| Slc6a14 | -0.270420041 | 0.015726 |
| Pcmt1 | -0.171387617 | 0.015726502 |
| Cox7b | -0.081451309 | 0.015742752 |
| Mlh1 | -0.21694343 | 0.015758657 |
| Ece1 | 0.167911914 | 0.015773275 |
| Ndufc1 | -0.115593985 | 0.01579462 |
| Cox5b | -0.14597915 | 0.015807189 |
| Ubap2 | 0.190751997 | 0.015811207 |
| Nudt19 | -0.233980551 | 0.015893617 |
| Sqrdl | -0.208603502 | 0.015968247 |
| Snx11 | 0.097701456 | 0.015988382 |
| Rabepk | -0.153865153 | 0.01599393 |
| Ceacam10 | -0.336926421 | 0.016037179 |
| Rfx1 | 0.129384421 | 0.016040478 |
| Synpo2 | 0.097198971 | 0.01604393 |
| Dos | 0.08964108 | 0.016066012 |
| Zfp189 | 0.088080787 | 0.016096341 |
| Mamstr | 0.067292583 | 0.016230733 |
| Pppde2 | 0.128785386 | 0.016246575 |
| Hmgb1 | -0.091738318 | 0.016282316 |
| Szt2 | 0.167371929 | 0.016285367 |
| Epas1 | 0.173990994 | 0.016287502 |
| Ptpmt1 | -0.261259339 | 0.0162997 |
| Ormdl1 | -0.336565616 | 0.016304268 |
| Tmem102 | 0.09157677 | 0.016322316 |
| Chrnb2 | 0.109964199 | 0.016361924 |
| Cdc23 | -0.532105632 | 0.016374391 |
| Rlf | 0.147875547 | 0.016376933 |
| Vti1b | -0.108351012 | 0.016384539 |
| 1700052N19Rik | -0.151712543 | 0.016390784 |
| Il17b | 0.102770225 | 0.01639094 |
| 1700029F09Rik | -0.207548151 | 0.016422574 |
| Inppl1 | 0.097109679 | 0.016452741 |
| Gfra2 | 0.103802767 | 0.016460639 |
| Hmbs | -0.228258447 | 0.016461909 |
| 0610009D07Rik | -0.27768778 | 0.016465896 |
| Lsm12 | 0.080551616 | 0.016477036 |
| Ifitm5 | 0.115138838 | 0.016505653 |
| Cnot1 | 0.119186845 | 0.016508717 |
| Pex3 | -0.188610323 | 0.016535145 |
| Mob4 | -0.164213474 | 0.016559605 |
| Odf2 | 0.116774402 | 0.016569408 |
| Mrpl27 | -0.202514845 | 0.016599634 |
| Epb4.1 | 0.206325599 | 0.016665563 |
| Pck1 | 0.279938498 | 0.016674249 |
| Adamtsl3 | 0.288322986 | 0.016734587 |
| Kdm4a | 0.116242996 | 0.016793583 |
| Tipin | -0.354528765 | 0.016807799 |
| Kcnh3 | 0.069580804 | 0.016837587 |
| Myo1d | 0.205619878 | 0.016872522 |
| Tnfrsf1b | 0.273430123 | 0.016877417 |
| Dock6 | 0.159522818 | 0.01694167 |
| 9530053A07Rik | 0.226611286 | 0.016978453 |
| Vps29 | -0.199031612 | 0.016986901 |
| Kbtbd11 | 0.11311916 | 0.016988043 |
| Ei24 | -0.169206244 | 0.017032425 |
| Atp5l | -0.25990027 | 0.017065381 |
| C1d | -0.179577697 | 0.017130386 |
| Abcc1 | 0.21247955 | 0.017184389 |
| Trp53inp1 | 0.213158001 | 0.017184743 |
| Zfp930 | -0.185453906 | 0.017198131 |
| 2400001E08Rik | -0.216654484 | 0.017231959 |
| Cacybp | -0.112045292 | 0.017238676 |
| Zfp455 | -0.308398685 | 0.017238841 |
| Grina | 0.149617078 | 0.017247393 |
| Zfp938 | -0.176584268 | 0.017258486 |
| Hist2h3c1 | 0.07987873 | 0.017287134 |
| Chdh | 0.205015729 | 0.017298562 |
| 2810047C21Rik1 | -0.26430301 | 0.017310063 |
| Rab11fip5 | 0.273303543 | 0.017313523 |
| Acsbg1 | 0.080390897 | 0.017341896 |
| Bub1b | 0.183889295 | 0.017343972 |
| H2-D1 | -0.116354536 | 0.017357144 |
| Rab12 | -0.148492294 | 0.017385078 |
| Tbc1d22a | 0.179512589 | 0.017405759 |
| Eftud2 | 0.087948712 | 0.017436332 |
| Pdcd2 | -0.10764489 | 0.01743863 |
| Itgb3bp | -0.125399291 | 0.01745728 |
| Ipmk | 0.147913922 | 0.017483709 |
| Ndufaf3 | -0.176158995 | 0.017500852 |
| Ube2l6 | -0.408097243 | 0.017537483 |
| Kdm6b | 0.186314444 | 0.017550623 |
| Psap | 0.083670629 | 0.01756423 |
| Gpr30 | 0.095346032 | 0.01761857 |
| Fam3c | 0.16874211 | 0.017627191 |
| Arhgef40 | 0.17695882 | 0.017636231 |
| Ublcp1 | -0.178218497 | 0.017637071 |
| Rpl5 | -0.11485783 | 0.017656826 |
| Vax2 | 0.099080967 | 0.017678266 |
| C2cd4b | 0.084459466 | 0.017696204 |
| Abt1 | -0.096726539 | 0.017698811 |
| Ndufa7 | -0.222338509 | 0.017743832 |
| Pmaip1 | 0.105197154 | 0.017770862 |
| Foxq1 | 0.175083911 | 0.01779233 |
| Mink1 | 0.131534638 | 0.017799129 |
| Naip6 | -0.156452919 | 0.017802926 |
| Trappc2 | -0.14401983 | 0.017817216 |
| Atg4a | -0.112507952 | 0.017824766 |
| 1110059E24Rik | -0.163293879 | 0.017826795 |
| Gm10088 | -0.087986999 | 0.017834117 |
| Megf6 | 0.173547421 | 0.017854801 |
| Igf2as | 0.084203401 | 0.017898562 |
| Nup188 | 0.119213712 | 0.017929836 |
| Nfyb | -0.125364025 | 0.01796158 |
| Cbx2 | 0.103671058 | 0.017982313 |
| AI314976 | -0.165187132 | 0.017999343 |
| Brox | -0.207388556 | 0.018114512 |
| Hmgb1 | -0.082478807 | 0.018122069 |
| Sesn2 | 0.216412146 | 0.018128666 |
| Fancl | -0.149183322 | 0.018170535 |
| Gnai2 | 0.091319801 | 0.018177852 |
| Ubl3 | -0.124277159 | 0.018197266 |
| Arpc5l | -0.145892766 | 0.018200756 |
| Kif21b | 0.111536123 | 0.01823634 |
| Nck1 | -0.159583084 | 0.018269732 |
| Pcdhb9 | -0.126029351 | 0.018285525 |
| E430025E21Rik | 0.181199694 | 0.018290178 |
| Vta1 | -0.184171552 | 0.018295672 |
| Crk | -0.07680492 | 0.018329709 |
| Ang6 | 0.078558164 | 0.018369986 |
| Taf9 | -0.205962344 | 0.01839286 |
| Ubxn8 | -0.137776353 | 0.018415866 |
| Clns1a | -0.221076659 | 0.018453034 |
| Mef2a | 0.121886538 | 0.01848847 |
| Krtap5-2 | 0.090282364 | 0.018500836 |
| Akna | 0.159606969 | 0.018532905 |
| Deb1 | -0.195594955 | 0.018546012 |
| Utp3 | -0.088908724 | 0.018552822 |
| Tgtp1 | -0.622066872 | 0.018553431 |
| Chmp2a | -0.166464946 | 0.018577966 |
| Ndufa11 | -0.178009076 | 0.018584582 |
| Eif4ebp2 | 0.148297747 | 0.01858857 |
| Trp53 | 0.116391676 | 0.018601662 |
| Rhou | 0.168183092 | 0.018614955 |
| Nipa1 | -0.180053902 | 0.018659888 |
| Cox6a2 | 0.18039947 | 0.018795091 |
| Hmgb1 | -0.082682264 | 0.01882441 |
| Gm6653 | 0.06928424 | 0.018844004 |
| Lcmt1 | -0.146430989 | 0.018863388 |
| Ccdc90b | -0.225110167 | 0.018863893 |
| Zmynd8 | 0.216372927 | 0.018954448 |
| Glo1 | -0.11307712 | 0.01902554 |
| Grhl3 | 0.08732054 | 0.019133544 |
| Tfb2m | -0.068595829 | 0.019148583 |
| Szt2 | 0.219152184 | 0.019226676 |
| Pdcd10 | -0.365561361 | 0.019233115 |
| E330020D12Rik | 0.102822615 | 0.019271957 |
| Gfpt1 | 0.113066814 | 0.019324735 |
| Prelp | 0.513056838 | 0.019353784 |
| Plekhm2 | 0.13804749 | 0.01935998 |
| Tgfbr1 | 0.123853608 | 0.019368688 |
| Chmp1b | -0.144806537 | 0.019381675 |
| Mir448 | 0.062764917 | 0.019407722 |
| Zfp873 | -0.211633289 | 0.019420232 |
| Nudt1 | -0.150739047 | 0.01942447 |
| Hmgb1 | -0.084946258 | 0.019433642 |
| Ccdc104 | -0.125623818 | 0.019433905 |
| Zcchc8 | -0.099784927 | 0.019434715 |
| Gm10158 | 0.211991293 | 0.019440139 |
| --- | 0.088953129 | 0.019503391 |
| --- | -0.132328252 | 0.019508408 |
| Tusc3 | -0.123618229 | 0.019522161 |
| Atg7 | 0.103701123 | 0.01952861 |
| Pdcl3 | -0.075548978 | 0.01956826 |
| Rangap1 | 0.148175558 | 0.019574935 |
| Anxa11 | 0.174302307 | 0.019622204 |
| Eif1ax | -0.106196614 | 0.01963531 |
| Prdm1 | 0.077787101 | 0.019636053 |
| Rpl5 | -0.134233418 | 0.01964171 |
| Zmiz1 | 0.180991646 | 0.019656098 |
| AB010352 | -0.220249175 | 0.019669819 |
| Grhl2 | 0.208257406 | 0.019669869 |
| Ctso | -0.19219018 | 0.019677091 |
| Cbx7 | 0.151096799 | 0.019704693 |
| Ppa1 | -0.121733719 | 0.019724518 |
| Ralbp2 | -0.147267812 | 0.019738617 |
| Zcchc10 | -0.141967565 | 0.019762192 |
| Dmkn | 0.071181987 | 0.019767804 |
| Tiprl | -0.149240506 | 0.019775103 |
| Fbp1 | -0.244274283 | 0.019775412 |
| Tox2 | 0.254801937 | 0.019781199 |
| Adra2c | -0.223547071 | 0.019782654 |
| Edn2 | 0.085851906 | 0.019791992 |
| Dhps | -0.193704604 | 0.019792323 |
| Atp5l | -0.266489254 | 0.019803244 |
| Esrp2 | 0.067133575 | 0.019806921 |
| Trim10 | 0.078027841 | 0.019814872 |
| Mfge8 | 0.176199791 | 0.019820036 |
| Kctd17 | 0.331259191 | 0.019850909 |
| Clk2 | 0.083106874 | 0.019853533 |
| Zfp748 | -0.19184519 | 0.019877137 |
| Ubxn6 | -0.191685023 | 0.019940193 |
| Irgm2 | -0.435907307 | 0.01998357 |
| Gpsm3 | 0.080028066 | 0.02000988 |
| Mafa | 0.088475698 | 0.020147574 |
| Sec24c | 0.131332151 | 0.020158755 |
| Zranb2 | -0.144234788 | 0.020162786 |
| Cnksr3 | 0.099392927 | 0.020187652 |
| Psmb7 | -0.09665335 | 0.020188835 |
| Hmgb1 | -0.078625444 | 0.020197466 |
| Inpp5d | 0.135487744 | 0.020207919 |
| Pcp4l1 | 0.063709808 | 0.020209837 |
| 8430410K20Rik | -0.145185052 | 0.020233799 |
| Ppp1r35 | -0.200894415 | 0.02024173 |
| Rxra | 0.136851743 | 0.020250618 |
| 2410002O22Rik | -0.080460003 | 0.020266962 |
| Gm5779 | -0.283840955 | 0.020268867 |
| H2-Q2 | -0.25876217 | 0.020297467 |
| Loxl3 | 0.464882792 | 0.02032917 |
| Mmp24 | -0.137292778 | 0.020331788 |
| Sec61g | -0.160634954 | 0.020335154 |
| Mgat5 | 0.241717151 | 0.020383579 |
| Zfp704 | 0.132547469 | 0.020413626 |
| Gtf2a2 | -0.20780045 | 0.020414461 |
| Evl | 0.207593704 | 0.020531599 |
| Cul5 | -0.079720249 | 0.020546907 |
| Mthfr | 0.2013069 | 0.020596687 |
| Ebag9 | -0.105207604 | 0.0205975 |
| Trp53i11 | 0.243871027 | 0.020657036 |
| Bms1 | 0.129380677 | 0.020665891 |
| Klk1b11 | -0.316065236 | 0.020731426 |
| Tnip1 | 0.064762874 | 0.020763698 |
| Cetn4 | -0.174548033 | 0.020768824 |
| Ralbp1 | 0.068480624 | 0.020772496 |
| Plin5 | 0.115461205 | 0.020781375 |
| Trim29 | 0.31454475 | 0.020795277 |
| Sparcl1 | 0.288051848 | 0.020804856 |
| Rpl5 | -0.104424505 | 0.02080637 |
| Gcsh | -0.287763681 | 0.020809801 |
| Grasp | 0.156421702 | 0.020813997 |
| Lpl | 0.411517321 | 0.020829372 |
| Mical2 | 0.191013044 | 0.020866308 |
| Atp2c1 | 0.088765948 | 0.020896433 |
| Gm16381 | -0.177191176 | 0.020899741 |
| Nup37 | -0.155582214 | 0.020901212 |
| Cyp2d22 | 0.273877384 | 0.020915006 |
| Zfp426 | -0.068252007 | 0.020974969 |
| Prkcd | 0.153936482 | 0.020984932 |
| Mrpl15 | -0.171139022 | 0.02099478 |
| Tmem192 | -0.17133761 | 0.020999235 |
| Oas1c | -0.216401621 | 0.021004332 |
| Lsm6 | -0.098750561 | 0.021017084 |
| Mrps28 | -0.112825378 | 0.021037187 |
| Zfp639 | -0.173211513 | 0.021077793 |
| Sumo2 | -0.066451519 | 0.021136978 |
| Gimap4 | -0.1509532 | 0.021160909 |
| Mtif3 | -0.118450896 | 0.021171581 |
| Eci1 | -0.083076111 | 0.021197822 |
| Arhgef2 | 0.16400516 | 0.021272826 |
| Fam160b2 | 0.093451709 | 0.021279549 |
| Grb7 | 0.242671815 | 0.021323168 |
| Rasl11b | 0.22010921 | 0.021349166 |
| Polr2h | -0.111948295 | 0.021394834 |
| Zfp772 | -0.261611669 | 0.021412253 |
| Tfb2m | -0.065977566 | 0.021429342 |
| Mir125a | 0.116087835 | 0.021443269 |
| Dennd1c | 0.11235368 | 0.021449303 |
| Gsta4 | -0.594726637 | 0.021450102 |
| Trmt5 | -0.100733397 | 0.021462126 |
| Lrrc14 | 0.104047757 | 0.021516881 |
| Cept1 | -0.05884808 | 0.021521876 |
| Epb4.1l2 | 0.154458427 | 0.0215535 |
| 2410002I01Rik | 0.082872472 | 0.021568853 |
| Ptger3 | 0.124303461 | 0.021572492 |
| Gm9558 | -0.107785288 | 0.021580551 |
| Wdr24 | -0.10854863 | 0.021602475 |
| Adcy1 | 0.090708497 | 0.021632633 |
| 1600012F09Rik | -0.193653229 | 0.021645712 |
| Gbp3 | -0.375969142 | 0.021661494 |
| Sp140 | -0.117619958 | 0.021706983 |
| Itga4 | 0.129450151 | 0.021708991 |
| Plagl2 | 0.112283033 | 0.021716993 |
| Rmnd5a | 0.153564124 | 0.021728346 |
| Rapgef3 | 0.083631001 | 0.021729251 |
| Chst12 | 0.153160781 | 0.021731522 |
| Gpc2 | 0.088505918 | 0.021740057 |
| Fam103a1 | -0.145182676 | 0.021743644 |
| 2410127L17Rik | -0.126803137 | 0.021773075 |
| Dimt1 | -0.197922486 | 0.021779958 |
| Pdpr | -0.100931424 | 0.021785569 |
| Gm4980 | 0.080767528 | 0.021786195 |
| Zfp157 | -0.088280421 | 0.021800218 |
| Zfp472 | -0.065973581 | 0.021832462 |
| C1rl | -0.189273866 | 0.021841637 |
| 2610044O15Rik | -0.196486211 | 0.021845721 |
| Szt2 | 0.176515333 | 0.021850085 |
| Brox | -0.100798126 | 0.021863053 |
| Clpp | -0.135829952 | 0.021903901 |
| Mafg | 0.099413177 | 0.021926884 |
| Tet3 | 0.175841861 | 0.021939725 |
| Gse1 | 0.147587646 | 0.021940861 |
| Reep4 | 0.22714551 | 0.021953903 |
| Plin4 | 0.303226869 | 0.021971354 |
| Men1 | 0.074090796 | 0.02200158 |
| Cd8b1 | -0.061370564 | 0.022008428 |
| Zfp68 | -0.167074376 | 0.022076736 |
| Nxt2 | -0.196955823 | 0.022095763 |
| Kif18b | 0.130968684 | 0.022097418 |
| Sumo3 | -0.135288398 | 0.0221153 |
| Rnf20 | 0.117513457 | 0.022189033 |
| Rhod | -0.171473853 | 0.022192903 |
| 4833420G17Rik | 0.089009283 | 0.022270094 |
| Ndufv2 | -0.113951871 | 0.022276773 |
| Grn | 0.097589387 | 0.022290943 |
| Pcdhb11 | -0.34128535 | 0.022297957 |
| Wdr70 | 0.128858122 | 0.022319674 |
| Zfp942 | -0.18059955 | 0.022331979 |
| Mrps27 | -0.185167697 | 0.022334867 |
| Ufc1 | -0.21462951 | 0.022334929 |
| Pnpla7 | 0.162801371 | 0.022360972 |
| Ndufc1 | -0.11942676 | 0.022370775 |
| H2-Q10 | -0.130215901 | 0.02238436 |
| Tmem41b | -0.216237073 | 0.022406344 |
| Rab11fip1 | 0.189790412 | 0.022407391 |
| Psma6 | -0.160956753 | 0.022426503 |
| Rwdd1 | -0.1232982 | 0.022449941 |
| Ccdc77 | 0.125581006 | 0.022469278 |
| Hnmt | -0.274503196 | 0.022479315 |
| Gm9958 | -0.231569858 | 0.022497351 |
| Sprr2a2 | 0.097567583 | 0.02250757 |
| Fam35a | -0.075121084 | 0.022518722 |
| H2-Q4 | -0.262280051 | 0.022521229 |
| Tmx3 | -0.123545773 | 0.02253832 |
| Rps11 | -0.140430498 | 0.022560078 |
| Gpc3 | 0.508819296 | 0.022584302 |
| Myh2 | 0.211917265 | 0.02262885 |
| 4930451I11Rik | 0.111903728 | 0.022669989 |
| Pnpla6 | 0.168041638 | 0.022700455 |
| Med31 | -0.148033627 | 0.022725421 |
| Phf11 | -0.189187709 | 0.022726427 |
| Mir697 | 0.083652094 | 0.022735442 |
| C330007P06Rik | -0.170083471 | 0.022783893 |
| Ahr | 0.162212607 | 0.022804008 |
| Cmc1 | -0.191093754 | 0.022811947 |
| Chit1 | 0.399057911 | 0.022850002 |
| Ict1 | -0.181087759 | 0.022865487 |
| Zfp280c | -0.078944817 | 0.022879851 |
| Tead4 | 0.230875525 | 0.022941675 |
| Ppa2 | -0.175321938 | 0.022977968 |
| Nav1 | 0.304233412 | 0.022991484 |
| Aebp1 | 0.547834286 | 0.023056444 |
| 3830406C13Rik | -0.124533288 | 0.023068367 |
| Thoc1 | -0.091943861 | 0.02311827 |
| Glce | -0.10615648 | 0.023131887 |
| Sumo2 | -0.07628269 | 0.023132848 |
| Tanc2 | 0.22000663 | 0.023156868 |
| Dalrd3 | -0.090433994 | 0.023186142 |
| Cox7b | -0.119624447 | 0.02323485 |
| Bst2 | -0.25267904 | 0.023252257 |
| Cdc5l | -0.0641779 | 0.023252458 |
| Cdc5l | -0.0641779 | 0.023252458 |
| Scrib | 0.197974206 | 0.023281558 |
| Abca1 | 0.18435821 | 0.023285657 |
| Sqstm1 | 0.084029866 | 0.023293678 |
| Gm7173 | -0.159486935 | 0.023317091 |
| Dock6 | 0.245124056 | 0.023327929 |
| Stk25 | -0.0974412 | 0.023330502 |
| Rnu1b1 | -0.344842813 | 0.023334161 |
| Tbc1d2 | 0.09555588 | 0.023346124 |
| Zfp58 | -0.192431891 | 0.023357312 |
| Glo1 | -0.117103313 | 0.023408859 |
| Elf4 | 0.139264853 | 0.023425098 |
| Zfp946 | -0.254008109 | 0.023451209 |
| Atp6v1h | -0.123903772 | 0.023463593 |
| Ankra2 | -0.061631539 | 0.02350731 |
| Rnf122 | -0.125616433 | 0.02350746 |
| Mslnl | 0.129722397 | 0.023509073 |
| Pcdhb5 | -0.233039982 | 0.023526614 |
| Prdx3 | -0.149001672 | 0.023549669 |
| Cfd | 0.418348514 | 0.023558096 |
| Ptrh2 | -0.159534673 | 0.023620983 |
| Rps27a | -0.234142885 | 0.0236241 |
| Cdc5l | -0.068360455 | 0.023630682 |
| Poglut1 | -0.104165539 | 0.023657178 |
| Rin2 | 0.133322977 | 0.023666115 |
| Sec61g | -0.145429759 | 0.023671791 |
| Lpcat3 | 0.119538023 | 0.023673179 |
| Taf11 | -0.086498124 | 0.023678909 |
| Rpain | -0.130858856 | 0.023689701 |
| Entpd4 | 0.092895429 | 0.023720091 |
| Szt2 | 0.181151903 | 0.023725105 |
| Chchd2 | -0.199765454 | 0.023730189 |
| Cdc5l | -0.071362498 | 0.023774723 |
| Hipk2 | 0.272834794 | 0.023782806 |
| A530054K11Rik | -0.063693212 | 0.023792179 |
| Ndufb3 | -0.135743702 | 0.023811521 |
| Mccc2 | -0.11130989 | 0.023836331 |
| Txnl1 | -0.167894919 | 0.023843023 |
| Mgmt | -0.138844744 | 0.023861571 |
| Taz | -0.110955926 | 0.023900689 |
| Loxl4 | 0.250891866 | 0.023904537 |
| Kcnk6 | 0.269373048 | 0.023916938 |
| Igsf5 | -0.127907864 | 0.023922577 |
| Cmklr1 | 0.105893912 | 0.023954779 |
| Ppp6c | -0.12901996 | 0.023960517 |
| Fam38b | 0.171237681 | 0.024148657 |
| Hmgb1 | -0.075601867 | 0.024164435 |
| Zfp952 | -0.136977787 | 0.024184135 |
| Nif3l1 | -0.161883879 | 0.024245893 |
| Tmed5 | -0.254465849 | 0.024247435 |
| St14 | 0.059712456 | 0.024281404 |
| Thyn1 | -0.219810126 | 0.024335553 |
| Gnptg | -0.140734881 | 0.024352846 |
| Cybrd1 | 0.301674369 | 0.024359627 |
| Anapc10 | -0.099429684 | 0.024360283 |
| AA467197 | -0.078679962 | 0.024405908 |
| Slco3a1 | 0.188783571 | 0.024415838 |
| Ckap2l | 0.104523242 | 0.024422543 |
| Mir326 | 0.094700323 | 0.024432171 |
| AI429214 | -0.112343852 | 0.02444976 |
| Syngap1 | 0.159753491 | 0.024462901 |
| Rad21 | 0.10722756 | 0.024468101 |
| Ppp1r3e | 0.10390543 | 0.024509655 |
| Kctd12 | 0.148959943 | 0.024547078 |
| Snrpc | -0.138479663 | 0.024566389 |
| BC031181 | -0.101002881 | 0.024590949 |
| 0610007C21Rik | -0.113611737 | 0.024611014 |
| Myst2 | 0.070265412 | 0.024624383 |
| Smpd1 | -0.131836889 | 0.024677433 |
| Gm12669 | -0.247873315 | 0.024690089 |
| Atp5e | -0.271859423 | 0.024734206 |
| Spata7 | -0.135196307 | 0.024768957 |
| 2610029I01Rik | -0.197750472 | 0.024776142 |
| Gnpda2 | -0.163482119 | 0.024810185 |
| Hsd17b7 | -0.132664042 | 0.024823775 |
| Zfp810 | -0.230438496 | 0.024838922 |
| Mmadhc | -0.130991806 | 0.024890579 |
| Rbp2 | 0.059975572 | 0.024915109 |
| Gm10033 | -0.149247115 | 0.024936225 |
| Spnb3 | 0.128574793 | 0.024980498 |
| Dnttip2 | -0.108297116 | 0.025021182 |
| Blvra | -0.157800161 | 0.025060121 |
| Tmem45b | 0.202200321 | 0.025075991 |
| Pcdhb20 | -0.179365773 | 0.025077934 |
| Sema3g | 0.06938292 | 0.02508131 |
| Tubgcp6 | 0.186491425 | 0.025146359 |
| Sgsm3 | 0.168684528 | 0.025186405 |
| Grik3 | 0.361264251 | 0.025187079 |
| Atg4a | -0.116675086 | 0.025196071 |
| Ncr1 | -0.15068623 | 0.025201162 |
| Trdn | 0.216741314 | 0.025231886 |
| Foxm1 | 0.167006347 | 0.025235756 |
| Sertad2 | 0.091320837 | 0.02524847 |
| Slc1a4 | 0.173249201 | 0.02526424 |
| Eid1 | -0.14961225 | 0.02526586 |
| Zfp738 | -0.07654394 | 0.025295168 |
| Uba3 | -0.119144122 | 0.025323207 |
| Nt5m | -0.121387069 | 0.025338828 |
| Ifi47 | -0.433441433 | 0.02540789 |
| Wtap | -0.153883729 | 0.025438424 |
| Tnks1bp1 | 0.135466769 | 0.025440395 |
| Abcd1 | 0.094179802 | 0.025483012 |
| Rpl24 | -0.091075795 | 0.025494614 |
| Rps15a-ps2 | -0.080768686 | 0.025500174 |
| Golga7 | 0.101514544 | 0.025514177 |
| Mrps36 | -0.227357805 | 0.025530397 |
| 2310067B10Rik | 0.135836854 | 0.02555651 |
| Dnajc30 | -0.105582216 | 0.025571075 |
| Celf1 | 0.090380094 | 0.025581751 |
| Mgat2 | -0.183001132 | 0.025592031 |
| Actr6 | -0.164581163 | 0.025594498 |
| Smn1 | -0.146518774 | 0.025608879 |
| Scarna17 | 0.116007841 | 0.02565921 |
| Scarna17 | 0.116007841 | 0.02565921 |
| 2810047C21Rik1 | -0.199777126 | 0.025683657 |
| Fahd2a | -0.077326062 | 0.025694357 |
| Dlc1 | 0.200222485 | 0.0257366 |
| 4930442E04Rik | 0.070188782 | 0.025808365 |
| Bnip2 | -0.11826783 | 0.0258192 |
| Dock6 | 0.200621337 | 0.025840975 |
| Tctex1d2 | -0.128897186 | 0.025865681 |
| Tcp11 | 0.087280654 | 0.025872997 |
| Mettl3 | 0.090193555 | 0.025876335 |
| Lsm6 | -0.079241041 | 0.025927482 |
| Zfp808 | -0.188631529 | 0.025985247 |
| Med6 | -0.181329146 | 0.02599909 |
| Rassf10 | 0.108295434 | 0.026008387 |
| Uxt | -0.158179089 | 0.02601749 |
| Zdhhc18 | 0.169776753 | 0.026078074 |
| Il17ra | 0.111581176 | 0.026092854 |
| Pik3r2 | 0.085870799 | 0.026126768 |
| D130043K22Rik | 0.071545189 | 0.026221953 |
| Zfp51 | -0.175209981 | 0.02622523 |
| Fhit | -0.191224689 | 0.026314378 |
| Bcap29 | -0.243115198 | 0.026356209 |
| D16H22S680E | -0.146796772 | 0.026360977 |
| Dlg5 | 0.138499781 | 0.026386962 |
| Gm7444 | -0.073596722 | 0.026392378 |
| Ensa | -0.108810597 | 0.026392581 |
| Sema6a | 0.29345547 | 0.026441886 |
| Col4a2 | 0.142324115 | 0.026473046 |
| Slc7a8 | 0.161066343 | 0.026477982 |
| Sec61g | -0.156434213 | 0.026495098 |
| Sin3b | 0.079863816 | 0.026538648 |
| Lmx1a | 0.108739299 | 0.026543595 |
| Atp6v1g2 | 0.064041477 | 0.026618794 |
| Mmp11 | 0.158247122 | 0.026623209 |
| Arhgef38 | 0.147954243 | 0.026633837 |
| Dnalc1 | -0.129804503 | 0.026651824 |
| Gm9897 | -0.066990521 | 0.026655122 |
| Ngrn | -0.182554081 | 0.026671893 |
| Mrpl48 | -0.162349497 | 0.026711025 |
| Cnnm3 | 0.110235111 | 0.026713071 |
| Ildr2 | 0.070957178 | 0.026737182 |
| Dhrs3 | 0.175925266 | 0.026758978 |
| Polr3a | 0.09967097 | 0.02677984 |
| Slc38a7 | 0.100423414 | 0.026830561 |
| Mettl17 | 0.060799698 | 0.026852068 |
| Exog | -0.102366419 | 0.026955705 |
| Gnpnat1 | -0.184857713 | 0.026999325 |
| Casq1 | 0.143666179 | 0.026999427 |
| Lrpap1 | -0.111451583 | 0.027004385 |
| Cd209a | 0.279933822 | 0.02700501 |
| Hist1h2ao | 0.072700436 | 0.027035528 |
| Nanp | -0.169131797 | 0.027058193 |
| Ski | 0.169972504 | 0.027095767 |
| Zfp750 | 0.16669086 | 0.02712064 |
| Skp1a | -0.102346019 | 0.027175412 |
| Cerk | 0.123765466 | 0.027184712 |
| Hnrnpc | -0.181383092 | 0.02719962 |
| Mrps21 | -0.08840893 | 0.027202268 |
| 6330416G13Rik | 0.209962569 | 0.027251411 |
| Slc35f5 | -0.116305633 | 0.027252764 |
| Hagh | -0.121334117 | 0.027262746 |
| Rrp8 | -0.115948867 | 0.027328437 |
| Fnbp1 | 0.170976101 | 0.027359158 |
| Gm16386 | -0.095549349 | 0.027391112 |
| Poc1b | 0.091456327 | 0.027393766 |
| Lta | -0.109946711 | 0.02739677 |
| Odf2l | -0.088398528 | 0.027451618 |
| Mrps36 | -0.255035851 | 0.027454529 |
| 1110037F02Rik | 0.134653153 | 0.02747142 |
| Timm17a | -0.100643631 | 0.027478154 |
| Spop | 0.116625001 | 0.027490423 |
| Sdhc | -0.11595715 | 0.027503495 |
| Hist1h2ao | 0.071028686 | 0.027513895 |
| BC003331 | -0.142766093 | 0.027518873 |
| Pnck | -0.23666298 | 0.027528063 |
| Fundc1 | -0.105957894 | 0.027529782 |
| Tekt2 | 0.056484644 | 0.027531752 |
| Xpc | 0.109062799 | 0.027603098 |
| Pafah1b2 | -0.144721964 | 0.027619962 |
| Solh | 0.129640811 | 0.027650093 |
| C530028O21Rik | 0.144193454 | 0.027657721 |
| Casp9 | 0.09494278 | 0.027680305 |
| Cat | 0.11772281 | 0.027681579 |
| 9930014A18Rik | -0.249910721 | 0.027742816 |
| Tomm40l | -0.214899339 | 0.027748973 |
| 9430038I01Rik | -0.135330829 | 0.027751729 |
| Irf7 | -0.399729013 | 0.027754292 |
| Kank2 | 0.133547563 | 0.027756362 |
| Chchd2 | -0.195511489 | 0.027783508 |
| Commd8 | -0.118782581 | 0.027803412 |
| Cspp1 | 0.09600986 | 0.027811372 |
| Psmd13 | -0.130385305 | 0.02782416 |
| Tmem5 | -0.185347053 | 0.027854975 |
| Hist1h2ao | 0.068426437 | 0.027866096 |
| Fam129a | 0.423319569 | 0.027866305 |
| Hist1h2ah | 0.070961598 | 0.027895682 |
| Efnb1 | 0.179130761 | 0.027901434 |
| 2400001E08Rik | -0.149621033 | 0.027901842 |
| Oaz1 | -0.167255163 | 0.027940944 |
| Bzw1 | -0.108291903 | 0.028009249 |
| Calhm2 | 0.13827446 | 0.028010662 |
| Fh1 | -0.1193367 | 0.028014181 |
| Raet1d | -0.09297754 | 0.028017078 |
| Uqcrfs1 | -0.150614136 | 0.028030749 |
| Timp3 | 0.131381913 | 0.028042736 |
| Bbs4 | -0.221163791 | 0.028043844 |
| Eif4e2 | -0.08481516 | 0.028045461 |
| Mterf | -0.075159614 | 0.028079727 |
| Haus1 | -0.176716861 | 0.028086607 |
| Aph1b | -0.106994856 | 0.028121376 |
| Tln1 | 0.156412084 | 0.028142331 |
| Gm9776 | -0.086361667 | 0.028152345 |
| Pgam2 | 0.162238162 | 0.028200846 |
| Hmgb1 | -0.070267251 | 0.028227834 |
| Naga | 0.094758194 | 0.028239855 |
| Tcea1 | -0.145922863 | 0.028250801 |
| Rfng | 0.088535593 | 0.028252606 |
| Gm4951 | -0.585349766 | 0.028284933 |
| Rcor1 | 0.174797016 | 0.028314516 |
| Pcdhb6 | -0.32281838 | 0.028314688 |
| Rps3a | -0.161672582 | 0.028315516 |
| Rhob | -0.132193025 | 0.028339716 |
| Pvrl4 | 0.127173665 | 0.028340275 |
| Uqcc | -0.137526049 | 0.028345483 |
| Adamts9 | 0.170520184 | 0.028360017 |
| Wasf2 | 0.162592342 | 0.028379142 |
| Cdk2 | -0.079655408 | 0.028386113 |
| Szt2 | 0.139495466 | 0.02840837 |
| Slc38a8 | 0.061030205 | 0.028411025 |
| Nagpa | 0.068469551 | 0.028530764 |
| Cd5l | 0.256588922 | 0.028535443 |
| Fam103a1 | -0.127081364 | 0.028542519 |
| Rfwd2 | -0.086619898 | 0.028583111 |
| Zfp935 | -0.193319668 | 0.028616468 |
| Hs6st3 | 0.061638407 | 0.028630887 |
| Myh9 | 0.259756629 | 0.028631894 |
| 2610528E23Rik | -0.164965384 | 0.028638866 |
| Clk1 | 0.103289038 | 0.028658782 |
| Igf2 | 0.072855062 | 0.028679423 |
| Rps27a | -0.169758908 | 0.028679459 |
| Card14 | 0.109603122 | 0.028693467 |
| Mir140 | 0.269484812 | 0.02873129 |
| Atmin | 0.105811641 | 0.028750812 |
| Tmem63a | 0.105880219 | 0.028783317 |
| Mir673 | 0.05880984 | 0.028809908 |
| Mup20 | -0.077790443 | 0.028865239 |
| Ccdc48 | 0.14830925 | 0.028883516 |
| Zfand1 | -0.194030795 | 0.028896282 |
| Rps27l | -0.283526379 | 0.028913359 |
| Dgka | 0.126695794 | 0.028942262 |
| Ddx51 | -0.071284161 | 0.028946849 |
| Taok3 | 0.125106167 | 0.02897674 |
| Olfr517 | 0.089990851 | 0.028983733 |
| Sar1b | -0.183993676 | 0.028991354 |
| Plxdc2 | 0.156578829 | 0.02899178 |
| Tbp | -0.133585307 | 0.029007004 |
| Stx18 | -0.13424927 | 0.029009284 |
| Ccdc94 | -0.106264866 | 0.029037825 |
| Vaultrc5 | -0.382701111 | 0.029041169 |
| Nono | 0.063373539 | 0.02905636 |
| Col4a1 | 0.166399104 | 0.029069503 |
| Denr | -0.145571762 | 0.029072887 |
| Hist1h2af | 0.070725203 | 0.029103775 |
| Sncb | 0.083854012 | 0.02911707 |
| Mxra8 | 0.363654884 | 0.029154953 |
| Cdc16 | -0.150531454 | 0.029157366 |
| Mrps17 | -0.195689224 | 0.029168738 |
| Rtcd1 | -0.155298589 | 0.02922898 |
| Smap2 | 0.096128362 | 0.029232876 |
| Nip7 | -0.200715304 | 0.029305917 |
| Cdh1 | 0.106757502 | 0.029317244 |
| Klhl9 | -0.158906391 | 0.02934149 |
| Ndufa11 | -0.12887154 | 0.029352859 |
| Dysf | 0.121591119 | 0.029395674 |
| Tk2 | -0.085612362 | 0.029415986 |
| AI462493 | -0.166586877 | 0.029443713 |
| Pla2g10 | 0.056749194 | 0.029464694 |
| 4930596D02Rik | 0.058305605 | 0.029470036 |
| Ccdc25 | -0.111552114 | 0.029494607 |
| Cenpv | -0.18576991 | 0.029510451 |
| Hnrnpa2b1 | 0.13777582 | 0.029534121 |
| Fdxr | 0.091034266 | 0.029538625 |
| Swi5 | -0.199996236 | 0.029539823 |
| Ccdc72 | -0.135866874 | 0.029550296 |
| Pdcd6 | -0.203235804 | 0.029572994 |
| Gm5037 | -0.059988033 | 0.02958741 |
| Rnu1b1 | -0.468413763 | 0.029615607 |
| Pde2a | 0.14484054 | 0.02967791 |
| Sap130 | 0.112019521 | 0.029678468 |
| Ankrd2 | 0.094680301 | 0.029758614 |
| Foxf1 | -0.094754064 | 0.029772935 |
| St7 | -0.098984788 | 0.029853419 |
| Nqo2 | -0.11061302 | 0.029864007 |
| Dock6 | 0.142529844 | 0.02988982 |
| Heatr5b | 0.102532094 | 0.029915018 |
| Pdk3 | 0.197795572 | 0.030003245 |
| Mrpl16 | -0.164949325 | 0.030006517 |
| Hscb | -0.178622907 | 0.030030865 |
| Smad2 | -0.129931071 | 0.030061222 |
| Sardh | 0.175737147 | 0.030061255 |
| Zfand1 | -0.182731071 | 0.030113346 |
| Gm4841 | -0.212704826 | 0.030116098 |
| Gxylt2 | 0.126245529 | 0.030118146 |
| Zc3h12c | -0.134595247 | 0.030155792 |
| Snx29 | 0.131682341 | 0.030196539 |
| Lipt1 | -0.106439459 | 0.030219561 |
| Usp48 | 0.223839762 | 0.030227474 |
| Slc22a4 | 0.271031821 | 0.030231457 |
| Tnpo3 | 0.088832363 | 0.030274508 |
| Itgb1bp1 | -0.143307545 | 0.030311754 |
| Atp6v0a4 | 0.128210978 | 0.030333666 |
| Arfip1 | -0.081821169 | 0.030346542 |
| Orc4 | -0.137221302 | 0.030360509 |
| Atpaf2 | -0.084011632 | 0.030419029 |
| Tmem30a | -0.124650251 | 0.030446292 |
| Hmcn1 | 0.13482155 | 0.03044643 |
| Copg | 0.123534497 | 0.030465291 |
| Mug-ps1 | 0.216902297 | 0.030481347 |
| Spen | 0.24867294 | 0.030490405 |
| Klhl31 | 0.092369261 | 0.030516753 |
| Lactb2 | -0.142750355 | 0.03055647 |
| Tmbim6 | 0.068282639 | 0.030589281 |
| Dpyd | -0.262537898 | 0.030604227 |
| Snx29 | 0.109063544 | 0.030613187 |
| Il28ra | 0.175046178 | 0.030650871 |
| Wdr34 | -0.203789502 | 0.030721663 |
| 5031439G07Rik | 0.121959362 | 0.030781103 |
| Tnrc6c | 0.151751032 | 0.030858846 |
| Exosc9 | -0.079695317 | 0.030879301 |
| Ltbp4 | 0.177990991 | 0.030892709 |
| Ube4b | 0.254327665 | 0.030921134 |
| Sec61g | -0.143046596 | 0.030961473 |
| Rpl35a | -0.104280652 | 0.030973704 |
| Gm4924 | -0.173603519 | 0.030974707 |
| Als2cl | 0.10864107 | 0.03102367 |
| 1500002C15Rik | 0.07921274 | 0.031036293 |
| Herc3 | -0.081461323 | 0.031036509 |
| Zfp568 | 0.111834556 | 0.031046702 |
| BC021785 | 0.079876724 | 0.031055406 |
| C80913 | -0.14000824 | 0.031057065 |
| Evpl | 0.082875172 | 0.031080953 |
| Gm8098 | 0.090707372 | 0.031086983 |
| Commd3 | -0.118475893 | 0.031089496 |
| Rpl5 | -0.094969362 | 0.031112789 |
| Idh3a | -0.155084297 | 0.031121547 |
| Mrpl41 | -0.224188386 | 0.031213297 |
| Reep6 | 0.174686806 | 0.031235124 |
| Pvrl3 | -0.128426418 | 0.031250958 |
| Snrpa1 | -0.159520178 | 0.031257064 |
| Rhbdf2 | 0.158318006 | 0.031271019 |
| Klrb1f | -0.126962155 | 0.031276688 |
| Oaz1 | -0.167920321 | 0.031286703 |
| Wipi1 | 0.176815441 | 0.031287333 |
| Gpr161 | 0.127326369 | 0.031291212 |
| Skiv2l2 | -0.103336133 | 0.031312511 |
| Banp | 0.13961979 | 0.031318497 |
| Dusp13 | 0.093430368 | 0.031447978 |
| Ahnak2 | 0.269971635 | 0.03150866 |
| Slc45a4 | 0.154791155 | 0.031514024 |
| Rassf3 | -0.104758151 | 0.031572441 |
| Col5a3 | 0.095600073 | 0.031589403 |
| Icam1 | 0.11783525 | 0.031593765 |
| Gm9766 | 0.38846761 | 0.031617891 |
| Mterfd3 | -0.077009357 | 0.031625363 |
| Gtf3a | -0.191324334 | 0.031627934 |
| Ndufs4 | -0.193899683 | 0.031631302 |
| Asap1 | 0.281983303 | 0.031632296 |
| Lyrm4 | -0.165222603 | 0.031668219 |
| Pcdhb3 | -0.249710781 | 0.031673479 |
| Dnajb6 | -0.090626637 | 0.031679059 |
| Hist1h2ao | 0.068973755 | 0.031701718 |
| Stk11 | 0.093495077 | 0.03173606 |
| Cntnap1 | 0.159362157 | 0.031737404 |
| 1810063B05Rik | -0.131190225 | 0.031780237 |
| Acot13 | -0.169735011 | 0.031785154 |
| Kdm5c | 0.116378757 | 0.031808799 |
| Fam120a | 0.115430613 | 0.03182735 |
| Olfr279 | 0.177955665 | 0.031845667 |
| A830018L16Rik | -0.198802747 | 0.031853784 |
| H2-DMa | -0.143465121 | 0.031856491 |
| Riok1 | -0.066639773 | 0.031873089 |
| Cct6a | -0.139316398 | 0.031890188 |
| Fggy | -0.119369493 | 0.031937054 |
| 1110002N22Rik | -0.235479925 | 0.031969151 |
| Polr2a | 0.142069419 | 0.031990326 |
| Ndufb6 | -0.186923059 | 0.031998302 |
| Rpp21 | -0.125866459 | 0.032003694 |
| Vkorc1l1 | -0.066422061 | 0.032101442 |
| Gm11711 | 0.168867133 | 0.032116476 |
| Chst11 | 0.247685216 | 0.032127369 |
| Kifc2 | 0.071538849 | 0.032142633 |
| Neurl1a | 0.096184693 | 0.032145376 |
| Aoc2 | 0.06502743 | 0.032171554 |
| Syngr2 | 0.063789567 | 0.032212997 |
| Fam59b | 0.077348467 | 0.032241656 |
| Iqcb1 | -0.108343562 | 0.03225043 |
| Rpl10l | -0.107119256 | 0.032259251 |
| Nat2 | -0.160563934 | 0.032290701 |
| Hbb-y | -0.07748693 | 0.032291421 |
| Prdx6b | 0.06032724 | 0.032302745 |
| Hmgb3 | -0.156372436 | 0.032318396 |
| E130311K13Rik | -0.147710964 | 0.032339145 |
| 4933411K16Rik | 0.063596404 | 0.032391777 |
| Pacs2 | 0.096145087 | 0.032410191 |
| Pgpep1 | 0.075271261 | 0.032428863 |
| B230216G23Rik | 0.068852936 | 0.032471184 |
| Extl1 | 0.260090263 | 0.032493287 |
| Lrrc26 | -0.321866694 | 0.03249408 |
| ATP6 | -0.222834826 | 0.032496201 |
| Slc35b3 | -0.146352663 | 0.032506795 |
| Eml3 | 0.068143464 | 0.03251761 |
| Phldb2 | 0.139123777 | 0.032543834 |
| Cit | 0.136465623 | 0.032549851 |
| Nln | -0.116296306 | 0.032579262 |
| Ndufb2 | -0.191785755 | 0.032584828 |
| Nap1l3 | -0.119252499 | 0.032603469 |
| Arf3 | 0.176909131 | 0.032621082 |
| Pigp | -0.144827119 | 0.032652633 |
| Mitd1 | -0.20952556 | 0.032659733 |
| Pcdhb15 | -0.278091456 | 0.032669794 |
| Kptn | -0.137236571 | 0.032679241 |
| Wrb | -0.191153071 | 0.032708478 |
| Synj2bp | -0.057993534 | 0.032771667 |
| Dnajb6 | -0.089274768 | 0.032784444 |
| AI987944 | -0.251942438 | 0.032802275 |
| Hey2 | -0.32093037 | 0.032803428 |
| Paip2 | -0.112081539 | 0.032820442 |
| Nae1 | -0.194991108 | 0.03282713 |
| Ttn | 0.507066101 | 0.032839747 |
| Psg20 | 0.070176298 | 0.03287719 |
| Slc35a5 | -0.073743036 | 0.032880092 |
| Atp5f1 | -0.072105222 | 0.032971715 |
| 1700012P22Rik | -0.067029871 | 0.033006021 |
| Vps13d | 0.233572996 | 0.033027597 |
| Gpd1 | 0.234913621 | 0.033047629 |
| Slc12a7 | 0.136223366 | 0.033070414 |
| Fbxo16 | -0.068481063 | 0.033071019 |
| Diap3 | 0.125387018 | 0.033112308 |
| 2900010M23Rik | -0.195429674 | 0.033134551 |
| Prss33 | 0.083200501 | 0.033136161 |
| Fgfr1 | 0.259237701 | 0.033140427 |
| B830017H08Rik | 0.092836778 | 0.033147431 |
| Fam133b | -0.112364595 | 0.033181706 |
| Gns | 0.112613851 | 0.03320005 |
| 1600012F09Rik | -0.159868712 | 0.03321924 |
| 1600012F09Rik | -0.159868712 | 0.03321924 |
| Arhgap23 | 0.074872015 | 0.033222728 |
| Gm8994 | -0.066297896 | 0.033308422 |
| Etfa | -0.16133635 | 0.033322917 |
| Ap3d1 | 0.127422468 | 0.033330283 |
| Pltp | 0.36467113 | 0.03335694 |
| Zfp266 | -0.106255219 | 0.033362522 |
| Btnl9 | 0.118603549 | 0.033365667 |
| Nrap | 0.100046702 | 0.033383424 |
| Ccl1 | -0.05758128 | 0.033391072 |
| Zfp825 | -0.1275197 | 0.033399357 |
| Plxnb2 | 0.183578996 | 0.033413438 |
| Gm10548 | -0.085234603 | 0.033473755 |
| Acox1 | 0.13895117 | 0.033480046 |
| Tmem213 | 0.072284712 | 0.033484845 |
| Itga7 | 0.085029289 | 0.03348692 |
| Cgrrf1 | -0.154934809 | 0.033505458 |
| Iqsec1 | 0.26527815 | 0.033508639 |
| Hmgb3 | -0.142066991 | 0.033522199 |
| Rhobtb3 | -0.10289725 | 0.033552342 |
| Nipa2 | -0.120701061 | 0.03357718 |
| Csnk1g1 | 0.07159252 | 0.033581944 |
| Ankrd52 | 0.121588755 | 0.033586991 |
| Casp12 | -0.296883466 | 0.033615372 |
| 6330403A02Rik | 0.103554904 | 0.033682057 |
| Gm10324 | -0.083386435 | 0.033691061 |
| Rev1 | -0.109261085 | 0.033756243 |
| Prorsd1 | -0.152192791 | 0.033762907 |
| Dcbld1 | -0.247615601 | 0.033828466 |
| Lasp1 | 0.199147267 | 0.033833304 |
| Parp8 | 0.156370032 | 0.033843882 |
| H2-Q1 | -0.127267233 | 0.033929917 |
| Rnu3a | -0.384540884 | 0.033934621 |
| Cd69 | -0.0750317 | 0.033935375 |
| Chchd2 | -0.175164387 | 0.033966473 |
| Adamts3 | -0.237395833 | 0.034030023 |
| Asf1a | -0.183652631 | 0.034030155 |
| Gm2695 | -0.189851482 | 0.034051974 |
| Kansl1 | 0.132032409 | 0.034063377 |
| Eif2s2 | -0.104212694 | 0.034085157 |
| Tusc5 | 0.152720591 | 0.034099583 |
| Ppp1r13l | 0.125536428 | 0.034122372 |
| 4930449I04Rik | 0.070314509 | 0.03414003 |
| Syce1l | 0.10611343 | 0.034154947 |
| Casp1 | -0.227289184 | 0.034172948 |
| Usp16 | -0.098639486 | 0.034190426 |
| Pde4a | 0.094606379 | 0.034193585 |
| Olfr1463 | -0.095642368 | 0.034199643 |
| Rfx3 | 0.110172357 | 0.034213632 |
| Mut | -0.097143954 | 0.034259147 |
| Zfp870 | -0.064831317 | 0.034264655 |
| Gadd45a | 0.19452677 | 0.034273967 |
| Tbc1d7 | -0.190171971 | 0.034298668 |
| Rcbtb2 | -0.184993472 | 0.034304632 |
| Gpr180 | -0.150630768 | 0.03435109 |
| Vps45 | -0.127013154 | 0.034363821 |
| Gm11711 | 0.192064789 | 0.034377276 |
| Mettl22 | -0.15919095 | 0.034400437 |
| Sox10 | 0.14420808 | 0.034438809 |
| Zfp882 | -0.093392205 | 0.034475338 |
| Rnf14 | -0.086509657 | 0.034529533 |
| L2hgdh | -0.117143426 | 0.034537675 |
| Tm2d2 | -0.191465557 | 0.034549332 |
| Dnajb6 | -0.084881516 | 0.034569616 |
| Gaa | 0.100678727 | 0.034572185 |
| Dennd1b | -0.129470546 | 0.034584224 |
| Abca9 | 0.220006963 | 0.034585414 |
| Sdccag8 | 0.13248953 | 0.034603825 |
| Lamp2 | -0.073268782 | 0.03462937 |
| Dusp11 | 0.09311125 | 0.03464081 |
| Gng12 | 0.115664768 | 0.034651006 |
| Lin28a | 0.054279415 | 0.034683063 |
| Spnb1 | 0.220145992 | 0.034728538 |
| Zfp322a | -0.121834694 | 0.034755479 |
| Pdzd11 | -0.192763755 | 0.034757773 |
| Derl2 | -0.172714846 | 0.034773769 |
| Slc25a40 | -0.075255091 | 0.034820735 |
| Otol1 | 0.069477541 | 0.034843951 |
| Slc36a2 | 0.283578692 | 0.03485222 |
| Cyp1b1 | 0.289992063 | 0.034852779 |
| Glod4 | -0.100568509 | 0.034932685 |
| Chd3 | 0.121431389 | 0.034946955 |
| Fam163a | 0.056255248 | 0.034952024 |
| Cyp20a1 | -0.171025509 | 0.034984158 |
| Eno1 | 0.079817545 | 0.034993745 |
| Myeov2 | -0.223528893 | 0.035043263 |
| Gna13 | 0.094352852 | 0.035049006 |
| 0610010F05Rik | -0.123727452 | 0.035070536 |
| Gm5901 | 0.062037641 | 0.035071278 |
| Zfp2 | -0.138070398 | 0.035089035 |
| Krba1 | 0.106399394 | 0.035092963 |
| Zfp707 | 0.078150844 | 0.035114151 |
| Uqcr10 | -0.118701433 | 0.035124099 |
| Rsrc1 | -0.124122892 | 0.035158626 |
| Tomm7 | -0.153525675 | 0.035174201 |
| Tdh | 0.089492435 | 0.035189745 |
| Sec14l2 | 0.103834066 | 0.035201214 |
| Erbb3 | 0.148134044 | 0.035210805 |
| Eef1a1 | -0.266179118 | 0.035251449 |
| Hmgcs2 | 0.087929597 | 0.035252396 |
| Rell1 | 0.175777343 | 0.035253954 |
| Cybb | 0.100058892 | 0.035296199 |
| Orc2 | -0.164622742 | 0.035321421 |
| Tns1 | 0.129484107 | 0.035324279 |
| Plod1 | 0.280384358 | 0.035334396 |
| Bbs7 | -0.148280981 | 0.035353564 |
| Psmb1 | -0.159512909 | 0.035384623 |
| Zik1 | -0.139070892 | 0.035388281 |
| Hist1h3i | 0.071060959 | 0.035396632 |
| Gcnt7 | 0.080945708 | 0.03549556 |
| Myo9b | 0.182901568 | 0.035496972 |
| Pard6g | -0.08106996 | 0.035543169 |
| 1110034B05Rik | -0.159448705 | 0.035553691 |
| Cnot6 | 0.073602521 | 0.035566816 |
| Trim65 | 0.098045345 | 0.03558733 |
| 5730469M10Rik | 0.087084057 | 0.035599135 |
| Mustn1 | 0.296292445 | 0.035605908 |
| Sh3kbp1 | 0.311741847 | 0.035655212 |
| Xirp2 | 0.27685443 | 0.03566543 |
| Mmp28 | 0.090669742 | 0.035668696 |
| Hist1h2ag | 0.067995606 | 0.035710536 |
| Ankrd49 | -0.284222085 | 0.03571128 |
| Diablo | -0.137061755 | 0.035717131 |
| Cbr3 | -0.106349794 | 0.035747456 |
| Rsl24d1 | -0.188033005 | 0.035759504 |
| Tfe3 | 0.084589313 | 0.03579306 |
| Asphd1 | 0.109819004 | 0.035871881 |
| H2-M3 | -0.2931965 | 0.035874121 |
| Srek1ip1 | -0.137005946 | 0.035894386 |
| Slc35e3 | 0.176809642 | 0.03594541 |
| Zmiz1 | 0.164961462 | 0.03596445 |
| Acin1 | 0.093628168 | 0.035967462 |
| Tubgcp6 | 0.180437055 | 0.035978863 |
| Zfp457 | -0.106590428 | 0.03599223 |
| Ly6g6c | 0.162057906 | 0.036098094 |
| Zfp341 | 0.113857521 | 0.036196363 |
| Ciz1 | 0.109004687 | 0.036215754 |
| 9430023L20Rik | 0.21049119 | 0.036261834 |
| Tspan15 | -0.128818937 | 0.036268227 |
| Zfp934 | -0.146097195 | 0.036275691 |
| Fabp3 | 0.100606519 | 0.036328252 |
| Prkab2 | 0.121220803 | 0.036365664 |
| Marveld1 | 0.19199562 | 0.036389594 |
| Fam181b | -0.069255848 | 0.036392703 |
| Cox4i1 | -0.172118034 | 0.036410176 |
| Rpl24 | -0.079567737 | 0.036436241 |
| Hmgb1-rs17 | -0.078902299 | 0.036451664 |
| Olfr1382 | 0.077724023 | 0.036477251 |
| Tns4 | 0.421972933 | 0.036479149 |
| Gga3 | 0.081214723 | 0.036501415 |
| Rbfa | -0.138064075 | 0.036508059 |
| Gmppb | -0.236439626 | 0.036512856 |
| Rpp38 | -0.173085415 | 0.036530163 |
| Cuedc2 | -0.225113411 | 0.036541568 |
| Bbs2 | 0.074909196 | 0.036552366 |
| Vkorc1 | -0.204108627 | 0.036581712 |
| Msh2 | -0.080201422 | 0.03658496 |
| Wisp3 | 0.107920721 | 0.036595149 |
| Dctn1 | 0.096773895 | 0.03661771 |
| Lmtk3 | 0.134668114 | 0.03666776 |
| 4930417G10Rik | 0.064979719 | 0.036692523 |
| Hax1 | -0.256995053 | 0.036702134 |
| Penk | 0.839697286 | 0.036709082 |
| 1110054O05Rik | -0.12899935 | 0.036713742 |
| Mkl1 | 0.117029848 | 0.036732297 |
| 4930591A17Rik | 0.076201362 | 0.036771724 |
| Tjp3 | 0.106992088 | 0.036788753 |
| Nanp | -0.15605183 | 0.036831683 |
| Wipf2 | 0.074081125 | 0.036833099 |
| Phxr1 | 0.147539871 | 0.036843797 |
| Cd44 | 0.126741369 | 0.036858251 |
| Bcl7b | -0.111698678 | 0.0368637 |
| Kcnj11 | 0.07659304 | 0.036897976 |
| Slc39a8 | -0.214204388 | 0.036912253 |
| Gnl3l | -0.056998864 | 0.036914311 |
| Vrk2 | -0.145878369 | 0.036936436 |
| Vbp1 | -0.232746585 | 0.03693842 |
| Prlr | 0.083612593 | 0.036971308 |
| Dock6 | 0.152786026 | 0.036973029 |
| Rpl7l1 | -0.098990018 | 0.037035971 |
| Rgag4 | -0.132625093 | 0.037039379 |
| Ptprs | 0.350469574 | 0.037047578 |
| Zmiz2 | 0.119094696 | 0.037059704 |
| Cbwd1 | -0.114464401 | 0.037088854 |
| H2-Bl | -0.108953109 | 0.037090594 |
| Clip2 | 0.182653713 | 0.037114916 |
| Patl1 | 0.088007177 | 0.037167284 |
| Fat4 | 0.093672133 | 0.03718607 |
| Vdac3 | -0.116331643 | 0.037232865 |
| Vdac3 | -0.116331643 | 0.037232865 |
| Ttc32 | -0.172484219 | 0.037270246 |
| Tor3a | -0.183071577 | 0.037298249 |
| Eif2c2 | 0.194364911 | 0.037303628 |
| Cdkl2 | -0.082040629 | 0.03735648 |
| Mrgprf | 0.092094976 | 0.037369917 |
| Pcdhb8 | -0.0782159 | 0.037384872 |
| Slc16a6 | 0.372853575 | 0.037394662 |
| Ccdc72 | -0.149529022 | 0.03748574 |
| Plek2 | -0.260934934 | 0.037522581 |
| Plekha6 | 0.146131658 | 0.037549307 |
| Zfp185 | 0.120194337 | 0.037621243 |
| Timm8b | -0.248971938 | 0.037644376 |
| Prkca | 0.234462794 | 0.037687327 |
| Rundc3b | -0.149698427 | 0.037732265 |
| Usp10 | -0.06600711 | 0.037785076 |
| Pfdn5 | -0.185298968 | 0.037806507 |
| Rhbdd2 | -0.065519238 | 0.037811624 |
| Clps | 0.071068951 | 0.037859362 |
| 4430402I18Rik | -0.12558379 | 0.037886177 |
| Nnat | 0.180205317 | 0.037933041 |
| Mrpl22 | -0.15145852 | 0.037941865 |
| Ppp1cb | -0.082476884 | 0.037959691 |
| Rap1gap2 | 0.143001725 | 0.037969611 |
| Chrnb4 | 0.094220914 | 0.037972816 |
| Cald1 | 0.136072979 | 0.037985095 |
| Gramd4 | 0.103230665 | 0.037993452 |
| Tab1 | 0.088641859 | 0.038008808 |
| Gstt3 | 0.05889884 | 0.038022674 |
| Slc4a11 | 0.316253409 | 0.038048677 |
| Prkrir | -0.235644731 | 0.038072224 |
| Trim30a | -0.434872703 | 0.038090846 |
| Rps27a | -0.170371514 | 0.038123448 |
| Ubl4 | -0.123967824 | 0.038129059 |
| Ogdhl | -0.112718681 | 0.038185345 |
| Lipt2 | -0.226018907 | 0.038194914 |
| 4933403G14Rik | 0.059632548 | 0.038216622 |
| Gdpd5 | 0.210908141 | 0.038228478 |
| Zcchc4 | -0.138498795 | 0.03824392 |
| Hist1h2bj | 0.06906874 | 0.038258692 |
| Ccs | -0.060549239 | 0.038321846 |
| Tmem161b | -0.124283978 | 0.038333795 |
| Xrcc4 | -0.144523977 | 0.038397131 |
| P2rx4 | -0.072715883 | 0.038501752 |
| Coro2a | 0.179147869 | 0.038505535 |
| Galnt4 | -0.100615562 | 0.038539388 |
| Wdr90 | 0.078628356 | 0.038544551 |
| BC023105 | -0.541865503 | 0.038556815 |
| 2010317E24Rik | 0.062262576 | 0.038587174 |
| Cfl2 | -0.104844186 | 0.038593846 |
| H2afb1 | 0.141328504 | 0.038606869 |
| Jph4 | 0.069625353 | 0.038657825 |
| Samd4 | 0.319885135 | 0.038731253 |
| Snx13 | -0.116753261 | 0.038752406 |
| Pecr | -0.249196686 | 0.038767444 |
| Disp2 | 0.080776435 | 0.038804237 |
| 5730455O13Rik | -0.080298615 | 0.03887142 |
| Enpep | 0.112146276 | 0.038875733 |
| Pear1 | 0.079722389 | 0.038928652 |
| 1700010I14Rik | -0.139472313 | 0.038959664 |
| Ccdc149 | -0.11088195 | 0.038980543 |
| Zdhhc13 | -0.125949673 | 0.038987088 |
| Cort | 0.091013634 | 0.039046241 |
| Rsl24d1 | -0.204733509 | 0.039100147 |
| Commd2 | -0.275818505 | 0.039125826 |
| Rps27a | -0.172457212 | 0.039155377 |
| Nmnat3 | -0.059564941 | 0.039162717 |
| Wdr59 | 0.111580833 | 0.039164215 |
| Zbtb7b | 0.095462967 | 0.039176377 |
| Sbsn | 0.068126729 | 0.039191743 |
| Aoc3 | 0.220064014 | 0.039247655 |
| AY036118 | -0.107437132 | 0.039288315 |
| Hp | 0.521093225 | 0.03929881 |
| Tmem126a | -0.164632959 | 0.039345473 |
| Lipe | 0.156143337 | 0.039368385 |
| Angptl2 | 0.35851381 | 0.039410516 |
| Vdac3 | -0.099047941 | 0.039411504 |
| Slc35d2 | -0.202346298 | 0.039472828 |
| 5730528L13Rik | -0.111843096 | 0.039544319 |
| D230037D09Rik | -0.11066027 | 0.039570036 |
| Pla2g16 | -0.203228667 | 0.0396241 |
| Ptges3 | -0.09864324 | 0.039658969 |
| Snord58b | -0.076455664 | 0.039660424 |
| Clec2g | -0.081862313 | 0.039670707 |
| Rpap2 | -0.122727234 | 0.039683241 |
| Fbxw22 | -0.075931531 | 0.039693607 |
| Serf1 | -0.178516981 | 0.03969538 |
| Snora31 | -0.348522918 | 0.039709946 |
| Heyl | 0.065106648 | 0.039712813 |
| Hells | -0.160372832 | 0.039744739 |
| Zfp617 | -0.128431359 | 0.039776893 |
| Olfr373 | 0.113769575 | 0.039807153 |
| Olfm4 | 0.08068467 | 0.03984119 |
| Foxj2 | 0.1408583 | 0.039914279 |
| 5730408K05Rik | -0.166719399 | 0.039916489 |
| H2-Eb1 | -0.070194518 | 0.039955529 |
| Tpd52l1 | -0.155700504 | 0.039981929 |
| Ctsw | -0.187203007 | 0.039986456 |
| Tpm2 | 0.29522644 | 0.040026624 |
| 2210408I21Rik | -0.107753831 | 0.040125258 |
| Mtmr9 | -0.085097084 | 0.040135746 |
| Yipf4 | -0.080775909 | 0.040164627 |
| E2f3 | -0.067232295 | 0.040180568 |
| Ppib | -0.121030001 | 0.040193942 |
| Sh3pxd2b | 0.154691013 | 0.040241124 |
| Tyms-ps | -0.133485223 | 0.040313062 |
| Sfpq | 0.172759606 | 0.040315893 |
| D930048N14Rik | 0.165927012 | 0.040362469 |
| 2810002N01Rik | -0.200744662 | 0.040411195 |
| Azi2 | -0.116329481 | 0.040425462 |
| Cbfa2t3 | 0.120895904 | 0.040459427 |
| Arhgef12 | -0.255415992 | 0.040486955 |
| Ces1a | 0.064600587 | 0.040489646 |
| Nt5dc1 | -0.087788852 | 0.040509189 |
| Eif2a | -0.115016799 | 0.040531955 |
| Rps27a | -0.171280625 | 0.04053474 |
| Mib2 | 0.070823061 | 0.040545029 |
| Mlycd | -0.128578849 | 0.040577362 |
| Fmnl3 | 0.145302944 | 0.040595158 |
| Gnas | -0.088423635 | 0.040599875 |
| R74862 | -0.093408374 | 0.040603353 |
| Mipol1 | -0.144370116 | 0.040673266 |
| Mybl2 | 0.08450507 | 0.040691702 |
| Gga1 | 0.122388737 | 0.040732546 |
| Gm5595 | -0.227810862 | 0.040744011 |
| Sorcs2 | 0.348531454 | 0.040762787 |
| Slc6a12 | 0.098456316 | 0.04081501 |
| Tob2 | 0.092954581 | 0.040816436 |
| Matr3 | -0.102698309 | 0.040816439 |
| Pigyl | -0.108382657 | 0.040832915 |
| Sgk1 | -0.077168653 | 0.040876918 |
| Ttc8 | -0.125850114 | 0.040881802 |
| Ighg | -0.053365861 | 0.040921209 |
| Hspe1 | -0.249033003 | 0.040945846 |
| Rab27a | -0.153533518 | 0.040980094 |
| Las1l | 0.067097236 | 0.041009268 |
| Lsm6 | -0.080832823 | 0.041017323 |
| Dleu2 | -0.063858373 | 0.041029477 |
| Cox5a | -0.118633964 | 0.041059708 |
| Htatip2 | -0.106165478 | 0.04112373 |
| Yars2 | -0.129446136 | 0.041125495 |
| Fam70b | 0.071000436 | 0.041129339 |
| Actl9 | 0.055693004 | 0.041141505 |
| Hmgb1 | -0.068760705 | 0.041143912 |
| Prrt2 | 0.098345155 | 0.041151694 |
| Zbtb26 | -0.112383162 | 0.041159601 |
| BC052040 | 0.102896929 | 0.041217521 |
| Wnk1 | 0.174179088 | 0.041226159 |
| 4833403I15Rik | 0.121933363 | 0.041234086 |
| Srsf3 | -0.07619117 | 0.041259381 |
| Des | 0.447056065 | 0.041288757 |
| Cluap1 | -0.123644785 | 0.041344661 |
| Prr7 | 0.081750588 | 0.041358516 |
| Gm12185 | -0.097001171 | 0.041399899 |
| Il2rb | -0.123135764 | 0.041425101 |
| Tsc1 | 0.107587947 | 0.041471641 |
| Snord96a | -0.183268856 | 0.041518283 |
| Ankrd32 | -0.128344167 | 0.041523957 |
| Sirt3 | -0.115365151 | 0.041545132 |
| Csnk1a1 | 0.065242656 | 0.041546377 |
| Lepr | 0.284489407 | 0.041621025 |
| Ublcp1 | -0.151991948 | 0.041624919 |
| Chac2 | -0.252780071 | 0.041639321 |
| Gbas | -0.171937343 | 0.041657179 |
| Klhdc2 | -0.197656271 | 0.041660401 |
| Rhox1 | 0.100360712 | 0.041671132 |
| Gnb1 | 0.100251267 | 0.041716274 |
| Trmu | 0.15452477 | 0.041742114 |
| Adck5 | 0.096112211 | 0.041760705 |
| Mcpt4 | 0.113298216 | 0.041846909 |
| A530016L24Rik | 0.077902907 | 0.041870613 |
| Nxt1 | -0.119417583 | 0.041889975 |
| Gm5712 | 0.070052481 | 0.041899701 |
| Rbfox2 | 0.117427551 | 0.041922619 |
| Pde6g | 0.082338748 | 0.041975197 |
| Tpp1 | 0.065203332 | 0.041991881 |
| Zfp71-rs1 | -0.103227733 | 0.041998537 |
| Cenpc1 | -0.099292591 | 0.042020604 |
| Rtkn | 0.101964899 | 0.042053501 |
| Rel | 0.170052353 | 0.042066739 |
| Clcn7 | 0.127676554 | 0.042070169 |
| Rps10 | -0.080961728 | 0.042100323 |
| Stard8 | 0.13802748 | 0.04211019 |
| Man1c1 | 0.253868363 | 0.042111931 |
| Slc19a1 | 0.107769626 | 0.042211437 |
| S100a13 | -0.173093488 | 0.042221469 |
| Zfp563 | -0.097824098 | 0.042266033 |
| Hnrnpk | -0.076474504 | 0.042286242 |
| H2-K2 | -0.143353325 | 0.042293296 |
| AB041803 | 0.422174488 | 0.042327956 |
| Etos1 | 0.077989828 | 0.042337596 |
| D17H6S56E-5 | -0.105388056 | 0.042388952 |
| Zxdc | 0.109520625 | 0.042411923 |
| Apoc4 | 0.069415812 | 0.042418496 |
| Cd7 | -0.347221137 | 0.042420928 |
| Ifi35 | -0.206175636 | 0.042442227 |
| Fpr-rs3 | 0.096388485 | 0.042449518 |
| Brms1 | -0.135876938 | 0.04245562 |
| Rragc | 0.060259611 | 0.042455869 |
| 2010109K11Rik | -0.077205475 | 0.042475054 |
| 2410089E03Rik | 0.159774578 | 0.042481866 |
| Pigv | 0.178522123 | 0.042514278 |
| Tap1 | -0.290993137 | 0.042516667 |
| Bche | -0.336200074 | 0.042519109 |
| Mea1 | -0.193274827 | 0.042531908 |
| Tmx2 | -0.086526346 | 0.042534668 |
| Etfb | -0.114935579 | 0.042557166 |
| Dock6 | 0.140522332 | 0.042579007 |
| Slc13a4 | 0.050971087 | 0.042596444 |
| Napepld | 0.113005374 | 0.042599775 |
| Grifin | 0.081233969 | 0.042618326 |
| Bend7 | -0.284170517 | 0.042662901 |
| Ddrgk1 | -0.102461977 | 0.04267067 |
| Mpv17l | -0.235183558 | 0.042671793 |
| Sp140 | -0.163088828 | 0.042699116 |
| Dvl1 | 0.129487228 | 0.042796971 |
| Sh3rf1 | 0.108162627 | 0.042829399 |
| Chd4 | 0.136437536 | 0.042845361 |
| Slc40a1 | 0.292058839 | 0.04284938 |
| Zfp955a | -0.099883786 | 0.042873537 |
| Hist1h2ai | 0.065943348 | 0.042893954 |
| Nkg7 | -0.254582369 | 0.042907343 |
| Sec11c | -0.181203442 | 0.042926781 |
| Pomp | -0.176047865 | 0.042931985 |
| Vmn2r43 | -0.099603664 | 0.042942073 |
| 0610010B08Rik | -0.233030008 | 0.042947517 |
| 0610010B08Rik | -0.233030008 | 0.042947517 |
| Eif4g3 | 0.237718817 | 0.042975723 |
| Wdr45l | -0.246138817 | 0.043025374 |
| Zfp260 | -0.101239882 | 0.04303187 |
| Fam193b | 0.083690276 | 0.043096018 |
| Lgals8 | -0.07141866 | 0.043165451 |
| Ltv1 | -0.079894733 | 0.04317679 |
| Srgap2 | 0.140621925 | 0.043192771 |
| G630016D24Rik | 0.096322893 | 0.043194106 |
| R3hdml | 0.085323147 | 0.043213143 |
| Adap2 | 0.133040701 | 0.043230909 |
| 1700054O13Rik | 0.057588261 | 0.043273828 |
| Spcs2 | -0.073991886 | 0.043332332 |
| Zbtb43 | 0.071628256 | 0.043363319 |
| Sec61b | -0.076375912 | 0.043394941 |
| Gm13375 | -0.131241421 | 0.043473515 |
| Slc6a6 | 0.211827451 | 0.043479802 |
| Wnk1 | 0.197572137 | 0.043481244 |
| Cpxm2 | 0.405654156 | 0.04348901 |
| Sfn | 0.121922413 | 0.043501981 |
| Tmem9b | -0.097795424 | 0.043511243 |
| Dot1l | 0.115960577 | 0.043511929 |
| Eif2ak3 | 0.137743249 | 0.043525576 |
| Hmcn1 | 0.124485552 | 0.043592807 |
| Aatk | 0.129374067 | 0.043604127 |
| Zfp939 | -0.155346982 | 0.043611193 |
| Lrdd | 0.067844597 | 0.043659996 |
| Ccdc53 | -0.076587717 | 0.043707992 |
| Dynlt3 | -0.097678063 | 0.043752403 |
| Mbnl2 | 0.127640272 | 0.043755626 |
| Hes2 | 0.070358149 | 0.043785969 |
| 1110001A16Rik | -0.27645534 | 0.043787211 |
| Pcp2 | 0.079016826 | 0.043825973 |
| Tmem202 | -0.077195779 | 0.043829037 |
| Zfp449 | -0.112565944 | 0.04384479 |
| Dyrk3 | -0.159970067 | 0.043932026 |
| Plekhm1 | 0.149055401 | 0.043941281 |
| Plxna4 | 0.065747787 | 0.043942782 |
| Eya3 | 0.194816841 | 0.043981326 |
| Cox11 | -0.080689681 | 0.044018115 |
| Sra1 | -0.14885687 | 0.04417966 |
| Dock11 | 0.118460523 | 0.044221145 |
| Mical3 | 0.181109108 | 0.044295299 |
| Hspb6 | 0.170464067 | 0.044306965 |
| l7Rn6 | -0.151361849 | 0.044407607 |
| Anp32-ps | -0.147918088 | 0.044428338 |
| Alms1 | 0.10963869 | 0.044429994 |
| Fam186b | 0.06083743 | 0.044463521 |
| Srgap1 | 0.094554061 | 0.044482073 |
| Gbp2 | -0.319082019 | 0.044505134 |
| Sumo2 | -0.057150752 | 0.044507927 |
| Thap4 | -0.159444377 | 0.044517089 |
| Triap1 | -0.295536874 | 0.044535922 |
| Slc25a37 | 0.148178635 | 0.044537818 |
| Snora70 | -0.204385345 | 0.04454723 |
| Irs3 | 0.098983281 | 0.044565088 |
| Rcn2 | -0.140651965 | 0.044573532 |
| Abca8a | 0.140062037 | 0.044658137 |
| Mbd6 | 0.168198233 | 0.044683113 |
| Dock6 | 0.187192793 | 0.044690369 |
| Gm11127 | -0.151674719 | 0.044754402 |
| Zfp52 | -0.156323692 | 0.044763025 |
| Myo18b | 0.07495855 | 0.044821758 |
| Zfp236 | 0.137911279 | 0.044892822 |
| Nfatc1 | 0.098207084 | 0.045005213 |
| Psmd10 | -0.201663961 | 0.04506954 |
| Prdm15 | 0.146289245 | 0.045079948 |
| 2610002M06Rik | -0.155566569 | 0.045082899 |
| Zfp87 | -0.102011745 | 0.045095608 |
| Tmem88 | -0.055408033 | 0.045141497 |
| Spata5l1 | 0.063574145 | 0.04514808 |
| 2410089E03Rik | 0.161161216 | 0.045148225 |
| Ctdsp2 | 0.146455717 | 0.045163891 |
| 1810037I17Rik | -0.084389888 | 0.045185051 |
| Rpe | -0.094024733 | 0.045196454 |
| Cox15 | -0.107696287 | 0.045206186 |
| 3110040N11Rik | -0.10675308 | 0.045217076 |
| Bcl2l11 | 0.232694384 | 0.045247397 |
| Smyd3 | 0.094598616 | 0.045278766 |
| Sharpin | 0.093746547 | 0.045299033 |
| Ephx3 | 0.082765807 | 0.045342621 |
| Col4a6 | 0.216154594 | 0.045385751 |
| Spata2l | 0.086807547 | 0.045388572 |
| Papd4 | -0.066653575 | 0.045400169 |
| Cox5b | -0.10082492 | 0.045438295 |
| BC002059 | -0.204813745 | 0.0454446 |
| C1qbp | -0.129045567 | 0.045458413 |
| Lmbr1l | 0.109101706 | 0.045476075 |
| Hspe1 | -0.244114516 | 0.045510817 |
| Top3a | 0.053891728 | 0.045533577 |
| Eda2r | -0.191135568 | 0.045548625 |
| Nmd3 | -0.134993251 | 0.045552993 |
| Muted | -0.176722633 | 0.045555888 |
| Tpra1 | 0.161995046 | 0.045562144 |
| Gpr68 | 0.078143833 | 0.045571415 |
| 1700071K01Rik | -0.147738214 | 0.045571793 |
| Cox6b2 | 0.0956759 | 0.045574441 |
| Armc10 | -0.085744198 | 0.045644916 |
| Zfp758 | -0.151173918 | 0.045659982 |
| Coro1c | 0.194395594 | 0.045712371 |
| Heph | -0.124931664 | 0.045748398 |
| Fam38a | 0.143517029 | 0.045758009 |
| Snord58b | -0.074603291 | 0.045760334 |
| Ndufs5 | -0.283010216 | 0.045762358 |
| Dnm1 | 0.173565436 | 0.045766211 |
| Hmgb1 | -0.047724069 | 0.045768151 |
| Szt2 | 0.187137376 | 0.045778869 |
| Ndufs6 | -0.089490982 | 0.045809055 |
| 1500031L02Rik | -0.068538877 | 0.045837561 |
| Wdr67 | 0.208651522 | 0.045857022 |
| Enpp5 | -0.253876804 | 0.045857649 |
| Snrpd2 | -0.215342531 | 0.04589266 |
| 2900097C17Rik | 0.14499436 | 0.045900482 |
| Thra | 0.093209152 | 0.045911368 |
| Rictor | 0.179593921 | 0.045947004 |
| Narfl | -0.103773101 | 0.045990586 |
| Tmem85 | -0.086313465 | 0.04599184 |
| Fcnb | 0.048253302 | 0.046023722 |
| Ifna2 | -0.123432533 | 0.046036008 |
| Gm5136 | -0.060350021 | 0.046043697 |
| Fbxw2 | -0.059173617 | 0.046063048 |
| Gtdc1 | -0.168107624 | 0.046083836 |
| Apof | -0.247026543 | 0.046130157 |
| BC089491 | 0.106595052 | 0.046146257 |
| 2700029M09Rik | -0.103917574 | 0.046158594 |
| Rab11a | -0.184108203 | 0.046158949 |
| Rps6ka2 | 0.059663565 | 0.046175824 |
| Trpv2 | 0.11186142 | 0.046182274 |
| Dock6 | 0.192629941 | 0.046196906 |
| Man2b1 | 0.096915757 | 0.046227716 |
| Upf1 | 0.128320295 | 0.046244457 |
| Hspa13 | -0.171438228 | 0.046285901 |
| Abcg4 | 0.080543614 | 0.046294607 |
| Fam70a | -0.171508564 | 0.046300118 |
| 1700040L02Rik | -0.312613507 | 0.046348569 |
| Rsu1 | 0.071564994 | 0.046350396 |
| Slc30a4 | -0.192443419 | 0.046373358 |
| D18Ertd653e | 0.175694098 | 0.04638467 |
| Irak2 | 0.136990514 | 0.046388399 |
| Arid1a | 0.227754223 | 0.046397855 |
| Kbtbd3 | -0.116157622 | 0.046407913 |
| Skp2 | 0.14374562 | 0.046440233 |
| Col24a1 | 0.062910369 | 0.046470206 |
| Gm13154 | -0.049824102 | 0.04648279 |
| Kif14 | 0.099260751 | 0.046533292 |
| Rnf207 | 0.158755263 | 0.046542875 |
| Uxt | -0.154553676 | 0.046614515 |
| Fgf21 | 0.077229457 | 0.046624543 |
| Pdlim7 | 0.220756211 | 0.046626503 |
| Armc1 | -0.121350347 | 0.046683041 |
| Pdzd7 | 0.066728007 | 0.046723586 |
| Tgs1 | 0.087469695 | 0.046726334 |
| Sipa1 | 0.084332786 | 0.046811038 |
| Mir22 | -0.220405779 | 0.046812093 |
| Rnf186 | 0.099193464 | 0.04683122 |
| Tmem100 | 0.068005759 | 0.046851718 |
| Krtap9-3 | 0.09768109 | 0.046862559 |
| Polr1d | -0.155723429 | 0.046886918 |
| 1700001G17Rik | -0.053140329 | 0.046897669 |
| Pde6d | -0.127847049 | 0.04692096 |
| Celf5 | 0.118034216 | 0.046921145 |
| D130040H23Rik | -0.068054224 | 0.046922639 |
| Snord118 | -0.42168602 | 0.046924202 |
| Snord118 | -0.42168602 | 0.046924202 |
| A830007P12Rik | -0.065337078 | 0.046951413 |
| Card10 | 0.121853259 | 0.046952226 |
| Qpct | -0.184687851 | 0.046969783 |
| Gyg | -0.112716976 | 0.04698135 |
| Alkbh7 | -0.225611129 | 0.046987673 |
| S100a16 | -0.137742549 | 0.047039527 |
| Dstn | -0.08645897 | 0.047062321 |
| Dlx3 | 0.079156311 | 0.047093821 |
| Ttpa | 0.226857399 | 0.047175754 |
| Abi2 | -0.083871944 | 0.047183618 |
| Mgat4a | -0.241496666 | 0.047185521 |
| Pbrm1 | 0.125229489 | 0.04723347 |
| 1810048J11Rik | -0.129994161 | 0.047233902 |
| Prpf38b | 0.086104659 | 0.047245295 |
| Sf3b5 | -0.15874909 | 0.047247286 |
| Gata1 | 0.055579066 | 0.047250986 |
| Tra2a | 0.214580647 | 0.047265153 |
| Cst7 | -0.072579935 | 0.0473028 |
| Gm8096 | 0.091040086 | 0.047316318 |
| Klf4 | 0.123720075 | 0.047334142 |
| Stk4 | 0.092200511 | 0.047354177 |
| Gbp6 | -0.472786754 | 0.047377471 |
| Nme3 | -0.22412967 | 0.047414092 |
| Rrp15 | -0.083499934 | 0.04745654 |
| Letm2 | 0.082153034 | 0.04747626 |
| Ripk3 | 0.137205329 | 0.047488678 |
| Psmg2 | -0.139781432 | 0.047493328 |
| Faim | -0.07462928 | 0.047530715 |
| Trim30d | -0.167885706 | 0.047532149 |
| Btbd10 | -0.119486523 | 0.047543787 |
| Armc8 | -0.096424933 | 0.047556285 |
| Mtg1 | -0.167793971 | 0.047584535 |
| Scrt2 | 0.121063578 | 0.047601949 |
| AA987161 | -0.163730388 | 0.047603473 |
| Sgca | 0.061574361 | 0.04763972 |
| 1810020D17Rik | -0.184242346 | 0.047641362 |
| S100a1 | -0.114789575 | 0.047645198 |
| Ccdc50 | -0.078001622 | 0.047649805 |
| Atp5h | -0.116070863 | 0.047693976 |
| Adcy3 | 0.139052622 | 0.047699117 |
| BC013529 | -0.125362822 | 0.047731436 |
| AW112010 | -0.166840534 | 0.04773471 |
| Apoa1bp | -0.097735747 | 0.047751945 |
| Eprs | 0.078737695 | 0.047773594 |
| Sdhaf1 | -0.25062341 | 0.04777766 |
| Npas2 | 0.494972112 | 0.047796913 |
| Gbp9 | -0.388644817 | 0.047800264 |
| Mknk1 | 0.185036074 | 0.047802778 |
| 1810027O10Rik | -0.249434098 | 0.047822644 |
| Rps15a | -0.106824954 | 0.047885824 |
| Mdh1 | -0.062356297 | 0.047954615 |
| C6 | 0.163454191 | 0.04798304 |
| Mrpl54 | -0.220828155 | 0.047992413 |
| Osbpl9 | 0.080680415 | 0.048020233 |
| Lmna | 0.119304742 | 0.04802503 |
| Cpeb2 | 0.079668984 | 0.048031746 |
| Hax1 | -0.238329048 | 0.048040666 |
| Rcan3 | 0.218867726 | 0.04805917 |
| Polr2g | -0.175412865 | 0.048084776 |
| Ypel4 | -0.066049962 | 0.048096964 |
| Alg2 | 0.098162791 | 0.048105024 |
| Zim1 | 0.06954584 | 0.048107728 |
| Mug-ps1 | 0.074462054 | 0.0481273 |
| Rbpjl | 0.089696774 | 0.048134876 |
| Gm14326 | -0.164554696 | 0.048141518 |
| Pcolce2 | 0.247997565 | 0.04814781 |
| Myod1 | 0.056728385 | 0.048183263 |
| Fam26e | -0.350517789 | 0.048287395 |
| Klhdc1 | -0.109928256 | 0.048372488 |
| Figf | 0.22681366 | 0.048414033 |
| 1110008J03Rik | -0.101991299 | 0.048471093 |
| Styx | -0.136590809 | 0.048486582 |
| Gba2 | 0.078778324 | 0.048538327 |
| 0610037L13Rik | 0.094573078 | 0.048570825 |
| Sema4c | 0.11262284 | 0.048585763 |
| Csnk1e | 0.169084527 | 0.048598173 |
| Heatr7a | 0.165563149 | 0.048599032 |
| Sost | 0.068641408 | 0.048604733 |
| Nuak1 | 0.270432212 | 0.048630946 |
| 4933424B01Rik | 0.121779545 | 0.048671148 |
| Rpl35a | -0.087408116 | 0.048674606 |
| D730001G18Rik | 0.102402026 | 0.048691645 |
| Zfp560 | -0.067109599 | 0.048696374 |
| Tsen15 | -0.346676426 | 0.048707638 |
| Drap1 | -0.075144663 | 0.048708445 |
| Plekhg6 | 0.07792991 | 0.048717014 |
| Eif1 | 0.066041631 | 0.048720898 |
| Krt14 | 0.087530219 | 0.048757775 |
| Dcstamp | 0.07452686 | 0.048810704 |
| Top2a | -0.169087856 | 0.048832051 |
| Clcn6 | 0.132269378 | 0.04884537 |
| Rnf138 | -0.148693898 | 0.048847999 |
| O3far1 | 0.062631578 | 0.048961151 |
| Hspa9 | 0.075710311 | 0.048989094 |
| Elof1 | -0.191740962 | 0.049009175 |
| Pdzd7 | 0.123807629 | 0.049038592 |
| Arc | 0.165088971 | 0.049050112 |
| BC025920 | -0.105280396 | 0.04905372 |
| Zyx | 0.216565845 | 0.049248531 |
| Atp5k | -0.116101526 | 0.049250793 |
| Osbpl10 | 0.096214621 | 0.049271554 |
| Fyttd1 | -0.157913404 | 0.049272756 |
| Actg1 | 0.093167904 | 0.049302851 |
| 2700062C07Rik | -0.089994023 | 0.049308957 |
| Ybx2 | 0.101873912 | 0.049310557 |
| Lrrk1 | 0.159243717 | 0.049368451 |
| Arhgap22 | 0.15900799 | 0.049371582 |
| Appl2 | 0.115271787 | 0.04937784 |
| Mtap1b | 0.441103599 | 0.049426528 |
| Polrmt | 0.058180331 | 0.049459381 |
| Ccl12 | -0.143583637 | 0.049509192 |
| Bpnt1 | -0.123278284 | 0.049516599 |
| Il10 | -0.063719818 | 0.049554081 |
| Nisch | 0.128749659 | 0.049579385 |
| Ano4 | -0.055912187 | 0.049591553 |
| Rfesd | -0.134180247 | 0.049609503 |
| Kcnh7 | -0.094225336 | 0.049617675 |
| Ctbs | -0.146779885 | 0.049638499 |
| Smg6 | 0.134439767 | 0.049682819 |
| Pdxp | 0.128221953 | 0.049707711 |
| Acer3 | -0.160256201 | 0.049743457 |
| Mocs2 | -0.161461547 | 0.049745164 |
| Ccz1 | -0.109848705 | 0.049749776 |
| Dtymk | -0.177982507 | 0.049781431 |
| Bag2 | -0.129970477 | 0.049782116 |
| Rbbp9 | -0.098742931 | 0.04979295 |
| Ube2d4 | -0.066168864 | 0.049813869 |
| 4933434E20Rik | -0.107198493 | 0.049816774 |
| Fam38b | 0.14209207 | 0.049838583 |
| Zfp385a | 0.131546812 | 0.04984621 |
| Gpr157 | 0.074396169 | 0.049847794 |
| Tns1 | 0.253732863 | 0.049859295 |
| Hist1h2an | 0.066091618 | 0.049867038 |
| Crtc1 | 0.107941601 | 0.049885516 |
| Zfp78 | -0.104328811 | 0.049895544 |
| Fam33a | -0.147856899 | 0.049900662 |
| Hdhd2 | -0.073723624 | 0.049926039 |
| Snhg11 | 0.053185501 | 0.049966277 |
| Rn28s1 | 0.06272241 | 0.04996986 |

**Appendix Table S2 Differential gene expression of TNC high (WT/shc) to TNC low (KO/shTNC) tumors (11 weeks)**

RNA sequencing data, p-value < 0.05, N = 2.

| **Gene Symbol** | **logFC** | **P,Value** |
| --- | --- | --- |
| Rny1 | -2,7682 | 1,60E-13 |
| Snora34 | -3,8175 | 3,89E-12 |
| Lpl | -2,9790 | 3,64E-11 |
| Gm23245 | -4,1993 | 3,32E-10 |
| Gm26440 | -3,8742 | 3,99E-10 |
| Gm22888 | -5,6099 | 7,32E-10 |
| Gm24119 | -4,0004 | 9,39E-10 |
| Cfd | -7,1911 | 2,50E-09 |
| Gm24620 | -5,0872 | 4,08E-09 |
| Ccdc80 | -2,7813 | 6,66E-09 |
| Gm23130 | -5,0140 | 2,06E-08 |
| Gm25360 | -2,1566 | 2,25E-08 |
| Gm6665 | -4,3147 | 3,08E-08 |
| Gm23136 | -3,8460 | 6,16E-08 |
| Gm25514 | 4,1450 | 6,62E-08 |
| Gm25970 | -4,3036 | 6,70E-08 |
| Scarna3a | -4,4375 | 1,34E-07 |
| Gm24091 | -4,2081 | 3,06E-07 |
| Snord17 | -4,0144 | 3,04E-07 |
| Gm22270 | -3,2415 | 4,26E-07 |
| Snord90 | -4,8730 | 8,39E-07 |
| Gm26448 | -2,1212 | 1,30E-06 |
| Adh7 | -5,7137 | 2,33E-06 |
| Prkg2 | -4,5903 | 2,35E-06 |
| Snord111 | -4,4373 | 2,68E-06 |
| Mmp13 | -3,3616 | 3,10E-06 |
| Scarna6 | -4,0396 | 3,51E-06 |
| Krt34 | -5,7511 | 4,52E-06 |
| Snord87 | -3,9374 | 4,54E-06 |
| Gm23711 | -3,9597 | 5,06E-06 |
| Mcl1 | -1,9961 | 5,00E-06 |
| Gm25852 | -4,9345 | 5,39E-06 |
| Aldh1a3 | -6,3307 | 5,76E-06 |
| Gm12013 | -3,3316 | 5,91E-06 |
| Igf2bp1 | -5,9226 | 5,84E-06 |
| H2-K2 | 2,5798 | 7,68E-06 |
| Cd200r1 | -3,9655 | 8,21E-06 |
| Snora2b | -3,1141 | 9,80E-06 |
| Akr1c14 | -4,4647 | 1,03E-05 |
| Fst | -5,2478 | 1,10E-05 |
| Mir1955 | -3,6542 | 1,10E-05 |
| Gm26287 | -3,7226 | 1,21E-05 |
| Scarna3b | -3,4815 | 1,26E-05 |
| Gm25848 | -5,8957 | 1,34E-05 |
| Gm22247 | -3,4625 | 1,41E-05 |
| Rps13-ps1 | -2,7816 | 1,38E-05 |
| Gm23238 | 2,4944 | 1,92E-05 |
| Inpp5j | 3,6319 | 1,95E-05 |
| Csmd3 | -6,7237 | 2,17E-05 |
| Gm25813 | -2,4278 | 2,16E-05 |
| Gm22488 | -2,9839 | 2,38E-05 |
| Snord7 | -2,9541 | 2,53E-05 |
| Gm7308 | -2,9514 | 2,72E-05 |
| Dcun1d5 | -4,5188 | 2,83E-05 |
| Abca9 | -2,8518 | 2,95E-05 |
| Gm23971 | -2,8239 | 2,95E-05 |
| Dbp | 2,1104 | 3,12E-05 |
| Chchd2 | -2,4262 | 3,18E-05 |
| F13a1 | -4,6711 | 3,93E-05 |
| n-R5s122 | -3,7543 | 4,01E-05 |
| Mgat3 | -4,1545 | 4,13E-05 |
| Lrrk2 | -3,0513 | 4,29E-05 |
| Pdk4 | -5,4228 | 4,27E-05 |
| Adam22 | -4,3843 | 4,51E-05 |
| Plin1 | -6,4871 | 5,01E-05 |
| Gm25107 | -3,5027 | 5,52E-05 |
| Scn7a | -3,2633 | 6,53E-05 |
| Phldb2 | -2,4663 | 6,71E-05 |
| Snora23 | -3,0310 | 6,79E-05 |
| Abca8a | -3,6552 | 7,07E-05 |
| Pygl | -2,4057 | 7,75E-05 |
| Ephx2 | -8,3911 | 7,96E-05 |
| Fabp4 | -3,3004 | 8,06E-05 |
| Adipoq | -6,2975 | 8,42E-05 |
| Gm22009 | -2,4936 | 8,62E-05 |
| Tbcb | -1,6167 | 8,67E-05 |
| Eps8l1 | 2,1290 | 9,14E-05 |
| Gm22486 | -3,7517 | 9,04E-05 |
| Lsr | 1,6246 | 9,64E-05 |
| Prf1 | -4,4361 | 9,51E-05 |
| Timm10 | -2,2204 | 9,54E-05 |
| Gab1 | 1,6249 | 9,82E-05 |
| Epha3 | -6,5529 | 1,07E-04 |
| Gpx4 | -2,1177 | 1,07E-04 |
| Fads3 | -2,8190 | 1,17E-04 |
| Gm24514 | 4,3856 | 1,25E-04 |
| Gm25791 | -3,6986 | 1,25E-04 |
| mt-Atp6 | -2,4618 | 1,23E-04 |
| Snora7a | -3,5308 | 1,29E-04 |
| Ttn | -4,3635 | 1,29E-04 |
| Zfp521 | -3,2689 | 1,28E-04 |
| 1810059H22Rik | 4,3698 | 1,34E-04 |
| Azin1 | -1,5034 | 1,38E-04 |
| Lepre1 | -1,8295 | 1,39E-04 |
| RP23-448H3,2 | 4,1996 | 1,40E-04 |
| Gm24924 | 2,1945 | 1,45E-04 |
| Gm22422 | -3,0778 | 1,47E-04 |
| Adamts5 | -1,9658 | 1,56E-04 |
| Dock11 | -2,7624 | 1,57E-04 |
| Hoxc8 | -3,5321 | 1,63E-04 |
| Mir16-2 | -5,2644 | 1,68E-04 |
| Apbb1ip | -2,1777 | 1,76E-04 |
| Csgalnact2 | -1,7047 | 1,71E-04 |
| Gm25835 | -3,7643 | 1,75E-04 |
| Pcgf5 | -2,1892 | 1,72E-04 |
| Gm25939 | -1,4171 | 1,88E-04 |
| Lgals3 | 1,4976 | 1,90E-04 |
| Gm26391 | -3,1942 | 1,99E-04 |
| Gm25188 | -3,4520 | 2,02E-04 |
| n-R5-8s1 | -1,7313 | 2,23E-04 |
| Cyp2e1 | -6,6336 | 2,27E-04 |
| Mir6236 | 2,4842 | 2,37E-04 |
| Rgs5 | -1,5170 | 2,42E-04 |
| n-R5s139 | -3,4516 | 2,47E-04 |
| Syf2 | -1,5286 | 2,49E-04 |
| Eif1ax | -2,3496 | 2,55E-04 |
| Gm22701 | 3,6860 | 2,75E-04 |
| Milr1 | -3,0252 | 2,78E-04 |
| Gm24698 | -3,4468 | 2,80E-04 |
| n-R5s117 | -3,3192 | 2,86E-04 |
| Yap1 | -3,6721 | 2,90E-04 |
| Camkk1 | -2,6937 | 2,98E-04 |
| Grik3 | 2,5032 | 2,97E-04 |
| Plin4 | -5,3472 | 3,01E-04 |
| B4galnt2 | 6,4115 | 3,10E-04 |
| Hspb8 | -3,5395 | 3,12E-04 |
| Snord55 | -3,8599 | 3,29E-04 |
| E2f3 | -2,1058 | 3,34E-04 |
| 0610011F06Rik | 1,9484 | 3,60E-04 |
| Gm26457 | -3,6304 | 3,62E-04 |
| Gm28438 | -2,7057 | 3,61E-04 |
| Papss2 | -3,4943 | 3,73E-04 |
| Magt1 | -1,9962 | 3,80E-04 |
| Gm24447 | 2,7723 | 3,91E-04 |
| n-R5s108 | -3,5004 | 3,95E-04 |
| Gm26225 | -3,2703 | 4,12E-04 |
| Gm26387 | -2,7803 | 4,13E-04 |
| Lama2 | -1,8090 | 4,09E-04 |
| Sema3c | -2,3819 | 4,18E-04 |
| Serping1 | -2,1003 | 4,15E-04 |
| Snph | 2,5813 | 4,17E-04 |
| Bnc2 | -2,6279 | 4,33E-04 |
| Kpna4 | -1,3746 | 4,34E-04 |
| Hba-a1 | 2,3273 | 4,44E-04 |
| Gstm2 | -2,2076 | 4,52E-04 |
| Has2 | -4,2963 | 4,55E-04 |
| Snora69 | -2,7422 | 4,51E-04 |
| Agpat4 | -2,7305 | 4,82E-04 |
| n-R5s143 | -3,1661 | 4,82E-04 |
| Serpinb9b | -5,7041 | 4,76E-04 |
| Trpv6 | 4,5452 | 4,82E-04 |
| Gm23639 | -2,3134 | 4,86E-04 |
| Akr1b3 | -2,5544 | 5,02E-04 |
| Dnajb9 | -1,7646 | 5,20E-04 |
| Snora30 | -3,0124 | 5,21E-04 |
| Birc2 | -3,3890 | 5,80E-04 |
| Cfh | -2,5960 | 5,86E-04 |
| Gm23734 | -3,0581 | 5,77E-04 |
| Gm26165 | -2,9090 | 5,57E-04 |
| Itgb4 | 1,3560 | 5,78E-04 |
| Mrc1 | -4,1377 | 5,82E-04 |
| n-R5s105 | -3,4321 | 5,72E-04 |
| Psmb9 | 1,5343 | 5,73E-04 |
| Rn5s | -3,0968 | 5,62E-04 |
| Eno1 | -1,7192 | 6,16E-04 |
| Igkv16-104 | -8,5581 | 6,27E-04 |
| Sdsl | 5,1375 | 6,29E-04 |
| Arhgef1 | 1,4270 | 6,61E-04 |
| Gstk1 | -5,2861 | 6,59E-04 |
| Prkcz | 2,4398 | 6,65E-04 |
| Tlr7 | -2,6177 | 6,73E-04 |
| Abcb1b | -3,3479 | 6,87E-04 |
| Snord16a | -2,9259 | 6,85E-04 |
| Gm26175 | -2,5435 | 6,98E-04 |
| Gm26205 | -3,0160 | 7,37E-04 |
| Ptgs2 | -3,3569 | 7,39E-04 |
| Samd10 | 1,6623 | 7,41E-04 |
| Prkar1b | -3,0845 | 7,54E-04 |
| B4galt2 | -3,2029 | 7,63E-04 |
| 4930405O22Rik | -2,4697 | 7,69E-04 |
| Gm23624 | 4,6128 | 7,79E-04 |
| Gm22567 | 3,5483 | 7,93E-04 |
| Gas7 | -3,0068 | 8,10E-04 |
| Gm23658 | -2,3268 | 8,03E-04 |
| Pck1 | -5,3730 | 8,11E-04 |
| Actn3 | -5,2597 | 8,17E-04 |
| Tbc1d17 | 1,5206 | 8,34E-04 |
| Dync2h1 | -3,0529 | 8,67E-04 |
| Gm24146 | 2,9619 | 8,80E-04 |
| mt-Nd3 | -2,5775 | 8,77E-04 |
| Speg | -2,7024 | 8,84E-04 |
| Snord123 | -2,6573 | 9,49E-04 |
| Snord49b | -2,8900 | 9,46E-04 |
| Cox4i1 | 1,4449 | 9,94E-04 |
| Gm22025 | 3,1249 | 9,95E-04 |
| Gm22519 | -2,0652 | 1,02E-03 |
| Gng2 | -1,9851 | 1,02E-03 |
| Gm25776 | -3,0446 | 1,03E-03 |
| Nap1l1 | -1,3889 | 1,05E-03 |
| Gm24497 | -1,6029 | 1,08E-03 |
| Armcx2 | -2,9161 | 1,12E-03 |
| Bcam | -2,1523 | 1,12E-03 |
| Gm24613 | -3,0006 | 1,11E-03 |
| Nfam1 | -2,3497 | 1,13E-03 |
| Snord72 | -2,9309 | 1,13E-03 |
| Gm22973 | -2,1416 | 1,17E-03 |
| Gm23472 | -1,5659 | 1,22E-03 |
| Gm26109 | -3,3176 | 1,21E-03 |
| Mmp1b | -6,7933 | 1,22E-03 |
| Ngf | -8,6490 | 1,24E-03 |
| Pla2r1 | -2,4256 | 1,23E-03 |
| Ptn | -3,9379 | 1,23E-03 |
| Rassf4 | -1,8074 | 1,22E-03 |
| Snora21 | -2,2729 | 1,24E-03 |
| Gm24044 | -2,2758 | 1,26E-03 |
| Erdr1 | 1,5483 | 1,30E-03 |
| Cpsf4l | 3,5760 | 1,31E-03 |
| Snord15a | -2,4493 | 1,31E-03 |
| Gm26035 | 2,9399 | 1,36E-03 |
| Lipg | -2,9102 | 1,35E-03 |
| Mmd | -2,3002 | 1,34E-03 |
| n-R5s144 | -3,4015 | 1,35E-03 |
| Gm24095 | -3,8391 | 1,37E-03 |
| Glul | -1,4851 | 1,39E-03 |
| Gm26315 | 2,4261 | 1,39E-03 |
| Folr2 | -3,1133 | 1,40E-03 |
| Igsf8 | 1,5354 | 1,44E-03 |
| Gvin1 | -2,0503 | 1,48E-03 |
| Tns1 | -1,6848 | 1,48E-03 |
| C920025E04Rik | 1,6379 | 1,49E-03 |
| Il1rl1 | -3,5898 | 1,51E-03 |
| Gm24233 | -3,0994 | 1,57E-03 |
| H2-DMb2 | 1,9398 | 1,57E-03 |
| Hotairm1 | -2,0197 | 1,57E-03 |
| Ifi205 | -3,0064 | 1,57E-03 |
| Mirlet7a-1 | -3,0079 | 1,54E-03 |
| Msrb3 | -1,6304 | 1,57E-03 |
| n-R5s121 | -3,1509 | 1,53E-03 |
| Sec16b | -1,4787 | 1,55E-03 |
| Hs6st2 | -2,4464 | 1,59E-03 |
| Tmem45b | -7,1794 | 1,59E-03 |
| Snord11 | -3,0848 | 1,61E-03 |
| Adamts14 | -3,8762 | 1,63E-03 |
| Pglyrp1 | 1,4853 | 1,63E-03 |
| Rpph1 | 1,2257 | 1,64E-03 |
| Tfap2a | 1,8235 | 1,68E-03 |
| Htra1 | -3,0572 | 1,69E-03 |
| Ighv3-6 | -8,0980 | 1,70E-03 |
| Gm14403 | -3,2963 | 1,71E-03 |
| Gm22748 | -1,8875 | 1,75E-03 |
| Gm25636 | -2,1439 | 1,74E-03 |
| Snora36b | -2,0745 | 1,74E-03 |
| Snord35a | -2,4830 | 1,75E-03 |
| Gm23444 | -1,5320 | 1,77E-03 |
| Smim22 | 2,3558 | 1,78E-03 |
| Pfkl | 1,1819 | 1,81E-03 |
| Ucp1 | -7,2901 | 1,82E-03 |
| Mmgt1 | -1,7847 | 1,85E-03 |
| Ndn | -6,2243 | 1,86E-03 |
| Syncrip | -1,4797 | 1,87E-03 |
| Acaca | -1,6513 | 1,92E-03 |
| Flrt2 | -3,6030 | 1,91E-03 |
| Gm24616 | -1,5653 | 1,91E-03 |
| Gm25117 | -2,0322 | 1,91E-03 |
| Rtn4 | -1,1870 | 1,89E-03 |
| Gm24313 | -2,8662 | 1,96E-03 |
| Nin | -1,2975 | 1,96E-03 |
| Ptk7 | 1,1781 | 1,95E-03 |
| Rprl1 | 2,4788 | 1,96E-03 |
| Cav1 | -3,3461 | 2,03E-03 |
| Greb1 | -3,8452 | 2,04E-03 |
| Wasf1 | -4,3949 | 2,05E-03 |
| Col14a1 | -1,6296 | 2,10E-03 |
| Gm24139 | 2,8665 | 2,10E-03 |
| Igsf6 | -2,5372 | 2,09E-03 |
| Gm16136 | 1,9123 | 2,12E-03 |
| Hk3 | -2,3404 | 2,12E-03 |
| Mier1 | -1,2724 | 2,14E-03 |
| Zmpste24 | -1,5938 | 2,14E-03 |
| Ccnd2 | -3,1196 | 2,18E-03 |
| Gm11808 | 1,8533 | 2,17E-03 |
| Runx1t1 | -3,2719 | 2,16E-03 |
| Yaf2 | -1,3960 | 2,18E-03 |
| Ociad2 | -1,4785 | 2,19E-03 |
| Gm22676 | -3,2399 | 2,25E-03 |
| Junb | 1,5872 | 2,29E-03 |
| Ndufb7 | 1,3747 | 2,28E-03 |
| Rbfox3 | 3,8793 | 2,27E-03 |
| Snord89 | -2,2626 | 2,29E-03 |
| Ppil2 | -1,6347 | 2,33E-03 |
| Cxcl12 | -2,5374 | 2,38E-03 |
| Abcg3 | -3,2148 | 2,44E-03 |
| Ptgds | 2,1821 | 2,47E-03 |
| Cdh11 | -1,9273 | 2,49E-03 |
| Pf4 | -2,8583 | 2,49E-03 |
| Dusp7 | 1,4585 | 2,53E-03 |
| Hs3st1 | 1,3737 | 2,56E-03 |
| Rny3 | -1,8623 | 2,58E-03 |
| Vcan | -2,9528 | 2,62E-03 |
| Abcd2 | -3,1484 | 2,63E-03 |
| Glipr1 | -2,6279 | 2,71E-03 |
| Gm25291 | -2,7545 | 2,72E-03 |
| n-R5s138 | -2,9789 | 2,69E-03 |
| Plcb4 | -1,9973 | 2,71E-03 |
| Tiparp | -1,4747 | 2,70E-03 |
| Snhg1 | -1,3827 | 2,73E-03 |
| Gm25687 | 3,3042 | 2,78E-03 |
| Rps28 | 2,0423 | 2,77E-03 |
| Gm22179 | 2,2244 | 2,80E-03 |
| Gm22370 | -2,6531 | 2,82E-03 |
| Gm23969 | -2,4722 | 2,85E-03 |
| Slc11a1 | -1,6077 | 2,87E-03 |
| Slc9a3r1 | 1,4929 | 2,87E-03 |
| Ccr2 | -2,9367 | 2,91E-03 |
| Phex | -3,5322 | 2,91E-03 |
| Lyz2 | -2,8703 | 2,96E-03 |
| Snhg4 | -1,6429 | 2,97E-03 |
| Wfdc2 | 3,1780 | 2,95E-03 |
| Cldn7 | 1,7627 | 3,03E-03 |
| Terc | 4,1804 | 3,04E-03 |
| Wnt9a | -2,8881 | 3,04E-03 |
| Camk1d | -2,4953 | 3,10E-03 |
| Adam23 | -3,4683 | 3,11E-03 |
| Gm23849 | -1,0900 | 3,17E-03 |
| Nedd8 | 1,4597 | 3,17E-03 |
| Pde3b | -2,1286 | 3,16E-03 |
| Angptl4 | -2,0237 | 3,20E-03 |
| RP23-463E8,1 | 1,3620 | 3,22E-03 |
| Slc16a7 | -2,6629 | 3,23E-03 |
| Erc2 | -8,2076 | 3,25E-03 |
| Fstl1 | -2,3578 | 3,26E-03 |
| Zbtb1 | -1,6274 | 3,26E-03 |
| Rab17 | 1,5803 | 3,28E-03 |
| Ppap2a | -2,5139 | 3,42E-03 |
| Cthrc1 | -3,2566 | 3,44E-03 |
| Gm23262 | -3,6413 | 3,50E-03 |
| Lrp10 | 1,3553 | 3,48E-03 |
| Pon3 | -2,6999 | 3,50E-03 |
| Ptgis | -5,6434 | 3,50E-03 |
| Carns1 | 3,3910 | 3,52E-03 |
| Snora68 | -3,0229 | 3,54E-03 |
| Rcc1 | -1,4266 | 3,56E-03 |
| Cyp2d10 | 2,2132 | 3,64E-03 |
| Evi5 | -1,1467 | 3,68E-03 |
| Gins4 | 1,5157 | 3,70E-03 |
| Lyz1 | -4,0675 | 3,61E-03 |
| Myl12b | -1,3676 | 3,61E-03 |
| Nfasc | -2,2558 | 3,69E-03 |
| Rnu2-10 | -1,0787 | 3,69E-03 |
| Serpinb6a | -1,3574 | 3,66E-03 |
| Snord100 | -2,7385 | 3,69E-03 |
| Ttll10 | 1,9514 | 3,62E-03 |
| Wwp1 | -1,4253 | 3,67E-03 |
| Scand1 | 4,2040 | 3,72E-03 |
| Tnxb | -3,5381 | 3,72E-03 |
| RP23-26I10,4 | -4,0846 | 3,77E-03 |
| Ddr2 | -2,1971 | 3,80E-03 |
| n-R5s104 | -3,0223 | 3,80E-03 |
| Dagla | -2,2377 | 3,91E-03 |
| Dbt | -2,4102 | 3,90E-03 |
| Gm22579 | -2,4390 | 3,89E-03 |
| Mfsd6l | 3,5244 | 3,90E-03 |
| n-R5s110 | -2,8099 | 3,90E-03 |
| RP24-282D16,11 | -3,2474 | 3,92E-03 |
| Steap2 | -3,6787 | 3,88E-03 |
| Use1 | 1,5693 | 3,85E-03 |
| Zfp9 | -2,2531 | 3,84E-03 |
| Cpsf6 | -1,0965 | 3,95E-03 |
| Dnajc21 | -1,3333 | 4,05E-03 |
| Fhl1 | -2,7997 | 4,07E-03 |
| Gm22634 | 1,8141 | 4,08E-03 |
| Gm23153 | 2,8619 | 4,08E-03 |
| Gm9843 | 1,5950 | 4,09E-03 |
| Rho | -4,5829 | 4,05E-03 |
| Sec61b | -1,5069 | 4,05E-03 |
| Snord14a | -2,4293 | 4,12E-03 |
| Pdpn | -2,7389 | 4,14E-03 |
| Gm25106 | -2,3182 | 4,23E-03 |
| Uggt2 | -2,0429 | 4,23E-03 |
| Kctd12 | -1,6982 | 4,27E-03 |
| Gm5778 | -1,3137 | 4,34E-03 |
| Tmem123 | -2,8136 | 4,34E-03 |
| Xpo1 | -1,2288 | 4,33E-03 |
| Gm24305 | 1,8801 | 4,37E-03 |
| Abhd11 | 1,4810 | 4,39E-03 |
| Ghr | -1,1473 | 4,50E-03 |
| Slc38a7 | 1,3663 | 4,49E-03 |
| Fgf11 | -2,2883 | 4,55E-03 |
| H60b | -3,4208 | 4,55E-03 |
| Prkar2b | -1,2434 | 4,55E-03 |
| Mirlet7f-2 | -3,2427 | 4,74E-03 |
| Gm24494 | -2,3405 | 4,83E-03 |
| Gm25018 | -2,8932 | 4,84E-03 |
| Nox4 | -2,4956 | 4,84E-03 |
| Rpl38 | 1,3987 | 4,82E-03 |
| Zfp120 | -1,3478 | 4,86E-03 |
| Bst2 | 1,4175 | 4,88E-03 |
| Serpinb6b | -2,9166 | 4,93E-03 |
| Snord35b | -2,3911 | 4,93E-03 |
| H2-Q4 | 1,2862 | 4,97E-03 |
| Nupr1 | 1,3026 | 4,97E-03 |
| Srpx | -2,8910 | 4,95E-03 |
| Rbms3 | -1,9688 | 4,99E-03 |
| Nlrp1b | -2,2085 | 5,07E-03 |
| Cdo1 | -3,4463 | 5,14E-03 |
| Decr1 | -3,0756 | 5,13E-03 |
| Gm13368 | -2,3695 | 5,12E-03 |
| Gm22442 | -2,2126 | 5,12E-03 |
| Cxcr6 | -3,5372 | 5,19E-03 |
| Ckm | -5,7599 | 5,25E-03 |
| Nfkbil1 | 1,6041 | 5,25E-03 |
| Grb7 | 1,4162 | 5,28E-03 |
| Fam187b | 2,0069 | 5,33E-03 |
| Fam198b | -2,1245 | 5,31E-03 |
| Gm22456 | 2,5352 | 5,31E-03 |
| Ptplad2 | -2,4870 | 5,33E-03 |
| Tmem9 | 1,2265 | 5,40E-03 |
| Polr2c | -1,1520 | 5,49E-03 |
| Hook2 | 1,3660 | 5,56E-03 |
| Zfp275 | -2,4034 | 5,60E-03 |
| Calcrl | -1,5621 | 5,63E-03 |
| Gm24950 | -1,0294 | 5,70E-03 |
| Gm2830 | 2,0552 | 5,71E-03 |
| RP24-370G18,1 | -2,8901 | 5,82E-03 |
| Amd1 | -1,4635 | 5,87E-03 |
| Gm13339 | -2,4756 | 5,88E-03 |
| Gm26461 | 2,6750 | 5,89E-03 |
| Nuak1 | -1,8237 | 5,85E-03 |
| Slc1a3 | -3,5219 | 5,98E-03 |
| Slc26a9 | 4,0877 | 6,05E-03 |
| Lrrc17 | -3,0573 | 6,12E-03 |
| n-R5s128 | -3,2206 | 6,12E-03 |
| Snord65 | -2,0148 | 6,14E-03 |
| Fam32a | 1,1704 | 6,18E-03 |
| Tnc | -1,5699 | 6,17E-03 |
| n-R5s100 | -3,6466 | 6,22E-03 |
| Snord91a | -2,3215 | 6,24E-03 |
| Zeb2 | -2,0197 | 6,38E-03 |
| Cdkn2aip | -1,5845 | 6,44E-03 |
| n-R5s123 | -2,9210 | 6,48E-03 |
| Tbxas1 | -2,4713 | 6,47E-03 |
| Gm22767 | -2,5284 | 6,50E-03 |
| Rab25 | 1,3925 | 6,51E-03 |
| Tmem180 | 1,8328 | 6,60E-03 |
| Tll1 | -2,1516 | 6,66E-03 |
| Cox7a2l | -1,1629 | 6,73E-03 |
| Ube2v2 | -1,5789 | 6,81E-03 |
| Abcc5 | -2,1766 | 6,90E-03 |
| Igkv4-79 | -8,0039 | 6,90E-03 |
| Lilrb4 | -1,9114 | 6,91E-03 |
| Leprel2 | -2,2349 | 6,95E-03 |
| Ankrd1 | -3,1936 | 6,97E-03 |
| Akirin1 | -1,4938 | 7,00E-03 |
| Mir6905 | -5,4020 | 7,04E-03 |
| Nr1h2 | 1,0983 | 7,12E-03 |
| Serpina3g | -1,6723 | 7,11E-03 |
| Mrpl52 | 2,0151 | 7,17E-03 |
| Pgr | -4,4771 | 7,17E-03 |
| Fam193b | 1,1933 | 7,21E-03 |
| A530032D15Rik | -2,5587 | 7,26E-03 |
| Nol4 | -7,8189 | 7,25E-03 |
| Olr1 | -4,8077 | 7,29E-03 |
| Slc37a2 | -1,6486 | 7,29E-03 |
| Acp5 | 1,2384 | 7,34E-03 |
| Arrb2 | -1,5772 | 7,42E-03 |
| Arsi | -2,8509 | 7,42E-03 |
| Eif1a | -1,1529 | 7,40E-03 |
| Gm11964 | -2,5382 | 7,36E-03 |
| Gm26316 | -1,3176 | 7,36E-03 |
| Itga4 | -1,8986 | 7,39E-03 |
| Sdhaf1 | 1,8613 | 7,42E-03 |
| BC021767 | 3,5238 | 7,49E-03 |
| Rab24 | 1,1370 | 7,50E-03 |
| Pear1 | -2,3672 | 7,53E-03 |
| Bcl6 | 1,1610 | 7,64E-03 |
| Peli3 | 2,3078 | 7,63E-03 |
| Gm26191 | 1,9480 | 7,68E-03 |
| Tnnc2 | -7,7898 | 7,68E-03 |
| Cep55 | -1,3123 | 7,71E-03 |
| Gm9796 | -6,2200 | 7,70E-03 |
| Bglap3 | 1,9225 | 7,79E-03 |
| Gm24336 | -1,9907 | 7,80E-03 |
| Rps2 | 1,3303 | 7,77E-03 |
| 1700049G17Rik | -3,0949 | 7,89E-03 |
| Gng7 | 1,1662 | 7,87E-03 |
| n-R5s142 | -3,0226 | 7,86E-03 |
| Slit3 | -1,6981 | 7,86E-03 |
| Snord53 | -2,7428 | 7,84E-03 |
| Snord23 | -2,6107 | 7,93E-03 |
| BC005537 | -1,1002 | 8,04E-03 |
| Kcnn4 | 1,1599 | 8,01E-03 |
| Peg10 | -4,7564 | 8,03E-03 |
| Slc8b1 | 1,2146 | 8,03E-03 |
| Gm24539 | -2,5946 | 8,07E-03 |
| Mirlet7c-1 | -2,5046 | 8,13E-03 |
| Pnpla3 | -4,1071 | 8,14E-03 |
| Lama5 | 0,9872 | 8,19E-03 |
| Usf1 | 1,3645 | 8,17E-03 |
| Ggnbp1 | 1,7933 | 8,24E-03 |
| Trmt61a | -1,4562 | 8,25E-03 |
| Tgfbr1 | -1,2728 | 8,29E-03 |
| Dhdh | -2,9360 | 8,35E-03 |
| Fas | -2,7997 | 8,36E-03 |
| Zfhx2 | 1,3210 | 8,35E-03 |
| 1110038B12Rik | -2,0347 | 8,39E-03 |
| C1qtnf3 | -1,8121 | 8,51E-03 |
| Ccdc149 | 1,5101 | 8,49E-03 |
| Ly6g6e | 1,7550 | 8,47E-03 |
| Rnu3a | -1,4598 | 8,55E-03 |
| Wtip | 1,3378 | 8,59E-03 |
| Gm25206 | -2,7703 | 8,65E-03 |
| Piga | -1,3073 | 8,68E-03 |
| Fam49b | -1,1969 | 8,70E-03 |
| Ssr2 | 1,2004 | 8,73E-03 |
| Gm22806 | -2,1599 | 8,85E-03 |
| Lars2 | 1,2650 | 8,79E-03 |
| Lyve1 | -4,2722 | 8,86E-03 |
| Mpp2 | 2,8131 | 8,90E-03 |
| Nek7 | -1,1481 | 8,77E-03 |
| Siglecg | -4,1418 | 8,85E-03 |
| Srsf10 | -1,2594 | 8,86E-03 |
| Tgm2 | 0,9885 | 8,89E-03 |
| Zfp825 | -2,6573 | 8,88E-03 |
| Figf | -3,9281 | 8,95E-03 |
| n-R5s146 | -2,7107 | 8,93E-03 |
| Fbxo33 | -1,2661 | 9,01E-03 |
| Gm24727 | -1,4846 | 9,01E-03 |
| Rnu73b | -2,3727 | 8,98E-03 |
| C4b | -2,0617 | 9,04E-03 |
| Pou2af1 | -4,0154 | 9,14E-03 |
| Gm25129 | -7,8516 | 9,16E-03 |
| Fkbp10 | -2,4653 | 9,22E-03 |
| Mxra7 | -2,5781 | 9,20E-03 |
| Sepp1 | -1,0746 | 9,18E-03 |
| Tln1 | -0,9845 | 9,23E-03 |
| Lamb2 | 1,3076 | 9,28E-03 |
| Rian | -1,9116 | 9,36E-03 |
| As3mt | -2,8391 | 9,43E-03 |
| Entpd1 | -1,9968 | 9,42E-03 |
| Gm27264 | -2,8630 | 9,43E-03 |
| Slc35a1 | -1,2837 | 9,46E-03 |
| Gm28437 | -1,5953 | 9,50E-03 |
| Atp13a3 | -1,0329 | 9,60E-03 |
| Dock6 | 1,1245 | 9,60E-03 |
| Krt79 | 3,4742 | 9,62E-03 |
| Fxr1 | -1,0181 | 9,68E-03 |
| Hist2h3b | 2,1583 | 9,67E-03 |
| 1110002L01Rik | -1,3407 | 9,88E-03 |
| 1700037H04Rik | 1,3908 | 9,94E-03 |
| 2700099C18Rik | -1,6728 | 9,86E-03 |
| Col20a1 | 1,4196 | 1,01E-02 |
| Gm25492 | -2,1028 | 1,00E-02 |
| Gm25788 | -2,2149 | 9,97E-03 |
| Gstt3 | 1,3290 | 9,94E-03 |
| Itga7 | -2,2080 | 1,00E-02 |
| Msln | -8,7216 | 1,01E-02 |
| Notch3 | 1,2099 | 1,00E-02 |
| Ogn | -3,5802 | 9,90E-03 |
| Rnase1 | 3,5438 | 1,01E-02 |
| Snx7 | -1,4016 | 1,00E-02 |
| Mylpf | -5,1530 | 1,01E-02 |
| Fads1 | -1,8525 | 1,02E-02 |
| Gfpt2 | -2,5667 | 1,02E-02 |
| Hist1h1c | 1,3729 | 1,02E-02 |
| Nlrc4 | -2,4938 | 1,02E-02 |
| Ckmt1 | 1,3751 | 1,03E-02 |
| Aspn | -2,3052 | 1,04E-02 |
| Ccdc3 | -4,3922 | 1,05E-02 |
| Gm24289 | -2,3065 | 1,05E-02 |
| Ngdn | 1,4120 | 1,04E-02 |
| Slc1a5 | -1,4904 | 1,04E-02 |
| Snord49a | -2,3822 | 1,04E-02 |
| Srpk2 | -1,0426 | 1,04E-02 |
| Wdr45 | 1,2669 | 1,05E-02 |
| Dse | -2,8272 | 1,05E-02 |
| Pvrl3 | -1,3784 | 1,05E-02 |
| Itgb7 | -2,9078 | 1,06E-02 |
| Rps23 | 1,1462 | 1,06E-02 |
| Nr4a1 | 1,3087 | 1,07E-02 |
| Pid1 | -2,1360 | 1,09E-02 |
| Ppap2c | 1,1580 | 1,09E-02 |
| 2310045N01Rik | 1,3833 | 1,10E-02 |
| Abcc1 | -1,9404 | 1,10E-02 |
| Adck3 | 1,3392 | 1,10E-02 |
| Serpina3h | -2,0303 | 1,10E-02 |
| Gm11249 | 1,5696 | 1,11E-02 |
| Ccdc85b | 2,4083 | 1,12E-02 |
| Isoc2a | 1,6495 | 1,11E-02 |
| Kpna1 | -1,1246 | 1,12E-02 |
| Ssu72 | -1,2131 | 1,12E-02 |
| Gm22109 | -2,8955 | 1,12E-02 |
| Osr2 | -4,8024 | 1,13E-02 |
| Rgs10 | -1,7545 | 1,13E-02 |
| Rhoa | -1,0851 | 1,13E-02 |
| Kng2 | 2,5597 | 1,14E-02 |
| Pyroxd2 | 1,1264 | 1,15E-02 |
| Slc16a10 | -2,3608 | 1,16E-02 |
| Col6a1 | -2,3282 | 1,18E-02 |
| Gm22291 | -2,5813 | 1,18E-02 |
| Hnrnpu | -1,4609 | 1,18E-02 |
| Spta1 | -2,8305 | 1,18E-02 |
| Zfp131 | -1,4448 | 1,18E-02 |
| Pbx3 | -1,3569 | 1,19E-02 |
| P2ry10 | -3,4067 | 1,20E-02 |
| Gm7221 | -3,6036 | 1,20E-02 |
| 2810008D09Rik | 1,4992 | 1,21E-02 |
| Acss3 | -4,1433 | 1,23E-02 |
| Anpep | 1,6015 | 1,22E-02 |
| Ereg | -4,5346 | 1,20E-02 |
| Gm22739 | 2,1230 | 1,21E-02 |
| Gm22980 | -2,3622 | 1,23E-02 |
| Igkv1-117 | -4,3998 | 1,22E-02 |
| mt-Atp8 | -2,0333 | 1,23E-02 |
| mt-Nd2 | -1,4029 | 1,22E-02 |
| n-R5s107 | -2,9111 | 1,23E-02 |
| Orc4 | -1,3668 | 1,21E-02 |
| Rab5a | -1,2480 | 1,22E-02 |
| Ripk1 | -1,1613 | 1,23E-02 |
| Tmc6 | 1,0573 | 1,23E-02 |
| Tmem256 | 1,6681 | 1,23E-02 |
| Mirlet7a-2 | -2,6272 | 1,23E-02 |
| Dnm3os | -2,1984 | 1,24E-02 |
| Krt19 | -2,7045 | 1,24E-02 |
| Shf | 1,2066 | 1,24E-02 |
| Cml2 | -1,6721 | 1,25E-02 |
| Crip2 | 0,9727 | 1,25E-02 |
| Gm23973 | 2,9375 | 1,25E-02 |
| Orc2 | -1,1500 | 1,25E-02 |
| Zfp316 | 1,2055 | 1,25E-02 |
| Rnase4 | -2,1898 | 1,26E-02 |
| Rpl10 | 0,9590 | 1,26E-02 |
| Hist1h2bl | -1,5823 | 1,26E-02 |
| Amy1 | -3,5714 | 1,26E-02 |
| Ces1d | -5,1437 | 1,27E-02 |
| Kctd17 | -2,4849 | 1,27E-02 |
| Pla2g4d | 4,1965 | 1,27E-02 |
| Thsd7a | -2,1756 | 1,27E-02 |
| Clec4e | -3,3161 | 1,28E-02 |
| Cyb561 | 1,3274 | 1,28E-02 |
| Fam98a | -1,1480 | 1,29E-02 |
| Gm14236 | 2,9242 | 1,29E-02 |
| Gm24407 | -1,8538 | 1,29E-02 |
| Rpl15 | 1,0092 | 1,28E-02 |
| Ccl9 | -2,7195 | 1,29E-02 |
| D7Ertd443e | 1,5499 | 1,29E-02 |
| Gm11878 | -1,5931 | 1,30E-02 |
| Hspa12a | -3,2113 | 1,30E-02 |
| Plxnb1 | 1,4490 | 1,30E-02 |
| Snord37 | -2,4050 | 1,29E-02 |
| Tmem33 | -1,0930 | 1,30E-02 |
| Ap1s2 | -1,4127 | 1,31E-02 |
| Cldn15 | -1,9605 | 1,31E-02 |
| Emr1 | -1,7743 | 1,31E-02 |
| Mylk | -1,2427 | 1,34E-02 |
| Timp2 | -1,7035 | 1,34E-02 |
| Rnasel | -1,4694 | 1,35E-02 |
| Eif3j1 | -1,4234 | 1,36E-02 |
| Fam19a2 | -5,5630 | 1,36E-02 |
| Gm15564 | 2,0960 | 1,37E-02 |
| BC034090 | -1,4857 | 1,38E-02 |
| Cript | -1,2779 | 1,39E-02 |
| Emc10 | 1,1921 | 1,40E-02 |
| Glrx3 | -1,4155 | 1,40E-02 |
| Gm22984 | -2,7016 | 1,39E-02 |
| Gypc | -3,5122 | 1,40E-02 |
| Ndfip1 | -1,2260 | 1,39E-02 |
| Spint2 | 1,3714 | 1,40E-02 |
| Sptssa | -1,0639 | 1,39E-02 |
| Dnajb4 | -1,1936 | 1,41E-02 |
| Ptma | -1,1326 | 1,41E-02 |
| Gabra3 | -6,4376 | 1,42E-02 |
| Slc22a18 | 2,7760 | 1,42E-02 |
| Gm15884 | 2,9071 | 1,45E-02 |
| Mir3473g | -3,2730 | 1,45E-02 |
| H2-Q1 | 1,5194 | 1,46E-02 |
| Rpl6 | 0,9844 | 1,48E-02 |
| Ncoa7 | -1,1947 | 1,48E-02 |
| Tlr4 | -1,1272 | 1,49E-02 |
| Tmed5 | -1,1164 | 1,48E-02 |
| Adprhl2 | -1,2328 | 1,49E-02 |
| Ccdc50 | -0,9854 | 1,50E-02 |
| Ctcf | -1,3575 | 1,50E-02 |
| Prune2 | -2,1539 | 1,50E-02 |
| Mpnd | 1,3687 | 1,51E-02 |
| Rab8b | -1,1607 | 1,52E-02 |
| Pi15 | -2,8406 | 1,52E-02 |
| Snora33 | -2,2157 | 1,52E-02 |
| Zmiz1os1 | 2,4540 | 1,52E-02 |
| Lbhd1 | -1,8006 | 1,53E-02 |
| Auh | 1,1770 | 1,55E-02 |
| Klf5 | 1,1994 | 1,55E-02 |
| Maf | -2,2803 | 1,55E-02 |
| Tnnt3 | -5,2921 | 1,54E-02 |
| Trmt10a | -1,3936 | 1,55E-02 |
| Dolk | 1,2817 | 1,55E-02 |
| Rph3al | 2,3249 | 1,55E-02 |
| Atp5k | 1,6778 | 1,58E-02 |
| Bnc1 | -3,0147 | 1,56E-02 |
| Btaf1 | -0,9618 | 1,59E-02 |
| Cryab | 1,2149 | 1,59E-02 |
| Ets2 | 1,0220 | 1,57E-02 |
| Gm23284 | -2,4940 | 1,56E-02 |
| Gm24059 | -5,5523 | 1,58E-02 |
| Gm25704 | 1,9730 | 1,58E-02 |
| H2-Q6 | 1,5277 | 1,57E-02 |
| Matn2 | -1,9503 | 1,59E-02 |
| Rpl17 | 0,9877 | 1,58E-02 |
| Slco2b1 | -2,1566 | 1,57E-02 |
| Tpm2 | -2,5541 | 1,58E-02 |
| Zcchc9 | -1,5243 | 1,58E-02 |
| 5330416C01Rik | -2,1431 | 1,61E-02 |
| Col3a1 | -1,8117 | 1,61E-02 |
| Dock4 | -0,9795 | 1,61E-02 |
| Fdx1l | 2,0116 | 1,60E-02 |
| Gm26572 | 2,8063 | 1,61E-02 |
| n-R5s141 | -2,4982 | 1,60E-02 |
| Nrp1 | -1,9173 | 1,60E-02 |
| Nxpe5 | -3,1905 | 1,60E-02 |
| Slc25a32 | -1,2491 | 1,62E-02 |
| Mir374b | -3,2151 | 1,63E-02 |
| Fancd2 | -1,2767 | 1,63E-02 |
| Gm8116 | -2,2081 | 1,63E-02 |
| Saysd1 | 1,6698 | 1,64E-02 |
| Snora24 | -1,9531 | 1,63E-02 |
| A530016L24Rik | -3,3841 | 1,68E-02 |
| Abcc4 | -2,5747 | 1,71E-02 |
| Angpt1 | -2,0872 | 1,69E-02 |
| Arxes2 | -2,9715 | 1,69E-02 |
| Clcn2 | 1,2811 | 1,66E-02 |
| Col6a3 | -1,9915 | 1,71E-02 |
| Dcn | -2,3437 | 1,69E-02 |
| Epb4,1l3 | -2,9699 | 1,71E-02 |
| Fosl1 | -3,8919 | 1,69E-02 |
| Gli3 | -2,0569 | 1,68E-02 |
| Lama3 | -2,2435 | 1,70E-02 |
| Lrrc29 | 3,5073 | 1,70E-02 |
| Lzts2 | 1,2946 | 1,70E-02 |
| Mir652 | -3,2479 | 1,71E-02 |
| Myo5a | -1,2322 | 1,70E-02 |
| Ncs1 | -3,5220 | 1,67E-02 |
| Pccb | 1,1315 | 1,67E-02 |
| Rgs19 | -1,2356 | 1,67E-02 |
| Rrp8 | -1,1231 | 1,68E-02 |
| Selt | -0,9885 | 1,67E-02 |
| Sh2b1 | 1,1082 | 1,67E-02 |
| Slc39a10 | -1,1113 | 1,71E-02 |
| Tmem200a | -4,5220 | 1,66E-02 |
| Tst | 1,2263 | 1,69E-02 |
| Ugcg | -1,7177 | 1,70E-02 |
| Zfhx4 | -2,1686 | 1,70E-02 |
| Zfp451 | -0,9355 | 1,65E-02 |
| Zfp943 | -1,5741 | 1,71E-02 |
| Igfbp6 | -2,5439 | 1,72E-02 |
| Egr1 | 1,1170 | 1,74E-02 |
| Fads6 | 1,8836 | 1,73E-02 |
| Fam89a | 1,6297 | 1,74E-02 |
| Ms4a14 | -2,2225 | 1,73E-02 |
| Pvrl2 | 1,1376 | 1,74E-02 |
| Rtkn2 | -2,6211 | 1,74E-02 |
| Ccnc | -1,3072 | 1,75E-02 |
| Fam111a | -2,0895 | 1,76E-02 |
| Hagh | 1,1700 | 1,75E-02 |
| Itih5 | -2,0007 | 1,76E-02 |
| Nqo1 | -5,5486 | 1,76E-02 |
| Paip1 | -1,0691 | 1,76E-02 |
| Slc30a4 | -0,9808 | 1,75E-02 |
| Dennd2a | -2,8987 | 1,76E-02 |
| Tg | -2,1304 | 1,77E-02 |
| Arl14ep | -1,2129 | 1,77E-02 |
| Hes6 | 1,5370 | 1,77E-02 |
| 6530402F18Rik | -1,9556 | 1,79E-02 |
| Cul2 | -1,0372 | 1,79E-02 |
| Dld | -1,2035 | 1,79E-02 |
| Lrp5 | 1,0013 | 1,79E-02 |
| Map3k7 | -0,9956 | 1,80E-02 |
| Mir744 | -2,7177 | 1,80E-02 |
| Spin2c | 2,2374 | 1,80E-02 |
| Adcy8 | 2,2722 | 1,82E-02 |
| B430306N03Rik | -2,1606 | 1,83E-02 |
| Cand1 | -1,8249 | 1,82E-02 |
| Cnksr1 | 1,4728 | 1,83E-02 |
| Gatad2a | 1,0228 | 1,80E-02 |
| Gm10263 | 2,4341 | 1,83E-02 |
| Gm13611 | -1,4480 | 1,83E-02 |
| Inpp5a | 1,3579 | 1,83E-02 |
| Mapk8ip1 | 1,0693 | 1,83E-02 |
| Meis2 | -1,9576 | 1,81E-02 |
| Mir7669 | -4,8432 | 1,82E-02 |
| Rbfa | 1,3678 | 1,83E-02 |
| Rnf5 | 1,5878 | 1,81E-02 |
| Zfp948 | -1,1890 | 1,81E-02 |
| Dctn6 | 2,1953 | 1,84E-02 |
| Cdc42ep4 | 1,2176 | 1,85E-02 |
| Rps19 | 1,0375 | 1,86E-02 |
| Fibin | -3,1467 | 1,86E-02 |
| Rps2-ps13 | 1,3082 | 1,86E-02 |
| Zfp952 | -1,4596 | 1,87E-02 |
| Gm22581 | -2,1257 | 1,88E-02 |
| Etfb | 1,7880 | 1,90E-02 |
| Flcn | 1,2461 | 1,90E-02 |
| Galt | 1,4429 | 1,90E-02 |
| Gm22865 | 3,1038 | 1,90E-02 |
| Reck | 1,5496 | 1,91E-02 |
| Ror1 | -1,8897 | 1,91E-02 |
| Slc7a6 | -2,2708 | 1,91E-02 |
| Snord92 | -2,2499 | 1,91E-02 |
| Zyx | -0,9652 | 1,90E-02 |
| Ampd3 | -3,0594 | 1,92E-02 |
| S100a11 | -1,6255 | 1,92E-02 |
| Acbd4 | 1,1184 | 1,93E-02 |
| Cmklr1 | -1,9416 | 1,93E-02 |
| Wt1 | -2,0689 | 1,93E-02 |
| Gng11 | -1,7284 | 1,94E-02 |
| Car3 | -6,9715 | 1,94E-02 |
| Trim59 | -1,3306 | 1,94E-02 |
| Ddx39b | 0,9237 | 1,95E-02 |
| Csrp2 | -1,5935 | 1,96E-02 |
| Ntrk2 | -4,1142 | 1,96E-02 |
| Pygm | -2,4895 | 1,96E-02 |
| Prdm1 | -1,5405 | 1,97E-02 |
| Gm24253 | -2,1030 | 1,97E-02 |
| Naa25 | -0,9291 | 1,97E-02 |
| Gm6311 | 1,3327 | 1,98E-02 |
| Gm7808 | 2,4385 | 1,98E-02 |
| Tppp3 | -1,7401 | 1,99E-02 |
| Cx3cr1 | -1,7610 | 2,00E-02 |
| Gbp8 | -2,0678 | 2,00E-02 |
| Sfpq | -0,8821 | 2,01E-02 |
| Gm4462 | 2,9703 | 2,01E-02 |
| Reep1 | -3,2679 | 2,03E-02 |
| Cited2 | 1,1177 | 2,03E-02 |
| Pcdh7 | -1,6010 | 2,03E-02 |
| Gm26852 | 2,2900 | 2,04E-02 |
| Neb | -3,5996 | 2,04E-02 |
| Spcs3 | -1,0477 | 2,04E-02 |
| Tmem203 | 3,1532 | 2,04E-02 |
| Id4 | 3,5003 | 2,05E-02 |
| Snord110 | -1,9495 | 2,05E-02 |
| Hsp90aa1 | -1,3794 | 2,06E-02 |
| Tmem110 | 1,2416 | 2,06E-02 |
| Ccar2 | 1,1827 | 2,07E-02 |
| Clec4n | -3,2497 | 2,08E-02 |
| Ephb4 | 0,8932 | 2,10E-02 |
| Gm14827 | -3,9535 | 2,10E-02 |
| Gm29113 | -4,3405 | 2,10E-02 |
| Lrrc49 | -1,3967 | 2,09E-02 |
| Mier3 | -1,0323 | 2,10E-02 |
| Rpl41 | 0,9761 | 2,10E-02 |
| Slc25a3 | 1,0994 | 2,09E-02 |
| Snord45b | -1,8474 | 2,10E-02 |
| Ufm1 | -1,1524 | 2,09E-02 |
| Ska2 | -1,2389 | 2,12E-02 |
| Elfn1 | -5,3007 | 2,14E-02 |
| Gm26444 | 1,5485 | 2,13E-02 |
| Ifi27 | 0,9606 | 2,14E-02 |
| March1 | -1,4778 | 2,13E-02 |
| Megf9 | -1,3550 | 2,14E-02 |
| Lfng | -1,9244 | 2,15E-02 |
| Car2 | -1,0304 | 2,15E-02 |
| Pten | -0,9317 | 2,16E-02 |
| Gm24888 | -1,8803 | 2,18E-02 |
| Ptrf | -2,0547 | 2,18E-02 |
| Tmem263 | -1,1644 | 2,17E-02 |
| Tnfaip8l2 | -1,6354 | 2,17E-02 |
| Wfdc3 | 2,1717 | 2,17E-02 |
| Hsdl1 | 1,3040 | 2,18E-02 |
| Krt8 | 0,9772 | 2,19E-02 |
| Ctxn1 | 2,7585 | 2,20E-02 |
| Gm22405 | 1,5883 | 2,19E-02 |
| Gm24812 | 3,0221 | 2,20E-02 |
| Liph | 1,1999 | 2,20E-02 |
| Mtmr9 | 1,1148 | 2,20E-02 |
| Tnfsf8 | -2,2134 | 2,20E-02 |
| Fcho2 | -1,3076 | 2,21E-02 |
| Il6st | -1,4961 | 2,22E-02 |
| Lims2 | -2,7882 | 2,22E-02 |
| Rnaseh2a | 1,0340 | 2,22E-02 |
| Zmat3 | -1,1415 | 2,22E-02 |
| Gpx7 | -2,8388 | 2,22E-02 |
| Tmem125 | 2,2477 | 2,23E-02 |
| Gnai1 | -4,5816 | 2,24E-02 |
| Idh3b | 1,3461 | 2,23E-02 |
| Inpp4b | -1,5639 | 2,24E-02 |
| Lmntd2 | 1,5583 | 2,23E-02 |
| Nbeal1 | -1,1667 | 2,24E-02 |
| Prodh | -2,1032 | 2,24E-02 |
| AF357399 | -1,5597 | 2,26E-02 |
| Cstb | 1,0143 | 2,26E-02 |
| Etohi1 | -1,7393 | 2,26E-02 |
| Gm12346 | -1,4005 | 2,26E-02 |
| Cldn3 | 1,1098 | 2,29E-02 |
| Gm11127 | 2,5837 | 2,27E-02 |
| Gm21887 | 1,2658 | 2,29E-02 |
| Gm22706 | -3,3910 | 2,29E-02 |
| Hipk2 | -0,9315 | 2,29E-02 |
| Ighm | -1,3323 | 2,29E-02 |
| Pim3 | 1,7690 | 2,28E-02 |
| Rbm15b | -0,9394 | 2,29E-02 |
| Sdad1 | -0,9955 | 2,29E-02 |
| Smim15 | -1,1124 | 2,29E-02 |
| Tmem56 | -1,5691 | 2,29E-02 |
| Adamtsl3 | -2,1703 | 2,31E-02 |
| Clip3 | -3,0767 | 2,31E-02 |
| B4galt5 | -2,5515 | 2,33E-02 |
| Chmp1a | 0,9154 | 2,33E-02 |
| Dock2 | -1,4858 | 2,33E-02 |
| Fbln2 | -2,3680 | 2,32E-02 |
| Lyzl4 | 1,6062 | 2,32E-02 |
| Mtpn | -0,9233 | 2,32E-02 |
| 9330182L06Rik | 2,9949 | 2,34E-02 |
| Chst11 | -1,0641 | 2,34E-02 |
| Ezr | 0,9369 | 2,34E-02 |
| P4ha3 | -2,7699 | 2,34E-02 |
| Sbsn | 1,5223 | 2,34E-02 |
| Slit2 | -1,8157 | 2,34E-02 |
| Phb2 | 1,0114 | 2,36E-02 |
| RP24-429G21,3 | -2,9261 | 2,36E-02 |
| Zfp748 | -1,3253 | 2,38E-02 |
| 4930448N21Rik | 1,5565 | 2,38E-02 |
| Gm8226 | 1,5659 | 2,39E-02 |
| Trhde | -5,4008 | 2,40E-02 |
| Chac2 | -2,0173 | 2,41E-02 |
| Rassf10 | 1,1987 | 2,41E-02 |
| Dyrk1b | 1,1823 | 2,42E-02 |
| Lage3 | 1,2723 | 2,42E-02 |
| Coasy | 1,1225 | 2,42E-02 |
| Slfn5 | -1,3322 | 2,43E-02 |
| Pofut1 | -0,9497 | 2,44E-02 |
| Gm6170 | 1,4753 | 2,44E-02 |
| Cyp1b1 | -2,2326 | 2,48E-02 |
| Grhl2 | 0,8864 | 2,48E-02 |
| Gspt1 | -0,9197 | 2,48E-02 |
| Hemk1 | 1,6949 | 2,49E-02 |
| Hsph1 | -1,8998 | 2,50E-02 |
| Kctd15 | 1,0758 | 2,50E-02 |
| Met | -0,8756 | 2,48E-02 |
| Mlx | -1,0235 | 2,49E-02 |
| n-R5s133 | -3,2172 | 2,46E-02 |
| Nfe2l1 | 0,8930 | 2,50E-02 |
| Pacsin3 | 1,1583 | 2,47E-02 |
| Pde11a | -2,3895 | 2,50E-02 |
| Pyhin1 | -1,9523 | 2,47E-02 |
| Rnf150 | -1,6964 | 2,48E-02 |
| RP24-114E18,7 | -1,3111 | 2,47E-02 |
| Rps2-ps5 | 3,1536 | 2,49E-02 |
| Rps3 | 0,9304 | 2,51E-02 |
| Saraf | 0,8809 | 2,46E-02 |
| Snord8 | -2,3172 | 2,50E-02 |
| Thbs3 | -2,5107 | 2,46E-02 |
| Tmem134 | 1,3792 | 2,45E-02 |
| Acaa1a | 1,0982 | 2,51E-02 |
| Nr2c2ap | 1,3053 | 2,51E-02 |
| Capza2 | -0,9241 | 2,52E-02 |
| Prrc2b | 0,8779 | 2,52E-02 |
| RP24-252K15,3 | 2,3963 | 2,52E-02 |
| Lrmp | -1,7373 | 2,54E-02 |
| Ncam1 | -2,3229 | 2,54E-02 |
| Ppp1r9a | -2,3562 | 2,54E-02 |
| Uba6 | -1,0836 | 2,54E-02 |
| Fbxo4 | -1,3857 | 2,55E-02 |
| Rgs11 | 1,0936 | 2,56E-02 |
| Mctp1 | -2,5966 | 2,57E-02 |
| Lrrc32 | -2,1855 | 2,57E-02 |
| Tacstd2 | 1,1510 | 2,59E-02 |
| Vps18 | 0,9330 | 2,59E-02 |
| Fcrls | -3,1024 | 2,60E-02 |
| Ak5 | -2,2201 | 2,61E-02 |
| Snord4a | -2,1577 | 2,61E-02 |
| Kdelc2 | -1,1221 | 2,62E-02 |
| Mbnl1 | -0,8728 | 2,62E-02 |
| Papln | 1,2361 | 2,62E-02 |
| Stab1 | -2,1342 | 2,62E-02 |
| RP23-242C11,1 | -2,2130 | 2,62E-02 |
| Eif3j2 | -1,2961 | 2,64E-02 |
| Itgb2 | -1,3699 | 2,64E-02 |
| Gm28361 | -2,8491 | 2,65E-02 |
| Gm10925 | -1,4740 | 2,65E-02 |
| Mtdh | -0,9097 | 2,67E-02 |
| Gm5511 | -2,3338 | 2,67E-02 |
| Gm17066 | -2,1405 | 2,68E-02 |
| H2-Q5 | 1,4360 | 2,68E-02 |
| RP23-392C14,2 | -1,1704 | 2,68E-02 |
| Clk1 | -1,4564 | 2,68E-02 |
| Gm24339 | -1,9045 | 2,69E-02 |
| Gm24916 | -1,9298 | 2,69E-02 |
| Zfand2a | 1,1242 | 2,69E-02 |
| Polr2b | -1,0116 | 2,70E-02 |
| Gm24105 | 2,0158 | 2,71E-02 |
| Tmx3 | -0,9703 | 2,71E-02 |
| Lox | -2,2884 | 2,71E-02 |
| Fkbp9 | -0,9691 | 2,72E-02 |
| Hist1h4b | 1,9981 | 2,72E-02 |
| Snord98 | -1,9841 | 2,72E-02 |
| Gm2115 | -4,4059 | 2,73E-02 |
| Stx2 | -1,4878 | 2,73E-02 |
| Gm22362 | -1,8749 | 2,73E-02 |
| Ric8 | 1,0480 | 2,74E-02 |
| Pex5 | 0,8991 | 2,74E-02 |
| Egln2 | 1,1629 | 2,76E-02 |
| Faah | 1,1532 | 2,78E-02 |
| Flad1 | 0,9708 | 2,77E-02 |
| Gm10076 | 1,5181 | 2,76E-02 |
| Gm12925 | -3,2074 | 2,77E-02 |
| Gm14326 | -1,7130 | 2,78E-02 |
| Gm24265 | -1,5746 | 2,79E-02 |
| Klhdc8b | 2,9957 | 2,77E-02 |
| Loh12cr1 | 1,5938 | 2,75E-02 |
| Penk | -2,8801 | 2,77E-02 |
| Rap2c | -1,1037 | 2,76E-02 |
| Snrpf | -1,4536 | 2,78E-02 |
| Zfp935 | -1,7932 | 2,78E-02 |
| Lpar2 | 1,2962 | 2,79E-02 |
| Gm25820 | -2,1471 | 2,79E-02 |
| Naca | 0,8820 | 2,80E-02 |
| 2610002J02Rik | 1,2379 | 2,82E-02 |
| Nhsl2 | -2,4044 | 2,82E-02 |
| Pparg | -2,9414 | 2,82E-02 |
| Zbed5 | -1,4774 | 2,82E-02 |
| Fam49a | -1,7817 | 2,83E-02 |
| Gipc1 | 0,9161 | 2,84E-02 |
| Kif2a | -1,3287 | 2,84E-02 |
| Pax3 | -3,5796 | 2,83E-02 |
| Pinc | -1,7658 | 2,85E-02 |
| Adam10 | -0,9040 | 2,87E-02 |
| Calcoco1 | 0,8963 | 2,87E-02 |
| Megf6 | 1,0087 | 2,87E-02 |
| Ythdf2 | -1,0463 | 2,88E-02 |
| Rps27 | 1,0338 | 2,89E-02 |
| Myo9a | -1,0184 | 2,90E-02 |
| Galnt2 | 2,7107 | 2,90E-02 |
| Skiv2l2 | -0,8929 | 2,90E-02 |
| RP23-379C24,1 | -2,3242 | 2,92E-02 |
| Col6a2 | -1,9463 | 2,94E-02 |
| Pfdn5 | 1,1155 | 2,94E-02 |
| Mapk3 | 0,9340 | 2,95E-02 |
| Dcbld2 | -1,1343 | 2,96E-02 |
| Wdr83 | 1,3767 | 2,96E-02 |
| Hsd11b1 | -1,6967 | 2,97E-02 |
| Naa15 | -1,0268 | 2,98E-02 |
| Tfap2c | 1,1916 | 2,98E-02 |
| Tmem55a | -1,0661 | 2,98E-02 |
| 1110017D15Rik | -3,6699 | 2,99E-02 |
| Elf4 | -1,6466 | 2,99E-02 |
| Snora16a | -1,8322 | 2,99E-02 |
| Acta2 | -1,6563 | 3,00E-02 |
| Aqp5 | 0,8251 | 3,00E-02 |
| Ccr1 | -2,1639 | 3,02E-02 |
| Gm15952 | -2,7381 | 3,02E-02 |
| Gm3608 | -1,1948 | 3,03E-02 |
| Hsf2 | -1,2238 | 3,03E-02 |
| Itga11 | -1,6963 | 3,01E-02 |
| Mllt6 | 0,9063 | 3,03E-02 |
| Rfx8 | -3,2735 | 3,03E-02 |
| Wwc1 | 0,9457 | 3,03E-02 |
| Col15a1 | -1,5762 | 3,04E-02 |
| Hspa1a | 1,9222 | 3,04E-02 |
| Gsn | -1,0689 | 3,05E-02 |
| Dcps | 1,3552 | 3,06E-02 |
| Npl | -2,2575 | 3,06E-02 |
| Slfn1 | -1,8682 | 3,06E-02 |
| Naa10 | 1,2518 | 3,06E-02 |
| Gpr183 | -2,2000 | 3,07E-02 |
| Fau | 1,4870 | 3,09E-02 |
| Dcdc5 | -3,6064 | 3,09E-02 |
| Antxr2 | -2,0906 | 3,10E-02 |
| Gm26591 | -2,3938 | 3,10E-02 |
| Lrrn4cl | -1,9038 | 3,10E-02 |
| Cbx4 | 0,9979 | 3,11E-02 |
| Folh1 | -4,4363 | 3,11E-02 |
| Zfp365 | -4,0284 | 3,12E-02 |
| Gm23751 | -2,0175 | 3,12E-02 |
| Cpm | 1,2869 | 3,13E-02 |
| Myom1 | -1,5742 | 3,14E-02 |
| Mpdz | -0,9575 | 3,14E-02 |
| Tmtc3 | -1,0340 | 3,15E-02 |
| Fpgt | -0,9804 | 3,16E-02 |
| 2410015M20Rik | 1,0454 | 3,17E-02 |
| Dmxl1 | -0,9654 | 3,16E-02 |
| F830016B08Rik | -2,3754 | 3,19E-02 |
| Grem1 | -1,7478 | 3,18E-02 |
| Hdgfrp3 | -2,1672 | 3,17E-02 |
| Litaf | 1,0192 | 3,18E-02 |
| Llph-ps2 | -1,7580 | 3,19E-02 |
| Mdm2 | -1,0693 | 3,18E-02 |
| Nucb2 | -1,1929 | 3,18E-02 |
| Zswim8 | 0,8966 | 3,19E-02 |
| Arhgap27 | 1,0310 | 3,20E-02 |
| Erp44 | -1,1499 | 3,20E-02 |
| Tifa | -1,6351 | 3,20E-02 |
| Dusp14 | -1,1696 | 3,21E-02 |
| Gpr135 | 2,0777 | 3,21E-02 |
| Gsdmd | -1,9427 | 3,21E-02 |
| 4631405K08Rik | 3,3461 | 3,22E-02 |
| Rnf138 | -1,5134 | 3,22E-02 |
| Pttg1 | 1,5614 | 3,23E-02 |
| Tlr13 | -1,7264 | 3,23E-02 |
| Svep1 | -1,4078 | 3,23E-02 |
| Cwh43 | 2,0113 | 3,24E-02 |
| Efr3a | -0,8292 | 3,24E-02 |
| Esr1 | -1,0736 | 3,24E-02 |
| Gm22154 | -1,9341 | 3,24E-02 |
| Cmah | -2,2205 | 3,25E-02 |
| Aldh16a1 | 0,9540 | 3,26E-02 |
| Col5a2 | -1,4812 | 3,27E-02 |
| Gm6576 | 1,6891 | 3,28E-02 |
| Hnrnpc | -1,1216 | 3,28E-02 |
| Ints4 | 0,9331 | 3,28E-02 |
| Nav2 | 1,0040 | 3,28E-02 |
| Romo1 | 1,6826 | 3,28E-02 |
| Rps10 | 1,0371 | 3,28E-02 |
| Gad1 | 2,7281 | 3,29E-02 |
| Tbx18 | -1,9789 | 3,30E-02 |
| Slc16a1 | -0,9892 | 3,31E-02 |
| Angpt4 | -2,5148 | 3,32E-02 |
| Mrpl17 | 1,1078 | 3,32E-02 |
| Nudt8 | 1,1650 | 3,32E-02 |
| Ppih | -1,5115 | 3,32E-02 |
| Rp2h | -0,9520 | 3,32E-02 |
| Rpl9 | -0,9116 | 3,32E-02 |
| Gabbr1 | -1,1090 | 3,33E-02 |
| n-R5s103 | -2,4983 | 3,33E-02 |
| Utp18 | -1,0436 | 3,34E-02 |
| Cdkn2b | -2,5752 | 3,34E-02 |
| Tmem218 | 1,8526 | 3,35E-02 |
| Btbd2 | 0,9432 | 3,36E-02 |
| Rnf39 | 3,1632 | 3,36E-02 |
| Yars2 | -1,0488 | 3,36E-02 |
| Tbcc | 1,1991 | 3,36E-02 |
| B4galnt1 | 0,9786 | 3,37E-02 |
| Golga7b | -4,4673 | 3,37E-02 |
| 2010320M18Rik | 1,6599 | 3,44E-02 |
| Bace2 | 0,9166 | 3,47E-02 |
| Col8a1 | -1,5439 | 3,43E-02 |
| Cox6a1 | 1,2365 | 3,43E-02 |
| Cspg5 | 1,1626 | 3,41E-02 |
| Ctdnep1 | -1,0331 | 3,45E-02 |
| Fbn2 | -3,2416 | 3,46E-02 |
| Frem1 | -2,7754 | 3,45E-02 |
| Gm22574 | -2,2593 | 3,44E-02 |
| Gm25679 | 1,4575 | 3,47E-02 |
| Gm9800 | -0,9282 | 3,45E-02 |
| Idh3g | 1,0579 | 3,45E-02 |
| Mrpl14 | 1,3157 | 3,47E-02 |
| Ndufs6 | 1,4228 | 3,46E-02 |
| Papola | -0,8677 | 3,42E-02 |
| Pde4dip | -0,8385 | 3,44E-02 |
| Phldb3 | 1,3022 | 3,45E-02 |
| Plxdc1 | -1,8310 | 3,42E-02 |
| Ptprc | -1,1845 | 3,39E-02 |
| Ralgps2 | -2,3002 | 3,47E-02 |
| Reep3 | -0,8643 | 3,46E-02 |
| Rnf44 | 0,9236 | 3,40E-02 |
| RP23-247F18,5 | -3,0604 | 3,39E-02 |
| Scarna9 | -2,1503 | 3,41E-02 |
| Slc25a39 | 0,8140 | 3,41E-02 |
| Slc9a9 | -2,2249 | 3,46E-02 |
| Snord13 | -1,9165 | 3,43E-02 |
| Ston1 | -0,9453 | 3,46E-02 |
| Tm4sf1 | 0,8160 | 3,44E-02 |
| Tmem165 | -0,8850 | 3,48E-02 |
| Trim16 | -1,0097 | 3,41E-02 |
| Wrb | -1,0511 | 3,40E-02 |
| Zfp771 | 1,3913 | 3,38E-02 |
| Zfp800 | -1,0589 | 3,42E-02 |
| Cdkl5 | 1,2294 | 3,50E-02 |
| Dcxr | 1,5536 | 3,51E-02 |
| Snap29 | -0,9881 | 3,51E-02 |
| Snora19 | -2,0588 | 3,51E-02 |
| Cbx8 | 1,1490 | 3,52E-02 |
| Cdr2 | -1,2863 | 3,52E-02 |
| 3222401L13Rik | -1,7810 | 3,52E-02 |
| Aldoart2 | 1,8082 | 3,53E-02 |
| Asrgl1 | 1,9278 | 3,55E-02 |
| Ccdc15 | -1,7962 | 3,55E-02 |
| Ckap4 | -1,2877 | 3,58E-02 |
| Des | -2,0426 | 3,54E-02 |
| Dhrs9 | -3,7929 | 3,57E-02 |
| Egr3 | 2,3776 | 3,56E-02 |
| Etaa1 | -1,3008 | 3,58E-02 |
| Gli1 | -4,3244 | 3,55E-02 |
| Gm8909 | 1,4176 | 3,56E-02 |
| Gpr85 | -4,2416 | 3,56E-02 |
| Ifrd2 | 1,3482 | 3,54E-02 |
| Rps27l | -1,2900 | 3,58E-02 |
| Sars | 0,8099 | 3,58E-02 |
| Serpinb9 | -1,5090 | 3,58E-02 |
| Tra2b | -0,8981 | 3,56E-02 |
| Vcam1 | -1,4399 | 3,54E-02 |
| Csrp1 | 1,0890 | 3,59E-02 |
| Gm8520 | 2,2626 | 3,59E-02 |
| Rcsd1 | -1,8986 | 3,59E-02 |
| Rps11-ps1 | 2,1505 | 3,59E-02 |
| C130026I21Rik | -1,6578 | 3,62E-02 |
| Foxp4 | 1,0401 | 3,61E-02 |
| Gm11914 | -2,5322 | 3,62E-02 |
| Gm26505 | 1,8303 | 3,61E-02 |
| Khnyn | 0,9915 | 3,60E-02 |
| Trim30d | -1,9255 | 3,60E-02 |
| Xirp2 | -4,3127 | 3,62E-02 |
| Adamtsl5 | -2,0415 | 3,63E-02 |
| Akirin2 | -1,0274 | 3,65E-02 |
| Ash2l | 0,8872 | 3,66E-02 |
| Foxo4 | 1,0531 | 3,65E-02 |
| Gm10033 | -2,0423 | 3,66E-02 |
| Gpr97 | 1,3138 | 3,65E-02 |
| Nr1d1 | 1,1053 | 3,66E-02 |
| Rpl37a | 1,2763 | 3,65E-02 |
| Rrad | -2,4814 | 3,64E-02 |
| Slc7a2 | -1,4560 | 3,65E-02 |
| Snora28 | -1,6681 | 3,64E-02 |
| Adssl1 | 1,5055 | 3,68E-02 |
| Amd2 | -1,1580 | 3,68E-02 |
| Ikbip | -1,2206 | 3,68E-02 |
| Ndufb4 | -1,5479 | 3,68E-02 |
| Sass6 | -1,1760 | 3,67E-02 |
| Gatsl3 | -2,3883 | 3,69E-02 |
| Mpc1-ps | -1,5518 | 3,69E-02 |
| Ms4a6b | -1,8339 | 3,70E-02 |
| Serpine1 | -1,9224 | 3,70E-02 |
| Thy1 | -1,0408 | 3,70E-02 |
| 4930594M22Rik | -2,7624 | 3,71E-02 |
| Rasal1 | 1,0150 | 3,71E-02 |
| Fam129b | 0,8175 | 3,72E-02 |
| Gm1966 | -1,4345 | 3,73E-02 |
| Atp2a1 | -7,0245 | 3,73E-02 |
| Krtcap3 | 1,4856 | 3,74E-02 |
| Mir26a-2 | -2,5005 | 3,75E-02 |
| Stard8 | -2,1258 | 3,76E-02 |
| Fam110c | 1,2800 | 3,78E-02 |
| Vdac2 | 0,8683 | 3,79E-02 |
| RP23-195A24,4 | -1,9545 | 3,80E-02 |
| Sdhc | 0,9974 | 3,79E-02 |
| Rassf9 | -1,6517 | 3,80E-02 |
| Slc25a24 | -0,8936 | 3,81E-02 |
| Foxa3 | 2,3360 | 3,81E-02 |
| Mrpl12 | 1,2337 | 3,81E-02 |
| Gm4070 | -1,2691 | 3,82E-02 |
| Sat2 | 1,6178 | 3,82E-02 |
| Tpst1 | -1,6236 | 3,83E-02 |
| Zfp687 | 0,9747 | 3,84E-02 |
| 2610035D17Rik | 1,8853 | 3,84E-02 |
| Il10ra | -1,2026 | 3,84E-02 |
| Invs | -1,2752 | 3,84E-02 |
| 4921536K21Rik | -2,6299 | 3,85E-02 |
| Gjb1 | -0,8495 | 3,85E-02 |
| Kctd1 | 0,9772 | 3,86E-02 |
| Alcam | -1,3955 | 3,90E-02 |
| Calhm2 | -2,2205 | 3,90E-02 |
| Crnde | 1,3612 | 3,94E-02 |
| Etv5 | 0,9781 | 3,94E-02 |
| Exosc5 | 1,3992 | 3,92E-02 |
| Gm11439 | -2,4414 | 3,87E-02 |
| Gm12057 | -2,7622 | 3,88E-02 |
| Gm25587 | 2,4588 | 3,92E-02 |
| Gtpbp2 | 0,8220 | 3,87E-02 |
| Hoxc6 | -2,3222 | 3,89E-02 |
| Igj | -2,8396 | 3,88E-02 |
| Krt18 | 0,7677 | 3,90E-02 |
| Mansc1 | 0,9266 | 3,92E-02 |
| Ndst3 | -4,0081 | 3,91E-02 |
| Ndufa3 | 1,2785 | 3,94E-02 |
| Pank3 | -0,8581 | 3,91E-02 |
| Parvb | -2,4498 | 3,90E-02 |
| Rhbdl3 | -3,1864 | 3,88E-02 |
| RP24-201C14,12 | -2,6684 | 3,93E-02 |
| Rpl7a-ps10 | 2,8446 | 3,90E-02 |
| Sytl5 | -2,3790 | 3,92E-02 |
| Timp3 | -1,3104 | 3,90E-02 |
| Trbc2 | -2,2580 | 3,91E-02 |
| Wnk1 | -0,7868 | 3,94E-02 |
| Rpl31 | 0,9824 | 3,94E-02 |
| 0610040J01Rik | 0,8536 | 3,95E-02 |
| RP23-18J18,1 | -2,9362 | 3,95E-02 |
| Tmem209 | -0,9790 | 3,96E-02 |
| Psme4 | -0,8774 | 3,96E-02 |
| 2510039O18Rik | 0,9563 | 3,97E-02 |
| Ccser1 | 0,9520 | 3,98E-02 |
| Eif3l | -0,9586 | 3,99E-02 |
| Gcdh | 0,9794 | 3,98E-02 |
| Mir6541 | -3,5903 | 3,98E-02 |
| Mt1 | 1,3907 | 3,99E-02 |
| Ttc22 | 1,2163 | 3,99E-02 |
| Zfp119a | -1,8830 | 3,97E-02 |
| Abhd6 | 1,5203 | 4,00E-02 |
| Atp2b4 | -2,0309 | 4,00E-02 |
| Mdfi | 1,0932 | 4,00E-02 |
| Uqcrfs1 | 0,8996 | 4,00E-02 |
| Chordc1 | -1,1321 | 4,01E-02 |
| 2010300C02Rik | 1,0220 | 4,02E-02 |
| Gm22303 | -1,3596 | 4,02E-02 |
| Rev3l | -0,8546 | 4,03E-02 |
| Ank2 | -2,0201 | 4,03E-02 |
| Ifrd1 | -0,8166 | 4,04E-02 |
| Tpt1-ps6 | 1,3954 | 4,04E-02 |
| RP23-46L5,1 | -1,3311 | 4,05E-02 |
| Smek1 | -0,8586 | 4,05E-02 |
| Gm9493 | -1,6357 | 4,06E-02 |
| Hist1h2al | 2,4233 | 4,06E-02 |
| Uqcrq | 1,3360 | 4,06E-02 |
| Ppargc1a | 1,1460 | 4,07E-02 |
| Cln3 | 0,8469 | 4,08E-02 |
| Rps3a1 | 0,8309 | 4,08E-02 |
| Gm10222 | -1,3826 | 4,09E-02 |
| Gm21718 | -1,1375 | 4,09E-02 |
| Gm23804 | 1,3850 | 4,09E-02 |
| Hist1h3c | 1,2973 | 4,10E-02 |
| Lgals1 | -2,3595 | 4,10E-02 |
| Zbtb6 | -1,0697 | 4,10E-02 |
| Gm4211 | -2,7040 | 4,11E-02 |
| Gm22068 | 1,3452 | 4,11E-02 |
| Cfl2 | -1,0858 | 4,14E-02 |
| Cotl1 | 0,9180 | 4,13E-02 |
| Eci2 | 0,8812 | 4,14E-02 |
| Fut4 | -1,8977 | 4,13E-02 |
| Hist1h4c | 1,2772 | 4,13E-02 |
| Klhdc10 | -0,8120 | 4,13E-02 |
| Rasd1 | 3,7449 | 4,12E-02 |
| Snx21 | 0,8944 | 4,14E-02 |
| Trpt1 | 2,4812 | 4,13E-02 |
| Phlpp1 | 0,9928 | 4,15E-02 |
| Tbc1d2b | -1,0462 | 4,16E-02 |
| RP24-227F14,1 | -1,7943 | 4,16E-02 |
| Klra2 | -1,6959 | 4,17E-02 |
| Tc2n | -1,0349 | 4,20E-02 |
| Trpm4 | 0,8504 | 4,20E-02 |
| Gm25500 | -2,0890 | 4,20E-02 |
| Itga5 | -2,1354 | 4,20E-02 |
| Fuk | 0,9574 | 4,21E-02 |
| Chid1 | -1,0341 | 4,22E-02 |
| Dock10 | -1,1462 | 4,23E-02 |
| Med29 | 2,7917 | 4,23E-02 |
| Rnf24 | -1,4860 | 4,22E-02 |
| sept-07 | -0,8826 | 4,24E-02 |
| Sox10 | 1,1126 | 4,24E-02 |
| Sphk2 | 1,0752 | 4,24E-02 |
| Srsf11 | -0,8822 | 4,23E-02 |
| Tmem205 | 2,9954 | 4,24E-02 |
| Cyth4 | -1,2314 | 4,25E-02 |
| Dyrk2 | -1,3719 | 4,25E-02 |
| Gm13608 | -2,1184 | 4,25E-02 |
| Dyrk3 | 1,1471 | 4,26E-02 |
| Rrm1 | -0,8762 | 4,27E-02 |
| Hmgb1 | -0,8136 | 4,28E-02 |
| Foxn2 | -0,8752 | 4,31E-02 |
| Kcnq3 | 2,4969 | 4,31E-02 |
| Nefm | -4,3150 | 4,30E-02 |
| Pds5b | -0,8339 | 4,30E-02 |
| Pkm | 0,9627 | 4,30E-02 |
| RP23-309A8,3 | -2,3502 | 4,30E-02 |
| Slc4a7 | -1,0769 | 4,30E-02 |
| Crnkl1 | -0,9098 | 4,35E-02 |
| Cry1 | -1,0985 | 4,34E-02 |
| Dpt | -1,8632 | 4,31E-02 |
| Gm15289 | -3,3474 | 4,34E-02 |
| Gm23121 | -2,4314 | 4,34E-02 |
| Ier2 | 1,2037 | 4,33E-02 |
| Ltbp4 | -1,5713 | 4,34E-02 |
| Maged2 | -1,0601 | 4,33E-02 |
| Mysm1 | -1,0641 | 4,34E-02 |
| Plp1 | -1,6410 | 4,34E-02 |
| Rem2 | 1,5573 | 4,32E-02 |
| RP24-546N2,4 | -2,8795 | 4,34E-02 |
| Cd302 | -2,1504 | 4,36E-02 |
| Dctpp1 | 1,5779 | 4,36E-02 |
| Rmdn1 | -1,1747 | 4,37E-02 |
| RP23-268A12,3 | -2,5625 | 4,36E-02 |
| Fbxw9 | 0,8960 | 4,38E-02 |
| Igtp | -1,0208 | 4,38E-02 |
| Jph2 | -1,8102 | 4,39E-02 |
| Pddc1 | 1,2602 | 4,38E-02 |
| Polm | 0,8700 | 4,39E-02 |
| Clec3b | -3,1913 | 4,39E-02 |
| Chadl | 1,3239 | 4,40E-02 |
| Ndrg2 | -1,9117 | 4,40E-02 |
| Ap2s1 | 0,9700 | 4,44E-02 |
| C1s1 | -1,4142 | 4,42E-02 |
| Ctsz | -1,3827 | 4,44E-02 |
| Gm20688 | -2,7757 | 4,42E-02 |
| Gm7389 | -1,7557 | 4,43E-02 |
| Nkrf | -1,3899 | 4,44E-02 |
| Prkdc | -0,9081 | 4,44E-02 |
| Tbcel | -1,7435 | 4,43E-02 |
| Tuba4a | 0,7851 | 4,45E-02 |
| Zfp523 | 0,9781 | 4,44E-02 |
| Brinp3 | -1,5004 | 4,46E-02 |
| Cald1 | -0,7719 | 4,47E-02 |
| Clec12a | -3,6883 | 4,47E-02 |
| Katnbl1 | -1,1279 | 4,46E-02 |
| Mlf1 | -2,7001 | 4,46E-02 |
| Picalm | -0,8804 | 4,46E-02 |
| Slc24a3 | -1,9258 | 4,47E-02 |
| Tpp2 | -0,9314 | 4,47E-02 |
| Gm26110 | 2,0410 | 4,49E-02 |
| Mepce | 1,1059 | 4,50E-02 |
| Ppp2ca | -0,8032 | 4,51E-02 |
| RP23-250G8,14 | -2,7131 | 4,51E-02 |
| Rpl13 | 0,7736 | 4,51E-02 |
| Rundc3a | -3,8938 | 4,51E-02 |
| Tfrc | -0,8388 | 4,51E-02 |
| Arnt2 | 1,1191 | 4,52E-02 |
| Sp100 | -0,9933 | 4,52E-02 |
| Birc3 | -4,8535 | 4,55E-02 |
| Chuk | -0,8707 | 4,55E-02 |
| Kptn | 1,4185 | 4,53E-02 |
| Layn | -2,2069 | 4,56E-02 |
| Mpp4 | -2,6849 | 4,54E-02 |
| Ppif | 1,0148 | 4,56E-02 |
| Setd6 | 1,0716 | 4,54E-02 |
| Spsb4 | 1,0343 | 4,56E-02 |
| Tap1 | 0,8767 | 4,55E-02 |
| Urm1 | 1,3005 | 4,55E-02 |
| Ythdc2 | -1,1382 | 4,55E-02 |
| Fam83h | 0,8595 | 4,56E-02 |
| Gm15440 | 3,1308 | 4,57E-02 |
| Gm15583 | -1,8771 | 4,57E-02 |
| Fam3a | 1,0325 | 4,58E-02 |
| Lrfn4 | 1,0413 | 4,58E-02 |
| Wap | 9,4235 | 4,58E-02 |
| Hspa12b | -1,8212 | 4,59E-02 |
| Abhd17a | 0,9146 | 4,60E-02 |
| Cdhr4 | 2,0434 | 4,60E-02 |
| Rnf25 | 1,5357 | 4,60E-02 |
| Ube2j1 | -1,0384 | 4,61E-02 |
| Zfp931 | -1,5426 | 4,61E-02 |
| Tspyl1 | -0,9501 | 4,62E-02 |
| Nicn1 | 1,3099 | 4,63E-02 |
| Manea | -0,9856 | 4,63E-02 |
| Gm22058 | 2,3469 | 4,64E-02 |
| Gm9762 | -1,7106 | 4,64E-02 |
| Medag | -2,1836 | 4,65E-02 |
| AI467606 | -1,7406 | 4,66E-02 |
| Thbd | -1,9186 | 4,66E-02 |
| Baiap2 | 0,8132 | 4,70E-02 |
| Bmpr1b | -4,0601 | 4,69E-02 |
| Camk2d | -0,8200 | 4,67E-02 |
| Gm12940 | -1,2508 | 4,68E-02 |
| Hddc3 | 1,0840 | 4,70E-02 |
| Itgb1 | -0,8643 | 4,69E-02 |
| Ltf | 1,8566 | 4,67E-02 |
| Mesp1 | 1,5978 | 4,70E-02 |
| Morc3 | -0,9995 | 4,69E-02 |
| Wwp2 | 0,8791 | 4,68E-02 |
| Tcn2 | 0,8779 | 4,71E-02 |
| Atad2 | -0,8658 | 4,72E-02 |
| Cd33 | -1,7136 | 4,72E-02 |
| Hmgn1 | 0,8207 | 4,72E-02 |
| Tex264 | 1,2881 | 4,72E-02 |
| Trappc12 | 0,9694 | 4,73E-02 |
| Fap | -1,7052 | 4,73E-02 |
| Gm22192 | -1,6936 | 4,75E-02 |
| Gm23722 | -2,3502 | 4,75E-02 |
| Hspa8 | -1,0579 | 4,75E-02 |
| Ppp4r2 | -0,9995 | 4,75E-02 |
| mt-Tt | -2,9361 | 4,77E-02 |
| Napa | 0,8234 | 4,76E-02 |
| Ttc19 | -0,9584 | 4,76E-02 |
| Wtap | -0,9291 | 4,77E-02 |
| Igsf9 | 1,1466 | 4,77E-02 |
| Pcdhgb1 | 1,2351 | 4,79E-02 |
| Apcdd1 | -1,9014 | 4,84E-02 |
| Bgn | -1,5982 | 4,84E-02 |
| Cldn12 | -1,4366 | 4,80E-02 |
| Cltc | -0,7785 | 4,80E-02 |
| Gm10221 | -1,7200 | 4,82E-02 |
| Hid1 | 1,3818 | 4,82E-02 |
| Hmox1 | -1,3449 | 4,81E-02 |
| Kras | -1,0598 | 4,82E-02 |
| Mpeg1 | -1,1096 | 4,83E-02 |
| Naa50 | -0,9639 | 4,83E-02 |
| Tle6 | 0,9136 | 4,84E-02 |
| Trf | 1,1858 | 4,83E-02 |
| Ccdc71l | -1,0395 | 4,85E-02 |
| Gm10548 | -3,1670 | 4,86E-02 |
| Kbtbd11 | -1,5751 | 4,85E-02 |
| Loxl4 | -3,0422 | 4,84E-02 |
| Slc35e3 | -0,9115 | 4,85E-02 |
| Wdr43 | -0,8548 | 4,86E-02 |
| Pura | -0,9476 | 4,86E-02 |
| 1700001L05Rik | -2,2663 | 4,88E-02 |
| Fbn1 | -1,7419 | 4,89E-02 |
| Rras | 1,1606 | 4,89E-02 |
| Bcl2l1 | 0,9437 | 4,91E-02 |
| Cd180 | -1,8515 | 4,92E-02 |
| Frs2 | -1,1671 | 4,93E-02 |
| Lsp1 | -1,7663 | 4,92E-02 |
| Map3k2 | -0,8324 | 4,92E-02 |
| Shisa5 | 0,9279 | 4,93E-02 |
| Syngr2 | 1,0384 | 4,90E-02 |
| Tpt1 | 0,9287 | 4,91E-02 |
| Trim26 | -0,8276 | 4,91E-02 |
| Ttc5 | 0,8868 | 4,92E-02 |
| Ublcp1 | -1,1569 | 4,92E-02 |
| Uqcc3 | 1,4247 | 4,92E-02 |
| Zbed3 | 1,7250 | 4,91E-02 |
| Atg4d | 1,1203 | 4,95E-02 |
| RP23-461O23,2 | -1,4423 | 4,95E-02 |
| Gm4951 | -1,7648 | 4,96E-02 |
| Snord82 | -1,6866 | 4,97E-02 |
| Serpinf1 | -1,2796 | 4,97E-02 |
| Vac14 | 0,9550 | 4,99E-02 |
| Gpbp1 | -0,8568 | 4,99E-02 |
| Slc2a1 | 1,0411 | 4,99E-02 |

**Appendix Table S3 Differential gene expression in TNC high (WT/shC) tumors upon AMD and PBS treatment (4 weeks model)**

RNA sequencing data, adj p-value < 0.1, N = 2.

| **Gene Symbol** | **logFC** | **adj. P value** |
| --- | --- | --- |
| Igkc | -6,16630 | 4,14E-115 |
| Cr2 | -5,43276 | 1,48E-46 |
| Igj | -5,73994 | 9,28E-45 |
| Gm4759 | -5,78730 | 8,49E-41 |
| Cyfip2 | -3,84167 | 2,14E-32 |
| Enpp2 | -5,27523 | 3,06E-31 |
| Bank1 | -6,60485 | 7,04E-31 |
| Rasgrp2 | -5,69314 | 2,46E-28 |
| Ighm | -3,34561 | 3,19E-28 |
| Igha | -4,46445 | 4,12E-27 |
| Pou2af1 | -5,43424 | 4,27E-26 |
| Spn | -3,84628 | 3,98E-24 |
| RP23-12D3,3 | -5,50411 | 1,35E-22 |
| Lck | -3,87927 | 1,59E-20 |
| Cmah | -4,17096 | 1,59E-20 |
| Cd3e | -4,64045 | 1,59E-20 |
| Car3 | -4,85229 | 3,65E-20 |
| Ralgps2 | -2,75366 | 4,95E-20 |
| Ttn | -3,03290 | 5,61E-20 |
| Skap1 | -4,77346 | 9,27E-20 |
| Cd2 | -4,70221 | 1,30E-19 |
| Ltb | -4,60399 | 1,70E-19 |
| Cd6 | -5,78010 | 7,76E-19 |
| sept-01 | -4,11819 | 1,32E-18 |
| Tnfrsf13c | -4,36674 | 1,56E-18 |
| Iigp1 | -2,09859 | 1,99E-18 |
| Jakmip1 | -6,71310 | 6,88E-18 |
| Slamf6 | -3,98947 | 7,66E-18 |
| Satb1 | -2,77487 | 9,68E-18 |
| Spib | -5,29382 | 2,61E-17 |
| Il21r | -3,38311 | 3,17E-17 |
| Fam78a | -3,60330 | 3,25E-17 |
| Traf1 | -4,30067 | 4,56E-17 |
| RP23-361M12,2 | -2,95480 | 4,90E-17 |
| Sash3 | -3,09218 | 8,42E-17 |
| Il27ra | -5,36689 | 1,19E-16 |
| Ighg2b | -5,37886 | 1,53E-16 |
| Fcrl1 | -5,68275 | 1,62E-16 |
| Lrrk2 | -2,72556 | 3,62E-16 |
| Birc3 | -2,98367 | 1,27E-15 |
| Traf3ip3 | -3,39367 | 1,27E-15 |
| Cd79a | -4,82451 | 1,52E-15 |
| Rac2 | -2,98032 | 2,19E-15 |
| Slfn1 | -5,20418 | 3,07E-15 |
| C530050E15Rik | -5,91537 | 3,50E-15 |
| Fam196b | -4,01983 | 4,65E-15 |
| Dnase1l3 | -4,35791 | 6,35E-15 |
| Lax1 | -4,86954 | 9,78E-15 |
| Rassf2 | -2,29624 | 1,02E-14 |
| Siglecg | -5,54433 | 1,36E-14 |
| Itga4 | -2,71059 | 1,63E-14 |
| Trac | -4,55545 | 2,50E-14 |
| Epsti1 | -2,67276 | 3,27E-14 |
| Slamf7 | -4,47718 | 5,57E-14 |
| Fcer2a | -3,04068 | 6,12E-14 |
| Ccl5 | -4,35875 | 7,26E-14 |
| Lat | -4,08808 | 9,16E-14 |
| Txnip | -1,13556 | 1,25E-13 |
| Galnt6 | -3,60752 | 1,34E-13 |
| Itgal | -2,65903 | 1,44E-13 |
| Ccr7 | -6,86663 | 2,40E-13 |
| Myo1g | -2,66101 | 2,62E-13 |
| Ptprcap | -3,40656 | 2,62E-13 |
| Ppp1r16b | -3,06715 | 4,61E-13 |
| Akna | -2,12290 | 4,65E-13 |
| Glycam1 | -13,23535 | 5,47E-13 |
| Arhgap30 | -2,20263 | 6,10E-13 |
| Tespa1 | -5,34269 | 7,83E-13 |
| Trbc1 | -5,23980 | 1,09E-12 |
| Ighd | -8,17080 | 1,11E-12 |
| Cd8a | -3,58749 | 1,11E-12 |
| Timd4 | -6,76994 | 1,11E-12 |
| Lrmp | -2,73193 | 1,32E-12 |
| Irf4 | -4,80116 | 1,83E-12 |
| 9930111J21Rik2 | -2,22076 | 2,21E-12 |
| Dpp4 | -4,02922 | 2,41E-12 |
| Syk | -2,27936 | 2,98E-12 |
| Stap1 | -3,80548 | 3,35E-12 |
| Dock11 | -2,40420 | 6,74E-12 |
| Cacna1e | -4,53991 | 7,68E-12 |
| Gimap7 | -6,35924 | 7,73E-12 |
| Ebf1 | -2,48462 | 7,85E-12 |
| Ipcef1 | -3,14591 | 7,85E-12 |
| Il16 | -2,71752 | 9,29E-12 |
| Chst3 | -3,54499 | 1,02E-11 |
| Gbp6 | -2,30537 | 1,49E-11 |
| Pik3cd | -2,20448 | 1,81E-11 |
| Cd247 | -3,61149 | 1,81E-11 |
| Pydc3 | -5,40905 | 1,85E-11 |
| Grap | -3,27243 | 1,91E-11 |
| Rasgrp1 | -2,77710 | 2,15E-11 |
| Rhoh | -3,46625 | 2,21E-11 |
| Coro1a | -1,94980 | 2,23E-11 |
| F830016B08Rik | -2,51534 | 2,25E-11 |
| Sh2d2a | -4,11342 | 2,71E-11 |
| Cfd | -4,72855 | 3,10E-11 |
| Marco | -5,62988 | 3,55E-11 |
| Sell | -7,99103 | 4,04E-11 |
| Cd53 | -2,19079 | 5,05E-11 |
| Tcf7 | -2,22606 | 5,93E-11 |
| Trpm2 | -2,96638 | 8,25E-11 |
| Ptpn22 | -3,08546 | 9,05E-11 |
| Cd74 | -1,19215 | 9,40E-11 |
| Gimap5 | -4,21125 | 9,40E-11 |
| Fmnl1 | -2,03513 | 1,18E-10 |
| Icosl | -2,35567 | 1,29E-10 |
| Lfng | -2,39794 | 1,43E-10 |
| Tgtp2 | -2,48313 | 1,43E-10 |
| Itgb2 | -1,63119 | 1,50E-10 |
| P2ry10 | -4,29684 | 1,50E-10 |
| Treml2 | -4,34186 | 1,50E-10 |
| Ikzf3 | -5,77052 | 1,66E-10 |
| Fabp4 | -2,47759 | 2,07E-10 |
| BC023105 | -2,69494 | 2,07E-10 |
| Ly9 | -2,83581 | 2,16E-10 |
| RP24-146B4,2 | -3,91392 | 2,16E-10 |
| Map4k1 | -2,87135 | 2,32E-10 |
| Pacsin1 | -4,46048 | 3,40E-10 |
| RP24-490B17,6 | -5,06140 | 3,77E-10 |
| Il2rg | -3,26412 | 4,55E-10 |
| Nxpe3 | -3,40547 | 5,08E-10 |
| Cd8b1 | -7,87472 | 5,72E-10 |
| Faim3 | -10,4693 | 5,85E-10 |
| Cd5 | -3,42509 | 5,92E-10 |
| Adam23 | -2,35174 | 5,92E-10 |
| Dnah8 | -5,63692 | 8,08E-10 |
| Sik1 | -1,02817 | 1,23E-09 |
| Grap2 | -2,96533 | 1,31E-09 |
| Gbp9 | -1,94378 | 1,50E-09 |
| Trp53inp1 | -1,35546 | 1,90E-09 |
| Laptm5 | -1,52648 | 1,97E-09 |
| S1pr1 | -2,13054 | 2,11E-09 |
| Arhgdib | -1,88331 | 2,32E-09 |
| 9930111J21Rik1 | -2,61629 | 2,69E-09 |
| Agap2 | -2,59024 | 2,70E-09 |
| Arhgap4 | -2,34231 | 2,78E-09 |
| RP24-490B17,5 | -5,55023 | 2,80E-09 |
| Fam49a | -2,20388 | 2,90E-09 |
| Dsc3 | -7,74134 | 2,90E-09 |
| RP23-373D16,4 | -6,18546 | 3,31E-09 |
| Kbtbd11 | -2,92469 | 3,70E-09 |
| Cd83 | -2,06138 | 4,44E-09 |
| Slc38a1 | -2,61511 | 4,64E-09 |
| Gimap1 | -3,44053 | 4,94E-09 |
| Cd19 | -11,4089 | 5,21E-09 |
| Trbc2 | -3,68604 | 5,25E-09 |
| Fcrla | -5,01486 | 5,59E-09 |
| Clec2i | -4,08228 | 5,80E-09 |
| Igtp | -1,75857 | 5,80E-09 |
| Cxcr5 | -3,05556 | 6,11E-09 |
| Elf4 | -1,93429 | 9,16E-09 |
| Rinl | -2,40533 | 9,16E-09 |
| H2-Aa | -1,11044 | 9,72E-09 |
| Blnk | -1,84233 | 9,87E-09 |
| Irf8 | -1,87356 | 1,10E-08 |
| Stk17b | -1,88873 | 1,11E-08 |
| Dock10 | -1,45481 | 1,17E-08 |
| Stat4 | -4,77057 | 1,27E-08 |
| Cd300lf | -1,89012 | 1,29E-08 |
| Plcl2 | -2,75136 | 1,42E-08 |
| Il9r | -7,29762 | 1,45E-08 |
| Slc4a8 | -4,31673 | 1,71E-08 |
| Pyhin1 | -3,20775 | 1,85E-08 |
| Pla2g2d | -9,92281 | 1,95E-08 |
| Gpr174 | -7,37457 | 1,95E-08 |
| Pik3r5 | -2,25725 | 1,98E-08 |
| Atp2a3 | -5,05663 | 2,17E-08 |
| Cnr2 | -3,32496 | 2,28E-08 |
| Arhgap9 | -2,71238 | 2,32E-08 |
| Cd52 | -2,46683 | 3,53E-08 |
| Fermt3 | -1,54594 | 3,58E-08 |
| RP24-490B17,7 | -4,19962 | 4,05E-08 |
| Igkv6-15 | -9,59975 | 4,08E-08 |
| Pax5 | -9,33057 | 5,69E-08 |
| Gbp2 | -1,55470 | 5,82E-08 |
| Tnxb | -2,77069 | 5,83E-08 |
| Ubash3a | -4,14507 | 6,13E-08 |
| Kcnab2 | -2,45417 | 6,44E-08 |
| Tgtp1 | -2,28027 | 6,44E-08 |
| Pde3b | -1,90869 | 6,46E-08 |
| Flt3 | -3,91378 | 6,46E-08 |
| Cecr2 | -3,98766 | 6,56E-08 |
| Ankrd44 | -1,65412 | 6,82E-08 |
| Vav1 | -1,58664 | 6,95E-08 |
| Samsn1 | -3,22969 | 7,05E-08 |
| Iglc2 | -9,50097 | 7,08E-08 |
| Inpp5d | -1,49558 | 8,17E-08 |
| Ighg1 | -8,04309 | 8,76E-08 |
| Csf2rb2 | -1,67870 | 1,02E-07 |
| Dennd1c | -2,03502 | 1,04E-07 |
| Irgm2 | -1,49635 | 1,08E-07 |
| Csf2rb | -1,54872 | 1,08E-07 |
| Cytip | -1,70512 | 1,13E-07 |
| Arhgap25 | -2,07864 | 1,13E-07 |
| Tap1 | -1,48475 | 1,14E-07 |
| Kcna3 | -4,68063 | 1,17E-07 |
| Zc3h12d | -2,29383 | 1,21E-07 |
| Orai2 | -2,56719 | 1,35E-07 |
| Il2rb | -2,79483 | 1,41E-07 |
| Plcb2 | -1,93458 | 1,53E-07 |
| Lyz1 | -3,99178 | 2,11E-07 |
| A630023P12Rik | -9,35883 | 2,33E-07 |
| Klhl6 | -2,27444 | 2,33E-07 |
| Slc9a7 | -3,33185 | 2,39E-07 |
| Cd3d | -6,19821 | 2,63E-07 |
| Fam46c | -2,04001 | 2,63E-07 |
| Gcsam | -5,39880 | 3,15E-07 |
| Il4i1 | -1,85570 | 3,79E-07 |
| Selplg | -1,81886 | 3,98E-07 |
| 9330175E14Rik | -2,61933 | 3,98E-07 |
| Cd3g | -4,76235 | 4,67E-07 |
| Ccdc88b | -1,87052 | 5,52E-07 |
| Cxcl13 | -8,82892 | 5,58E-07 |
| Cd79b | -10,6943 | 5,64E-07 |
| Ms4a1 | -9,07103 | 5,76E-07 |
| Nsg2 | -9,19937 | 5,81E-07 |
| Inpp4b | -1,56112 | 5,97E-07 |
| Kmo | -2,97460 | 5,99E-07 |
| H2-Eb2 | -9,23697 | 6,69E-07 |
| Fam65b | -1,47763 | 6,87E-07 |
| Gpr68 | -3,20639 | 7,25E-07 |
| Tnfrsf12a | 1,33381 | 7,46E-07 |
| Fli1 | -1,65398 | 8,16E-07 |
| Gbp7 | -1,22567 | 8,92E-07 |
| AI467606 | -3,03436 | 8,92E-07 |
| Slamf1 | -4,80994 | 9,20E-07 |
| Txk | -3,80295 | 9,20E-07 |
| Rgs14 | -2,98139 | 9,65E-07 |
| Slfn8 | -1,51616 | 9,94E-07 |
| Clec2d | -1,21000 | 1,00E-06 |
| Iglv3 | -8,94726 | 1,10E-06 |
| Myh11 | -2,07951 | 1,12E-06 |
| Fyb | -1,50532 | 1,14E-06 |
| Traf5 | -1,80536 | 1,30E-06 |
| RP23-59N15,2 | -6,69502 | 1,32E-06 |
| Rltpr | -2,78620 | 1,38E-06 |
| Lcp2 | -1,90050 | 1,39E-06 |
| Ccnd2 | -1,55765 | 1,43E-06 |
| Per1 | -0,98487 | 1,52E-06 |
| Gm11346 | -6,95137 | 1,52E-06 |
| Fcho1 | -2,81437 | 1,65E-06 |
| H2-DMb2 | -1,74599 | 1,68E-06 |
| F730311O21Rik | -5,33539 | 1,70E-06 |
| Ighg3 | -8,93911 | 1,87E-06 |
| Gm15987 | -3,56307 | 1,90E-06 |
| Runx3 | -1,56603 | 1,91E-06 |
| Lpxn | -2,52986 | 1,93E-06 |
| Gpr171 | -3,30453 | 1,95E-06 |
| Cd69 | -4,14597 | 1,99E-06 |
| Nlrc3 | -2,33481 | 1,99E-06 |
| 5031414D18Rik | -3,71662 | 1,99E-06 |
| Aim2 | -2,24241 | 1,99E-06 |
| Apbb1ip | -1,68046 | 2,14E-06 |
| Plxnc1 | -1,74751 | 2,17E-06 |
| Nuggc | -3,29417 | 2,18E-06 |
| Zap70 | -3,68051 | 2,39E-06 |
| Synpo2 | -2,41138 | 2,48E-06 |
| Prex1 | -1,39429 | 2,57E-06 |
| H2-Ab1 | -0,96225 | 2,97E-06 |
| Itih5 | -2,09863 | 3,11E-06 |
| Serping1 | -1,26683 | 3,28E-06 |
| Dusp10 | -2,94870 | 3,28E-06 |
| Prkcb | -1,79625 | 3,35E-06 |
| Lsp1 | -1,28867 | 3,41E-06 |
| Hcls1 | -1,37842 | 3,46E-06 |
| Icam1 | -1,95648 | 3,50E-06 |
| Ighv3-2 | -8,71090 | 3,50E-06 |
| Siglec1 | -2,02684 | 3,85E-06 |
| Dusp2 | -2,89579 | 3,95E-06 |
| Sidt1 | -2,14140 | 4,29E-06 |
| Vcam1 | -1,65841 | 4,48E-06 |
| Il10ra | -1,24263 | 4,63E-06 |
| Gdpd3 | -1,81218 | 4,66E-06 |
| RP23-68O7,2 | -2,22308 | 5,23E-06 |
| Ccl22 | -8,07932 | 5,39E-06 |
| B3gnt5 | -8,15944 | 5,43E-06 |
| Sp110 | -1,51970 | 5,91E-06 |
| Rapgef4 | -2,31335 | 6,02E-06 |
| Ifi203 | -1,99616 | 6,06E-06 |
| Mcoln2 | -3,05107 | 6,11E-06 |
| Jun | 0,92729 | 6,11E-06 |
| Gsdmd | -1,99757 | 6,33E-06 |
| Gm19980 | -10,7859 | 6,36E-06 |
| Igkv10-96 | -5,08673 | 7,00E-06 |
| Siglech | -2,83417 | 7,18E-06 |
| Gm4951 | -2,25661 | 7,31E-06 |
| C1s1 | -1,75071 | 8,03E-06 |
| Mical1 | -1,89099 | 8,77E-06 |
| Ctse | -2,54414 | 9,20E-06 |
| Spry4 | 0,99758 | 9,56E-06 |
| H60b | -2,62237 | 9,63E-06 |
| Icos | -3,38189 | 9,79E-06 |
| Il2ra | -2,66066 | 1,05E-05 |
| Samhd1 | -1,08539 | 1,10E-05 |
| Tdrp | -3,74613 | 1,12E-05 |
| Was | -1,98516 | 1,16E-05 |
| Apoe | -0,84241 | 1,18E-05 |
| Pkp1 | 1,12728 | 1,22E-05 |
| Tox2 | -4,53570 | 1,40E-05 |
| Tmem71 | -2,79857 | 1,64E-05 |
| Ikbke | -1,21864 | 1,81E-05 |
| Pglyrp2 | -4,71111 | 1,87E-05 |
| Ighj4 | -5,18424 | 1,93E-05 |
| Slc28a2 | -5,54559 | 2,09E-05 |
| Parp14 | -0,99614 | 2,23E-05 |
| A430078G23Rik | -7,00366 | 2,26E-05 |
| Vtn | -5,09203 | 2,34E-05 |
| Cd84 | -1,32708 | 2,34E-05 |
| Palm3 | -4,98705 | 2,35E-05 |
| Ccr6 | -2,61927 | 2,38E-05 |
| Hmcn1 | -1,81841 | 2,42E-05 |
| Sla | -1,54553 | 2,46E-05 |
| Sh2d3c | -1,65900 | 2,55E-05 |
| RP23-373D16,3 | -8,38502 | 2,95E-05 |
| Pcdh15 | -3,33591 | 2,96E-05 |
| Rin3 | -1,35961 | 3,24E-05 |
| Abcg1 | -1,19368 | 3,32E-05 |
| Parvg | -1,82071 | 3,47E-05 |
| RP24-444I15,4 | -2,98935 | 3,54E-05 |
| RP23-397E2,4 | -8,45043 | 3,62E-05 |
| RP24-88B13,3 | -2,46589 | 3,74E-05 |
| C920009B18Rik | -3,70583 | 3,77E-05 |
| Ctss | -0,81344 | 3,86E-05 |
| Zfp831 | -6,51472 | 3,86E-05 |
| Gm12250 | -2,20275 | 4,30E-05 |
| Tmem156 | -5,00949 | 4,41E-05 |
| Oas2 | -1,90194 | 4,51E-05 |
| Ankrd33b | -3,05999 | 4,81E-05 |
| Chst15 | -1,43568 | 4,81E-05 |
| Ighv1-9 | -8,17083 | 4,90E-05 |
| Il7r | -4,39066 | 4,90E-05 |
| Pou2f2 | -1,58082 | 4,90E-05 |
| Cd72 | -1,30472 | 4,96E-05 |
| Abcg3 | -2,44646 | 5,13E-05 |
| Btg2 | -1,39394 | 5,31E-05 |
| Igkv8-27 | -7,55175 | 5,53E-05 |
| Tnfrsf13b | -2,24219 | 5,75E-05 |
| BC035044 | -4,75374 | 5,91E-05 |
| Hp | -3,00864 | 6,16E-05 |
| Bcl11a | -1,12607 | 6,19E-05 |
| Cd22 | -4,86298 | 6,19E-05 |
| Ly6d | -6,27572 | 6,19E-05 |
| Mef2c | -1,12008 | 6,38E-05 |
| Cyr61 | 1,11881 | 6,38E-05 |
| Cd274 | -2,58028 | 6,44E-05 |
| Trbv3 | -5,30450 | 6,48E-05 |
| Cd48 | -2,17526 | 7,12E-05 |
| RP23-291L24,4 | -8,17177 | 7,14E-05 |
| Prkcq | -4,95505 | 7,19E-05 |
| Ccdc88c | -1,18394 | 7,28E-05 |
| Gbp8 | -2,15187 | 7,28E-05 |
| S1pr4 | -3,70400 | 7,41E-05 |
| Traj6 | -8,19371 | 7,64E-05 |
| Pigr | -0,98170 | 7,68E-05 |
| Cd37 | -4,79302 | 7,71E-05 |
| AI504432 | -2,60928 | 7,73E-05 |
| Mreg | -3,82358 | 7,84E-05 |
| Nckap1l | -1,16745 | 7,84E-05 |
| Igkv8-30 | -7,48859 | 7,98E-05 |
| Gimap8 | -3,69878 | 8,00E-05 |
| Cd40 | -2,50177 | 8,31E-05 |
| Ikzf1 | -1,27220 | 8,33E-05 |
| Il6ra | -1,14390 | 8,37E-05 |
| Irf5 | -1,26831 | 8,45E-05 |
| Sp100 | -2,93036 | 8,46E-05 |
| Arhgap15 | -3,28523 | 8,67E-05 |
| Havcr1 | -7,54063 | 8,80E-05 |
| Srgn | -1,33181 | 8,87E-05 |
| Wdfy4 | -2,90242 | 9,04E-05 |
| Bin2 | -1,66332 | 9,08E-05 |
| Ighv5-17 | -4,01327 | 9,17E-05 |
| St8sia4 | -1,49864 | 9,22E-05 |
| Tox | -2,85972 | 9,61E-05 |
| Tbcel | -2,17643 | 1,02E-04 |
| Ighe | -6,17476 | 1,03E-04 |
| Dgka | -0,93303 | 1,05E-04 |
| Slco2b1 | -1,78216 | 1,06E-04 |
| Cd226 | -2,94028 | 1,09E-04 |
| Mfng | -2,14433 | 1,11E-04 |
| Ciita | -1,08689 | 1,11E-04 |
| Spic | -4,05412 | 1,11E-04 |
| Psmb9 | -1,37712 | 1,15E-04 |
| Atp8b4 | -2,93127 | 1,17E-04 |
| Abcc4 | -1,88749 | 1,22E-04 |
| Cbfa2t3 | -1,64378 | 1,24E-04 |
| Dcp1b | -2,56222 | 1,24E-04 |
| Igkv1-117 | -7,32041 | 1,29E-04 |
| Cd4 | -3,39345 | 1,33E-04 |
| Aff3 | -3,77789 | 1,40E-04 |
| Hmha1 | -3,04954 | 1,49E-04 |
| Dok3 | -1,48086 | 1,55E-04 |
| Dclk1 | -1,55461 | 1,55E-04 |
| Gimap3 | -5,31881 | 1,56E-04 |
| Slco5a1 | -3,14841 | 1,59E-04 |
| AI662270 | -2,13489 | 1,69E-04 |
| Rnase6 | -2,50986 | 1,73E-04 |
| RP24-490B17,1 | -7,32159 | 1,73E-04 |
| Pydc4 | -7,55350 | 1,75E-04 |
| Igkv1-110 | -7,33436 | 1,75E-04 |
| Il22ra2 | -7,31049 | 1,76E-04 |
| Strip2 | -4,15072 | 1,78E-04 |
| Plin4 | -1,74301 | 1,84E-04 |
| Ctla4 | -3,49574 | 1,84E-04 |
| Pik3ap1 | -0,81649 | 1,89E-04 |
| Themis | -4,85053 | 1,89E-04 |
| Cxcl12 | -1,35988 | 1,92E-04 |
| Gimap4 | -3,30814 | 1,94E-04 |
| Rgs1 | -2,07894 | 2,08E-04 |
| Itk | -7,03021 | 2,09E-04 |
| Filip1l | -0,91566 | 2,21E-04 |
| Igkv12-46 | -7,18316 | 2,23E-04 |
| Ubd | -7,28092 | 2,24E-04 |
| Bcl11b | -4,16696 | 2,25E-04 |
| H2-M2 | -1,84606 | 2,38E-04 |
| RP23-307N14,2 | -4,42343 | 2,38E-04 |
| Ms4a4c | -3,69502 | 2,41E-04 |
| Dock2 | -2,81105 | 2,42E-04 |
| AW112010 | -2,03152 | 2,42E-04 |
| Gm5970 | -4,67620 | 2,51E-04 |
| Nrros | -1,52421 | 2,52E-04 |
| Adrbk2 | -1,67626 | 2,52E-04 |
| H2-T3 | -3,36530 | 2,58E-04 |
| Igkv16-104 | -8,05353 | 2,60E-04 |
| 4930523C07Rik | -1,09324 | 2,60E-04 |
| Rgs13 | -7,90171 | 2,70E-04 |
| Rhof | -1,77433 | 2,73E-04 |
| Fcamr | -4,55668 | 2,78E-04 |
| Rbm38 | -1,80082 | 2,84E-04 |
| Ptprc | -2,38592 | 2,88E-04 |
| Plin1 | -7,11560 | 2,91E-04 |
| H2-Ea-ps | -2,41587 | 2,91E-04 |
| Ppm1m | -1,85750 | 2,93E-04 |
| Card11 | -3,78282 | 2,96E-04 |
| Itgae | -1,88604 | 2,98E-04 |
| Rftn1 | -1,36802 | 2,98E-04 |
| Lincpint | -0,77559 | 2,98E-04 |
| Gbp4 | -2,87715 | 2,98E-04 |
| Igkv12-44 | -7,75037 | 2,98E-04 |
| Slfn2 | -1,30829 | 3,08E-04 |
| C3 | -3,01989 | 3,10E-04 |
| Igkj1 | -7,73301 | 3,23E-04 |
| Ampd3 | -2,05314 | 3,28E-04 |
| Cers4 | -2,31946 | 3,29E-04 |
| Cd40lg | -7,15742 | 3,36E-04 |
| A630001G21Rik | -1,06150 | 3,59E-04 |
| Shank1 | -2,70711 | 3,64E-04 |
| Gbp3 | -0,95675 | 3,72E-04 |
| Bdh1 | -4,07243 | 3,74E-04 |
| RP23-328G14,1 | -5,90075 | 3,80E-04 |
| Krt19 | -5,97811 | 3,85E-04 |
| Plcb4 | -1,50776 | 3,87E-04 |
| Helz2 | -2,81939 | 3,91E-04 |
| Bend4 | -3,77628 | 4,01E-04 |
| Stab2 | -3,52273 | 4,05E-04 |
| Rasal3 | -3,27875 | 4,11E-04 |
| Snx20 | -2,16448 | 4,15E-04 |
| Celsr1 | -2,32449 | 4,24E-04 |
| RP24-490B17,4 | -4,52033 | 4,37E-04 |
| Cxcl9 | -1,96785 | 4,37E-04 |
| Ighv1-64 | -7,75235 | 4,47E-04 |
| Pck1 | -3,46849 | 4,51E-04 |
| Lmo2 | -1,70126 | 4,52E-04 |
| Sfmbt2 | -4,17496 | 4,52E-04 |
| Tbc1d10c | -3,98371 | 4,55E-04 |
| Gpr132 | -3,10951 | 4,56E-04 |
| Ighv1-34 | -7,67163 | 4,60E-04 |
| RP23-459L15,7 | -0,89200 | 4,78E-04 |
| 4931429I11Rik | -7,70742 | 4,80E-04 |
| Ets1 | -0,75861 | 4,81E-04 |
| Igkv4-57 | -7,67278 | 4,90E-04 |
| Gm8995 | -2,89453 | 5,00E-04 |
| Cpne4 | -7,12233 | 5,26E-04 |
| Ak4 | 1,54950 | 5,29E-04 |
| RP24-146B4,3 | -2,33532 | 5,41E-04 |
| WI1-2075L23,1 | -3,09820 | 5,47E-04 |
| Ighv1-26 | -5,65086 | 5,57E-04 |
| Rel | -1,34902 | 5,61E-04 |
| Igkv8-21 | -7,60877 | 5,67E-04 |
| Abcc5 | -1,40942 | 5,98E-04 |
| Pip4k2a | -1,00250 | 6,01E-04 |
| Fbxo32 | 0,61268 | 6,07E-04 |
| Fam208a | -1,15238 | 6,07E-04 |
| Jak3 | -1,00471 | 6,25E-04 |
| Lyz2 | -0,92824 | 6,43E-04 |
| Rcsd1 | -3,50021 | 6,44E-04 |
| Tns1 | -0,90983 | 6,54E-04 |
| Fam46a | -0,96736 | 6,55E-04 |
| Igkv17-121 | -7,56152 | 6,56E-04 |
| Ighv1-82 | -7,70494 | 6,64E-04 |
| Tpi1 | 0,69859 | 6,68E-04 |
| Cacna2d4 | -3,43288 | 6,68E-04 |
| Hey1 | -0,57788 | 6,83E-04 |
| Cyp1b1 | -1,80384 | 6,98E-04 |
| Serpinb6b | -2,45782 | 6,98E-04 |
| Itgb7 | -4,38603 | 7,05E-04 |
| Cd180 | -1,90152 | 7,19E-04 |
| Ifi205 | -2,04236 | 7,26E-04 |
| Dusp6 | 0,75317 | 7,37E-04 |
| Dtx1 | -1,31435 | 7,45E-04 |
| Clec10a | -2,19421 | 7,62E-04 |
| H2-Oa | -4,21599 | 8,09E-04 |
| Ighv3-1 | -6,84242 | 8,09E-04 |
| Kif21b | -2,36367 | 8,15E-04 |
| Klk8 | -4,03809 | 8,21E-04 |
| Ddx60 | -1,04415 | 8,23E-04 |
| Aicda | -6,90323 | 8,23E-04 |
| Pld4 | -1,07676 | 8,53E-04 |
| RP24-88B13,7 | -2,79137 | 8,86E-04 |
| Kcnq5 | -5,03698 | 8,93E-04 |
| Gm4841 | -2,73155 | 9,05E-04 |
| RP24-490B17,3 | -7,56911 | 9,06E-04 |
| Gm26877 | -5,64650 | 9,42E-04 |
| AU020206 | -1,05233 | 9,48E-04 |
| Fgr | -1,40533 | 9,49E-04 |
| Fgl2 | -1,30005 | 9,70E-04 |
| Klrk1 | -2,94259 | 9,89E-04 |
| Camk1d | -1,49606 | 1,00E-03 |
| Tfrc | 0,64504 | 1,01E-03 |
| Fas | -1,97770 | 1,02E-03 |
| Cd7 | -3,84799 | 1,02E-03 |
| Tlr1 | -2,26876 | 1,02E-03 |
| Ctsw | -1,98175 | 1,05E-03 |
| March1 | -1,30789 | 1,11E-03 |
| Fcrls | 1,86348 | 1,16E-03 |
| Arid3b | -1,28826 | 1,17E-03 |
| Gcnt4 | 1,60395 | 1,19E-03 |
| Hmgcs2 | -2,96864 | 1,25E-03 |
| Des | -2,53211 | 1,26E-03 |
| Nefh | -3,35488 | 1,28E-03 |
| Ighj3 | -6,74604 | 1,30E-03 |
| Camk4 | -1,65825 | 1,31E-03 |
| Slc2a3 | -2,67537 | 1,34E-03 |
| N4bp2l1 | -0,95984 | 1,34E-03 |
| Igkv6-17 | -10,0480 | 1,39E-03 |
| Hbegf | 1,35782 | 1,41E-03 |
| Btk | -1,79822 | 1,41E-03 |
| Gm22567 | 0,87181 | 1,41E-03 |
| Plbd1 | -1,29294 | 1,42E-03 |
| Ighv2-2 | -7,42548 | 1,44E-03 |
| Tspan32 | -1,26738 | 1,51E-03 |
| RP23-328G14,2 | -6,70539 | 1,52E-03 |
| Gm17230 | -1,48031 | 1,53E-03 |
| Chst4 | -7,69027 | 1,54E-03 |
| Klf2 | -1,05341 | 1,57E-03 |
| Col8a2 | -1,26106 | 1,65E-03 |
| Arid5a | -0,65799 | 1,68E-03 |
| Ighj2 | -4,20237 | 1,74E-03 |
| Folr4 | -4,36718 | 1,74E-03 |
| Ighv1-66 | -6,63007 | 1,75E-03 |
| Ggt5 | -2,07055 | 1,75E-03 |
| Napsa | -2,22897 | 1,76E-03 |
| Igkv6-25 | -7,37847 | 1,76E-03 |
| Cacna1i | -2,94884 | 1,81E-03 |
| Ighv8-11 | -5,52591 | 1,86E-03 |
| Mbd4 | -1,02044 | 1,86E-03 |
| Bach2 | -1,07698 | 1,86E-03 |
| Igkv6-23 | -4,54730 | 1,87E-03 |
| Slc1a3 | -2,72427 | 1,88E-03 |
| Slfn5 | -1,08398 | 1,89E-03 |
| Wnk4 | 0,66390 | 1,89E-03 |
| Rapgef4os3 | -3,61770 | 1,89E-03 |
| Slpi | -1,68130 | 1,95E-03 |
| RP23-175B15,2 | -1,48178 | 1,96E-03 |
| Slc8a1 | -1,19083 | 1,98E-03 |
| Neurl3 | -1,29814 | 2,02E-03 |
| Egln3 | 0,73824 | 2,03E-03 |
| RP23-397E2,5 | -6,61447 | 2,05E-03 |
| Hpse | -1,94600 | 2,06E-03 |
| Adam6b | -7,32660 | 2,09E-03 |
| Gm16151 | -7,28330 | 2,16E-03 |
| Tnfrsf14 | -1,37917 | 2,19E-03 |
| Aim1l | -1,86335 | 2,21E-03 |
| Igkv10-94 | -7,53569 | 2,21E-03 |
| Tnfaip3 | -1,01789 | 2,21E-03 |
| Nfam1 | -1,41401 | 2,21E-03 |
| Trerf1 | -1,68901 | 2,25E-03 |
| Man2a2 | -1,00589 | 2,26E-03 |
| Trpv2 | -1,66623 | 2,27E-03 |
| Zfp750 | -0,71152 | 2,29E-03 |
| B4galt5 | -1,19142 | 2,32E-03 |
| Zdhhc15 | -3,80404 | 2,33E-03 |
| Dapl1 | -6,57440 | 2,38E-03 |
| Map4k2 | -0,97295 | 2,40E-03 |
| Slc16a5 | -4,62222 | 2,43E-03 |
| Rasgrp3 | -1,43788 | 2,45E-03 |
| Scube1 | -3,50940 | 2,52E-03 |
| Ppnr | -1,01306 | 2,55E-03 |
| Gm8909 | -1,11730 | 2,62E-03 |
| RP23-36P10,10 | -7,23479 | 2,66E-03 |
| Trav3-3 | -7,28296 | 2,71E-03 |
| Igkv15-103 | -6,67087 | 2,85E-03 |
| Def6 | -0,97764 | 2,88E-03 |
| Serpina3f | -2,55879 | 2,97E-03 |
| Gpr83 | -7,19640 | 2,99E-03 |
| Nup210 | -0,86679 | 3,10E-03 |
| Chic1 | -2,01078 | 3,16E-03 |
| Cd200r1 | -3,40773 | 3,17E-03 |
| Glul | -1,06603 | 3,18E-03 |
| H2-K2 | -1,41470 | 3,23E-03 |
| Clcf1 | -1,65355 | 3,33E-03 |
| Igkv3-5 | -7,29991 | 3,34E-03 |
| Igkj5 | -6,46748 | 3,37E-03 |
| H2-M3 | -1,35106 | 3,55E-03 |
| Ero1l | 0,76695 | 3,63E-03 |
| Ighv8-8 | -9,14383 | 3,66E-03 |
| Ighv1-53 | -7,15151 | 3,71E-03 |
| Man1a | -0,94295 | 3,77E-03 |
| Tsc22d3 | -0,70229 | 3,87E-03 |
| 4930503L19Rik | 0,70454 | 3,91E-03 |
| Ms4a6b | -2,76267 | 3,94E-03 |
| Traj22 | -6,47394 | 3,95E-03 |
| Gm14446 | -5,69537 | 3,96E-03 |
| Traj12 | -7,24505 | 3,99E-03 |
| 2210016F16Rik | -1,99855 | 4,07E-03 |
| Xlr4a | -7,11766 | 4,11E-03 |
| Bnip3 | 0,95151 | 4,14E-03 |
| Dok1 | 0,72814 | 4,18E-03 |
| Ngfr | -4,08100 | 4,19E-03 |
| Arid3a | -1,04571 | 4,21E-03 |
| Serpina10 | -7,13259 | 4,23E-03 |
| Rgs3 | -1,42937 | 4,23E-03 |
| Adh1 | -6,38580 | 4,30E-03 |
| Themis2 | -1,11948 | 4,32E-03 |
| Ehbp1l1 | -0,75160 | 4,46E-03 |
| Gimap6 | -2,95925 | 4,46E-03 |
| BC094916 | -3,34501 | 4,46E-03 |
| Ptpn6 | -0,68341 | 4,63E-03 |
| Trbv2 | -7,14747 | 4,63E-03 |
| Ighv1-55 | -7,09827 | 4,63E-03 |
| Ighv1-69 | -6,42050 | 4,66E-03 |
| RP24-74O18,2 | -1,08510 | 4,67E-03 |
| Susd3 | -2,04111 | 4,74E-03 |
| Fgd5 | -1,08610 | 4,82E-03 |
| Zbp1 | -0,92486 | 4,88E-03 |
| Rgs10 | -1,02003 | 4,89E-03 |
| Tgfbi | -0,68689 | 4,91E-03 |
| Lta | -3,19320 | 4,92E-03 |
| Nod2 | -2,02956 | 5,11E-03 |
| Igkv17-127 | -7,05600 | 5,11E-03 |
| Snord15a | 0,53170 | 5,18E-03 |
| H2-DMa | -1,27138 | 5,21E-03 |
| Pecam1 | -0,69730 | 5,21E-03 |
| Tap2 | -0,70542 | 5,37E-03 |
| Gm26551 | -2,32083 | 5,47E-03 |
| Fam26f | -3,52505 | 5,47E-03 |
| Fgd3 | -1,17179 | 5,51E-03 |
| Cd209b | -6,15146 | 5,51E-03 |
| Mmp13 | 1,34882 | 5,52E-03 |
| Uba7 | -0,84061 | 5,53E-03 |
| Lpar5 | -4,10524 | 5,53E-03 |
| Arrdc3 | -0,51513 | 5,65E-03 |
| Gm12462 | -6,44192 | 5,73E-03 |
| Kcnk5 | -0,58301 | 5,73E-03 |
| Cxcr4 | -0,66862 | 5,74E-03 |
| Slc9a9 | -1,56680 | 5,77E-03 |
| Col6a4 | -6,30965 | 5,77E-03 |
| Ldha | 0,53043 | 5,79E-03 |
| Irgm1 | -0,68822 | 5,93E-03 |
| Ccl8 | -1,56757 | 5,98E-03 |
| Eomes | -3,27484 | 6,01E-03 |
| Qprt | -7,16983 | 6,08E-03 |
| Il6st | -0,59471 | 6,09E-03 |
| Slc15a3 | -1,46629 | 6,10E-03 |
| Haao | -2,54136 | 6,14E-03 |
| Igkv5-43 | -6,26595 | 6,15E-03 |
| Sirpa | -0,62573 | 6,20E-03 |
| Irf7 | -0,83728 | 6,34E-03 |
| Trbv1 | -4,44889 | 6,45E-03 |
| Adipoq | -3,82599 | 6,45E-03 |
| Atp10d | -1,30921 | 6,74E-03 |
| Jade2 | -0,80200 | 6,77E-03 |
| H2-T23 | -0,79157 | 6,78E-03 |
| Egr2 | 1,09042 | 6,82E-03 |
| Adcy5 | -1,52535 | 6,96E-03 |
| RP23-84E4,3 | -3,16239 | 7,16E-03 |
| Tmem229b | -0,75138 | 7,18E-03 |
| Gm10521 | -4,64907 | 7,19E-03 |
| Sla2 | -2,19491 | 7,21E-03 |
| Amica1 | -2,50976 | 7,22E-03 |
| Igkv2-137 | -7,24210 | 7,38E-03 |
| Trim30b | -5,37060 | 7,46E-03 |
| Bmf | -0,70360 | 7,71E-03 |
| Colq | -3,63287 | 7,83E-03 |
| D16Ertd472e | -1,69939 | 7,89E-03 |
| Pltp | -1,32342 | 8,04E-03 |
| Abca1 | -0,65077 | 8,06E-03 |
| Arl5c | -2,02690 | 8,06E-03 |
| RP23-216O10,9 | -6,93124 | 8,08E-03 |
| Rbp7 | 0,71636 | 8,14E-03 |
| Unc93b1 | -0,68711 | 8,14E-03 |
| Pard3b | -1,76430 | 8,15E-03 |
| Igkv6-14 | -6,91771 | 8,19E-03 |
| Cybb | -0,68686 | 8,22E-03 |
| Cd86 | -1,30631 | 8,29E-03 |
| Trim35 | -1,04030 | 8,33E-03 |
| Myo1f | -0,94560 | 8,41E-03 |
| Sit1 | -5,11152 | 8,41E-03 |
| Dennd4b | -1,10186 | 8,41E-03 |
| Irf1 | -0,77446 | 8,46E-03 |
| Gm1966 | -2,25813 | 8,47E-03 |
| Gm16439 | 1,17834 | 8,67E-03 |
| Gbp5 | -2,59456 | 8,76E-03 |
| RP23-354D10,2 | -2,70697 | 8,77E-03 |
| Mbnl1 | -0,62152 | 8,77E-03 |
| Ccnd1 | 0,65400 | 8,79E-03 |
| Nes | 0,75489 | 8,89E-03 |
| Ly6c2 | -3,98480 | 8,89E-03 |
| Cntrl | -0,60034 | 9,01E-03 |
| RP23-145F9,3 | -6,24623 | 9,22E-03 |
| Gm12158 | -3,36037 | 9,41E-03 |
| Pfkfb3 | -0,67644 | 9,57E-03 |
| Nfkbid | -1,67035 | 9,57E-03 |
| C920025E04Rik | -0,92355 | 9,62E-03 |
| Eln | -1,44970 | 9,68E-03 |
| Gm10499 | -1,28468 | 9,72E-03 |
| Plek | -0,98098 | 9,74E-03 |
| Dpy19l3 | -2,00136 | 9,77E-03 |
| Fcgbp | -1,14977 | 9,88E-03 |
| Mycl | -4,70093 | 9,98E-03 |
| Svep1 | -0,76760 | 1,00E-02 |
| Nuak2 | -1,61017 | 1,00E-02 |
| Hvcn1 | -1,00905 | 1,04E-02 |
| Gm16150 | -6,98126 | 1,05E-02 |
| Gem | -1,56749 | 1,06E-02 |
| Hal | -2,00142 | 1,06E-02 |
| Crebrf | -0,54024 | 1,07E-02 |
| Evl | -0,76829 | 1,10E-02 |
| Sdc3 | -0,59450 | 1,12E-02 |
| Gm25835 | 0,54299 | 1,12E-02 |
| Il1rap | 0,57979 | 1,13E-02 |
| Nudt16 | -2,40021 | 1,13E-02 |
| Fam107b | -1,08641 | 1,13E-02 |
| Tnfsf11 | -3,55846 | 1,15E-02 |
| Igkv12-41 | -6,09995 | 1,16E-02 |
| Afap1l1 | 0,63259 | 1,17E-02 |
| Fam162a | 0,71750 | 1,18E-02 |
| Cd28 | -4,74856 | 1,18E-02 |
| Igkv4-55 | -6,89653 | 1,18E-02 |
| Fasl | -4,53090 | 1,20E-02 |
| Gm15872 | -2,85348 | 1,21E-02 |
| Axin2 | -1,88736 | 1,21E-02 |
| Cidec | -4,02916 | 1,23E-02 |
| Gvin1 | -1,86320 | 1,23E-02 |
| Pik3cg | -0,61002 | 1,24E-02 |
| Taok3 | -0,73597 | 1,24E-02 |
| 6530402F18Rik | -1,94391 | 1,26E-02 |
| Rny1 | 0,91905 | 1,26E-02 |
| Lyve1 | -2,55098 | 1,26E-02 |
| Sipa1 | -0,78296 | 1,27E-02 |
| Lifr | -0,60902 | 1,27E-02 |
| Cbx7 | -0,80293 | 1,27E-02 |
| Thy1 | -1,18942 | 1,29E-02 |
| Tbx21 | -3,70754 | 1,30E-02 |
| Cst7 | -3,23136 | 1,31E-02 |
| Wipf1 | -0,75805 | 1,33E-02 |
| Ms4a6c | -1,18094 | 1,33E-02 |
| Bnc1 | 6,78284 | 1,33E-02 |
| Il1b | -1,13326 | 1,34E-02 |
| Mpeg1 | -0,56545 | 1,34E-02 |
| Rnf213 | -0,64022 | 1,35E-02 |
| Cd151 | 0,62546 | 1,36E-02 |
| Nfatc2 | -0,75213 | 1,38E-02 |
| Tnip1 | -0,76663 | 1,38E-02 |
| Slc12a6 | -0,64895 | 1,38E-02 |
| Bcor | -0,75139 | 1,38E-02 |
| H2-T10 | -0,84738 | 1,38E-02 |
| Gm16565 | -6,87688 | 1,40E-02 |
| Fosl2 | -0,51960 | 1,42E-02 |
| Serpina3g | -1,91166 | 1,43E-02 |
| Acap1 | -2,62391 | 1,44E-02 |
| Igf1 | -0,97196 | 1,46E-02 |
| Marveld1 | -0,94123 | 1,46E-02 |
| Itga9 | -0,93619 | 1,48E-02 |
| Hdac9 | -1,50524 | 1,48E-02 |
| St3gal1 | -1,23616 | 1,48E-02 |
| Oasl2 | -0,93257 | 1,48E-02 |
| Serpinb1a | -2,27014 | 1,48E-02 |
| H2-Q1 | -1,16693 | 1,48E-02 |
| Tm6sf1 | -1,32531 | 1,51E-02 |
| Ms4a4b | -4,67202 | 1,53E-02 |
| Malt1 | -0,64130 | 1,54E-02 |
| H2-T24 | -1,17842 | 1,54E-02 |
| Il12b | -6,84034 | 1,55E-02 |
| Stat2 | -0,80979 | 1,58E-02 |
| Hsh2d | -3,72400 | 1,59E-02 |
| Socs5 | 0,61781 | 1,59E-02 |
| Susd1 | -2,67257 | 1,59E-02 |
| 2010016I18Rik | -0,87716 | 1,59E-02 |
| RP24-88B13,5 | -2,07054 | 1,59E-02 |
| Ccnd3 | -0,64732 | 1,62E-02 |
| Iglv1 | -6,72527 | 1,62E-02 |
| Ighv1-7 | -6,73018 | 1,63E-02 |
| Gm13185 | -4,52586 | 1,64E-02 |
| Neto2 | 0,70407 | 1,65E-02 |
| Lama1 | -1,89683 | 1,66E-02 |
| Apobec1 | -0,79989 | 1,71E-02 |
| Xlr4b | -3,46726 | 1,71E-02 |
| Dok2 | -2,03869 | 1,71E-02 |
| Tm4sf1 | 0,66321 | 1,71E-02 |
| Rasgrf2 | -2,63815 | 1,71E-02 |
| Gad1 | 1,33317 | 1,71E-02 |
| Gm18301 | -2,02407 | 1,71E-02 |
| RP23-36P10,9 | -6,80285 | 1,71E-02 |
| Olfml1 | -5,94881 | 1,73E-02 |
| Lrp12 | 0,70681 | 1,74E-02 |
| Nfatc1 | -0,68722 | 1,74E-02 |
| Mfap3l | 0,73836 | 1,75E-02 |
| Igkv4-91 | -3,72124 | 1,76E-02 |
| Zfp275 | -1,31064 | 1,77E-02 |
| Slc2a6 | -1,05373 | 1,79E-02 |
| Asap1 | -0,65657 | 1,80E-02 |
| Susd2 | -2,33589 | 1,81E-02 |
| Olfr164 | -3,81114 | 1,81E-02 |
| Fgf13 | -3,55789 | 1,85E-02 |
| Dbn1 | 0,76122 | 1,85E-02 |
| Dscaml1 | -6,70479 | 1,89E-02 |
| Wisp1 | 0,81646 | 1,90E-02 |
| Abca7 | -0,68123 | 1,92E-02 |
| Stk10 | -0,61329 | 1,92E-02 |
| Csf1r | -0,53581 | 1,92E-02 |
| Arhgap29 | 0,58138 | 1,99E-02 |
| Vmn2r84 | -6,65411 | 2,00E-02 |
| RP24-444I15,1 | -3,37126 | 2,00E-02 |
| Nfkbia | -0,64886 | 2,01E-02 |
| Zeb2 | -0,63254 | 2,02E-02 |
| Prf1 | -2,70644 | 2,02E-02 |
| Sh2d1a | -3,80317 | 2,03E-02 |
| Gm4070 | -1,50338 | 2,04E-02 |
| Cacna1b | -4,69017 | 2,05E-02 |
| Gpr35 | -1,30005 | 2,05E-02 |
| Rhob | 0,56280 | 2,05E-02 |
| Arrb2 | -0,77380 | 2,05E-02 |
| Gm4735 | 0,68986 | 2,10E-02 |
| Apln | 1,36575 | 2,10E-02 |
| Lrg1 | -1,62757 | 2,12E-02 |
| Kcnj10 | -1,39148 | 2,13E-02 |
| H2-Ob | -3,20922 | 2,13E-02 |
| Tbxas1 | -1,31164 | 2,15E-02 |
| Clec3b | -3,14615 | 2,16E-02 |
| Dnajc6 | -4,20289 | 2,16E-02 |
| Trim16 | 0,80016 | 2,16E-02 |
| Gm10800 | 0,87125 | 2,19E-02 |
| Pcbp4 | 0,62907 | 2,19E-02 |
| Nlrc5 | -2,24559 | 2,19E-02 |
| Cerk | -0,89800 | 2,20E-02 |
| Traj15 | -6,72984 | 2,21E-02 |
| C4b | -2,33580 | 2,22E-02 |
| Gm16152 | -4,12276 | 2,32E-02 |
| mmu-mir-5103 | -2,74924 | 2,33E-02 |
| Asb2 | -1,58688 | 2,33E-02 |
| Dtx3l | -0,52752 | 2,34E-02 |
| Gm11814 | 0,93326 | 2,34E-02 |
| Gm20506 | -6,60522 | 2,36E-02 |
| Gatsl2 | -1,11681 | 2,36E-02 |
| Sqle | 0,64107 | 2,37E-02 |
| Hmcn2 | -0,88611 | 2,39E-02 |
| Gigyf1 | -0,53472 | 2,39E-02 |
| Rps6ka5 | -0,89477 | 2,39E-02 |
| Dab1 | -6,62117 | 2,39E-02 |
| Cdc42ep1 | 0,51840 | 2,39E-02 |
| Itgax | -0,70674 | 2,40E-02 |
| Tnfsf8 | -1,61973 | 2,42E-02 |
| Gm28693 | -3,61360 | 2,42E-02 |
| 2900052N01Rik | -4,87200 | 2,46E-02 |
| Rsrp1 | -0,50607 | 2,47E-02 |
| Hsd11b1 | -1,35332 | 2,48E-02 |
| Adrb2 | -1,95298 | 2,48E-02 |
| Cdr2 | 0,73377 | 2,49E-02 |
| Sntb2 | 0,51667 | 2,52E-02 |
| S100a16 | 0,62025 | 2,54E-02 |
| Lama3 | -1,49741 | 2,56E-02 |
| Nkg7 | -2,89871 | 2,57E-02 |
| 6030445D17Rik | 6,56724 | 2,57E-02 |
| 4833407H14Rik | -2,26251 | 2,57E-02 |
| Zfp423 | -1,60552 | 2,57E-02 |
| Ighj1 | -5,81322 | 2,57E-02 |
| Rnasel | -1,18188 | 2,58E-02 |
| 1500015O10Rik | 0,86188 | 2,59E-02 |
| Pdlim7 | 0,63241 | 2,60E-02 |
| Bcl6 | -0,86079 | 2,61E-02 |
| Gapt | -6,61740 | 2,68E-02 |
| Klrd1 | -3,07825 | 2,70E-02 |
| 4930444A19Rik | -2,16320 | 2,70E-02 |
| Nr4a3 | -2,00741 | 2,78E-02 |
| Slc14a1 | -4,08322 | 2,78E-02 |
| Pdcd1 | -3,22765 | 2,80E-02 |
| Naip5 | -1,12295 | 2,80E-02 |
| Cldn4 | 0,82063 | 2,81E-02 |
| Nfkbie | -0,81121 | 2,84E-02 |
| RP23-349H13,2 | -0,89230 | 2,85E-02 |
| Igkv9-120 | -6,59383 | 2,86E-02 |
| Gm17169 | -4,88001 | 2,87E-02 |
| Zdhhc18 | -0,69582 | 2,90E-02 |
| Chsy1 | 0,55203 | 2,92E-02 |
| Xcr1 | -3,70870 | 2,92E-02 |
| Bcl2l11 | -0,45684 | 2,92E-02 |
| Macf1 | -0,48867 | 2,92E-02 |
| Car9 | 1,04555 | 3,03E-02 |
| Apol7c | -6,51548 | 3,08E-02 |
| A630072L19Rik | -2,19197 | 3,08E-02 |
| Pgk1 | 0,65318 | 3,15E-02 |
| Egln1 | 0,54978 | 3,16E-02 |
| Cyp27a1 | -1,58064 | 3,23E-02 |
| Hunk | 0,56100 | 3,25E-02 |
| Glipr2 | -1,40045 | 3,26E-02 |
| Trbv19 | -5,77679 | 3,28E-02 |
| Igkj2 | -5,77679 | 3,28E-02 |
| Kctd7 | 0,86490 | 3,29E-02 |
| Igkv4-72 | -6,71003 | 3,33E-02 |
| BE692007 | -3,48521 | 3,34E-02 |
| Foxk2 | 0,46377 | 3,35E-02 |
| Postn | 0,67019 | 3,39E-02 |
| Trim12c | -0,68087 | 3,39E-02 |
| Perp | 0,60444 | 3,45E-02 |
| Etv4 | 0,65060 | 3,48E-02 |
| Adam19 | -0,76257 | 3,48E-02 |
| Myadm | 0,50863 | 3,48E-02 |
| Apol6 | -0,83525 | 3,61E-02 |
| Traj24 | -6,49751 | 3,63E-02 |
| Klk10 | 1,40723 | 3,64E-02 |
| C6 | -2,77358 | 3,66E-02 |
| Avpr2 | -4,18629 | 3,66E-02 |
| Gm26740 | -4,35650 | 3,66E-02 |
| Icam2 | -1,66310 | 3,68E-02 |
| Ifi30 | -0,59460 | 3,72E-02 |
| Trat1 | -5,04887 | 3,74E-02 |
| Mir142b | -5,82434 | 3,74E-02 |
| Gm6166 | 0,54186 | 3,74E-02 |
| Lyl1 | -1,29954 | 3,76E-02 |
| Krt8 | 0,59105 | 3,76E-02 |
| Gabrp | 0,44796 | 3,76E-02 |
| Xylt1 | -1,26514 | 3,76E-02 |
| Idh3a | 0,53184 | 3,80E-02 |
| Snora21 | 0,47519 | 3,81E-02 |
| Prkd2 | -0,73373 | 3,83E-02 |
| Ighv1-4 | -6,57171 | 3,85E-02 |
| Raver2 | -1,75128 | 3,86E-02 |
| Ppp1r9a | -2,13987 | 3,86E-02 |
| Gnb4 | -0,80103 | 3,87E-02 |
| Chst10 | -0,87814 | 3,94E-02 |
| Galnt7 | -0,90453 | 3,96E-02 |
| Fut7 | -3,87520 | 3,98E-02 |
| F3 | 0,60939 | 4,04E-02 |
| Gm8399 | 0,61983 | 4,04E-02 |
| Hcst | -3,05082 | 4,05E-02 |
| Gm18828 | -6,44385 | 4,05E-02 |
| Cyba | -0,87494 | 4,14E-02 |
| Snx30 | -1,01396 | 4,14E-02 |
| Traj7 | -6,41372 | 4,14E-02 |
| Acer2 | -1,93704 | 4,15E-02 |
| St3gal6 | -1,48043 | 4,16E-02 |
| Adamtsl2 | -1,68025 | 4,16E-02 |
| Hdgfrp3 | -1,32740 | 4,18E-02 |
| Igkv5-48 | -4,81168 | 4,18E-02 |
| Rab6a | 0,48242 | 4,19E-02 |
| Clk1 | -0,42516 | 4,20E-02 |
| Sox21 | 0,72258 | 4,26E-02 |
| Gpr18 | -0,99510 | 4,27E-02 |
| Samd9l | -0,64713 | 4,30E-02 |
| Rasa4 | -1,38913 | 4,30E-02 |
| Ablim1 | -1,65588 | 4,36E-02 |
| Herc6 | -0,77394 | 4,36E-02 |
| Ighg2c | -6,07562 | 4,36E-02 |
| Msmo1 | 0,74930 | 4,40E-02 |
| Zeb1 | -0,66402 | 4,40E-02 |
| Ddx43 | -2,69314 | 4,47E-02 |
| C1qa | -0,54737 | 4,49E-02 |
| Ptp4a3 | -1,05035 | 4,52E-02 |
| Lama4 | 0,45813 | 4,57E-02 |
| Cd55 | -0,52408 | 4,57E-02 |
| Ccdc125 | -1,96667 | 4,59E-02 |
| Phf11d | -1,10366 | 4,59E-02 |
| Gm12064 | -2,11969 | 4,59E-02 |
| Ptprb | -0,60867 | 4,59E-02 |
| Pdcd1lg2 | -6,38815 | 4,59E-02 |
| Ighv3-6 | -6,38194 | 4,59E-02 |
| Gm16334 | -6,38194 | 4,59E-02 |
| 4833422M21Rik | -6,38815 | 4,59E-02 |
| Klhl14 | -5,61083 | 4,62E-02 |
| Ighv1-72 | -6,72065 | 4,62E-02 |
| Traj40 | -6,40053 | 4,64E-02 |
| S100a14 | 1,31533 | 4,66E-02 |
| Gnl3 | 0,53568 | 4,66E-02 |
| D930015E06Rik | -0,56140 | 4,67E-02 |
| Fermt2 | 0,44266 | 4,67E-02 |
| Plod2 | 0,54493 | 4,67E-02 |
| Gm16333 | -5,56921 | 4,68E-02 |
| Igkv2-109 | -5,56921 | 4,68E-02 |
| Nostrin | -1,61291 | 4,72E-02 |
| Fndc1 | 0,54348 | 4,83E-02 |
| Prss35 | 2,15922 | 4,94E-02 |
| Kynu | -2,08547 | 4,97E-02 |
| Rragb | -3,64331 | 4,97E-02 |
| Gm13833 | -1,07959 | 4,97E-02 |
| Clec4e | -6,35052 | 4,97E-02 |
| Mir142hg | -2,68691 | 4,97E-02 |
| H2-Eb1 | -1,66241 | 4,98E-02 |
| Gstt2 | -1,83602 | 4,99E-02 |
| Errfi1 | 0,53248 | 5,08E-02 |
| Steap4 | -1,01107 | 5,09E-02 |
| Fxyd5 | -0,60840 | 5,13E-02 |
| Fzd5 | 0,50701 | 5,13E-02 |
| Ighv1-81 | -5,60332 | 5,15E-02 |
| Igkv4-57-1 | -6,37041 | 5,17E-02 |
| 1810055G02Rik | 0,79697 | 5,17E-02 |
| Insrr | -4,16495 | 5,18E-02 |
| Rasgef1a | -6,32378 | 5,34E-02 |
| Traj18 | -4,18957 | 5,34E-02 |
| Ighv1-80 | -8,39278 | 5,36E-02 |
| Col6a6 | -2,62026 | 5,37E-02 |
| Dstn | 0,53715 | 5,38E-02 |
| Cd27 | -1,07566 | 5,40E-02 |
| Spata31d1b | -6,31729 | 5,41E-02 |
| RP23-428M4,4 | -3,00006 | 5,41E-02 |
| Eno1 | 0,50100 | 5,42E-02 |
| RP24-273P12,4 | -0,91329 | 5,44E-02 |
| Ctso | -0,65030 | 5,45E-02 |
| Acpp | -2,10554 | 5,54E-02 |
| Prr33 | -2,09166 | 5,54E-02 |
| Igkv4-53 | -6,49167 | 5,56E-02 |
| Pde4b | -0,61421 | 5,58E-02 |
| Lzts1 | -2,79373 | 5,59E-02 |
| Rassf4 | -0,72232 | 5,60E-02 |
| Zmynd8 | -0,60401 | 5,61E-02 |
| Gm25360 | 0,70143 | 5,61E-02 |
| Gm28720 | -4,09533 | 5,61E-02 |
| Ugcg | -0,67474 | 5,61E-02 |
| Unc79 | 2,36920 | 5,62E-02 |
| Flrt3 | 0,65036 | 5,68E-02 |
| Gm29438 | -0,69904 | 5,72E-02 |
| Rere | -0,43422 | 5,75E-02 |
| Fam169b | -4,74697 | 5,76E-02 |
| Slc15a2 | -0,44566 | 5,76E-02 |
| Btla | -0,91520 | 5,76E-02 |
| Gm15356 | -1,83252 | 5,88E-02 |
| Aldoa | 0,43796 | 5,88E-02 |
| Ehd3 | -0,78019 | 5,94E-02 |
| Mif | 0,54007 | 5,94E-02 |
| Gm21971 | -1,67628 | 5,96E-02 |
| Gpsm3 | -1,06991 | 5,96E-02 |
| Rasa3 | -0,60561 | 5,98E-02 |
| Batf | -2,68702 | 5,98E-02 |
| Il17ra | -0,63120 | 6,01E-02 |
| D8Ertd738e | 0,48086 | 6,03E-02 |
| Bsg | 0,49475 | 6,03E-02 |
| Vopp1 | -1,04573 | 6,06E-02 |
| sept-06 | -0,66956 | 6,06E-02 |
| Cmya5 | -0,76828 | 6,08E-02 |
| Samd3 | -3,62970 | 6,08E-02 |
| Ighv9-3 | -6,30320 | 6,08E-02 |
| 5830416P10Rik | -1,34394 | 6,14E-02 |
| Il20rb | 0,50623 | 6,14E-02 |
| Pfkp | 0,45026 | 6,14E-02 |
| St8sia6 | 0,61465 | 6,18E-02 |
| Pnpla7 | -0,58489 | 6,20E-02 |
| Zfp783 | -1,72987 | 6,23E-02 |
| Dusp8 | 1,06334 | 6,28E-02 |
| Cxxc5 | 0,51256 | 6,31E-02 |
| Cyp4f18 | -3,56561 | 6,34E-02 |
| Atf7ip | -0,41516 | 6,36E-02 |
| Emb | 0,64750 | 6,38E-02 |
| Man1c1 | -0,71376 | 6,40E-02 |
| Stat1 | -1,63764 | 6,43E-02 |
| Mgp | 0,73824 | 6,44E-02 |
| RP24-74O18,4 | -1,03988 | 6,46E-02 |
| Tapbp | -0,43038 | 6,48E-02 |
| RP23-388I22,1 | -1,83356 | 6,49E-02 |
| Slc20a1 | 0,51723 | 6,49E-02 |
| Gm9821 | -0,74117 | 6,54E-02 |
| Bche | 0,48682 | 6,56E-02 |
| Pkm | 0,50370 | 6,56E-02 |
| Zmiz1os1 | -0,72619 | 6,58E-02 |
| Decr1 | -1,42565 | 6,59E-02 |
| Ldlr | 0,56864 | 6,61E-02 |
| Pianp | -1,04651 | 6,62E-02 |
| Hdac7 | -0,52889 | 6,66E-02 |
| RP23-145F9,4 | -6,54993 | 6,66E-02 |
| B230319C09Rik | -0,75688 | 6,66E-02 |
| Gltscr1 | -0,52540 | 6,66E-02 |
| Igkv3-2 | -6,24278 | 6,71E-02 |
| Rundc3b | -3,95362 | 6,75E-02 |
| Traj30 | -6,32959 | 6,75E-02 |
| Uqcc2 | 0,55082 | 6,81E-02 |
| Gfra2 | -1,58396 | 6,84E-02 |
| Adra1a | -6,39416 | 6,84E-02 |
| Igkj3 | -6,39416 | 6,84E-02 |
| Akr1cl | -5,44976 | 6,85E-02 |
| Gm16534 | -6,22786 | 6,89E-02 |
| Mcoln3 | -6,24852 | 6,91E-02 |
| Tesc | 0,67824 | 6,95E-02 |
| Gm16340 | -1,45970 | 6,95E-02 |
| Rrs1 | 0,51815 | 7,00E-02 |
| Igkj4 | -6,25537 | 7,00E-02 |
| Gm22206 | -6,25537 | 7,00E-02 |
| Igkv9-124 | -3,49702 | 7,00E-02 |
| Flrt1 | -1,28235 | 7,01E-02 |
| Gpr183 | -0,95004 | 7,01E-02 |
| Samd5 | 0,80499 | 7,07E-02 |
| Pgk1-rs7 | 0,73411 | 7,08E-02 |
| n-R5s124 | 0,48860 | 7,08E-02 |
| Pdk4 | -3,76511 | 7,13E-02 |
| Lepr | -1,81013 | 7,20E-02 |
| Znf41-ps | -1,80202 | 7,20E-02 |
| Gm15326 | -2,51323 | 7,20E-02 |
| Fam189b | -1,51249 | 7,24E-02 |
| Trbv29 | -4,56820 | 7,24E-02 |
| Ntng2 | -1,87258 | 7,26E-02 |
| Gm15472 | -3,86293 | 7,26E-02 |
| Plekho1 | -0,62782 | 7,26E-02 |
| Tubb2a | 0,52470 | 7,28E-02 |
| Ntrk1 | -5,52070 | 7,29E-02 |
| Foxred2 | -3,35076 | 7,39E-02 |
| Yaf2 | 0,51179 | 7,39E-02 |
| Gm27201 | -3,32377 | 7,39E-02 |
| Tstd3 | -2,28479 | 7,41E-02 |
| Krt7 | 0,56450 | 7,42E-02 |
| C1ra | -0,65533 | 7,44E-02 |
| Csmd1 | -4,30615 | 7,44E-02 |
| Cep250 | -0,50162 | 7,45E-02 |
| Ncf1 | -0,85089 | 7,47E-02 |
| Gm5873 | 0,86262 | 7,47E-02 |
| Oas3 | -2,00569 | 7,51E-02 |
| Trib2 | 0,48173 | 7,56E-02 |
| Igkv6-32 | -6,27589 | 7,56E-02 |
| Gm13889 | -5,36703 | 7,56E-02 |
| Lpar1 | -1,12756 | 7,59E-02 |
| Klri1 | -4,53696 | 7,64E-02 |
| Ighv1-84 | -6,37674 | 7,67E-02 |
| Gna15 | -1,50598 | 7,74E-02 |
| RP24-308L17,5 | -0,91496 | 7,74E-02 |
| Gm16158 | -5,48604 | 7,82E-02 |
| Pim1 | -0,58377 | 7,83E-02 |
| Scn7a | -0,83144 | 7,85E-02 |
| Pkib | -1,54006 | 7,85E-02 |
| Prom1 | 0,60204 | 7,86E-02 |
| Flt3l | -1,46207 | 7,86E-02 |
| Vmn2r96 | -3,33803 | 7,87E-02 |
| Nop58 | 0,44399 | 7,93E-02 |
| Chrdl1 | -2,90949 | 7,93E-02 |
| Gm19585 | -4,03678 | 7,93E-02 |
| Entpd1 | -0,60445 | 7,96E-02 |
| Calcrl | -0,78221 | 7,98E-02 |
| Gmip | -0,69356 | 8,00E-02 |
| Ptafr | -1,09895 | 8,00E-02 |
| Traj21 | -6,45635 | 8,00E-02 |
| Gm12070 | 0,61057 | 8,11E-02 |
| Map3k14 | -0,57610 | 8,11E-02 |
| Mafb | -0,46510 | 8,11E-02 |
| Nr4a2 | -0,88207 | 8,11E-02 |
| Fam115a | 0,47763 | 8,18E-02 |
| Vcl | 0,48709 | 8,20E-02 |
| Snx29 | -0,69685 | 8,20E-02 |
| Ammecr1 | 0,49891 | 8,22E-02 |
| Tcrg-C2 | -5,33438 | 8,24E-02 |
| Gm27240 | -1,41105 | 8,24E-02 |
| Fnbp1l | 0,42444 | 8,24E-02 |
| Snn | -0,64275 | 8,24E-02 |
| Gm7967 | -0,51659 | 8,25E-02 |
| Trbv24 | -5,45434 | 8,25E-02 |
| Gm16685 | -3,14828 | 8,26E-02 |
| Cpt1a | -0,71648 | 8,37E-02 |
| Gm26799 | -1,95895 | 8,40E-02 |
| Lcp1 | -0,50949 | 8,42E-02 |
| Crlf3 | -0,71788 | 8,42E-02 |
| Ighv1-67 | -6,17135 | 8,47E-02 |
| Steap3 | 0,56185 | 8,48E-02 |
| Gm20645 | -0,93624 | 8,51E-02 |
| 5830468F06Rik | -6,22011 | 8,51E-02 |
| RP23-459L15,8 | -0,83504 | 8,55E-02 |
| Nr1h3 | -0,87837 | 8,60E-02 |
| Acot9 | 0,56546 | 8,60E-02 |
| Sema3b | 0,68631 | 8,60E-02 |
| Gm7678 | -4,38088 | 8,66E-02 |
| Cers6 | -0,73158 | 8,70E-02 |
| Mef2d | -0,40225 | 8,70E-02 |
| Slc25a5 | 0,45758 | 8,70E-02 |
| Pik3r3 | 0,67167 | 8,70E-02 |
| Pglyrp1 | 0,65829 | 8,70E-02 |
| Igkv13-84 | -5,70012 | 8,76E-02 |
| Ighv6-3 | -6,40718 | 8,80E-02 |
| Cblb | -0,65651 | 8,82E-02 |
| Nipal2 | 0,51616 | 8,88E-02 |
| Bckdhb | -1,44788 | 8,88E-02 |
| Gm5559 | 0,65512 | 8,89E-02 |
| Lrrc18 | -2,41486 | 8,93E-02 |
| Traj16 | -6,13254 | 8,96E-02 |
| Dzip1 | -1,05410 | 8,97E-02 |
| Gm7809 | 0,58828 | 8,99E-02 |
| Ighv1-22 | -5,32477 | 9,02E-02 |
| Col27a1 | -0,65781 | 9,06E-02 |
| Slc7a10 | -6,11017 | 9,07E-02 |
| Gm26541 | -2,08315 | 9,08E-02 |
| Cyth4 | -0,68914 | 9,13E-02 |
| Rnu3a | 0,42210 | 9,13E-02 |
| Trav14-1 | -6,14734 | 9,13E-02 |
| Tpm1 | 0,43753 | 9,14E-02 |
| Sema3d | 0,55577 | 9,15E-02 |
| Gm15675 | -0,94099 | 9,15E-02 |
| Smox | 0,60275 | 9,16E-02 |
| Gm9208 | -5,42251 | 9,16E-02 |
| Ssh2 | -0,39571 | 9,24E-02 |
| Atp2b4 | -0,89110 | 9,31E-02 |
| Abi3 | -1,16384 | 9,32E-02 |
| Coch | -1,58245 | 9,33E-02 |
| Panx1 | -1,77068 | 9,36E-02 |
| Zfp382 | -0,99461 | 9,36E-02 |
| Tnn | 3,46859 | 9,37E-02 |
| Gm1840 | 0,42498 | 9,37E-02 |
| Bach2it1 | -2,29935 | 9,40E-02 |
| Lmna | 0,46071 | 9,48E-02 |
| Casz1 | -0,57189 | 9,49E-02 |
| BC100451 | -1,40322 | 9,49E-02 |
| Jak1 | -0,40078 | 9,50E-02 |
| Fscn1 | -0,57634 | 9,50E-02 |
| H2-DMb1 | -0,76249 | 9,50E-02 |
| Dram1 | -0,99795 | 9,52E-02 |
| Morc4 | 0,53418 | 9,52E-02 |
| Jkamp | 0,57176 | 9,59E-02 |
| Ppm1e | -1,29656 | 9,60E-02 |
| Akap12 | 0,43169 | 9,61E-02 |
| Fam214a | -0,48104 | 9,63E-02 |
| Gcnt2 | -1,11944 | 9,69E-02 |
| Sptssa | 0,53031 | 9,71E-02 |
| Ncr1 | -2,95832 | 9,71E-02 |
| Gpr114 | -0,97533 | 9,72E-02 |
| Tigit | -2,09568 | 9,76E-02 |
| Tubb6 | 0,67592 | 9,78E-02 |
| Tlr9 | -1,14400 | 9,79E-02 |
| Robo2 | -1,68170 | 9,85E-02 |
| Zfp141 | -1,62584 | 9,89E-02 |
| RP24-329K22,1 | 6,15008 | 9,93E-02 |
| Pcdh12 | 0,66268 | 9,94E-02 |
| Ncoa3 | -0,40915 | 9,94E-02 |
| Plxna4 | -1,11550 | 9,94E-02 |
| RP24-271M20,4 | -0,79817 | 9,98E-02 |
| Atcay | -3,69755 | 9,98E-02 |
| Klra1 | -3,89207 | 9,98E-02 |
| Me1 | 0,47743 | 1,00E-01 |
| Scn2b | -2,15650 | 1,00E-01 |
| Atxn7l1 | -0,59629 | 1,01E-01 |
| Gdap10 | -0,74294 | 1,01E-01 |
| Oprm1 | -3,37362 | 1,02E-01 |
| Gm14453 | -3,40487 | 1,02E-01 |
| Gm10801 | 1,37667 | 1,02E-01 |
| Ighv1-50 | -6,07086 | 1,02E-01 |
| A530032D15Rik | -2,54247 | 1,02E-01 |
| Traj14 | -6,06311 | 1,02E-01 |
| Eno1b | 0,53275 | 1,03E-01 |
| Gm12185 | -2,16125 | 1,03E-01 |
| Blk | -5,34564 | 1,03E-01 |
| Fcgr2b | -0,79303 | 1,03E-01 |
| Fam83a | 0,51766 | 1,04E-01 |
| Tnf | -1,92833 | 1,04E-01 |
| Cd97 | -0,63722 | 1,05E-01 |
| Cmas | 0,44355 | 1,05E-01 |
| Zfp395 | -0,38251 | 1,05E-01 |
| 1600014C10Rik | -0,64835 | 1,05E-01 |
| Klra2 | -1,00039 | 1,05E-01 |
| Fam180a | -4,30396 | 1,05E-01 |
| Tfap2c | 0,42793 | 1,05E-01 |
| Traj4 | -6,09398 | 1,05E-01 |
| Igkv3-12 | -6,09398 | 1,05E-01 |
| Fgd2 | -0,86032 | 1,05E-01 |
| Epha2 | 0,39378 | 1,05E-01 |
| Ctsz | -0,69602 | 1,05E-01 |
| Batf2 | -1,34959 | 1,05E-01 |
| Ighv1-19 | -6,03966 | 1,05E-01 |
| RP23-125I14,1 | -0,81457 | 1,05E-01 |
| Tnfaip8l2 | -1,08230 | 1,05E-01 |
| Gm12216 | -1,08918 | 1,06E-01 |
| Matn2 | 0,67993 | 1,06E-01 |
| Gzma | -6,30353 | 1,06E-01 |
| Myc | 0,40188 | 1,06E-01 |
| P2ry2 | 0,83634 | 1,06E-01 |
| Cyp4v3 | -0,80800 | 1,07E-01 |
| Gm2245 | -1,95179 | 1,07E-01 |
| Spryd7 | 0,56593 | 1,07E-01 |
| Abce1 | 0,43000 | 1,07E-01 |
| Tnfrsf25 | -1,61528 | 1,08E-01 |
| Rab31 | 0,59844 | 1,08E-01 |
| Scn3b | -3,02463 | 1,08E-01 |
| Cav2 | 0,58584 | 1,09E-01 |
| Dsc2 | 0,43263 | 1,09E-01 |
| Spsb4 | 0,69100 | 1,10E-01 |
| Rassf5 | -0,57894 | 1,10E-01 |

**Appendix Table S4 Differential gene expression in TNC high (WT/shC) in comparison to TNC low (WT/shTNC) tumors upon PBS treatment (4 weeks model)**

RNA sequencing data, adj p-value < 0.1, N = 2.

| **Gene Symbol** | **logFC** | **adj. P value** |
| --- | --- | --- |
| Nxf3 | 6,7551 | 6,06E-107 |
| Nrxn1 | 3,3936 | 2,07E-61 |
| 1500015O10Rik | 3,1905 | 2,55E-55 |
| Glycam1 | -8,5872 | 7,81E-46 |
| Emid1 | -2,7315 | 6,79E-45 |
| H2-Q6 | -2,7460 | 4,17E-37 |
| Dlgap1 | 2,0277 | 1,27E-36 |
| Slfn4 | 3,4843 | 2,65E-36 |
| BC021891 | -3,6796 | 3,37E-34 |
| Nckap5 | -5,1271 | 5,23E-34 |
| Tfap2b | -2,8249 | 4,67E-32 |
| Igj | -3,8527 | 8,75E-32 |
| Bank1 | -5,7209 | 2,40E-31 |
| Soga3 | -4,6610 | 2,40E-31 |
| Ighm | -3,3329 | 1,03E-29 |
| Ttn | -3,5035 | 2,53E-28 |
| Nt5c3b | -2,5373 | 1,12E-23 |
| Akr1c12 | 4,1338 | 1,53E-23 |
| Ighg1 | -6,6553 | 1,31E-22 |
| Moxd1 | -4,2261 | 1,88E-22 |
| Lrrk2 | -3,2257 | 2,14E-22 |
| Veph1 | 1,6455 | 1,35E-21 |
| RP23-12D3,3 | -5,2157 | 1,82E-21 |
| Akr1c13 | 2,3289 | 3,61E-21 |
| Sparcl1 | 1,1433 | 2,55E-18 |
| Cd79a | -6,0710 | 3,05E-18 |
| H2-Q7 | -1,9136 | 4,21E-17 |
| Chst3 | -4,9776 | 7,42E-17 |
| Skap1 | -4,4856 | 8,01E-17 |
| Myo5c | -1,1929 | 9,89E-17 |
| Tbc1d10c | -3,6379 | 2,49E-16 |
| Lck | -3,2289 | 3,24E-16 |
| H2-D1 | -1,2361 | 3,33E-16 |
| Il27ra | -6,0457 | 4,94E-16 |
| H2-Q4 | -1,4657 | 5,46E-16 |
| Trp63 | 1,8329 | 5,79E-16 |
| Ighg2b | -4,8902 | 9,16E-16 |
| H2-Ob | -3,4865 | 9,25E-16 |
| Gm28321 | 3,4774 | 1,06E-15 |
| Gimap3 | -3,8630 | 1,19E-15 |
| Fam78a | -3,4059 | 1,85E-15 |
| Tnfrsf13c | -3,6664 | 5,71E-15 |
| Chil1 | 1,1090 | 7,62E-15 |
| Zfp518a | -2,0871 | 1,13E-14 |
| Ets1 | -1,3878 | 1,29E-14 |
| sept-01 | -3,4224 | 1,35E-14 |
| B2m | -1,1196 | 2,02E-14 |
| Ccr7 | -4,9579 | 2,30E-14 |
| Pogk | -2,4136 | 2,31E-14 |
| Cd2 | -3,6199 | 2,43E-14 |
| Ralgps2 | -2,1852 | 2,76E-14 |
| Cd79b | -7,0437 | 5,20E-14 |
| 9930111J21Rik2 | -2,3137 | 6,05E-14 |
| Slamf6 | -3,4011 | 8,10E-14 |
| C530050E15Rik | -5,1794 | 8,10E-14 |
| Sash3 | -2,7057 | 8,86E-14 |
| RP24-146B4,2 | -5,9822 | 9,22E-14 |
| 4930500J02Rik | 4,4428 | 1,19E-13 |
| Stap1 | -4,1904 | 1,65E-13 |
| Aff3 | -2,9852 | 2,10E-13 |
| Siglecg | -4,5977 | 2,34E-13 |
| Spib | -4,0080 | 2,37E-13 |
| Lgr6 | -1,3287 | 3,26E-13 |
| Myo1g | -2,5177 | 1,22E-12 |
| Psmb8 | -1,5902 | 1,22E-12 |
| Cmah | -2,8950 | 1,51E-12 |
| Fscn1 | -1,7587 | 1,58E-12 |
| Tespa1 | -5,1565 | 2,22E-12 |
| Birc3 | -2,6783 | 2,40E-12 |
| Slc5a3 | 0,9142 | 2,76E-12 |
| Lax1 | -4,1690 | 2,88E-12 |
| Lrmp | -2,7789 | 3,15E-12 |
| Fmnl1 | -1,9830 | 4,14E-12 |
| Ar | -2,4495 | 5,12E-12 |
| Dsc3 | -5,9306 | 7,92E-12 |
| Rassf2 | -2,0484 | 1,07E-11 |
| Adcy1 | -1,3981 | 1,39E-11 |
| Cd209b | -3,7177 | 1,61E-11 |
| Fcer2a | -2,6658 | 1,63E-11 |
| Klhl6 | -2,8381 | 1,97E-11 |
| Fam169b | -4,8020 | 2,04E-11 |
| Stk17b | -2,0541 | 2,25E-11 |
| Csmd1 | -4,0700 | 2,25E-11 |
| Ppp1r16b | -2,7484 | 2,66E-11 |
| Ccnd2 | -2,0474 | 3,52E-11 |
| Galnt6 | -3,1218 | 3,76E-11 |
| F3 | 1,1500 | 3,84E-11 |
| Il21r | -2,5188 | 3,85E-11 |
| P2ry10 | -5,0990 | 3,91E-11 |
| Pde7b | 1,1177 | 4,13E-11 |
| Il16 | -2,4174 | 4,88E-11 |
| H2-K1 | -1,1245 | 5,59E-11 |
| Gm4759 | -4,9485 | 6,36E-11 |
| Bcl11a | -1,5942 | 8,29E-11 |
| Cd74 | -1,0959 | 8,79E-11 |
| Lsp1 | -1,6048 | 9,24E-11 |
| Cd83 | -2,1807 | 1,00E-10 |
| Cacna1e | -3,5901 | 1,27E-10 |
| Cd28 | -4,4671 | 1,27E-10 |
| Ms4a4b | -4,5367 | 1,27E-10 |
| Slc38a1 | -2,7677 | 1,44E-10 |
| Ipcef1 | -2,6944 | 1,65E-10 |
| H2-Aa | -1,1290 | 1,77E-10 |
| 9330159F19Rik | -3,3936 | 1,95E-10 |
| Sh3kbp1 | -1,1636 | 2,03E-10 |
| Cnr2 | -3,9490 | 2,03E-10 |
| Ms4a1 | -10,5214 | 2,22E-10 |
| Col6a1 | -1,0321 | 2,24E-10 |
| Ebf1 | -2,2761 | 3,25E-10 |
| Grap2 | -2,9324 | 4,03E-10 |
| Fam208a | -1,7887 | 4,18E-10 |
| Psmb9 | -1,5534 | 4,27E-10 |
| Zfhx4 | -1,9552 | 5,11E-10 |
| Fam196b | -2,9024 | 5,23E-10 |
| Otoa | 2,0259 | 6,12E-10 |
| Gpr174 | -4,7082 | 6,25E-10 |
| Cd247 | -3,2913 | 6,35E-10 |
| Rhoh | -3,1571 | 6,41E-10 |
| Papln | 0,8046 | 6,74E-10 |
| Samsn1 | -3,8173 | 7,39E-10 |
| Lat | -3,2257 | 7,39E-10 |
| Col6a3 | -0,9914 | 8,51E-10 |
| RP24-272N10,6 | 2,5320 | 8,51E-10 |
| Pigr | -1,0205 | 8,52E-10 |
| Gimap8 | -2,2689 | 9,51E-10 |
| Pdcd4 | 0,7953 | 9,72E-10 |
| Laptm5 | -1,4692 | 1,03E-09 |
| Epsti1 | -2,1590 | 1,04E-09 |
| Ccl5 | -3,0583 | 1,23E-09 |
| Faim3 | -10,2641 | 1,32E-09 |
| Trbc1 | -3,8972 | 1,40E-09 |
| Zfp385b | 2,0700 | 1,48E-09 |
| Cd200 | 1,1309 | 1,80E-09 |
| Fry | 0,8332 | 1,83E-09 |
| Col6a2 | -1,0564 | 1,93E-09 |
| Traf1 | -2,7878 | 1,93E-09 |
| Ptprcap | -2,9405 | 2,35E-09 |
| Grap | -3,1835 | 2,98E-09 |
| Tgtp2 | -2,1466 | 3,16E-09 |
| B3gnt5 | -5,3756 | 3,36E-09 |
| Nr3c2 | -2,4738 | 3,37E-09 |
| Itgb2 | -1,3858 | 3,49E-09 |
| Serpinb6b | -3,8534 | 3,51E-09 |
| Slfn1 | -3,5377 | 3,55E-09 |
| Blk | -6,4744 | 3,58E-09 |
| Dnase1l3 | -2,7429 | 3,87E-09 |
| Col3a1 | -0,7823 | 4,03E-09 |
| RP23-373D16,4 | -4,7789 | 4,06E-09 |
| Clec2i | -4,3250 | 4,23E-09 |
| Ptgds | 1,1756 | 5,25E-09 |
| 9930111J21Rik1 | -2,5144 | 5,26E-09 |
| Zeb1 | -1,4148 | 5,34E-09 |
| Cd8b1 | -3,7175 | 5,78E-09 |
| Cxcr5 | -3,0360 | 6,34E-09 |
| Bsg | 0,9884 | 6,34E-09 |
| Cd19 | -9,7606 | 6,73E-09 |
| Vav1 | -1,6389 | 6,73E-09 |
| Slc4a8 | -3,7854 | 6,84E-09 |
| Igkv6-15 | -6,3698 | 7,39E-09 |
| Ankrd44 | -1,7237 | 7,49E-09 |
| Pip4k2a | -1,5630 | 8,32E-09 |
| Fcgbp | 2,9870 | 9,25E-09 |
| Ly9 | -2,5022 | 1,06E-08 |
| Fli1 | -1,8024 | 1,10E-08 |
| Cd53 | -1,9291 | 1,29E-08 |
| Man1a | -1,3714 | 1,31E-08 |
| Osbpl6 | 1,7077 | 1,33E-08 |
| Enpp2 | -4,7801 | 1,44E-08 |
| Postn | -1,1781 | 1,65E-08 |
| Elf4 | -1,8973 | 1,70E-08 |
| Timd4 | -3,7451 | 1,74E-08 |
| Six1 | 0,9154 | 1,96E-08 |
| Zap70 | -4,9446 | 2,07E-08 |
| Cited4 | -5,3968 | 2,24E-08 |
| Il2rg | -2,8723 | 2,26E-08 |
| Igha | -2,1007 | 2,48E-08 |
| Trac | -2,9572 | 2,51E-08 |
| Icosl | -2,1227 | 2,64E-08 |
| Myh10 | -1,0362 | 2,69E-08 |
| Cilp | -0,9690 | 2,83E-08 |
| Nxpe3 | -3,2583 | 3,45E-08 |
| Plxna1 | -1,1884 | 3,80E-08 |
| Lyz2 | -1,1710 | 4,15E-08 |
| Il9r | -9,5183 | 4,28E-08 |
| Slamf7 | -2,8875 | 5,30E-08 |
| Tap1 | -1,2707 | 5,39E-08 |
| H2-Ab1 | -1,0058 | 5,41E-08 |
| RP24-490B17,7 | -4,2746 | 5,76E-08 |
| B3galt5 | -1,4468 | 5,94E-08 |
| Grem1 | -9,5455 | 5,94E-08 |
| Treml2 | -3,5083 | 6,34E-08 |
| Gimap1 | -2,8173 | 6,39E-08 |
| Map4k1 | -2,4722 | 6,66E-08 |
| Lrp8 | -1,9450 | 6,83E-08 |
| Gm17229 | 1,8486 | 7,14E-08 |
| Cd52 | -2,3321 | 7,90E-08 |
| Dnaic2 | 3,5031 | 8,21E-08 |
| Notch1 | -0,8429 | 9,24E-08 |
| H2-K2 | -1,6360 | 1,04E-07 |
| Trbc2 | -3,2721 | 1,24E-07 |
| Pydc3 | -3,6657 | 1,26E-07 |
| Gabrp | 0,8218 | 1,29E-07 |
| Sell | -6,2293 | 1,43E-07 |
| Unc79 | 3,5514 | 1,62E-07 |
| Cd84 | -1,6552 | 1,69E-07 |
| 5031415H12Rik | 2,3979 | 1,75E-07 |
| Agt | 1,1426 | 1,82E-07 |
| Prkcb | -1,8572 | 1,82E-07 |
| Dnah8 | -4,8360 | 1,89E-07 |
| D8Ertd82e | 0,8352 | 2,00E-07 |
| Plxnc1 | -1,6888 | 2,00E-07 |
| Gimap7 | -3,3290 | 2,21E-07 |
| Selplg | -1,9634 | 2,32E-07 |
| Ptpn22 | -2,3978 | 2,37E-07 |
| Dpysl5 | -1,4473 | 2,38E-07 |
| S1pr1 | -1,7963 | 2,38E-07 |
| Plekha6 | -1,5978 | 2,59E-07 |
| Irf4 | -3,0101 | 2,66E-07 |
| Gimap5 | -3,0858 | 2,79E-07 |
| A630023P12Rik | -4,7179 | 2,80E-07 |
| Ighg2c | -2,9036 | 2,82E-07 |
| Qsox1 | 0,8880 | 2,82E-07 |
| Orai2 | -2,5134 | 2,94E-07 |
| Satb1 | -3,3962 | 2,95E-07 |
| Tspan32 | -1,9126 | 3,06E-07 |
| Inpp5d | -1,3511 | 3,06E-07 |
| Plcb2 | -1,7266 | 3,14E-07 |
| Col9a1 | 0,7353 | 3,35E-07 |
| Ero1l | 1,2489 | 3,42E-07 |
| Slc9a7 | -3,2889 | 3,68E-07 |
| Irf8 | -1,5737 | 3,73E-07 |
| Slco5a1 | -5,1573 | 3,91E-07 |
| Shank1 | -3,2261 | 5,00E-07 |
| Tcf7 | -3,4918 | 5,15E-07 |
| Paqr6 | 1,0821 | 5,40E-07 |
| Nsg2 | -5,5487 | 5,47E-07 |
| RP24-490B17,2 | -9,1597 | 5,72E-07 |
| Dcn | -1,1187 | 5,78E-07 |
| Filip1l | -1,1220 | 5,83E-07 |
| Steap4 | -2,0593 | 6,12E-07 |
| Trim47 | -1,6210 | 6,44E-07 |
| 2010016I18Rik | -1,4245 | 6,89E-07 |
| Acpp | -3,4013 | 7,28E-07 |
| Cyfip2 | -3,1170 | 7,30E-07 |
| Adora1 | 1,2481 | 7,30E-07 |
| Pde3b | -1,7984 | 7,70E-07 |
| Pyhin1 | -2,5791 | 7,90E-07 |
| Fut9 | -2,1977 | 8,33E-07 |
| Dclk1 | -1,9647 | 8,43E-07 |
| H2-M3 | -1,7749 | 8,67E-07 |
| Ghr | 0,7604 | 8,75E-07 |
| Was | -2,1041 | 8,80E-07 |
| Slamf1 | -5,9750 | 9,09E-07 |
| Arhgap25 | -1,8890 | 9,61E-07 |
| Ctse | -2,4948 | 1,02E-06 |
| Chst15 | -1,6835 | 1,13E-06 |
| Tgfb3 | 0,6873 | 1,24E-06 |
| Apoe | -0,7547 | 1,25E-06 |
| Rbm38 | -2,2090 | 1,25E-06 |
| Cd40 | -2,9100 | 1,27E-06 |
| Apbb1ip | -1,6111 | 1,35E-06 |
| Vegfa | 0,9094 | 1,35E-06 |
| Ptpru | -1,4616 | 1,44E-06 |
| Kcnab2 | -2,1192 | 1,44E-06 |
| Cd3d | -4,1405 | 1,45E-06 |
| Cd163 | 1,9896 | 1,45E-06 |
| Col1a2 | -0,8511 | 1,59E-06 |
| H2-M2 | -2,0478 | 1,61E-06 |
| Icos | -3,8597 | 1,81E-06 |
| Ccdc149 | -1,8363 | 1,81E-06 |
| Igkc | -4,1682 | 1,87E-06 |
| Lfng | -1,7382 | 1,91E-06 |
| Boc | 0,7238 | 1,99E-06 |
| Dusp2 | -2,7348 | 2,15E-06 |
| Pacsin1 | -3,1821 | 2,15E-06 |
| Rbm20 | 2,0873 | 2,18E-06 |
| Mical1 | -1,9195 | 2,18E-06 |
| RP24-444I15,4 | -4,1814 | 2,18E-06 |
| Dll1 | 0,6805 | 2,19E-06 |
| Cyp2d9 | 1,7002 | 2,23E-06 |
| RP24-146B4,3 | -3,0477 | 2,31E-06 |
| Ubash3a | -3,5517 | 2,32E-06 |
| Thbs1 | 0,9518 | 2,35E-06 |
| Runx1 | -0,7854 | 2,48E-06 |
| H2-Eb2 | -4,7753 | 2,56E-06 |
| Igtp | -1,3451 | 2,56E-06 |
| Sdc2 | -0,7321 | 2,65E-06 |
| AI467606 | -2,5824 | 2,65E-06 |
| Gyk | 1,2457 | 2,66E-06 |
| March1 | -1,7453 | 2,66E-06 |
| Sulf1 | -1,3221 | 2,80E-06 |
| S100b | 1,2868 | 2,81E-06 |
| Pfkp | 0,7524 | 2,90E-06 |
| Trim9 | 2,6403 | 3,12E-06 |
| Gm11346 | -6,7473 | 3,20E-06 |
| A230050P20Rik | -2,2827 | 3,30E-06 |
| Bmp7 | 1,9410 | 3,43E-06 |
| Ica1 | -2,4275 | 3,63E-06 |
| Fermt3 | -1,2910 | 3,88E-06 |
| Prkcq | -4,7163 | 4,17E-06 |
| Ceacam10 | 1,2230 | 4,27E-06 |
| Rftn1 | -1,5817 | 4,40E-06 |
| Zfp608 | 0,9276 | 4,63E-06 |
| Mt1 | -1,5921 | 4,76E-06 |
| Ikzf3 | -4,1959 | 5,03E-06 |
| Calcrl | -1,4667 | 5,09E-06 |
| St8sia4 | -1,5974 | 5,19E-06 |
| Tnfaip3 | -1,3507 | 5,29E-06 |
| Ccl22 | -3,1134 | 5,29E-06 |
| Rin3 | -1,3499 | 5,40E-06 |
| Pou6f1 | -1,3615 | 5,80E-06 |
| Mme | 0,6545 | 5,81E-06 |
| Fam65b | -1,3267 | 5,81E-06 |
| Fam49a | -1,6835 | 5,82E-06 |
| Dpp4 | -2,4139 | 5,91E-06 |
| Cd22 | -4,8595 | 5,95E-06 |
| Sdc3 | -0,8217 | 6,09E-06 |
| Rgs1 | -2,3911 | 6,40E-06 |
| Nabp1 | 0,7113 | 6,41E-06 |
| Cxcr4 | -0,8978 | 6,64E-06 |
| H2-Q10 | -1,4406 | 6,97E-06 |
| Itk | -4,4442 | 7,08E-06 |
| Csf2rb | -1,2554 | 7,26E-06 |
| Agap2 | -1,9080 | 7,28E-06 |
| F730311O21Rik | -6,4056 | 7,48E-06 |
| Slc39a8 | 1,1272 | 8,08E-06 |
| Plcl2 | -1,9207 | 8,12E-06 |
| Marco | -8,0164 | 8,16E-06 |
| sept-06 | -1,0479 | 8,16E-06 |
| Igkv12-46 | -8,4244 | 8,16E-06 |
| Tril | 0,8231 | 8,37E-06 |
| S1pr4 | -4,5174 | 9,04E-06 |
| Spn | -3,2521 | 9,45E-06 |
| Tnfrsf22 | -2,3911 | 9,52E-06 |
| Igkv10-96 | -4,5742 | 9,52E-06 |
| RP24-490B17,6 | -3,2623 | 9,88E-06 |
| Plcb4 | -1,8185 | 1,02E-05 |
| Arrdc4 | 0,7334 | 1,02E-05 |
| Cp | 0,7983 | 1,03E-05 |
| Pla2g4a | -0,9681 | 1,04E-05 |
| Dtx1 | -1,5956 | 1,05E-05 |
| H2-DMb2 | -1,6082 | 1,05E-05 |
| F830016B08Rik | -1,6072 | 1,05E-05 |
| Serpinb1a | -3,2911 | 1,08E-05 |
| Ifi47 | -1,2945 | 1,09E-05 |
| Jak1 | -0,6689 | 1,12E-05 |
| Kcnj10 | -1,7521 | 1,12E-05 |
| Sh3pxd2a | -0,6623 | 1,12E-05 |
| Pglyrp2 | -5,3637 | 1,12E-05 |
| WI1-2075L23,1 | -5,0381 | 1,12E-05 |
| Dgka | -0,9303 | 1,18E-05 |
| Hs3st1 | 0,7524 | 1,22E-05 |
| Atp1b1 | 0,7618 | 1,24E-05 |
| 9330175E14Rik | -2,2145 | 1,29E-05 |
| Trim35 | -1,5142 | 1,41E-05 |
| Slc22a17 | 1,9323 | 1,41E-05 |
| Arhgap29 | 0,8097 | 1,44E-05 |
| Phka1 | 1,2240 | 1,46E-05 |
| Cdh19 | 0,7087 | 1,50E-05 |
| C1qtnf3 | -1,2745 | 1,54E-05 |
| Arhgap9 | -1,9855 | 1,58E-05 |
| Iglc2 | -7,8523 | 1,58E-05 |
| RP23-175B15,2 | -1,8973 | 1,59E-05 |
| Flt3 | -2,5363 | 1,71E-05 |
| Hsd11b1 | -1,9750 | 1,75E-05 |
| Bcor | -1,1011 | 1,78E-05 |
| Clcf1 | -1,8217 | 1,78E-05 |
| Mzb1 | -3,3225 | 1,83E-05 |
| Mpzl2 | 0,7587 | 1,83E-05 |
| RP24-490B17,1 | -4,7911 | 1,84E-05 |
| Fam129a | -1,1360 | 1,84E-05 |
| Stat4 | -2,9748 | 1,86E-05 |
| Emilin1 | -1,3196 | 1,88E-05 |
| Bcl11b | -3,8902 | 1,96E-05 |
| AI504432 | -2,6440 | 1,97E-05 |
| Rps6ka6 | -6,7798 | 2,01E-05 |
| Gm4955 | -6,4740 | 2,02E-05 |
| Fndc9 | -3,7424 | 2,04E-05 |
| Usp11 | -1,4252 | 2,29E-05 |
| Cpne4 | -8,3506 | 2,38E-05 |
| BE692007 | -2,5438 | 2,43E-05 |
| Nt5e | -1,2742 | 2,47E-05 |
| Aebp1 | -0,9141 | 2,48E-05 |
| Igkv1-135 | -6,1100 | 2,51E-05 |
| Tmem56 | 1,2441 | 2,63E-05 |
| Zfp831 | -5,0749 | 2,65E-05 |
| Numb | 0,6192 | 2,65E-05 |
| Antxr1 | -1,0227 | 2,72E-05 |
| H2-Q1 | -1,1244 | 2,74E-05 |
| Ctss | -0,7162 | 2,75E-05 |
| Cd3e | -4,0995 | 2,76E-05 |
| Pla2g2d | -9,7194 | 2,79E-05 |
| Rora | 0,6218 | 2,87E-05 |
| Dusp10 | -2,6151 | 2,87E-05 |
| Sh2d2a | -2,3874 | 2,87E-05 |
| Aim2 | -1,9892 | 2,99E-05 |
| Rgs14 | -2,4316 | 2,99E-05 |
| RP23-68O7,2 | -2,0593 | 3,06E-05 |
| Fcrl1 | -5,9278 | 3,23E-05 |
| Gm8909 | -1,0424 | 3,23E-05 |
| Runx3 | -1,3913 | 3,30E-05 |
| Marveld1 | -1,3892 | 3,62E-05 |
| Camk1d | -1,7763 | 3,91E-05 |
| Ighv3-1 | -8,0851 | 3,91E-05 |
| Asap1 | -0,9684 | 3,92E-05 |
| RP23-373D16,3 | -8,1800 | 4,05E-05 |
| 4930403O15Rik | 2,1456 | 4,24E-05 |
| Lcp2 | -1,5878 | 4,32E-05 |
| Txk | -3,6102 | 4,32E-05 |
| Igkv1-117 | -3,6580 | 4,32E-05 |
| Cxcl12 | -1,3864 | 4,34E-05 |
| AI662270 | -2,2175 | 4,46E-05 |
| Gm26685 | 2,0696 | 4,62E-05 |
| RP23-397E2,4 | -8,2456 | 4,76E-05 |
| RP23-428M4,4 | -8,0506 | 4,78E-05 |
| Srgn | -1,3412 | 4,79E-05 |
| Vim | -0,7508 | 4,89E-05 |
| Pou2af1 | -4,1959 | 4,94E-05 |
| Gcsam | -3,9438 | 4,94E-05 |
| Fam162a | 0,9785 | 5,05E-05 |
| Traf5 | -1,5031 | 5,06E-05 |
| Rgs11 | 0,7569 | 5,09E-05 |
| Adamtsl3 | -2,1591 | 5,20E-05 |
| Mrps6 | 0,7192 | 5,20E-05 |
| Gpr171 | -2,7244 | 5,21E-05 |
| Acap1 | -3,1467 | 5,35E-05 |
| Cplx2 | 0,8050 | 5,43E-05 |
| Cd5 | -2,3712 | 5,48E-05 |
| Rbp7 | 0,8685 | 5,68E-05 |
| Snord17 | -0,7386 | 5,77E-05 |
| Steap2 | -2,6550 | 5,83E-05 |
| Evl | -1,0154 | 5,96E-05 |
| Irf2bp2 | -0,6346 | 6,00E-05 |
| Egln3 | 0,7727 | 6,03E-05 |
| Ccdc88c | -1,1183 | 6,30E-05 |
| Cd37 | -3,5786 | 6,55E-05 |
| H2-T3 | -4,1405 | 6,57E-05 |
| Ppm1m | -1,9769 | 6,62E-05 |
| Basp1 | -0,8237 | 6,65E-05 |
| Nckap1l | -1,1363 | 6,67E-05 |
| Cml2 | 1,9270 | 6,71E-05 |
| Ppap2b | 0,6095 | 6,81E-05 |
| Robo2 | -2,5700 | 6,81E-05 |
| Ciita | -1,0304 | 6,88E-05 |
| Fetub | 1,9295 | 6,88E-05 |
| Ccdc88b | -1,4715 | 6,92E-05 |
| Cemip | 1,5613 | 7,33E-05 |
| Icam1 | -1,5262 | 7,41E-05 |
| Il10ra | -1,0173 | 7,86E-05 |
| Gm16439 | 1,4574 | 8,04E-05 |
| Iigp1 | -0,9713 | 8,41E-05 |
| Dpysl3 | -1,3053 | 8,47E-05 |
| Calml3 | 1,2103 | 8,47E-05 |
| Cyp2d10 | 1,3124 | 8,47E-05 |
| Smtnl2 | 1,3895 | 9,03E-05 |
| Ighd | -6,5415 | 9,09E-05 |
| Atp2a3 | -3,6547 | 9,16E-05 |
| Sfmbt2 | -4,9093 | 9,23E-05 |
| Cecr2 | -2,8736 | 9,53E-05 |
| Cd226 | -3,1458 | 1,00E-04 |
| Pou2f2 | -1,4864 | 1,06E-04 |
| Lama5 | 0,5788 | 1,07E-04 |
| Il2rb | -2,0620 | 1,08E-04 |
| Igkv8-27 | -7,3384 | 1,09E-04 |
| Per1 | -0,7686 | 1,14E-04 |
| Gpr132 | -2,6194 | 1,15E-04 |
| RP23-397E2,5 | -7,8420 | 1,22E-04 |
| Sp110 | -1,1523 | 1,24E-04 |
| Hlf | 0,8509 | 1,24E-04 |
| Btg2 | -1,2110 | 1,26E-04 |
| Fat4 | -1,2356 | 1,30E-04 |
| Acp5 | -0,9063 | 1,32E-04 |
| Gdpd3 | -1,4963 | 1,33E-04 |
| Msn | -0,5590 | 1,36E-04 |
| Tox2 | -3,7698 | 1,38E-04 |
| Tenm4 | 1,0095 | 1,41E-04 |
| Ighv8-8 | -7,4955 | 1,41E-04 |
| Rasgef1b | -0,9991 | 1,43E-04 |
| Ak1 | 0,9358 | 1,43E-04 |
| Dapl1 | -7,8197 | 1,45E-04 |
| Pecam1 | -0,8617 | 1,47E-04 |
| D16Ertd472e | -2,2262 | 1,48E-04 |
| Ldlrad3 | -1,0310 | 1,48E-04 |
| Ppp1r18 | -0,7600 | 1,52E-04 |
| Gpa33 | 4,5669 | 1,56E-04 |
| Fxyd5 | -0,9413 | 1,57E-04 |
| Kmo | -2,3237 | 1,60E-04 |
| Cacna2d4 | -4,0335 | 1,68E-04 |
| Cd3g | -3,3693 | 1,77E-04 |
| Gpr64 | -3,1284 | 1,77E-04 |
| Ighg3 | -7,2924 | 1,80E-04 |
| Col1a1 | -0,7576 | 1,82E-04 |
| Lef1 | -0,7220 | 1,82E-04 |
| Bnip3 | 1,1769 | 1,84E-04 |
| Cdkn1a | 1,1725 | 1,86E-04 |
| Zc3h12d | -1,5305 | 1,88E-04 |
| Peg3 | 0,5888 | 1,89E-04 |
| Arpc1b | -0,6405 | 1,98E-04 |
| Fam107b | -1,3525 | 2,02E-04 |
| Gm19980 | -7,5403 | 2,02E-04 |
| Lyz1 | -2,6024 | 2,03E-04 |
| Sik1 | -0,5778 | 2,04E-04 |
| Tap2 | -0,7626 | 2,15E-04 |
| Clec9a | -2,6789 | 2,16E-04 |
| Cyba | -1,2937 | 2,18E-04 |
| Me1 | 0,7020 | 2,18E-04 |
| Pced1b | -1,2811 | 2,19E-04 |
| Rel | -1,3409 | 2,26E-04 |
| Synpo2 | -1,8381 | 2,33E-04 |
| Ly6d | -4,0936 | 2,39E-04 |
| RP23-307N14,2 | -4,7879 | 2,40E-04 |
| Pdgfra | 0,6911 | 2,43E-04 |
| Zfp275 | -1,9073 | 2,43E-04 |
| Kcnb1 | 0,7440 | 2,43E-04 |
| Cr2 | -4,6328 | 2,44E-04 |
| Kctd12 | -1,3838 | 2,44E-04 |
| Lpxn | -2,4481 | 2,46E-04 |
| Gimap1os | -3,8792 | 2,47E-04 |
| Nav2 | 0,5421 | 2,47E-04 |
| RP23-361M12,2 | -2,6364 | 2,48E-04 |
| Cd24a | -0,6778 | 2,51E-04 |
| Otogl | 3,8522 | 2,52E-04 |
| Dock10 | -0,9787 | 2,52E-04 |
| H2-Oa | -5,2649 | 2,56E-04 |
| Gm14290 | 1,0591 | 2,61E-04 |
| BC035044 | -4,0759 | 2,65E-04 |
| Galnt15 | -0,7368 | 2,79E-04 |
| Dscam | -7,6788 | 2,80E-04 |
| B4galt5 | -1,4694 | 2,80E-04 |
| Capn6 | 1,3991 | 2,82E-04 |
| Rcsd1 | -2,9192 | 2,84E-04 |
| Camk4 | -1,7865 | 2,84E-04 |
| Prex1 | -1,0494 | 3,05E-04 |
| Ighv3-2 | -7,0636 | 3,10E-04 |
| Gpi1 | 0,6235 | 3,11E-04 |
| RP24-88B13,3 | -2,1409 | 3,17E-04 |
| Il22ra2 | -7,0984 | 3,20E-04 |
| Aicda | -5,6888 | 3,21E-04 |
| Slc2a9 | -2,0351 | 3,25E-04 |
| Gm20541 | 0,9857 | 3,27E-04 |
| Col11a1 | 0,5645 | 3,31E-04 |
| Gm25436 | 2,6815 | 3,31E-04 |
| Atp10d | -1,4732 | 3,31E-04 |
| Tlr1 | -2,2161 | 3,35E-04 |
| 1700125H20Rik | 2,1435 | 3,36E-04 |
| Cacna1i | -3,3793 | 3,37E-04 |
| Rgs13 | -7,6968 | 3,42E-04 |
| Gm10277 | 0,9868 | 3,42E-04 |
| Csf2rb2 | -1,1259 | 3,47E-04 |
| Zfp750 | -0,6536 | 3,52E-04 |
| Mreg | -3,2667 | 3,52E-04 |
| Ighj4 | -4,1610 | 3,80E-04 |
| Cd69 | -3,3788 | 3,91E-04 |
| Gm1840 | 0,6159 | 3,98E-04 |
| Sh2d1a | -7,5547 | 3,99E-04 |
| Plcg2 | -0,6468 | 4,04E-04 |
| Pvrl1 | -1,8925 | 4,14E-04 |
| Wnt5a | 1,1455 | 4,15E-04 |
| Spon1 | -0,6526 | 4,28E-04 |
| Sipa1 | -0,9382 | 4,28E-04 |
| Tdrp | -2,8756 | 4,34E-04 |
| Gm12462 | -7,6678 | 4,34E-04 |
| Colec12 | -1,0812 | 4,35E-04 |
| Map7d2 | 1,3452 | 4,36E-04 |
| Abcg1 | -0,9784 | 4,38E-04 |
| Nfkbia | -0,8019 | 4,38E-04 |
| Spg20 | 0,5924 | 4,40E-04 |
| Ifi44 | 0,9107 | 4,40E-04 |
| Arhgap15 | -3,1322 | 4,53E-04 |
| Trbv3 | -7,1492 | 4,55E-04 |
| Sh2d3c | -1,4330 | 4,63E-04 |
| Pcdhga1 | 1,0873 | 4,63E-04 |
| Svip | 1,4077 | 4,66E-04 |
| Mef2c | -1,0055 | 4,76E-04 |
| Tmem71 | -2,2511 | 4,84E-04 |
| Trpv2 | -1,8060 | 5,06E-04 |
| Gm14005 | 0,9620 | 5,07E-04 |
| Neurl3 | -1,3540 | 5,17E-04 |
| Themis | -4,6628 | 5,18E-04 |
| Cst3 | -0,6469 | 5,34E-04 |
| Wdfy4 | -2,4419 | 5,66E-04 |
| Cd72 | -1,0887 | 5,66E-04 |
| Arhgap24 | -1,3089 | 5,72E-04 |
| Enpp5 | 0,6625 | 5,87E-04 |
| Rhoj | -0,8325 | 6,04E-04 |
| Tnfrsf14 | -1,4837 | 6,04E-04 |
| AU018091 | 1,9261 | 6,17E-04 |
| Pcdhga6 | 0,9062 | 6,30E-04 |
| H60b | -2,0853 | 6,38E-04 |
| Fgr | -1,4365 | 6,38E-04 |
| Baz1a | -0,6019 | 6,40E-04 |
| Fbxo32 | 0,5367 | 6,50E-04 |
| Deptor | 0,6409 | 6,50E-04 |
| Pcdh15 | -7,2021 | 6,51E-04 |
| Hmha1 | -2,7871 | 6,52E-04 |
| Eif4b | 0,5007 | 6,53E-04 |
| Gm26551 | -2,7032 | 6,53E-04 |
| Coro1a | -2,0854 | 6,67E-04 |
| Hdgfrp3 | -1,8106 | 6,68E-04 |
| Colq | -5,6915 | 6,68E-04 |
| Ighj3 | -4,5352 | 6,68E-04 |
| Il2ra | -2,1910 | 6,88E-04 |
| Ighe | -6,9953 | 6,94E-04 |
| Fgd3 | -1,3504 | 6,96E-04 |
| Rasgrp3 | -1,5256 | 6,99E-04 |
| 5031414D18Rik | -2,6633 | 7,01E-04 |
| Ubash3b | -1,5764 | 7,03E-04 |
| Scgb2b27 | 2,0245 | 7,12E-04 |
| Ighv5-17 | -3,9693 | 7,12E-04 |
| Cd40lg | -3,4807 | 7,17E-04 |
| Ptprc | -2,1527 | 7,46E-04 |
| Prrx1 | -1,0874 | 7,66E-04 |
| Lmo2 | -1,5996 | 7,66E-04 |
| Wnk4 | 0,6024 | 7,66E-04 |
| Tenm3 | -1,4206 | 7,73E-04 |
| H2-T10 | -0,8233 | 7,73E-04 |
| Traf3ip3 | -3,1398 | 7,77E-04 |
| Hcls1 | -1,0256 | 7,91E-04 |
| Batf2 | -1,5804 | 8,19E-04 |
| Wipf1 | -0,8812 | 8,22E-04 |
| Pgm2 | 0,8159 | 8,23E-04 |
| Tmem123 | -0,5461 | 8,23E-04 |
| Igkv17-121 | -7,3560 | 8,46E-04 |
| Rapgef5 | -1,3225 | 8,50E-04 |
| Ltb | -3,5269 | 8,80E-04 |
| Btk | -1,7787 | 8,80E-04 |
| A430078G23Rik | -4,4069 | 8,80E-04 |
| Rltpr | -1,9952 | 8,98E-04 |
| Rapgef4 | -1,7267 | 8,99E-04 |
| Cd180 | -1,8266 | 9,10E-04 |
| Tmem229a | -1,9385 | 9,21E-04 |
| Col6a5 | -2,3621 | 9,24E-04 |
| Dab2 | 0,5896 | 9,45E-04 |
| Cyth1 | -0,6072 | 9,54E-04 |
| Sema7a | -1,2264 | 9,97E-04 |
| Nlrc5 | -1,8740 | 1,05E-03 |
| Fndc1 | -0,7091 | 1,12E-03 |
| Slc16a6 | -1,3104 | 1,12E-03 |
| RP24-490B17,3 | -7,3641 | 1,14E-03 |
| Ppfia4 | -1,1697 | 1,15E-03 |
| Ccr6 | -1,9998 | 1,15E-03 |
| Itgal | -2,4980 | 1,20E-03 |
| Klf2 | -1,0328 | 1,24E-03 |
| Dock11 | -2,4391 | 1,27E-03 |
| Haao | -2,6367 | 1,29E-03 |
| Rac2 | -2,7478 | 1,29E-03 |
| Parvg | -1,5206 | 1,35E-03 |
| Ehbp1l1 | -0,7358 | 1,40E-03 |
| Rinl | -1,3933 | 1,40E-03 |
| Hexa | -0,8632 | 1,42E-03 |
| Mmp16 | -1,8395 | 1,44E-03 |
| Snx20 | -2,0046 | 1,45E-03 |
| Fam46c | -1,3271 | 1,45E-03 |
| Kif21b | -2,3195 | 1,46E-03 |
| Bin2 | -1,3309 | 1,47E-03 |
| Thbs2 | -0,6017 | 1,51E-03 |
| Gm16340 | -2,1819 | 1,56E-03 |
| Tpi1 | 0,6206 | 1,58E-03 |
| Ppp1r1b | 1,8583 | 1,59E-03 |
| Tns4 | -1,0242 | 1,61E-03 |
| Mamld1 | -1,3284 | 1,61E-03 |
| St3gal4 | -1,4640 | 1,63E-03 |
| Tgfbi | -0,6521 | 1,70E-03 |
| Akna | -2,0764 | 1,70E-03 |
| Cxcl13 | -5,9755 | 1,72E-03 |
| Vmn2r96 | -7,2111 | 1,73E-03 |
| Galnt3 | 0,5879 | 1,76E-03 |
| Folr4 | -3,8716 | 1,78E-03 |
| Gm23455 | -0,8321 | 1,78E-03 |
| Ank | 0,6509 | 1,79E-03 |
| Dusp6 | 0,5886 | 1,81E-03 |
| Myo1f | -0,9548 | 1,82E-03 |
| Pcolce | -0,8361 | 1,82E-03 |
| 9130230L23Rik | 0,8406 | 1,82E-03 |
| RP23-291L24,4 | -4,5332 | 1,84E-03 |
| Cd97 | -1,0090 | 1,84E-03 |
| St8sia1 | -0,7139 | 1,84E-03 |
| Adamts3 | 0,4856 | 1,86E-03 |
| Plod2 | 0,6715 | 1,87E-03 |
| Pik3r5 | -1,3405 | 1,94E-03 |
| AU020206 | -0,9500 | 1,94E-03 |
| Igfals | 0,7146 | 1,96E-03 |
| Zmiz1 | 0,4663 | 1,97E-03 |
| Rasa3 | -0,7632 | 1,97E-03 |
| Aim1l | -1,8296 | 2,12E-03 |
| Lbh | 0,5593 | 2,12E-03 |
| Degs1 | -1,1762 | 2,12E-03 |
| Tnfrsf13b | -1,7426 | 2,13E-03 |
| Helb | -0,8094 | 2,13E-03 |
| Traj6 | -4,2300 | 2,13E-03 |
| Igkv1-110 | -2,9590 | 2,14E-03 |
| Nr1h5 | -7,1384 | 2,14E-03 |
| Igkv6-25 | -7,1734 | 2,18E-03 |
| RP24-88B13,7 | -2,9476 | 2,20E-03 |
| Xlr4b | -3,6927 | 2,22E-03 |
| Cnp | -0,6759 | 2,26E-03 |
| Gga2 | -0,8755 | 2,28E-03 |
| Lgals3 | 0,6561 | 2,28E-03 |
| Slc2a1 | 0,6119 | 2,29E-03 |
| Ifngr1 | -0,6169 | 2,32E-03 |
| Mmp14 | -0,5974 | 2,33E-03 |
| Tprn | 0,7141 | 2,35E-03 |
| Pygo1 | 1,1717 | 2,37E-03 |
| Samhd1 | -0,7503 | 2,42E-03 |
| Ighv1-26 | -4,1598 | 2,45E-03 |
| Rasal3 | -2,7856 | 2,52E-03 |
| Rgs3 | -1,2889 | 2,52E-03 |
| Fut7 | -7,0816 | 2,54E-03 |
| Acot6 | -3,6751 | 2,55E-03 |
| Adam6b | -7,1215 | 2,59E-03 |
| Stab2 | -2,5322 | 2,64E-03 |
| Zfand2a | 0,7622 | 2,73E-03 |
| Ckap4 | 0,7034 | 2,75E-03 |
| Gm10499 | -1,3785 | 2,75E-03 |
| Nuak2 | -1,7531 | 2,83E-03 |
| Smpdl3a | -1,1926 | 2,83E-03 |
| Cd96 | -2,8647 | 2,84E-03 |
| Tox | -2,3357 | 2,89E-03 |
| RP24-490B17,4 | -4,0738 | 2,91E-03 |
| Dock2 | -2,3960 | 2,93E-03 |
| Ptp4a3 | -1,4000 | 2,97E-03 |
| Sema4f | -5,2656 | 2,98E-03 |
| Rassf3 | 0,4973 | 3,02E-03 |
| Traj22 | -4,6956 | 3,05E-03 |
| Mgat4b | 0,6044 | 3,05E-03 |
| Kcna3 | -2,7332 | 3,05E-03 |
| Syk | -2,0958 | 3,08E-03 |
| Fads1 | -1,0520 | 3,10E-03 |
| Pfkfb3 | -0,6786 | 3,15E-03 |
| Nr4a2 | -1,2387 | 3,19E-03 |
| Apobr | 0,6204 | 3,19E-03 |
| Mrc2 | -0,8034 | 3,21E-03 |
| Igkv6-23 | -6,3947 | 3,21E-03 |
| RP23-36P10,10 | -7,0296 | 3,30E-03 |
| Trav3-3 | -7,0780 | 3,30E-03 |
| B3gnt3 | 1,6824 | 3,32E-03 |
| Scnn1g | 1,5808 | 3,34E-03 |
| Myo18a | 0,4494 | 3,34E-03 |
| Igkj5 | -4,6591 | 3,35E-03 |
| Sp100 | -2,3519 | 3,36E-03 |
| Adam23 | -1,1530 | 3,36E-03 |
| Igkv8-30 | -2,9775 | 3,48E-03 |
| Kcnq5 | -4,0732 | 3,51E-03 |
| Tmsb4x | -0,4568 | 3,52E-03 |
| Ly6c2 | -3,8496 | 3,55E-03 |
| Helz2 | -2,3805 | 3,60E-03 |
| Axl | -0,6815 | 3,64E-03 |
| Vopp1 | -1,3741 | 3,65E-03 |
| RP24-444I15,1 | -5,1857 | 3,65E-03 |
| Mpeg1 | -0,5675 | 3,65E-03 |
| Irf5 | -0,9557 | 3,66E-03 |
| Amotl2 | 0,4853 | 3,67E-03 |
| BC094916 | -3,3780 | 3,67E-03 |
| Il4i1 | -1,0631 | 3,69E-03 |
| Gpr158 | 3,2851 | 3,77E-03 |
| Pdgfrl | -1,2700 | 3,77E-03 |
| Car9 | 1,2433 | 3,78E-03 |
| Lpcat1 | 0,5433 | 3,79E-03 |
| Pcdhgb6 | 0,7320 | 3,79E-03 |
| Plekho1 | -0,7365 | 3,81E-03 |
| Sla | -1,1954 | 3,83E-03 |
| Ccnd3 | -0,6737 | 3,84E-03 |
| Igkv3-5 | -7,0951 | 3,89E-03 |
| Gpx3 | -0,7547 | 3,95E-03 |
| Mylk | 0,7091 | 3,95E-03 |
| Hbegf | 1,1641 | 3,95E-03 |
| Lingo2 | 6,3555 | 3,95E-03 |
| Gpr18 | -1,2535 | 3,95E-03 |
| RP24-222G3,1 | 0,9526 | 3,95E-03 |
| Pcdhb14 | -0,9589 | 3,96E-03 |
| Arhgap30 | -2,0891 | 3,97E-03 |
| 2210016F16Rik | -1,9205 | 3,97E-03 |
| Lamc1 | 0,4692 | 4,03E-03 |
| Hmgcs2 | -2,4862 | 4,03E-03 |
| Rnf19b | -0,7960 | 4,03E-03 |
| Igkv5-48 | -7,0985 | 4,04E-03 |
| Kbtbd11 | -3,3474 | 4,06E-03 |
| Igkj2 | -7,0296 | 4,06E-03 |
| Strip2 | -2,9185 | 4,15E-03 |
| Ttc39b | -0,6873 | 4,15E-03 |
| Apobec1 | -0,8645 | 4,15E-03 |
| Cytip | -2,5723 | 4,16E-03 |
| Prtg | 1,0375 | 4,19E-03 |
| Dennd1c | -1,1725 | 4,22E-03 |
| Clec2d | -0,7389 | 4,26E-03 |
| Ctsk | -1,1073 | 4,27E-03 |
| Gm15987 | -2,0840 | 4,27E-03 |
| 4930447C04Rik | 7,0649 | 4,27E-03 |
| Phf11d | -0,8795 | 4,30E-03 |
| Tnfsf8 | -1,9368 | 4,32E-03 |
| Cxcl14 | 1,0987 | 4,32E-03 |
| Plscr2 | 0,5464 | 4,37E-03 |
| Icam2 | -1,9114 | 4,37E-03 |
| RP24-88B13,5 | -2,2608 | 4,38E-03 |
| Dse | -1,0334 | 4,40E-03 |
| Card11 | -2,6983 | 4,48E-03 |
| Tbx15 | -1,7131 | 4,51E-03 |
| Lhpp | 1,1784 | 4,54E-03 |
| Plek | -0,9727 | 4,55E-03 |
| Cd300lf | -0,9959 | 4,55E-03 |
| Col4a5 | 0,4968 | 4,60E-03 |
| Mcoln2 | -2,1603 | 4,60E-03 |
| Hvcn1 | -1,0711 | 4,60E-03 |
| Trim65 | -0,7060 | 4,60E-03 |
| Gimap4 | -2,5545 | 4,65E-03 |
| Sema4c | -0,5765 | 4,78E-03 |
| Fads6 | 1,1530 | 4,84E-03 |
| Lama3 | -1,6329 | 4,85E-03 |
| Anpep | 0,5070 | 4,91E-03 |
| Itgb7 | -2,9924 | 4,94E-03 |
| Tnfsf11 | -3,8999 | 4,94E-03 |
| Fam111a | -0,8388 | 4,96E-03 |
| Gm8378 | 3,5377 | 4,98E-03 |
| Slitrk4 | -5,0577 | 4,99E-03 |
| Msi1 | 1,6892 | 4,99E-03 |
| Clca1 | 0,7282 | 4,99E-03 |
| Mgll | 0,4698 | 5,00E-03 |
| Cerk | -1,0322 | 5,00E-03 |
| Klk8 | -2,8379 | 5,00E-03 |
| Stard13 | 0,5562 | 5,01E-03 |
| Gem | -1,6844 | 5,03E-03 |
| Ddr2 | -0,8578 | 5,10E-03 |
| Spic | -2,9185 | 5,12E-03 |
| Trbv2 | -6,9425 | 5,25E-03 |
| Trpm2 | -1,3075 | 5,32E-03 |
| Gm8995 | -2,4826 | 5,41E-03 |
| Pim1 | -0,7379 | 5,41E-03 |
| Isyna1 | -1,0373 | 5,42E-03 |
| Gsdmd | -1,3600 | 5,42E-03 |
| C1s1 | -1,1596 | 5,42E-03 |
| Creb3l1 | -0,6115 | 5,42E-03 |
| Slc7a6 | -1,4455 | 5,42E-03 |
| Igkv16-104 | -4,8303 | 5,49E-03 |
| RP24-119I10,2 | -6,9046 | 5,49E-03 |
| Rnase6 | -1,8562 | 5,51E-03 |
| Pdgfc | 0,5646 | 5,54E-03 |
| Trp53inp1 | -0,6733 | 5,56E-03 |
| Fgfr2 | -0,9160 | 5,61E-03 |
| Ighv8-11 | -4,2751 | 5,61E-03 |
| Fndc5 | -2,4035 | 5,66E-03 |
| Nrp1 | -0,6009 | 5,66E-03 |
| Gpr68 | -1,9431 | 5,67E-03 |
| Trerf1 | -1,5106 | 5,68E-03 |
| Gdf11 | -1,4295 | 5,88E-03 |
| Antxr2 | -0,9782 | 5,89E-03 |
| D930015E06Rik | -0,5994 | 5,89E-03 |
| RP24-74O18,2 | -0,8008 | 5,90E-03 |
| Dcp1b | -1,9055 | 6,07E-03 |
| Ifi203 | -1,2678 | 6,09E-03 |
| Hk2 | 0,5729 | 6,10E-03 |
| Tmem156 | -2,9121 | 6,13E-03 |
| Prom2 | 0,6127 | 6,24E-03 |
| Prex2 | -0,5470 | 6,30E-03 |
| PCDHGB6 | 1,0265 | 6,36E-03 |
| RP23-354D10,2 | -2,7758 | 6,37E-03 |
| 4930523C07Rik | -0,8404 | 6,37E-03 |
| Tgfbr3 | 0,4645 | 6,40E-03 |
| RP24-92A2,1 | 0,6660 | 6,40E-03 |
| Ms4a6c | -1,2153 | 6,50E-03 |
| Klhl14 | -6,8339 | 6,52E-03 |
| S100a4 | -2,2724 | 6,72E-03 |
| Csrp1 | -0,5559 | 6,72E-03 |
| Hdac7 | -0,6742 | 6,72E-03 |
| Dok3 | -1,1591 | 6,81E-03 |
| Cspg5 | 0,7036 | 6,88E-03 |
| Gm26877 | -3,4040 | 6,95E-03 |
| Tm6sf1 | -1,3895 | 7,29E-03 |
| Npnt | 0,5214 | 7,29E-03 |
| Inpp4b | -0,9113 | 7,31E-03 |
| Nefh | -2,8330 | 7,43E-03 |
| Mir142hg | -2,9454 | 7,47E-03 |
| Lpar1 | -1,2571 | 7,51E-03 |
| Blnk | -0,9073 | 7,54E-03 |
| Loxl1 | -0,7484 | 7,58E-03 |
| Igkv12-44 | -6,1039 | 7,61E-03 |
| Ugcg | -0,8628 | 7,64E-03 |
| Tbc1d1 | -0,5089 | 7,64E-03 |
| Armcx4 | -1,1812 | 7,64E-03 |
| Guf1 | 0,5180 | 7,71E-03 |
| Cd4 | -2,4674 | 7,71E-03 |
| Rhof | -1,3399 | 7,72E-03 |
| Chmp5 | -0,6256 | 7,75E-03 |
| Csn3 | 0,6269 | 7,76E-03 |
| Plekhb1 | 0,4972 | 7,83E-03 |
| Cybb | -0,6311 | 7,85E-03 |
| Zfp516 | 0,4584 | 7,88E-03 |
| Lrig3 | -0,9742 | 7,98E-03 |
| Slc25a40 | -0,9815 | 8,02E-03 |
| Arrb2 | -0,7965 | 8,02E-03 |
| Gm26797 | 1,7425 | 8,02E-03 |
| Cers4 | -1,6343 | 8,05E-03 |
| Wnt7a | 2,6507 | 8,06E-03 |
| A130023I24Rik | 1,6531 | 8,19E-03 |
| Them6 | -2,3556 | 8,19E-03 |
| Fam198b | -1,0646 | 8,19E-03 |
| Eml4 | -0,5473 | 8,22E-03 |
| Tead4 | -1,0035 | 8,24E-03 |
| AW112010 | -1,4659 | 8,25E-03 |
| Plbd1 | -1,0803 | 8,30E-03 |
| Pdk1 | 0,5532 | 8,41E-03 |
| Gm7967 | -0,5668 | 8,46E-03 |
| Crim1 | -0,5874 | 8,48E-03 |
| Axin2 | -1,9908 | 8,53E-03 |
| Nfatc1 | -0,6738 | 8,74E-03 |
| Sparc | -0,5195 | 8,74E-03 |
| Mfng | -1,4737 | 8,74E-03 |
| Plxdc1 | -1,7847 | 8,80E-03 |
| Perp | 0,6233 | 8,80E-03 |
| Swap70 | -0,4691 | 8,80E-03 |
| Masp1 | 1,3425 | 8,80E-03 |
| Pknox2 | 1,3682 | 8,91E-03 |
| Lpar5 | -3,8442 | 8,92E-03 |
| Malt1 | -0,6226 | 8,94E-03 |
| Irgm2 | -0,8177 | 8,94E-03 |
| RP23-216O10,9 | -6,7255 | 8,97E-03 |
| Adamts14 | -2,5132 | 8,99E-03 |
| Gm10521 | -4,4620 | 9,03E-03 |
| Slc9a9 | -1,4107 | 9,04E-03 |
| Kcnk6 | -1,4648 | 9,04E-03 |
| Panx1 | -1,9594 | 9,06E-03 |
| Lrp5 | 0,4539 | 9,13E-03 |
| Coro2b | 1,0411 | 9,13E-03 |
| Rasgrp2 | -3,9199 | 9,33E-03 |
| Myc | 0,4656 | 9,36E-03 |
| Adcy7 | -0,5094 | 9,47E-03 |
| Dnajc27 | -1,0496 | 9,53E-03 |
| Zfp648 | 6,8326 | 9,59E-03 |
| Nfkbid | -1,4982 | 9,59E-03 |
| Dmd | 0,5949 | 9,70E-03 |
| Cdh11 | -0,7513 | 9,73E-03 |
| Zfp251 | -2,3316 | 9,78E-03 |
| Atp8b4 | -1,9849 | 9,79E-03 |
| Pcdhgb1 | 0,6976 | 1,00E-02 |
| Igkj1 | -3,7649 | 1,00E-02 |
| Ighv1-64 | -4,1043 | 1,00E-02 |
| Smap2 | -0,6698 | 1,01E-02 |
| Ptpn14 | -0,5373 | 1,02E-02 |
| H2-DMa | -1,1501 | 1,02E-02 |
| Nkg7 | -2,7102 | 1,02E-02 |
| Ncf4 | -1,0020 | 1,03E-02 |
| Ak4 | 1,3125 | 1,04E-02 |
| Trbv1 | -3,8574 | 1,04E-02 |
| Slmap | 0,4054 | 1,04E-02 |
| H2-Q2 | -0,5955 | 1,05E-02 |
| Nr1h3 | -1,0992 | 1,05E-02 |
| Sdr16c5 | 1,9416 | 1,05E-02 |
| Esrrg | 0,7642 | 1,06E-02 |
| Itgae | -1,3477 | 1,06E-02 |
| Fyn | -0,4946 | 1,06E-02 |
| Slc14a1 | -4,2795 | 1,08E-02 |
| Gimap6 | -2,7800 | 1,08E-02 |
| Lrrc15 | -0,6434 | 1,09E-02 |
| Lrrc18 | -3,0691 | 1,09E-02 |
| Col5a1 | -0,5510 | 1,11E-02 |
| Atp6v1a | 0,5125 | 1,11E-02 |
| Arc | -1,9494 | 1,11E-02 |
| Akr1cl | -6,6720 | 1,11E-02 |
| Atp2b4 | -1,1629 | 1,11E-02 |
| Dpp10 | -1,2990 | 1,12E-02 |
| Tfap2c | 0,4989 | 1,13E-02 |
| H2-T23 | -0,7166 | 1,13E-02 |
| D1Ertd622e | -0,7500 | 1,13E-02 |
| Fam69a | -1,2891 | 1,14E-02 |
| Cdyl2 | -1,5923 | 1,14E-02 |
| Sox21 | 0,7755 | 1,15E-02 |
| Igkv8-21 | -5,9622 | 1,16E-02 |
| Rasl11b | -1,3094 | 1,17E-02 |
| Cd7 | -2,9765 | 1,17E-02 |
| E2f2 | -1,5152 | 1,17E-02 |
| Irf1 | -0,6368 | 1,17E-02 |
| Rap1gap2 | 0,6109 | 1,17E-02 |
| Mbnl1 | -0,5188 | 1,18E-02 |
| Fam46a | -0,7265 | 1,18E-02 |
| Hr | 0,4950 | 1,19E-02 |
| Gypc | -2,5493 | 1,19E-02 |
| Zdhhc15 | -3,0726 | 1,20E-02 |
| Egfl7 | -0,8452 | 1,21E-02 |
| Atp13a4 | 0,6370 | 1,21E-02 |
| C1ra | -0,8366 | 1,21E-02 |
| Grk6 | -0,5873 | 1,21E-02 |
| Ntrk1 | -6,7425 | 1,22E-02 |
| Mgst2 | 1,0763 | 1,22E-02 |
| Il7r | -3,0668 | 1,22E-02 |
| Prss35 | 1,9618 | 1,23E-02 |
| Dnm1 | -0,9826 | 1,24E-02 |
| Bmf | -0,6173 | 1,24E-02 |
| Fcrl5 | -5,9371 | 1,24E-02 |
| 6030445D17Rik | 6,7707 | 1,25E-02 |
| Tbcel | -1,6442 | 1,25E-02 |
| Ampd3 | -1,4893 | 1,25E-02 |
| Igkv4-55 | -6,6904 | 1,25E-02 |
| Cgnl1 | 0,4045 | 1,26E-02 |
| Ccdc141 | -0,6716 | 1,26E-02 |
| Ednra | -1,2133 | 1,26E-02 |
| Itgb3 | 0,5067 | 1,26E-02 |
| Gm16152 | -4,8492 | 1,26E-02 |
| Socs2 | 0,4508 | 1,28E-02 |
| Lbp | 0,5289 | 1,28E-02 |
| Prdm5 | -2,5840 | 1,28E-02 |
| Irs1 | 0,3849 | 1,28E-02 |
| Mpzl3 | 0,5430 | 1,29E-02 |
| Angptl2 | -0,6647 | 1,30E-02 |
| Nupr1 | 0,5174 | 1,30E-02 |
| Ptpn6 | -0,5740 | 1,31E-02 |
| Ctla4 | -2,4956 | 1,33E-02 |
| Pcdh17 | -2,3209 | 1,33E-02 |
| Atxn1 | 0,4078 | 1,33E-02 |
| Itga11 | -1,1313 | 1,33E-02 |
| Pitpnm2 | -0,7148 | 1,33E-02 |
| Gm28693 | -3,3198 | 1,33E-02 |
| H2-Ea-ps | -1,7189 | 1,34E-02 |
| Cacna1c | -1,0562 | 1,36E-02 |
| Arl5c | -1,8435 | 1,36E-02 |
| Tle4 | -0,5943 | 1,36E-02 |
| Gria1 | -3,2333 | 1,36E-02 |
| Rsad2 | 1,3375 | 1,37E-02 |
| Pcdhga4 | 1,0237 | 1,38E-02 |
| Trbv24 | -6,7122 | 1,39E-02 |
| Lurap1l | 0,6695 | 1,42E-02 |
| Cyth4 | -0,7875 | 1,43E-02 |
| Rab6b | 1,6685 | 1,44E-02 |
| Igkv12-41 | -5,9070 | 1,44E-02 |
| Thy1 | -1,1619 | 1,46E-02 |
| Gm8221 | -6,6033 | 1,47E-02 |
| Cers6 | -0,8644 | 1,48E-02 |
| Sec24d | -0,6431 | 1,49E-02 |
| Rbms3 | -1,0094 | 1,49E-02 |
| Cd36 | 1,2803 | 1,50E-02 |
| Glcci1 | -2,6464 | 1,52E-02 |
| Pde2a | -0,6938 | 1,52E-02 |
| PCDHGA9 | 0,7653 | 1,53E-02 |
| Prom1 | -0,8765 | 1,53E-02 |
| 4930439D14Rik | -6,6309 | 1,56E-02 |
| Cd86 | -1,1490 | 1,57E-02 |
| Rragb | -4,8020 | 1,58E-02 |
| Fam180a | -6,5401 | 1,58E-02 |
| 5031434C07Rik | 0,8371 | 1,58E-02 |
| Gstm2 | 0,8580 | 1,59E-02 |
| Ffar1 | -6,6355 | 1,62E-02 |
| Hif1a | 0,4150 | 1,63E-02 |
| Nrg2 | 0,5919 | 1,63E-02 |
| Gm1966 | -2,2340 | 1,63E-02 |
| Gm9208 | -6,6813 | 1,63E-02 |
| Serpina3f | -2,0952 | 1,64E-02 |
| Tsc22d3 | -0,5670 | 1,68E-02 |
| Stag3 | -3,9154 | 1,68E-02 |
| Ighv1-66 | -3,4181 | 1,69E-02 |
| Ighv1-34 | -4,0340 | 1,69E-02 |
| Lap3 | -0,4620 | 1,70E-02 |
| Sfrp4 | 3,6294 | 1,73E-02 |
| Gstm4 | 0,8638 | 1,74E-02 |
| Iglv1 | -6,5200 | 1,74E-02 |
| Ighv1-7 | -6,5250 | 1,74E-02 |
| Tet2 | 0,4342 | 1,74E-02 |
| Trp53i11 | -0,8310 | 1,76E-02 |
| Mamdc2 | -2,1043 | 1,77E-02 |
| Nup210 | -0,6992 | 1,77E-02 |
| Hhipl2 | 0,5111 | 1,77E-02 |
| Gm14133 | 1,1563 | 1,77E-02 |
| Baiap2l1 | 0,5085 | 1,78E-02 |
| Rcn3 | -0,9002 | 1,78E-02 |
| Map4k2 | -0,7112 | 1,78E-02 |
| Ssc4d | 1,1189 | 1,79E-02 |
| Fcrla | -6,1378 | 1,82E-02 |
| 4930503L19Rik | 0,6081 | 1,83E-02 |
| Vtcn1 | 0,4910 | 1,83E-02 |
| Npr3 | 1,4256 | 1,83E-02 |
| Ptger4 | -0,9331 | 1,83E-02 |
| Gm13394 | 0,4762 | 1,84E-02 |
| 5330416C01Rik | -2,9580 | 1,84E-02 |
| Oat | 0,4382 | 1,86E-02 |
| Neu2 | 1,4277 | 1,86E-02 |
| Lta | -3,2794 | 1,88E-02 |
| Slc25a34 | 0,7792 | 1,88E-02 |
| 1600014C10Rik | -0,7385 | 1,88E-02 |
| Mal | -1,8983 | 1,88E-02 |
| Nfkbie | -0,7814 | 1,89E-02 |
| Hist1h1a | -0,8007 | 1,91E-02 |
| Sfrp1 | 0,4599 | 1,92E-02 |
| St3gal6 | -1,5684 | 1,92E-02 |
| Abcg3 | -1,5256 | 1,94E-02 |
| Chst4 | -6,0399 | 1,96E-02 |
| Adam12 | 0,5753 | 1,96E-02 |
| Gm4134 | -2,9467 | 1,96E-02 |
| Olfr164 | -3,9758 | 1,96E-02 |
| Ddit4l | 1,8013 | 1,96E-02 |
| Kcnq3 | -2,6073 | 1,96E-02 |
| Ucp2 | -0,3920 | 1,97E-02 |
| Cpeb4 | 0,4225 | 1,97E-02 |
| Dscaml1 | -6,4997 | 1,99E-02 |
| Spi1 | -0,9615 | 2,00E-02 |
| Pitpnc1 | 0,4193 | 2,00E-02 |
| C1qc | -0,5289 | 2,00E-02 |
| Cadm1 | -0,4809 | 2,00E-02 |
| Fam115c | -0,6506 | 2,01E-02 |
| Zbtb20 | 0,3854 | 2,04E-02 |
| Ighv2-2 | -4,7703 | 2,05E-02 |
| Ppp1r13l | 0,4667 | 2,05E-02 |
| BC051142 | -6,6544 | 2,05E-02 |
| mmu-mir-5103 | -2,2285 | 2,07E-02 |
| Fbn1 | -0,5151 | 2,07E-02 |
| Siglech | -1,5601 | 2,07E-02 |
| Ighj2 | -3,0720 | 2,07E-02 |
| Acvrl1 | -0,9589 | 2,08E-02 |
| Peli2 | -0,9743 | 2,08E-02 |
| Kif26b | -0,8424 | 2,08E-02 |
| Snx22 | -0,7414 | 2,08E-02 |
| Macc1 | 0,5081 | 2,08E-02 |
| Vmn2r84 | -6,4484 | 2,10E-02 |
| Afap1l2 | 0,4367 | 2,11E-02 |
| Itga4 | -2,0077 | 2,11E-02 |
| Cd207 | -3,0170 | 2,11E-02 |
| Ugp2 | 0,4498 | 2,14E-02 |
| Gm25395 | -0,5379 | 2,14E-02 |
| Itga9 | -0,7380 | 2,15E-02 |
| Fbn2 | -0,6926 | 2,15E-02 |
| C1qa | -0,5523 | 2,15E-02 |
| Dennd4b | -0,9300 | 2,15E-02 |
| Btnl2 | -4,4348 | 2,16E-02 |
| F730043M19Rik | -2,1428 | 2,17E-02 |
| Bahcc1 | -0,4695 | 2,18E-02 |
| Ly75 | -2,1828 | 2,18E-02 |
| Mb | 0,9657 | 2,20E-02 |
| Gm20511 | -6,4585 | 2,21E-02 |
| Hist1h2bn | -0,6500 | 2,22E-02 |
| Bach2 | -0,7982 | 2,23E-02 |
| Rerg | -1,7487 | 2,25E-02 |
| Cyp26b1 | -1,2028 | 2,26E-02 |
| Olfr98 | -6,5251 | 2,26E-02 |
| Traj15 | -6,5251 | 2,26E-02 |
| Gm16310 | 0,6494 | 2,26E-02 |
| Tab3 | 0,5608 | 2,26E-02 |
| Ighv1-80 | -3,1526 | 2,30E-02 |
| Mettl9 | 0,4679 | 2,31E-02 |
| Abcc4 | -1,2021 | 2,33E-02 |
| Kitl | 0,4955 | 2,35E-02 |
| Cep112 | -1,7574 | 2,36E-02 |
| Tmem47 | -0,5878 | 2,36E-02 |
| Aspn | -0,9095 | 2,38E-02 |
| Bgn | -0,6142 | 2,39E-02 |
| Dbi | 0,3887 | 2,41E-02 |
| Mir99ahg | 0,5586 | 2,41E-02 |
| Mbd4 | -0,7257 | 2,41E-02 |
| Jade2 | -0,6525 | 2,41E-02 |
| Kdm3a | 0,4098 | 2,43E-02 |
| Gatsl2 | -1,0624 | 2,47E-02 |
| Kynu | -1,9545 | 2,47E-02 |
| Dab1 | -6,4161 | 2,49E-02 |
| Slc35f2 | -6,4214 | 2,52E-02 |
| Rps6ka5 | -0,8178 | 2,53E-02 |
| Igkv10-94 | -5,8906 | 2,55E-02 |
| Ccr2 | -0,7350 | 2,56E-02 |
| Pdlim2 | -1,4626 | 2,56E-02 |
| Wfdc2 | -1,7073 | 2,56E-02 |
| Gm4117 | 0,5211 | 2,56E-02 |
| Bai2 | -3,7315 | 2,56E-02 |
| Zfr2 | 0,6209 | 2,56E-02 |
| Entpd1 | -0,6603 | 2,56E-02 |
| Snhg18 | -1,1342 | 2,57E-02 |
| Vtn | -2,4826 | 2,57E-02 |
| Slc28a2 | -2,4295 | 2,57E-02 |
| Cblb | -0,7205 | 2,57E-02 |
| Man2a2 | -0,7456 | 2,61E-02 |
| Chka | 0,3804 | 2,62E-02 |
| Pik3ap1 | -0,5281 | 2,62E-02 |
| 6030407O03Rik | 1,7963 | 2,63E-02 |
| Nrbp2 | 0,3999 | 2,63E-02 |
| Gm3719 | 2,5030 | 2,64E-02 |
| Dnajc22 | 2,0584 | 2,66E-02 |
| Gas7 | -0,8124 | 2,68E-02 |
| Slc2a3 | -1,8553 | 2,68E-02 |
| Ldhd | -0,5851 | 2,68E-02 |
| Tsc22d2 | 0,4139 | 2,71E-02 |
| Mcm3 | -0,4926 | 2,71E-02 |
| Ifit2 | -0,8260 | 2,72E-02 |
| Eif4e3 | -1,2787 | 2,73E-02 |
| Relb | -0,6621 | 2,73E-02 |
| Lonrf3 | 0,7168 | 2,74E-02 |
| Zeb2 | -0,5623 | 2,74E-02 |
| Tesc | 0,6536 | 2,74E-02 |
| Xrcc5 | 0,5150 | 2,75E-02 |
| Wtip | 0,7300 | 2,78E-02 |
| Prol1 | 1,9237 | 2,79E-02 |
| Marcks | -0,4027 | 2,79E-02 |
| Ppp3ca | 0,3847 | 2,80E-02 |
| Ctso | -0,6570 | 2,81E-02 |
| 2900052N01Rik | -5,6238 | 2,81E-02 |
| Pdrg1 | 0,4777 | 2,81E-02 |
| Adrb1 | 0,5069 | 2,81E-02 |
| 9130019P16Rik | -0,8407 | 2,82E-02 |
| Cbfa2t3 | -1,0198 | 2,83E-02 |
| Ctnnd2 | -1,5316 | 2,84E-02 |
| Aox1 | 0,6017 | 2,84E-02 |
| Gm16151 | -4,6429 | 2,87E-02 |
| Oasl2 | 0,6446 | 2,93E-02 |
| Cd55 | -0,5332 | 2,95E-02 |
| Fzd9 | -4,1491 | 2,97E-02 |
| 3425401B19Rik | -6,5002 | 2,97E-02 |
| Gm7334 | -1,7528 | 2,98E-02 |
| BC017643 | -0,5541 | 2,98E-02 |
| Gm11737 | -1,0265 | 2,98E-02 |
| Lrrc75b | -0,5848 | 2,98E-02 |
| Lrp2 | -5,7649 | 2,99E-02 |
| Slc41a2 | 0,6451 | 3,01E-02 |
| Sipa1l2 | -0,5039 | 3,04E-02 |
| Bckdhb | -1,6759 | 3,04E-02 |
| Gpr35 | -1,1924 | 3,05E-02 |
| Sidt1 | -2,3974 | 3,06E-02 |
| Ank2 | -1,0828 | 3,07E-02 |
| Gpr153 | -0,8875 | 3,09E-02 |
| F13a1 | 1,0665 | 3,10E-02 |
| Gabbr2 | -4,6155 | 3,14E-02 |
| Kcng1 | -6,3159 | 3,14E-02 |
| Fcho1 | -1,2060 | 3,15E-02 |
| Apol7c | -6,3101 | 3,15E-02 |
| Ppp4r1l-ps | 0,4570 | 3,17E-02 |
| Gpr83 | -4,5482 | 3,18E-02 |
| Rassf4 | -0,7603 | 3,18E-02 |
| Taf13 | 0,6300 | 3,18E-02 |
| RP23-328G14,2 | -2,5880 | 3,18E-02 |
| Casc4 | -1,6805 | 3,19E-02 |
| Gm13502 | -0,9904 | 3,20E-02 |
| Nod2 | -1,5127 | 3,21E-02 |
| Igkv4-72 | -6,5055 | 3,24E-02 |
| Foxc2 | -2,8546 | 3,27E-02 |
| Slc43a2 | -0,5370 | 3,30E-02 |
| Slc25a36 | 0,4262 | 3,30E-02 |
| BC006965 | 0,3573 | 3,31E-02 |
| Pdgfrb | -0,6410 | 3,32E-02 |
| Psd4 | -0,6220 | 3,33E-02 |
| Dkk2 | -2,0026 | 3,33E-02 |
| Olfml3 | -0,5767 | 3,35E-02 |
| Gm14010 | 1,2352 | 3,35E-02 |
| Fcamr | -2,5893 | 3,36E-02 |
| Ms4a6b | -2,2219 | 3,38E-02 |
| Ano1 | 0,3484 | 3,39E-02 |
| Cttnbp2nl | 0,4450 | 3,39E-02 |
| Celsr1 | -1,4752 | 3,42E-02 |
| Rasgrp1 | -2,0006 | 3,43E-02 |
| Sspo | -4,1922 | 3,43E-02 |
| Napsa | -1,5852 | 3,44E-02 |
| Aif1 | -0,8947 | 3,44E-02 |
| Grm6 | -6,3557 | 3,45E-02 |
| Wipf3 | 0,5417 | 3,48E-02 |
| Enox1 | -2,2816 | 3,48E-02 |
| Tmem98 | -0,6773 | 3,50E-02 |
| Gm21718 | 2,1573 | 3,50E-02 |
| Fgf13 | -2,9408 | 3,53E-02 |
| Snx7 | -0,8144 | 3,54E-02 |
| Ppic | -0,6907 | 3,55E-02 |
| Gm11734 | 1,4530 | 3,55E-02 |
| 4833407H14Rik | -2,2327 | 3,56E-02 |
| Me2 | -0,7604 | 3,56E-02 |
| Cd48 | -1,3061 | 3,58E-02 |
| Trat1 | -4,9064 | 3,58E-02 |
| Apln | 1,3311 | 3,58E-02 |
| Slc15a5 | 1,5430 | 3,58E-02 |
| H2-DMb1 | -0,7918 | 3,58E-02 |
| Traj24 | -6,2925 | 3,60E-02 |
| St3gal1 | -1,0539 | 3,65E-02 |
| Cdh2 | -1,2647 | 3,65E-02 |
| A930006K02Rik | -3,0238 | 3,65E-02 |
| Arhgdib | -1,8584 | 3,66E-02 |
| Prrg1 | -2,0578 | 3,66E-02 |
| Ncor2 | 0,3409 | 3,66E-02 |
| Mxi1 | 0,4119 | 3,68E-02 |
| Ccdc30 | 3,6916 | 3,68E-02 |
| RP23-328G14,1 | -2,4936 | 3,68E-02 |
| Fmnl3 | -0,4680 | 3,70E-02 |
| Fam105a | -0,8539 | 3,70E-02 |
| 3110021N24Rik | 4,1104 | 3,72E-02 |
| Cd274 | -1,4132 | 3,72E-02 |
| Endou | -5,5874 | 3,74E-02 |
| Ighv1-4 | -6,3670 | 3,74E-02 |
| Fosl2 | -0,4140 | 3,75E-02 |
| Rgs10 | -0,7747 | 3,75E-02 |
| Ighv1-53 | -4,5034 | 3,75E-02 |
| Ctsl | 0,3944 | 3,75E-02 |
| Scarna6 | -0,5251 | 3,77E-02 |
| RP23-349H13,2 | -0,7924 | 3,77E-02 |
| Ighv1-69 | -3,4105 | 3,77E-02 |
| Zak | 0,5070 | 3,78E-02 |
| Atcay | -4,5541 | 3,78E-02 |
| C530043A13Rik | 0,5924 | 3,81E-02 |
| Scd3 | 1,1026 | 3,83E-02 |
| Fam134b | 0,4016 | 3,86E-02 |
| Gpsm3 | -1,1667 | 3,87E-02 |
| Cxcl16 | -0,5992 | 3,91E-02 |
| Adrbk1 | -0,4216 | 3,92E-02 |
| Aatk | -0,4783 | 3,93E-02 |
| Limd2 | -0,5042 | 3,93E-02 |
| Gm16432 | 0,5535 | 3,98E-02 |
| Cenph | -0,8393 | 3,98E-02 |
| Padi2 | -0,6999 | 3,98E-02 |
| Appl2 | 0,4146 | 4,00E-02 |
| Rundc3b | -4,7238 | 4,00E-02 |
| Serpina1b | -2,0009 | 4,00E-02 |
| Gm18828 | -6,2387 | 4,00E-02 |
| Btnl7-ps | -3,2141 | 4,00E-02 |
| Rgs19 | -0,7139 | 4,01E-02 |
| Vpreb3 | -6,2270 | 4,02E-02 |
| Fndc3b | 0,4393 | 4,05E-02 |
| Spred3 | 0,6235 | 4,05E-02 |
| Insrr | -5,5279 | 4,07E-02 |
| Il17rd | 0,4551 | 4,08E-02 |
| Slc5a5 | 3,2066 | 4,09E-02 |
| Nuak1 | -0,8575 | 4,09E-02 |
| Traj7 | -6,2082 | 4,09E-02 |
| Gm20645 | -1,0066 | 4,09E-02 |
| Gm26753 | 2,3493 | 4,12E-02 |
| Amdhd2 | -1,5425 | 4,13E-02 |
| Art4 | -6,3217 | 4,15E-02 |
| Zfp618 | 0,8338 | 4,15E-02 |
| Itga6 | 0,3954 | 4,16E-02 |
| Trbv19 | -4,5950 | 4,16E-02 |
| Gm13185 | -3,5676 | 4,16E-02 |
| Xlr4a | -5,4669 | 4,16E-02 |
| Fam49b | -0,5031 | 4,16E-02 |
| Dyrk2 | -0,5802 | 4,16E-02 |
| Gm19585 | -5,4744 | 4,16E-02 |
| Csf1r | -0,4885 | 4,17E-02 |
| Tgfbr1 | -0,4323 | 4,17E-02 |
| Serpina10 | -5,4869 | 4,18E-02 |
| Rcn1 | -0,5505 | 4,20E-02 |
| Anxa9 | 0,5044 | 4,20E-02 |
| Zfp938 | 0,6705 | 4,21E-02 |
| Gm12250 | -1,2154 | 4,22E-02 |
| Tmem82 | 1,0331 | 4,24E-02 |
| Zfp606 | 0,5130 | 4,25E-02 |
| Micu3 | 0,6345 | 4,25E-02 |
| RP23-1B19,2 | 2,3699 | 4,25E-02 |
| Ncam1 | -0,8451 | 4,25E-02 |
| Hist1h2af | -0,4665 | 4,25E-02 |
| Smoc2 | -0,8457 | 4,26E-02 |
| Tom1l1 | 0,4113 | 4,26E-02 |
| Apol9b | 0,9996 | 4,27E-02 |
| Gm4951 | -1,1127 | 4,27E-02 |
| Prkch | -0,5289 | 4,28E-02 |
| Sntb2 | 0,4136 | 4,30E-02 |
| Chic1 | -1,4966 | 4,30E-02 |
| Pcdh7 | -0,6880 | 4,32E-02 |
| 4931406P16Rik | 0,3951 | 4,33E-02 |
| C920025E04Rik | -0,6948 | 4,34E-02 |
| Sirpa | -0,4652 | 4,35E-02 |
| Golm1 | -0,7267 | 4,35E-02 |
| Ctsw | -1,3437 | 4,35E-02 |
| Ltbp4 | -0,8133 | 4,35E-02 |
| Scn2b | -2,4214 | 4,35E-02 |
| Trib3 | 0,5068 | 4,36E-02 |
| Bicc1 | -0,7485 | 4,39E-02 |
| Mif | 0,4763 | 4,40E-02 |
| Cyp27a1 | -1,4242 | 4,40E-02 |
| Prkd2 | -0,6589 | 4,42E-02 |
| Zdhhc18 | -0,5781 | 4,44E-02 |
| Col7a1 | -0,9539 | 4,44E-02 |
| Gbp7 | -0,6139 | 4,45E-02 |
| Pdcd1lg2 | -6,1829 | 4,46E-02 |
| Galnt7 | -0,8221 | 4,46E-02 |
| Ighv3-6 | -6,1766 | 4,47E-02 |
| Ppp2r2b | 0,5334 | 4,50E-02 |
| Vldlr | 0,3801 | 4,50E-02 |
| Ptprt | 0,8920 | 4,50E-02 |
| Tnfaip8l2 | -1,1947 | 4,50E-02 |
| Errfi1 | 0,4600 | 4,51E-02 |
| Obsl1 | 0,6186 | 4,52E-02 |
| Slc28a3 | 1,2111 | 4,60E-02 |
| Decr1 | -1,4280 | 4,62E-02 |
| Gstt3 | 0,5126 | 4,64E-02 |
| Epb4,1l3 | -1,2006 | 4,65E-02 |
| Pde4a | -1,3506 | 4,70E-02 |
| Ppm1j | -1,7588 | 4,71E-02 |
| Agrn | 0,3525 | 4,71E-02 |
| Slc22a21 | -2,6474 | 4,71E-02 |
| Cbx5 | 0,4169 | 4,71E-02 |
| Clstn2 | -1,7311 | 4,71E-02 |
| Emb | 0,5634 | 4,72E-02 |
| 2610203C20Rik | 0,4312 | 4,72E-02 |
| Rab37 | -1,4562 | 4,74E-02 |
| Col6a4 | -3,0827 | 4,75E-02 |
| Mfsd4 | -0,4470 | 4,75E-02 |
| Ehd4 | 0,3966 | 4,76E-02 |
| Pcyt2 | -0,5679 | 4,78E-02 |
| Pik3cd | -1,6586 | 4,83E-02 |
| 5031439G07Rik | 0,3663 | 4,84E-02 |
| Ormdl3 | 0,5501 | 4,84E-02 |
| Gm26742 | 0,8338 | 4,84E-02 |
| Ssx2ip | 0,3974 | 4,84E-02 |
| Pdlim4 | -1,4909 | 4,85E-02 |
| Tchh | -2,2895 | 4,85E-02 |
| Mex3b | -1,5166 | 4,87E-02 |
| Inhbb | 0,3807 | 4,89E-02 |
| G6pdx | 0,4420 | 4,89E-02 |
| Tnip1 | -0,6030 | 4,91E-02 |
| Amd1 | 0,5167 | 4,93E-02 |
| Mycbpap | -2,0931 | 4,94E-02 |
| Fgl2 | -0,8603 | 4,95E-02 |
| Myo7a | 0,4621 | 4,95E-02 |
| Igkv4-57 | -3,4262 | 4,95E-02 |
| Myh9 | -0,3314 | 4,99E-02 |
| Hpse | -1,3858 | 4,99E-02 |
| Ace | -0,6326 | 5,06E-02 |
| Ncf1 | -0,8572 | 5,06E-02 |
| Chmp4c | 0,3964 | 5,06E-02 |
| Traj18 | -4,0535 | 5,07E-02 |
| RP23-322P13,2 | -6,1444 | 5,08E-02 |
| Clmp | -1,2027 | 5,13E-02 |
| Map3k12 | 0,5604 | 5,14E-02 |
| Dlx3 | -3,4209 | 5,14E-02 |
| Zfp612 | 0,5147 | 5,17E-02 |
| Igkv4-53 | -6,2871 | 5,17E-02 |
| Caprin2 | 0,5806 | 5,19E-02 |
| Spata31d1b | -6,1118 | 5,21E-02 |
| S1pr3 | -1,2986 | 5,24E-02 |
| Fam13a | -0,9115 | 5,24E-02 |
| RP24-540L1,2 | -1,2823 | 5,24E-02 |
| Kdm7a | 0,3666 | 5,25E-02 |
| Rasgrf2 | -2,0777 | 5,26E-02 |
| Ift57 | 0,6293 | 5,26E-02 |
| Prf1 | -2,7131 | 5,26E-02 |
| Naaa | -0,7621 | 5,27E-02 |
| Plcxd2 | -1,4696 | 5,27E-02 |
| Galm | -1,1562 | 5,29E-02 |
| Gnpda2 | 0,5876 | 5,29E-02 |
| Epdr1 | -1,3033 | 5,29E-02 |
| 4930432E11Rik | 2,2449 | 5,29E-02 |
| Ighj1 | -3,6212 | 5,29E-02 |
| Sorbs3 | 0,4306 | 5,33E-02 |
| Gpr183 | -0,8856 | 5,37E-02 |
| Gm27240 | -1,2677 | 5,37E-02 |
| Igkv2-137 | -5,5911 | 5,41E-02 |
| Ighv1-81 | -5,4174 | 5,41E-02 |
| Igfbp5 | 0,3414 | 5,45E-02 |
| Gm15472 | -3,9953 | 5,45E-02 |
| Mast1 | -3,9697 | 5,46E-02 |
| A630033H20Rik | -3,6585 | 5,47E-02 |
| Apol9a | 0,7179 | 5,50E-02 |
| Fermt2 | 0,3639 | 5,51E-02 |
| Cd6 | -4,2901 | 5,52E-02 |
| Tsc22d1 | -0,4461 | 5,53E-02 |
| Gys1 | 0,4861 | 5,56E-02 |
| Etnk1 | 0,4075 | 5,60E-02 |
| Csad | 0,4525 | 5,61E-02 |
| Traj12 | -4,0087 | 5,61E-02 |
| Klrk1 | -1,9350 | 5,62E-02 |
| Hey2 | -1,8063 | 5,66E-02 |
| Susd3 | -1,5251 | 5,66E-02 |
| Ccdc148 | 0,8669 | 5,66E-02 |
| Gm8787 | 4,4355 | 5,66E-02 |
| Shisa3 | -2,1221 | 5,69E-02 |
| Dok7 | 1,0471 | 5,70E-02 |
| Slfn8 | -0,6965 | 5,74E-02 |
| Aak1 | 0,3983 | 5,82E-02 |
| Tcn2 | 0,3860 | 5,84E-02 |
| Bglap3 | 0,9680 | 5,84E-02 |
| Gls | 0,3509 | 5,84E-02 |
| 4930402H24Rik | -0,6714 | 5,85E-02 |
| Rnf144b | 0,6318 | 5,88E-02 |
| Fgfr1 | 0,3720 | 5,91E-02 |
| Ighv1-82 | -3,2595 | 5,93E-02 |
| BC039966 | 2,0826 | 5,99E-02 |
| Adam19 | -0,6032 | 6,00E-02 |
| Rell1 | 0,4455 | 6,04E-02 |
| Gm4841 | -1,6887 | 6,08E-02 |
| Ldha | 0,3547 | 6,10E-02 |
| Jakmip1 | -4,5037 | 6,13E-02 |
| Nr1d2 | 0,4519 | 6,14E-02 |
| Slc35g1 | -1,0371 | 6,16E-02 |
| Egln1 | 0,4336 | 6,17E-02 |
| Rps6ka1 | -0,4113 | 6,20E-02 |
| Dnajb4 | 0,5105 | 6,20E-02 |
| Rpgr | -2,0205 | 6,20E-02 |
| Grhl1 | -0,4668 | 6,22E-02 |
| Mmp2 | -0,6176 | 6,22E-02 |
| Gcnt4 | -1,6361 | 6,22E-02 |
| Susd1 | -2,0657 | 6,23E-02 |
| Actr3 | -0,3523 | 6,24E-02 |
| Csk | -0,4660 | 6,27E-02 |
| Nr2c1 | 0,4175 | 6,28E-02 |
| Tnfsf10 | -0,5261 | 6,28E-02 |
| Igkv2-109 | -4,3707 | 6,28E-02 |
| H2-Eb1 | -1,4139 | 6,28E-02 |
| Phlpp1 | 0,3657 | 6,34E-02 |
| Nhs | 0,5445 | 6,35E-02 |
| Bhlhe41 | -0,4337 | 6,35E-02 |
| Mill2 | -0,6463 | 6,35E-02 |
| Igkj3 | -6,1896 | 6,38E-02 |
| Wnk2 | 0,3426 | 6,47E-02 |
| Igkv6-14 | -5,2665 | 6,48E-02 |
| Cacna1b | -3,6763 | 6,54E-02 |
| Reck | 0,6948 | 6,54E-02 |
| Epb4,1l1 | 0,3446 | 6,57E-02 |
| Lgals1 | -0,6605 | 6,58E-02 |
| Calcoco1 | 0,3861 | 6,59E-02 |
| Col11a2 | 0,3717 | 6,59E-02 |
| Themis2 | -0,8122 | 6,59E-02 |
| Gm10800 | 0,6952 | 6,59E-02 |
| Trbv29 | -5,3651 | 6,59E-02 |
| Mcoln3 | -6,0435 | 6,61E-02 |
| Klf6 | 0,3979 | 6,62E-02 |
| Ctnnal1 | 0,6127 | 6,67E-02 |
| Adamts6 | -0,8228 | 6,67E-02 |
| Igkj4 | -6,0504 | 6,67E-02 |
| Gm22206 | -6,0504 | 6,67E-02 |
| Dbp | 0,8391 | 6,68E-02 |
| Nudt16 | -1,7550 | 6,73E-02 |
| Arrb1 | 0,3370 | 6,78E-02 |
| Gm10093 | -0,4679 | 6,78E-02 |
| Jmy | 0,3571 | 6,79E-02 |
| Igkv9-124 | -3,6442 | 6,79E-02 |
| Dact1 | -1,4329 | 6,79E-02 |
| Cd8a | -2,6537 | 6,79E-02 |
| Tmcc2 | -1,6098 | 6,82E-02 |
| Olfr756-ps1 | -3,6177 | 6,82E-02 |
| Fam26f | -2,2834 | 6,82E-02 |
| Ggt5 | -1,3264 | 6,85E-02 |
| Fam129b | 0,3527 | 6,85E-02 |
| Cyp2d11 | 2,5075 | 6,89E-02 |
| Btbd11 | -1,7214 | 6,93E-02 |
| Frmd4a | -0,5415 | 6,93E-02 |
| Tns1 | -0,5137 | 6,93E-02 |
| Sri | -0,4297 | 6,97E-02 |
| H2-T22 | -0,7768 | 7,04E-02 |
| Hist1h1d | -0,3813 | 7,08E-02 |
| Lrpap1 | 0,3908 | 7,08E-02 |
| Aplp2 | 0,3727 | 7,08E-02 |
| Pcdhgb5 | 0,7067 | 7,09E-02 |
| St3gal2 | -0,7493 | 7,09E-02 |
| Acvr1b | 0,4775 | 7,11E-02 |
| Mpped2 | 0,7278 | 7,12E-02 |
| Mfsd7c | 0,5231 | 7,15E-02 |
| Khdrbs3 | -2,7363 | 7,16E-02 |
| Kifc3 | 0,3597 | 7,16E-02 |
| Igkv6-32 | -6,0711 | 7,16E-02 |
| Ighv1-84 | -6,1703 | 7,16E-02 |
| Gm29245 | 6,0909 | 7,16E-02 |
| Phyh | 0,6234 | 7,18E-02 |
| Ubd | -1,8931 | 7,18E-02 |
| Gm7173 | 1,9228 | 7,18E-02 |
| Gm16150 | -5,3362 | 7,18E-02 |
| Pianp | -0,9786 | 7,23E-02 |
| Txndc15 | 0,4654 | 7,25E-02 |
| Pdzd2 | 0,3394 | 7,25E-02 |
| Zfp110 | 0,4029 | 7,25E-02 |
| Pid1 | -0,7563 | 7,26E-02 |
| Fyb | -1,4142 | 7,27E-02 |
| Gm3828 | 2,4135 | 7,27E-02 |
| Slc1a5 | -0,7813 | 7,27E-02 |
| Casp8 | -0,4847 | 7,29E-02 |
| Rtn1 | 0,4658 | 7,34E-02 |
| Nr4a3 | -2,0358 | 7,35E-02 |
| Klhl29 | -2,8972 | 7,40E-02 |
| Mkl1 | -0,4231 | 7,40E-02 |
| Fam115a | 0,4243 | 7,40E-02 |
| Slain1 | -0,5222 | 7,41E-02 |
| Gabbr1 | -0,7140 | 7,41E-02 |
| Spryd3 | 0,4648 | 7,42E-02 |
| Fgd2 | -0,8193 | 7,49E-02 |
| Crip1 | -1,1977 | 7,51E-02 |
| Unc93b1 | -0,5178 | 7,53E-02 |
| Sema3d | 0,4254 | 7,53E-02 |
| Wif1 | -5,9938 | 7,54E-02 |
| Bpgm | -0,5823 | 7,54E-02 |
| Tmem173 | -0,4272 | 7,54E-02 |
| Kcna6 | -0,8962 | 7,57E-02 |
| Erbb2 | 0,3034 | 7,57E-02 |
| Pcdhb15 | -1,7244 | 7,60E-02 |
| BC021614 | -3,3461 | 7,61E-02 |
| Prodh | -1,0150 | 7,64E-02 |
| RP23-156A3,1 | 0,9912 | 7,64E-02 |
| Gm973 | -1,8548 | 7,66E-02 |
| Atp1a1 | 0,3986 | 7,67E-02 |
| Esyt1 | -0,4460 | 7,72E-02 |
| Pear1 | -1,0654 | 7,73E-02 |
| Syt8 | -1,6182 | 7,73E-02 |
| Uvrag | -0,4174 | 7,73E-02 |
| 9530077C05Rik | -2,4153 | 7,73E-02 |
| RP24-490B17,5 | -4,3277 | 7,73E-02 |
| Mybl1 | -0,8475 | 7,74E-02 |
| Pamr1 | -2,0151 | 7,74E-02 |
| Sorbs2os | 0,4591 | 7,74E-02 |
| Gm26580 | -6,0082 | 7,77E-02 |
| Gbp8 | -1,1165 | 7,80E-02 |
| Slc38a2 | 0,3475 | 7,82E-02 |
| Wscd2 | -4,4071 | 7,83E-02 |
| C130074G19Rik | -0,9398 | 7,85E-02 |
| RP24-227F14,5 | -2,2962 | 7,86E-02 |
| Stk10 | -0,4528 | 7,86E-02 |
| Slc11a2 | -0,6061 | 7,86E-02 |
| Smchd1 | -0,3608 | 7,86E-02 |
| Qprt | -3,9405 | 7,86E-02 |
| Klhdc7a | 0,3618 | 7,86E-02 |
| Ctsz | -0,6473 | 7,87E-02 |
| Clstn1 | 0,3180 | 7,88E-02 |
| RP23-148E7,2 | -5,9428 | 7,88E-02 |
| Etl4 | 0,3167 | 8,03E-02 |
| Hgf | -1,3774 | 8,05E-02 |
| Gm5637 | -0,6598 | 8,07E-02 |
| Tmx4 | 0,3747 | 8,08E-02 |
| Zc3h12a | -0,7009 | 8,08E-02 |
| Itih5l-ps | 0,9278 | 8,08E-02 |
| 5430416O09Rik | 0,4050 | 8,09E-02 |
| AF529169 | 2,0588 | 8,10E-02 |
| Gm16158 | -5,2675 | 8,11E-02 |
| RP23-435E15,3 | 0,5432 | 8,17E-02 |
| Trpm3 | 0,4441 | 8,19E-02 |
| PCDHGA12 | 0,6238 | 8,19E-02 |
| Acy1 | 0,6980 | 8,22E-02 |
| Scd2 | 0,3292 | 8,25E-02 |
| Col12a1 | -0,4237 | 8,28E-02 |
| Cpd | 0,3811 | 8,30E-02 |
| Tnfrsf12a | 0,6280 | 8,31E-02 |
| Rspo1 | -4,6333 | 8,39E-02 |
| RP24-222G3,2 | 0,7111 | 8,39E-02 |
| Rwdd2a | 1,1505 | 8,42E-02 |
| Slc16a5 | -2,8177 | 8,43E-02 |
| Pip4k2b | -0,4766 | 8,45E-02 |
| Igkv13-84 | -5,5156 | 8,50E-02 |
| Tnfrsf26 | -2,2364 | 8,52E-02 |
| Gm12058 | -5,9199 | 8,53E-02 |
| Copz1 | 0,4102 | 8,55E-02 |
| Cfap44 | 1,0902 | 8,56E-02 |
| Mob1b | 0,4218 | 8,58E-02 |
| Ppargc1b | -0,9309 | 8,58E-02 |
| Mir142b | -3,6040 | 8,62E-02 |
| Trav14-1 | -5,9424 | 8,66E-02 |
| Ssc5d | -1,3437 | 8,66E-02 |
| Lamb1 | 0,4514 | 8,69E-02 |
| Hcst | -2,5472 | 8,69E-02 |
| RP23-82P18,4 | 1,1355 | 8,69E-02 |
| Prr33 | -1,9395 | 8,69E-02 |
| 1500012F01Rik | 0,5659 | 8,69E-02 |
| AA474331 | -0,5439 | 8,70E-02 |
| Flrt3 | 0,5497 | 8,70E-02 |
| Sp2 | -0,5054 | 8,72E-02 |
| Sorbs2 | 0,3357 | 8,73E-02 |
| Gm12060 | 0,6968 | 8,74E-02 |
| Slco2b1 | -1,0009 | 8,74E-02 |
| Sytl3 | 0,5384 | 8,74E-02 |
| Spry2 | 0,4321 | 8,75E-02 |
| Pde1b | -1,3126 | 8,77E-02 |
| Iglv2 | -6,0297 | 8,77E-02 |
| Tdp1 | -0,6615 | 8,79E-02 |
| Erlin2 | 0,3880 | 8,79E-02 |
| Igkv6-17 | -4,5763 | 8,79E-02 |
| Dram1 | -0,9835 | 8,84E-02 |
| Hsd17b11 | -0,4559 | 8,88E-02 |
| Serpina3n | -0,5859 | 8,89E-02 |
| Dip2b | 0,3296 | 8,89E-02 |
| Lect1 | 0,3209 | 8,93E-02 |
| Vipr1 | -0,5136 | 8,94E-02 |
| Pag1 | 0,4357 | 8,97E-02 |
| Gbp6 | -0,7217 | 8,97E-02 |
| 5830418P13Rik | -5,2810 | 8,97E-02 |
| Zranb2 | 0,3434 | 9,01E-02 |
| Mbnl3 | -0,7293 | 9,01E-02 |
| Nrros | -0,8809 | 9,04E-02 |
| Wtap | 0,3344 | 9,05E-02 |
| Slco1a5 | 0,4142 | 9,11E-02 |
| Rapgef4os3 | -2,1761 | 9,11E-02 |
| Gm17334 | -1,6974 | 9,11E-02 |
| Gm29157 | 2,1627 | 9,12E-02 |
| Nrp2 | -0,5538 | 9,14E-02 |
| Arid5a | -0,4145 | 9,14E-02 |
| Tlr11 | -3,7324 | 9,14E-02 |
| Tert | -3,5030 | 9,16E-02 |
| Aknaos | -3,2103 | 9,19E-02 |
| RP24-273P12,4 | -0,7494 | 9,19E-02 |
| Xylt1 | -1,1828 | 9,22E-02 |
| Cdc42se2 | -0,4700 | 9,23E-02 |
| Il20rb | 0,4251 | 9,23E-02 |
| H1fx | -2,7960 | 9,31E-02 |
| Gm16685 | -3,3061 | 9,31E-02 |
| Cd244 | -1,3045 | 9,32E-02 |
| Slc15a3 | -1,1893 | 9,34E-02 |
| Shroom4 | -0,5126 | 9,35E-02 |
| Entpd2 | -3,2562 | 9,35E-02 |
| Arid3a | -0,6840 | 9,38E-02 |
| Fhdc1 | 0,3327 | 9,40E-02 |
| Evi5 | 0,4159 | 9,42E-02 |
| Ptgdr2 | -4,2962 | 9,42E-02 |
| Ssr2 | -0,4584 | 9,43E-02 |
| Kcnk1 | 0,3946 | 9,43E-02 |
| Gbp4 | -1,5942 | 9,44E-02 |
| Gm15872 | -2,3897 | 9,47E-02 |
| Ighv1-22 | -5,0994 | 9,51E-02 |
| Mapk3 | 0,3702 | 9,51E-02 |
| Lama1 | -1,4630 | 9,52E-02 |
| Nlrc4 | -1,5052 | 9,52E-02 |
| 4931429I11Rik | -3,0745 | 9,55E-02 |
| Nfam1 | -0,8850 | 9,59E-02 |
| Slc12a4 | -0,8700 | 9,60E-02 |
| Figf | 2,0637 | 9,63E-02 |
| Ighv1-50 | -5,8657 | 9,68E-02 |
| Gzma | -6,0991 | 9,74E-02 |
| Schip1 | 1,5843 | 9,75E-02 |
| Tmeff1 | -2,0195 | 9,81E-02 |
| 6530402F18Rik | -1,3947 | 9,81E-02 |
| RP23-388I22,1 | -1,6879 | 9,81E-02 |
| Stk4 | -0,4324 | 9,85E-02 |
| Egfros | 0,5678 | 9,88E-02 |
| Hmga2 | -1,6065 | 9,90E-02 |
| Nt5dc3 | 0,4205 | 9,90E-02 |
| Htra1 | -0,8488 | 9,91E-02 |
| Parp8 | -0,4922 | 9,92E-02 |
| Traj4 | -5,8891 | 9,92E-02 |
| Gm10524 | 0,5659 | 9,95E-02 |
| Lamc2 | 0,4308 | 9,95E-02 |
| Epb4,1l2 | -0,3540 | 1,00E-01 |
| Nap1l3 | -0,7536 | 1,00E-01 |
| Cnr1 | -4,2658 | 1,01E-01 |
| Cpt1a | -0,6686 | 1,01E-01 |
| Hk1 | 0,3257 | 1,01E-01 |
| Hmcn1 | -0,8592 | 1,01E-01 |
| Nlrc3 | -2,0124 | 1,02E-01 |
| Plod1 | 0,4483 | 1,02E-01 |
| Gm22579 | -0,7210 | 1,02E-01 |
| Lrrc8d | 0,3303 | 1,02E-01 |
| Rab8b | -0,4621 | 1,02E-01 |
| Mmp10 | -3,8010 | 1,03E-01 |
| Ank3 | 0,3389 | 1,03E-01 |
| Pcdhgb4 | 0,6311 | 1,03E-01 |
| Adrbk2 | -0,8932 | 1,03E-01 |
| Gm12064 | -1,8078 | 1,03E-01 |
| Serinc1 | 0,3508 | 1,03E-01 |
| Tgfbr2 | -0,3084 | 1,04E-01 |
| BC023105 | -0,8259 | 1,04E-01 |
| Gm15356 | -2,1672 | 1,04E-01 |
| Lpp | 0,2940 | 1,04E-01 |
| Fgfr3 | -2,5602 | 1,04E-01 |
| Mir143hg | 0,4929 | 1,04E-01 |
| Slc7a2 | -0,9685 | 1,04E-01 |
| Socs1 | -1,4645 | 1,04E-01 |
| Syne1 | -0,3699 | 1,05E-01 |
| Capn5 | 0,3906 | 1,05E-01 |
| Fendrr | -4,2063 | 1,05E-01 |
| Oaf | -1,0073 | 1,05E-01 |
| Slit2 | -0,6074 | 1,05E-01 |
| Olfr755-ps1 | -3,7843 | 1,06E-01 |
| Ighv1-9 | -1,9378 | 1,06E-01 |
| Asb2 | -1,2335 | 1,07E-01 |
| A530099J19Rik | -3,0252 | 1,07E-01 |
| Bmpr1b | -4,1653 | 1,07E-01 |
| Krt8 | 0,4165 | 1,07E-01 |
| Myb | -0,6521 | 1,07E-01 |
| Bnip3l | 0,4078 | 1,07E-01 |
| Brinp3 | -2,0541 | 1,08E-01 |
| Pcdhb16 | -0,7204 | 1,08E-01 |
| Gm13152 | -4,1262 | 1,08E-01 |
| Slc12a2 | 0,3668 | 1,08E-01 |
| A630072L19Rik | -1,6668 | 1,08E-01 |
| Mmp13 | -1,0665 | 1,08E-01 |
| Pgap1 | -0,5879 | 1,09E-01 |
| Phldb2 | -0,6112 | 1,09E-01 |

**Appendix Table S5 List of antibodies for flow cytometry**

| **Antibody** | **Color** | **Reference** | **Clone** | **µg / application** |
| --- | --- | --- | --- | --- |
| B220 | APC | 17-0452-82 | RA3-6B2 | 0,25 |
| CD11b | AF700 | 56-0112-80 | M1/70 | 0,25 |
| CD11c | PE | 12-0114-81 | N418 | 0,5 |
| CD206 | PE-Cy7 | 25-2061-80 | MR6F3 | 0,25 |
| CD3e | PE | 12-0031-81 | 145-2C11 | 0,5 |
| CD4 | APC-e780 | 47-0041-80 | GK1.5 | 0,125 |
| CD45 | FITC | 11-0451-82 | 30-F11 | 0,5 |
| CD80 | PerCP-e710 | 46-0801-80 | 16-10A1 | 0,125 |
| CD86 | PE-Cy7 | 25-0862-80 | GL1 | 0,25 |
| CD8a | APC | 17-0081-81 | 53-6.7 | 0,125 |
| CTLA4 | PE-Cy7 | 25-1522-80 | UC10-4B9 | 0,5 |
| F4/80 | APC- e780 | 47-4801-82 | BM8 | 0,5 |
| GZMB | PE-Cy7 | 25-8898-80 | NGZB | 0,125 |
| IFNy | PerCP Cy5.5 | 45-7311-80 | XMG1.2 | 0,25 |
| Ly-6G | PerCP e710 | 46-9668-80 | 1A8-Ly6g | 0,125 |
| MHCII | APC- e780 | 47-5321-80 | M5/114.15.2 | 0,5 |
| NK1.1 | PE | 12-5941-81 | PK136 | 0,25 |
| PD1 | PerCP Cy5.5 | 46-9985-80 | J43 | 0,125 |

All antibodies were purchased from ThermoFischer Scientific.

**Appendix Table S6 List of antibodies for tissue staining**

| **Antigen** | **Host** | **Antibody reference** | **Company** | **Dilution** |
| --- | --- | --- | --- | --- |
| TNC | Rat | MTn12 (Aufderheide and Ekblom 1988) |  | 2 µg/mL |
| TNC2.1 | Rabbit | Spenle et al., 2015 | G.Orend Lab | 1/1000 |
| TNC | Rabbit | Ab19011 | Merck Millipore | 1/150 |
| ErbB2 | Rabbit | MA5-13675 | ThermoFisher Scientific | 1/50 |
| Cytokeratin  CK8/18 | Guinea pig | GP11 | Progen | 1/500 |
| Pan-laminin | Rabbit | Ln6 7s (Simo et al. 1992) |  | 1/2000 |
| Collagen IV | Rabbit | (De Arcangelis et al. 1996) |  | 1/200 |
| Fibronectin | Rabbit | F3648 | Sigma | 1/200 |
| E-cadherin | Rat | 13-1900 | Life technology | 1/200 |
| Vimentin | Rabbit | 2707-1 | Epitomics | 1/500 |
| Caspase-3  cleaved | Rabbit | 9661 | Cell signaling | 1/500 |
| Ki-67 | Rabbit | RM-9106 | Thermofisher | 1/600 |
| CD8a | Rat | 550281 | BD Pharmingen | 1/400 |
| CD4 | Rat | 553727 | BD Pharmingen | 1/800 |
| CD45 | Rat | 550566 | BD Pharmingen | 1/500 |
| F4/80 | Rat | MCA497G | AbD serotec | 1/50 |
| CD11c | Hamster | 550283 | BD Pharmingen | 1/50 |
| CXCL12 | Rabbit | Ab9797 | Abcam | 1/1000 |
| Anti-rat IgG | Goat | A11006 | Jackson Lab | 1/1000 |
| Anti-rabbit IgG | Goat | 111-165-003 | Jackson Lab. | 1/1000 |
| Anti-Armenian Hamster IgG | Goat | 127-005-160 | Jackson Lab | 1/1000 |
| Anti-guinea Pig | Donkey | 706-165-148 | Jackson Lab | 1/1000 |
| Anti-goat IgG | Donkey | 705-165-147 | Jackson Lab | 1/1000 |

List of antibodies used for immunofluorescence tissue staining at the indicated dilutions.

**Appendix Table S7 List of primers**

| **Gene** | **Forward primer (5’ to 3’)** | **Reverse primer (5’ to 3’)** |
| --- | --- | --- |
| ErbB2 | CCTGCCCTCTGAGACTGATG | CAAGTACTCGGGGTTCTCCA |
| Granzyme B | Taqman probe: Mm00442837_m1 Thermofisher | |
| Perforin | Taqman probe: Mm00812512_m1 Thermofisher | |
| CXCL12 | Taqman probe: Mm00445553_m1 Thermofisher | |
| CXCR4 | Taqman probe: Mm01996749_s1 Thermofisher | |
| CXCR7 | Taqman probe: Mm00442837_m1 Thermofisher | |
| BAX | Taqman probe: Mm00432051_m1 Thermofisher | |
| BCL-2 | Taqman probe: Mm00477631_m1 Thermofisher | |
| BCL-xl | Taqman probe: Mm00437783_m1 Thermofisher | |
| IFNγ | Taqman probe: Mm01168134_m1 Thermofisher | |
