# Supplementary material for: Tenascin‐C immobilizes infiltrating T lymphocytes through CXCL12 promoting breast cancer progression
Source: EMBO Mol Med. 2021 May 14;13(6):e13270. doi: 10.15252/emmm.202013270 (PMC8185552; doi:10.15252/emmm.202013270)
Supplement: Supplementary file 2 — Expanded View Figures PDF [file EMMM-13-e13270-s002.pdf]

Expanded View Figures

Figure EV1. Loss of the TNC protein in both cancer and stromal cells impacts tumor gene expression.

A Schematic depiction of the experimental setup in NeuNT mice with a WT or TNCKO background.
B Juxtaposed heatmaps representing RNA chip analysis data of MMTV-NeuNT tumors (WT, TNCKO; *N* = 3).
C Schematic depiction of the experimental setup in the NT193 grafting model followed by analysis of the tumor and the lungs as indicated. In the grafting experiments, four conditions were used where shC or shTNC tumor cells (sh1, sh2) were engrafted into the mammary gland of a WT or TNCKO (KO) host giving rise to TNC-high (WT/shC) and TNC-low tumors (WT/shTNC, KO/shC, KO/shTNC).
D Juxtaposed heatmaps representing the RNA sequencing results from NT193 tumors (WT and TNCKO mice injected with shC or sh2TNC cells; *N* = 2). Conditions further used in the study are shown in bold as TNC high and TNC low, respectively. Note, juxtaposition shows similarities in the pattern of gene expression between TNC-high (MMTV-NeuNT/WT, WT/shC) tumors (blue squares) (B) and TNC-low (MMTV-NeuNT/KO, WT/shTNC, KO/shTNC) tumors (yellow squares) (D).

© 2021 The Authors

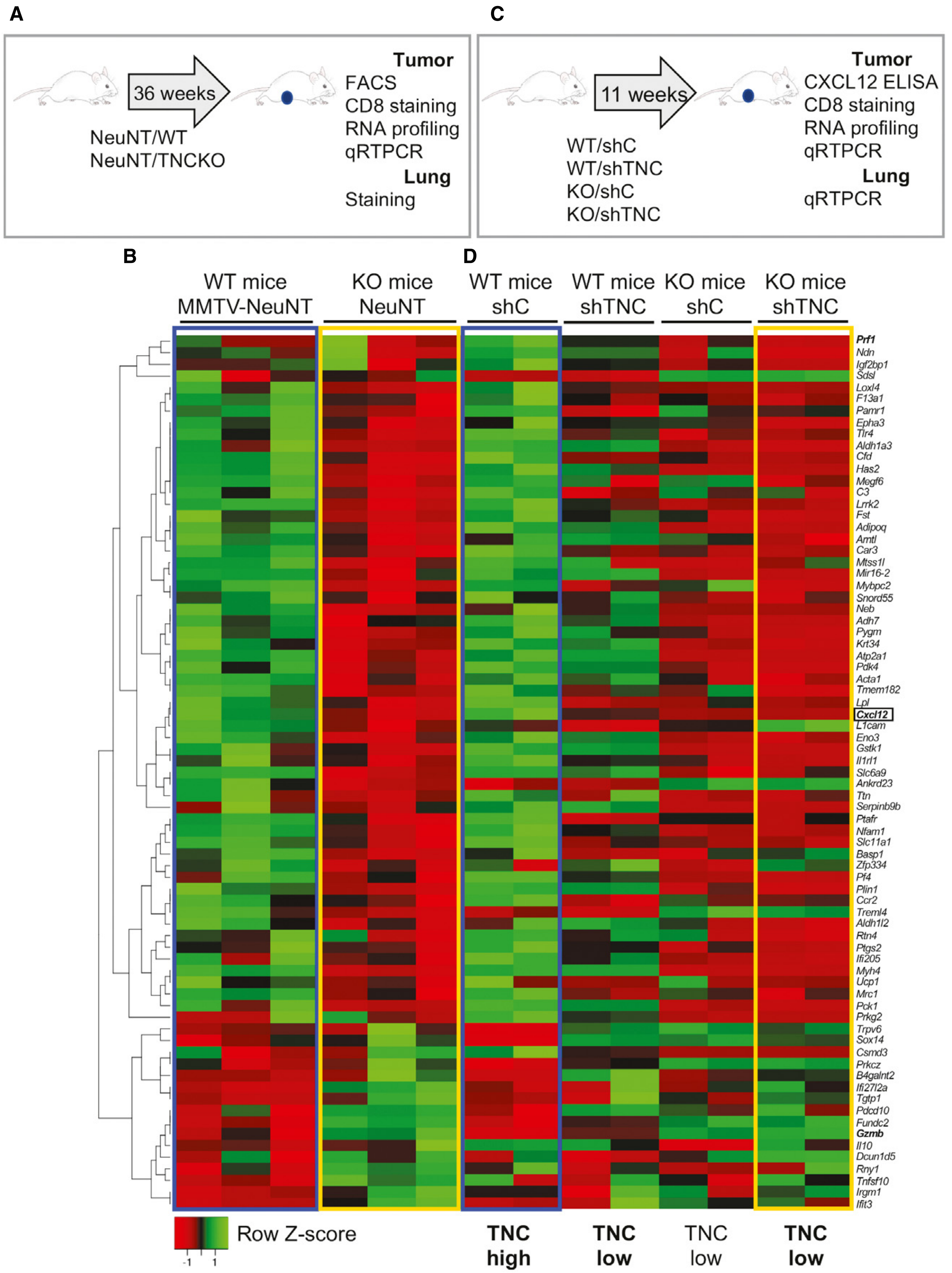
A
36 weeks
NeuNT/WT
NeuNT/TNCKO
Tumor
FACS
CD8 staining
RNA profiling
qRTPCR
Lung
Staining
C
11 weeks
WT/shC
WT/shTNC
KO/shC
KO/shTNC
Tumor
CXCL12 ELISA
CD8 staining
RNA profiling
qRTPCR
Lung
qRTPCR
B
D
WT mice MMTV-NeuNT
KO mice NeuNT
WT mice shC
WT mice shTNC
KO mice shC
KO mice shTNC
Prf1
Ndn
Igf2bp1
Sdsl
Loxl4
F13a1
Pamr1
Epha3
Tlr4
Aldh1a3
Cfd
Has2
Megf6
C3
Lrrk2
Fst
Adipoq
Arntl
Car3
Mtss1l
Mir16-2
Mybpc2
Snord55
Neb
Adh7
Pygm
Krt34
Atp2a1
Pdk4
Acta1
Tmem182
Lpl
Cxcl12
L1cam
Eno3
Gstk1
Il1rl1
Slc6a9
Ankrd23
Ttn
Serpinb9b
Ptafr
Nfam1
Slc11a1
Basp1
Zfp334
Pf4
Plin1
Ccr2
Treml4
Aldh1l2
Rtn4
Ptgs2
Ifi205
Myh4
Ucp1
Mrc1
Pck1
Prkg2
Trpv6
Sox14
Csmd3
Prkcz
B4galnt2
Ifi27l2a
Tgtp1
Pdcd10
Fundc2
Gzmb
Il10
Dcun1d5
Rny1
Tnfsf10
Irgm1
Ifit3
Row Z-score
-1
1
TNC high
TNC low
TNC low
TNC low

Figure EV1.

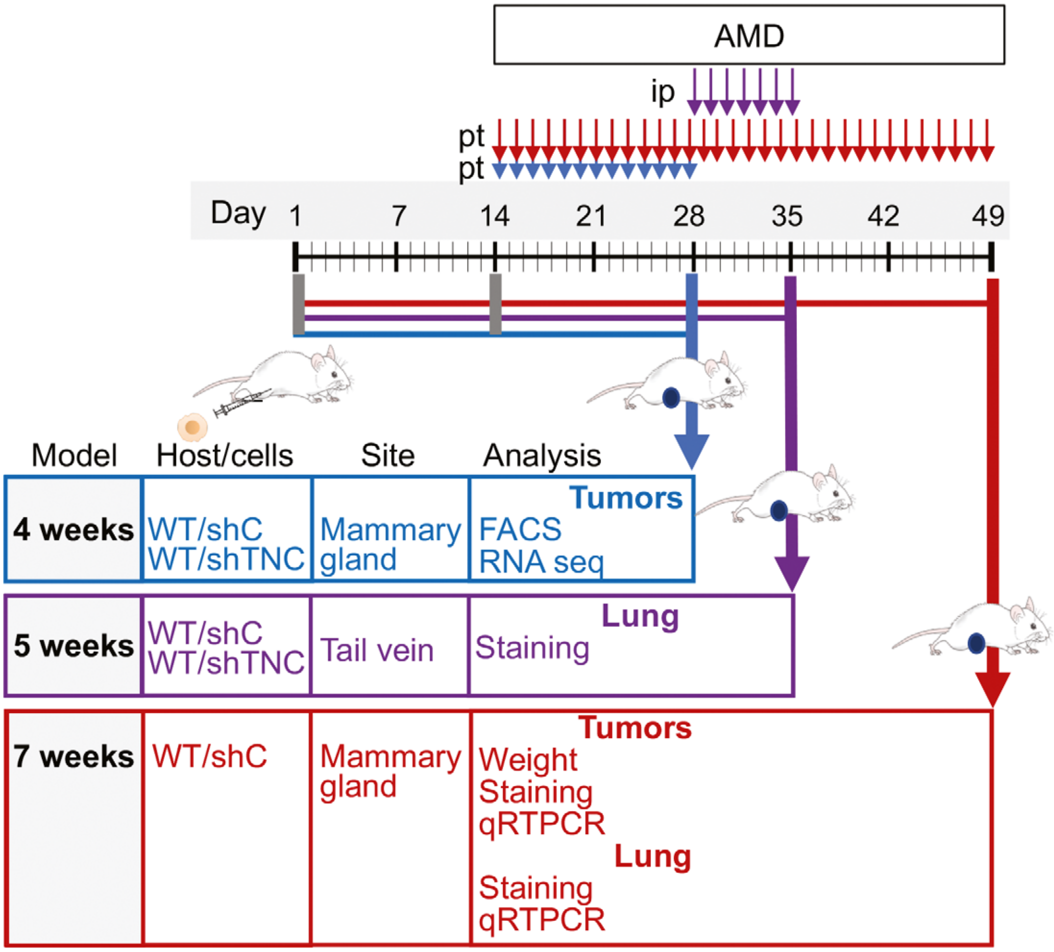

Figure EV2. Experimental setup of the CXCR4 inhibition experiments in the NT193 grafting model.

Experimental setup for the 4, 5, and 7 weeks protocols. 4-week model: WT mice were engrafted with shC or shTNC tumor cells in the mammary gland followed by AMD treatment (5 mg/kg/day, peritumoral (pt)) for 2 weeks (week 2–4) before sacrifice and investigation of the primary tumor by flow cytometry and RNA-seq (WT/shC and WT/shTNC). 5-week model: WT mice were iv engrafted with shC or shTNC tumor cells for 4 weeks followed by AMD treatment (7.5 mg/kg/day, ip) for 1 week (week 5) before sacrifice and analysis of the lungs by tissue staining. 7-week model: WT mice were engrafted with shC tumor cells into the mammary gland followed by AMD treatment (5 mg/kg/day, pt) for 5 weeks (week 2–7) before sacrifice and investigation of the tumor (weight, tissue staining, qRT–PCR) and the lungs (tissue staining, qRT–PCR).

Figure EV3. CXCL12 expression and binding to TNC.

A, B RNA-seq gene expression results represented as heatmap for the fifty most deregulated genes in TNC-high (WT/shC) and TNC-low (KO/shTNC) tumors (N = 2 tumors per condition), 11-week model (A) and in shC and shTNC cells (N = 2) (B).

C CXCL12 mRNA levels in NT193 tumors, 11-week model (N = 7 tumors for WT/shC, WT/sh1TNC, TNCKO/sh1TNC, and TNCKO/sh2TNC conditions, N = 8 tumors for WT/sh2TNC and TNCKO/shC conditions), *P = 0.0205, Mann–Whitney test. Mean ± SEM.

D CXCL12 mRNA levels in MMTV-NeuNT/WT compared to TNCKO tumors as determined by qRT–PCR (N = 13 and 6 tumors, respectively), *P = 0.0123, Mann–Whitney test. Mean ± SEM.

E–G Negative EM analysis of binding of unlabeled beads to TNC (E) and upon binding of CXCL12-adsorbed gold beads upon incubation with unlabeled CXCL12 (F) and heparin (G) at the indicated molar ratios to TNC. Asterisk points at fibrinogen globe. Scale bars, 100 nm (top), 50 nm (bottom), (E). Representation of TNC monomer, oligomerization domain (triangle) to form hexamers as seen in (E) FNIII repeats (gray boxes, constant domains, white boxes, alternative domains), fibrinogen like domain (circle). Representative result (3 independent experiments, n = 500 TNC molecules each). Mean ± SEM.

 © 2021 The Authors

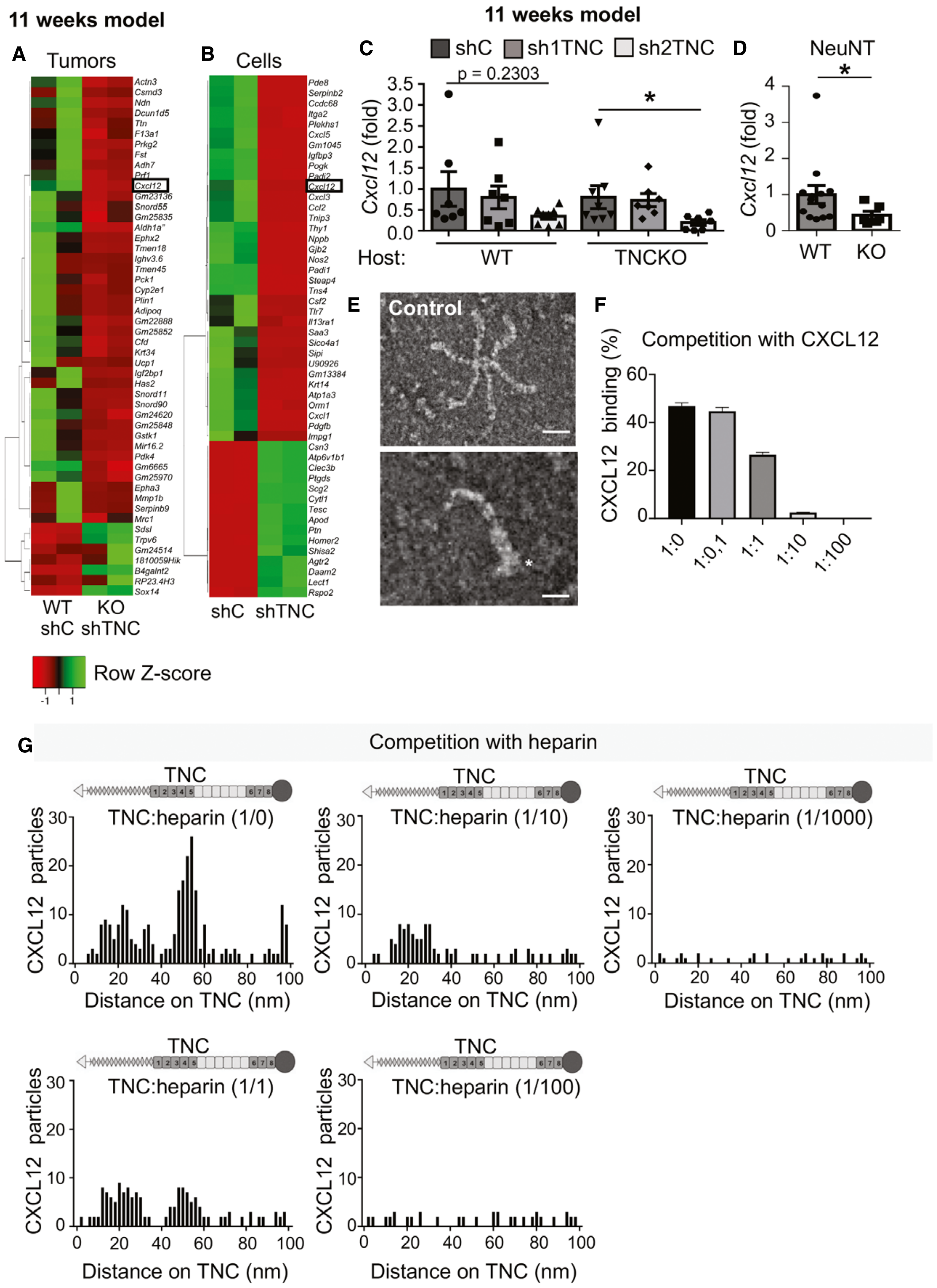
11 weeks model
A Tumors
B Cells
WT shC
KO shTNC
shC shTNC
Row Z-score
11 weeks model
C
shC sh1TNC sh2TNC
p = 0.2303
*
Cxcl12 (fold)
Host: WT TNCKO
D NeuNT
*
Cxcl12 (fold)
WT KO
E Control
F Competition with CXCL12
CXCL12 binding (%)
1:0 1:0,1 1:1 1:10 1:100
G Competition with heparin
TNC
TNC:heparin (1/0)
TNC:heparin (1/10)
TNC:heparin (1/1000)
TNC:heparin (1/1)
TNC:heparin (1/100)
CXCL12 particles
Distance on TNC (nm)

Figure EV3.

Figure EV4. Impact of CXCR4 on the tumor immune cell infiltrate and gene expression in the tumors.

A–G 4-week model. Abundance of the indicated immune cell subtypes per tumor as determined by flow cytometry. $N = 8$ tumors per condition. ns: $P > 0.05$, Mann–Whitney test. Mean ± SEM.

H 4-week model. IFNγ expression in MACS isolated CD8 T leukocytes as determined by flow cytometry. $N = 8$ tumors per group, $^{**}P = 0.0015$, unpaired *t*-test. Mean ± SEM.

I–K 7-week model. Expression of the indicated molecules in tumors as determined by qRT–PCR. $N = 14$ (PBS), $N = 16$ (AMD) tumors. ns: $P > 0.05$, unpaired *t*-test. Mean ± SEM.

© 2021 The Authors

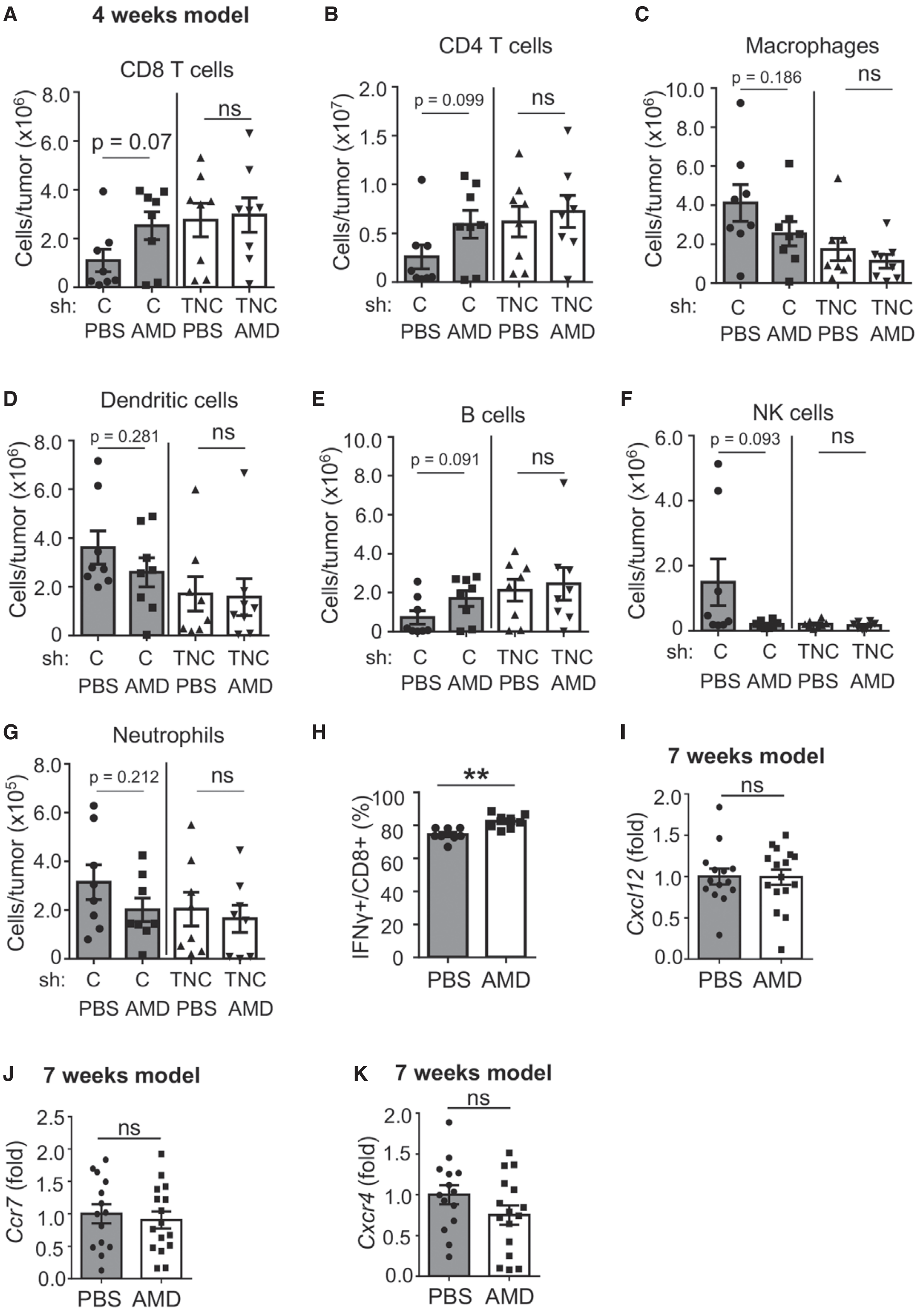
A
4 weeks model
CD8 T cells
Cells/tumor (x10^6)
p = 0.07
ns
sh: C C TNC TNC
PBS AMD PBS AMD
B
CD4 T cells
Cells/tumor (x10^7)
p = 0.099
ns
C
Macrophages
p = 0.186
D
Dendritic cells
p = 0.281
E
B cells
p = 0.091
F
NK cells
p = 0.093
G
Neutrophils
Cells/tumor (x10^5)
p = 0.212
H
**
IFNγ+/CD8+ (%)
PBS AMD
I
7 weeks model
Cxcl12 (fold)
J
7 weeks model
Ccr7 (fold)
K
7 weeks model
Cxcr4 (fold)

Figure EV4.

Figure EV5. Impact of CXCR4 on gene expression and abundance of macrophages.

A–E 4-week model. Gene set enrichment analysis of differentially expressed genes in TNC-high (WT/shC) tumors treated with PBS or AMD (A, B, D, E) and in WT/shC and WT/shTNC tumors treated with PBS (C, D, E). (A, C) False discovery rate (FDR) > 0.05 and ≤ 0.05. $N = 2$ tumors per group. RNA-seq gene expression results represented as heatmap for genes that belong to the angiogenesis pathways ($P = 2.31 \times 10^{-9}$) (B), immune suppression ($P = 7.87 \times 10^{-7}$) (D), IL-10 production and response signaling ($P = 8.12 \times 10^{-8}$) (E) according to the Panther software and Geneontology functional database.

F Analysis of *Cd274* levels by qRT–PCR in shC tumor cells upon treatment with the indicated inhibitors, $N = 6$ independent experiments, $^{**}P = 0.0022$, Mann–Whitney test. Mean ± SEM.

G, H 7-week model. Staining of tumor tissue (WT/shC) for the indicated molecules. Scale bar, 50 μm. $N = 5$ tumors, $n = 2$ slides per condition. Arrows point at F4/80$^+$ (G) and CD206$^+$ cells (H). Asterisks point at the F4/80$^+$ cells localized in the tumor cell nest (G).

© 2021 The Authors

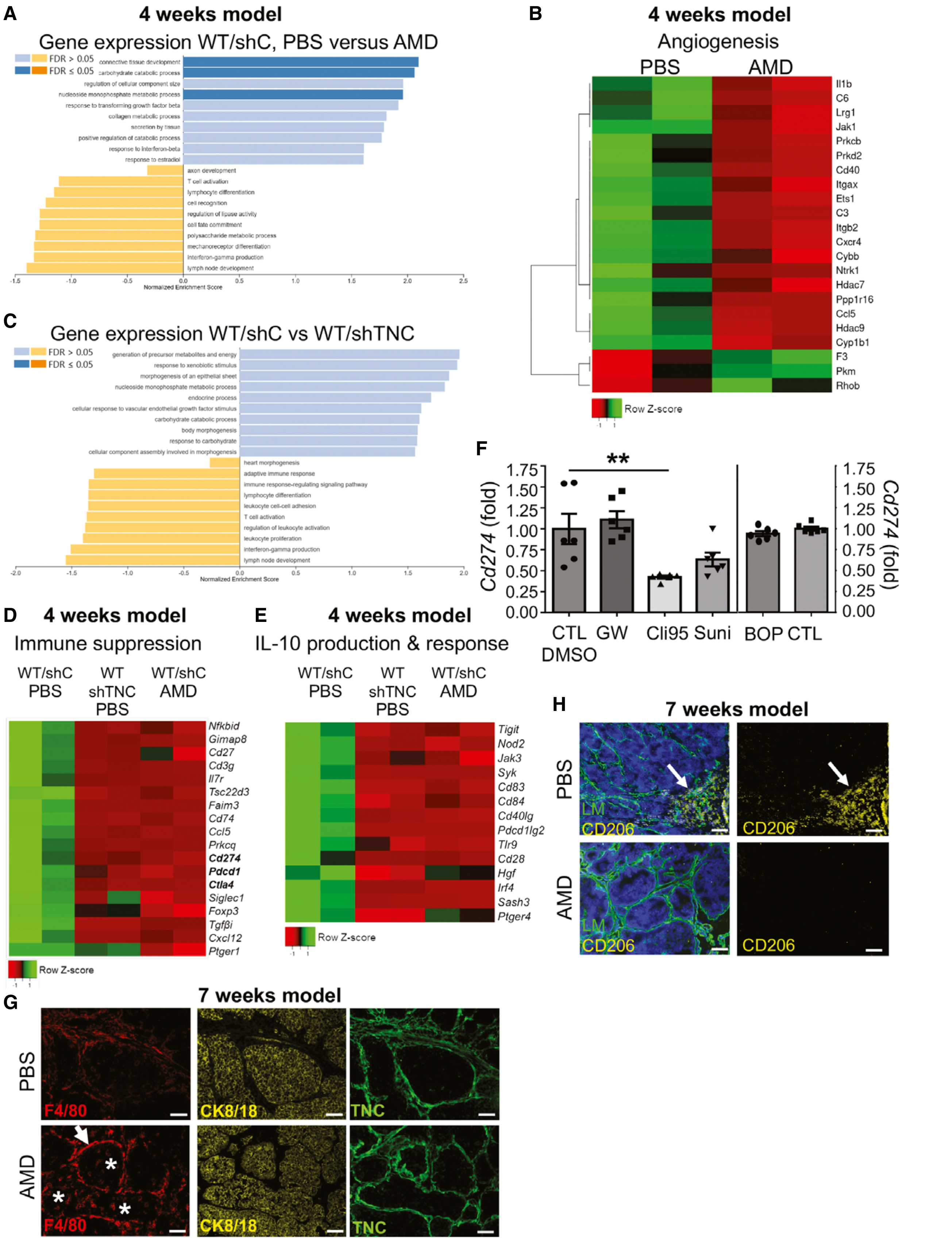
A
4 weeks model
Gene expression WT/shC, PBS versus AMD
FDR > 0.05
FDR ≤ 0.05
connective tissue development
carbohydrate catabolic process
regulation of cellular component size
nucleoside monophosphate metabolic process
response to transforming growth factor beta
collagen metabolic process
secretion by tissue
positive regulation of catabolic process
response to interferon-beta
response to estradiol
axon development
T cell activation
lymphocyte differentiation
cell recognition
regulation of lipase activity
cell fate commitment
polysaccharide metabolic process
mechanoreceptor differentiation
interferon-gamma production
lymph node development
Normalized Enrichment Score
B
4 weeks model
Angiogenesis
PBS
AMD
Il1b
C6
Lrg1
Jak1
Prkcb
Prkd2
Cd40
Itgax
Ets1
C3
Itgb2
Cxcr4
Cybb
Ntrk1
Hdac7
Ppp1r16
Ccl5
Hdac9
Cyp1b1
F3
Pkm
Rhob
Row Z-score
C
Gene expression WT/shC vs WT/shTNC
FDR > 0.05
FDR ≤ 0.05
generation of precursor metabolites and energy
response to xenobiotic stimulus
morphogenesis of an epithelial sheet
nucleoside monophosphate metabolic process
endocrine process
cellular response to vascular endothelial growth factor stimulus
carbohydrate catabolic process
body morphogenesis
response to carbohydrate
cellular component assembly involved in morphogenesis
heart morphogenesis
adaptive immune response
immune response-regulating signaling pathway
lymphocyte differentiation
leukocyte cell-cell adhesion
T cell activation
regulation of leukocyte activation
leukocyte proliferation
interferon-gamma production
lymph node development
Normalized Enrichment Score
F
Cd274 (fold)
**
CTL DMSO
GW
Cli95
Suni
BOP
CTL
D
4 weeks model
Immune suppression
WT/shC PBS
WT shTNC PBS
WT/shC AMD
Nfkbid
Gimap8
Cd27
Cd3g
Il7r
Tsc22d3
Faim3
Cd74
Ccl5
Prkcq
Cd274
Pdcd1
Ctla4
Siglec1
Foxp3
Tgfβi
Cxcl12
Ptger1
Row Z-score
E
4 weeks model
IL-10 production & response
WT/shC PBS
WT shTNC PBS
WT/shC AMD
Tigit
Nod2
Jak3
Syk
Cd83
Cd84
Cd40lg
Pdcd1lg2
Tlr9
Cd28
Hgf
Irf4
Sash3
Ptger4
Row Z-score
H
7 weeks model
PBS
AMD
LM
CD206
G
7 weeks model
PBS
AMD
F4/80
CK8/18
TNC

Figure EV5.
