# Supplementary figures and images for: Tenascin‐C immobilizes infiltrating T lymphocytes through CXCL12 promoting breast cancer progression
Source: EMBO Mol Med. 2021 May 14;13(6):e13270. doi: 10.15252/emmm.202013270 (PMC8185552; doi:10.15252/emmm.202013270)

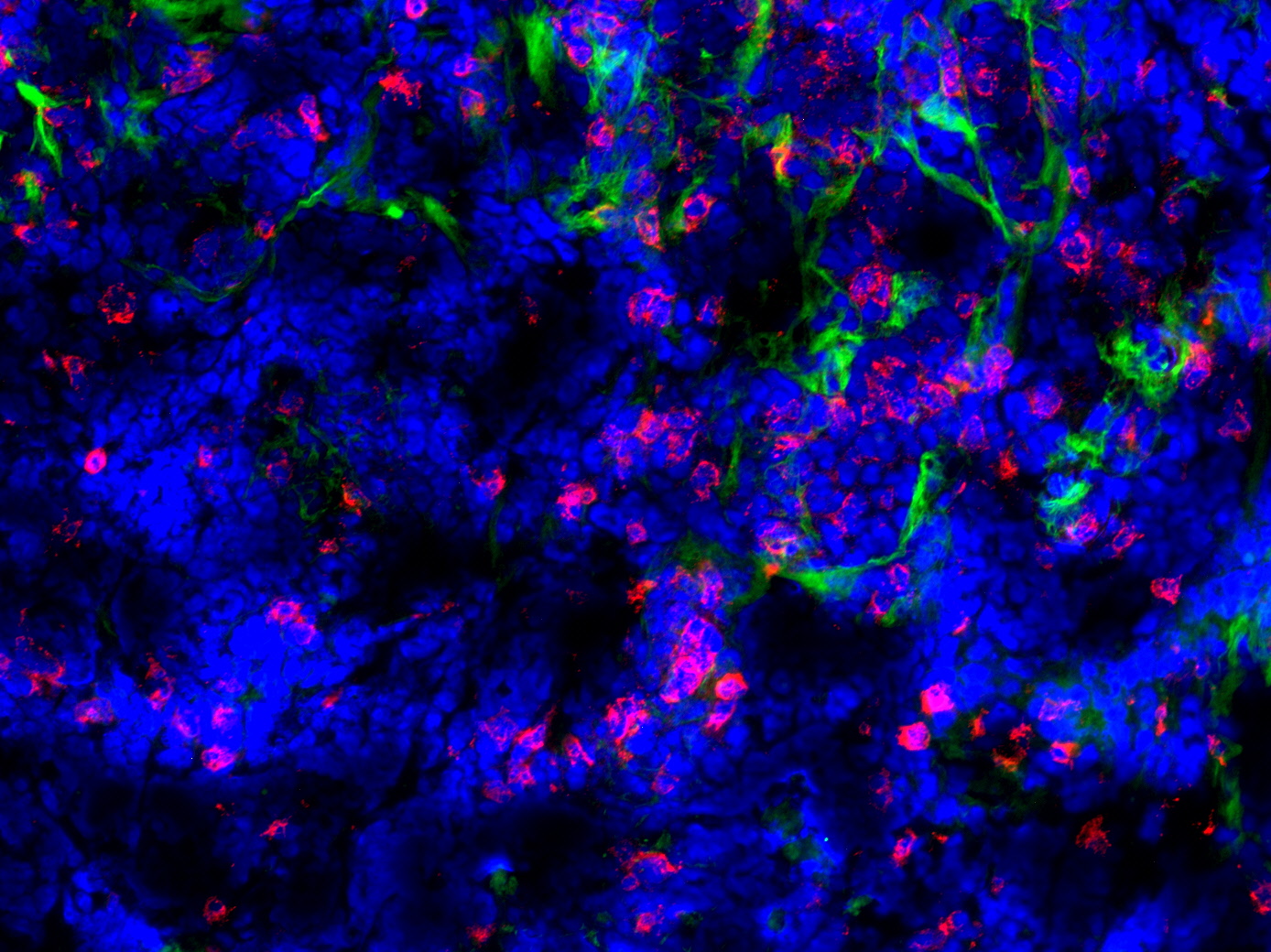

Supplement: Supplementary file 8 — Source Data for Figure 5 [file EMMM-13-e13270-s003.zip › Fig5/Fig5D/b22_s9_tnccd8_20x_7+_(DAPI+GFP+Cy3).JPG]

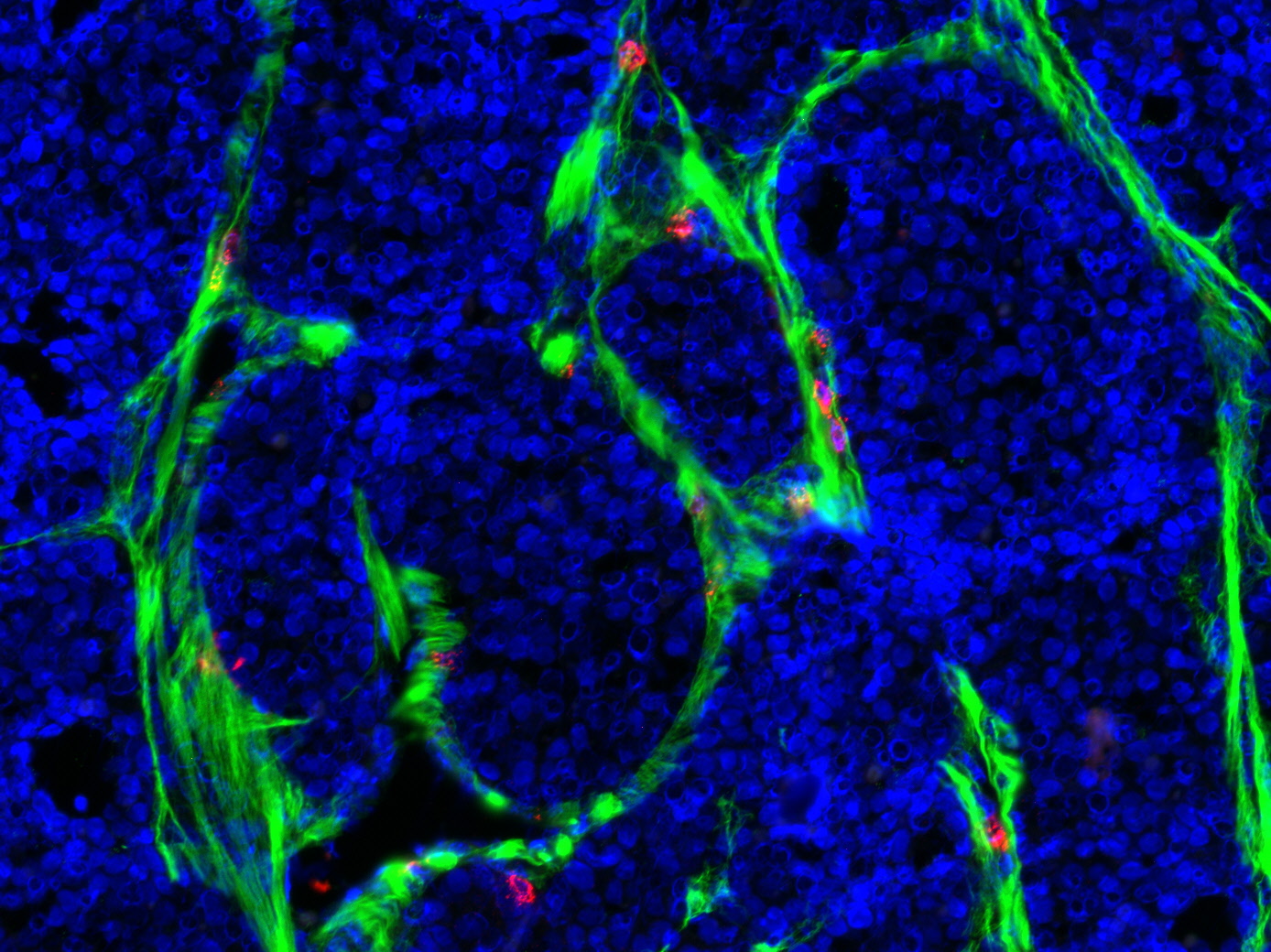

Supplement: Supplementary file 8 — Source Data for Figure 5 [file EMMM-13-e13270-s003.zip › Fig5/Fig5D/b33_s7_tnccd8_20x_6_(DAPI+GFP+Cy3).JPG]

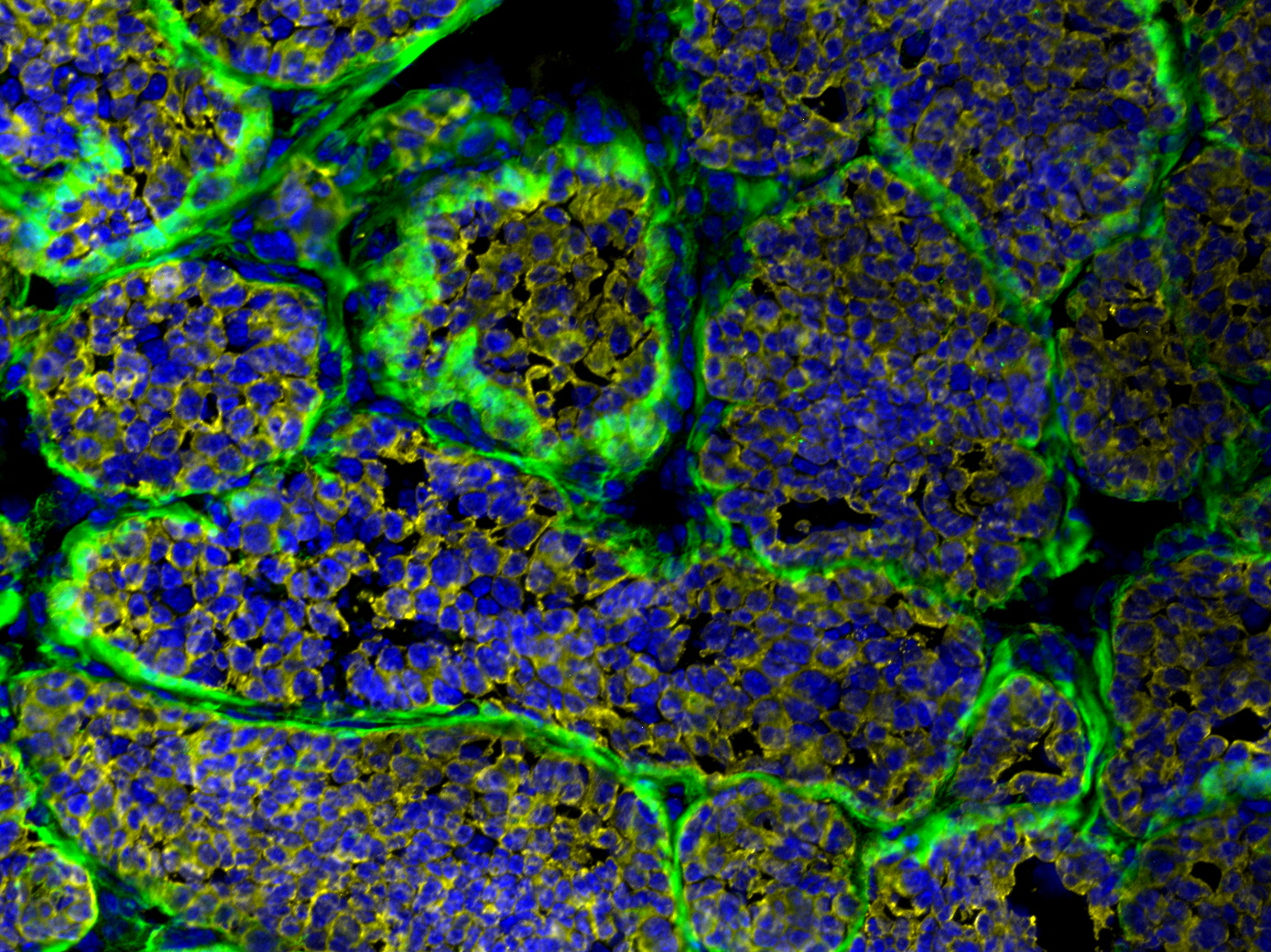

Supplement: Supplementary file 8 — Source Data for Figure 5 [file EMMM-13-e13270-s003.zip › Fig5/Fig5I/Fig 5I AMD CK818.JPG]

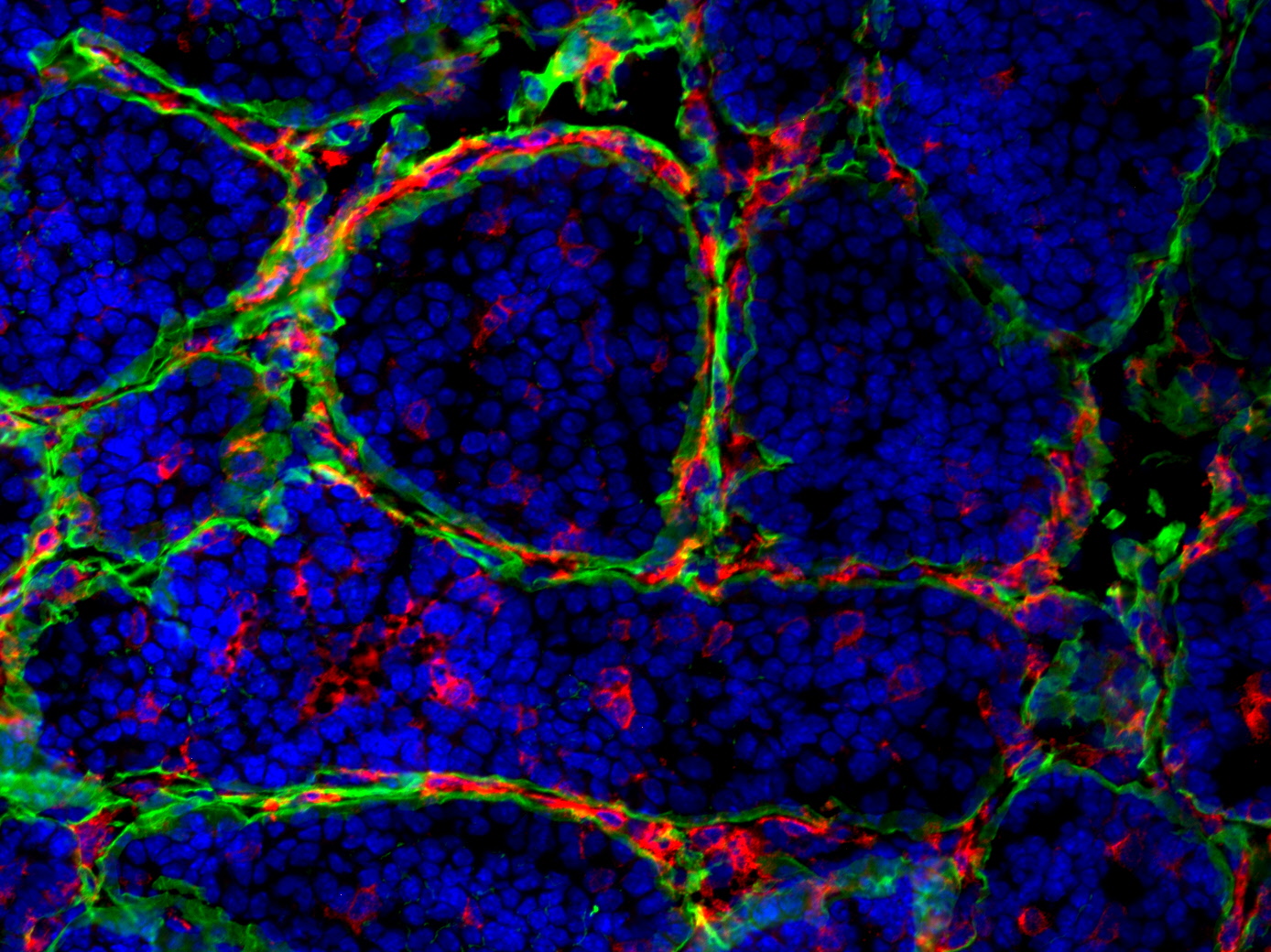

Supplement: Supplementary file 8 — Source Data for Figure 5 [file EMMM-13-e13270-s003.zip › Fig5/Fig5I/Fig 5I AMD.JPG]

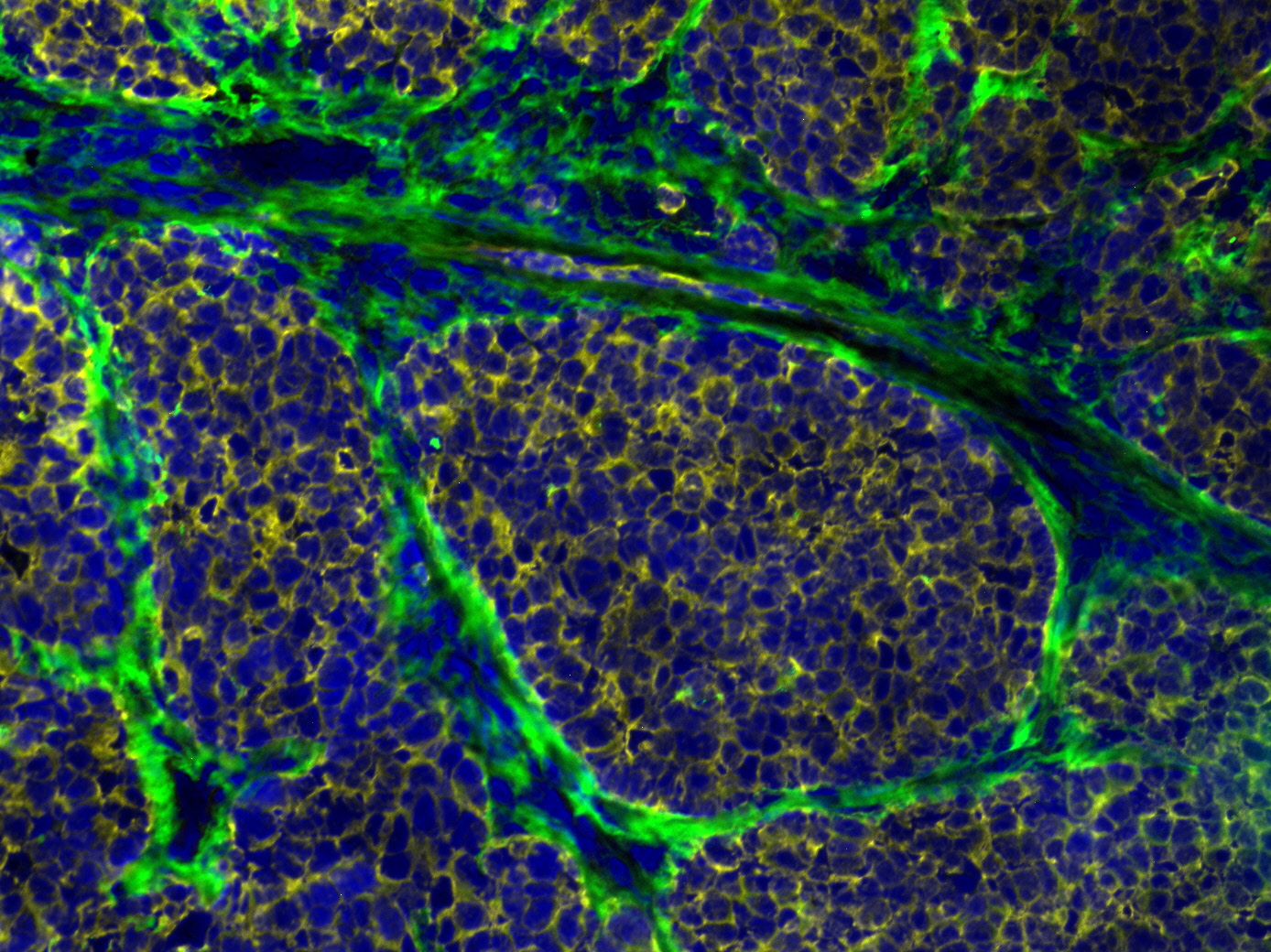

Supplement: Supplementary file 8 — Source Data for Figure 5 [file EMMM-13-e13270-s003.zip › Fig5/Fig5I/Fig 5I PBS CK818.JPG]

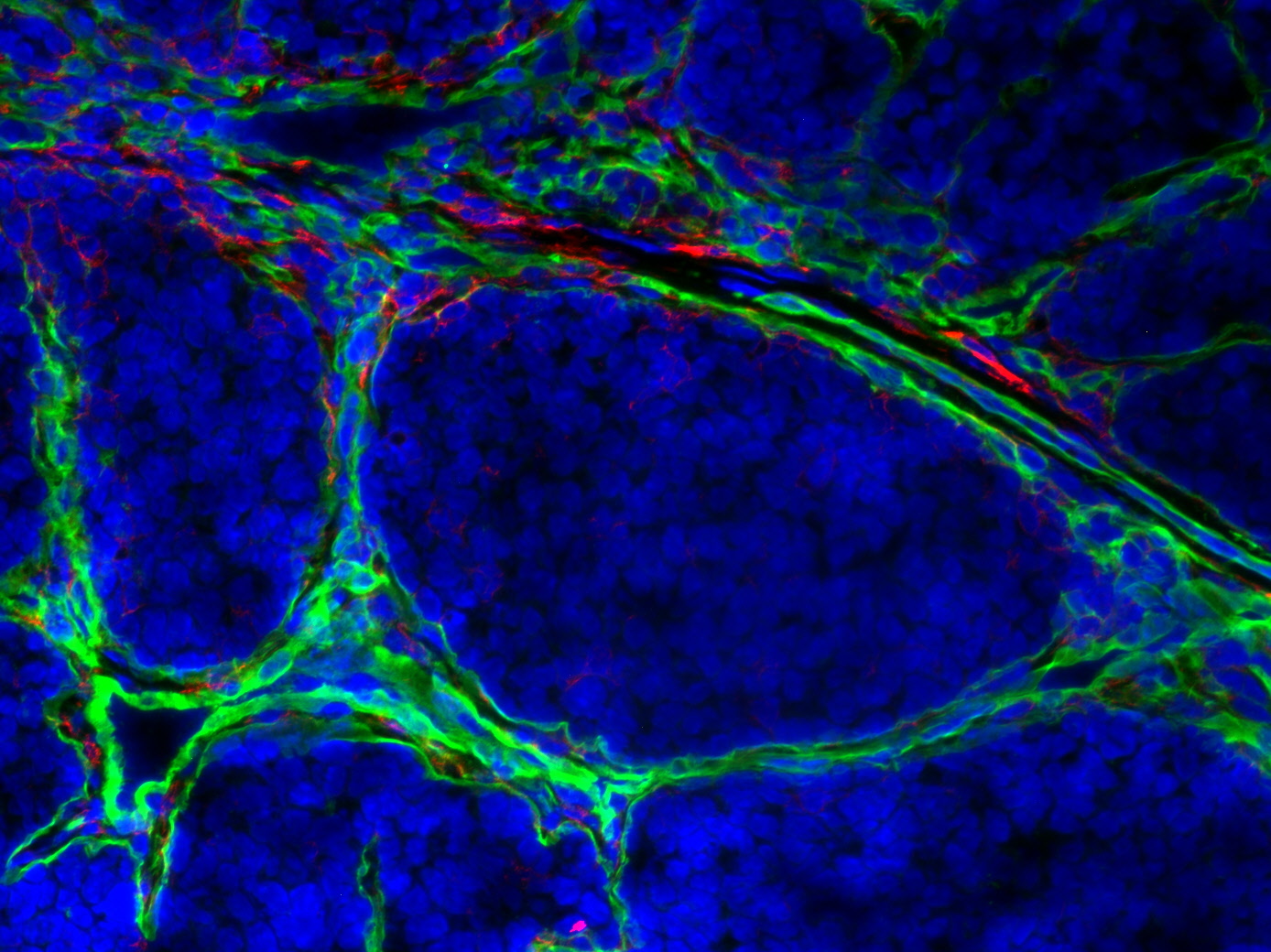

Supplement: Supplementary file 8 — Source Data for Figure 5 [file EMMM-13-e13270-s003.zip › Fig5/Fig5I/Fig 5I PBS.JPG]

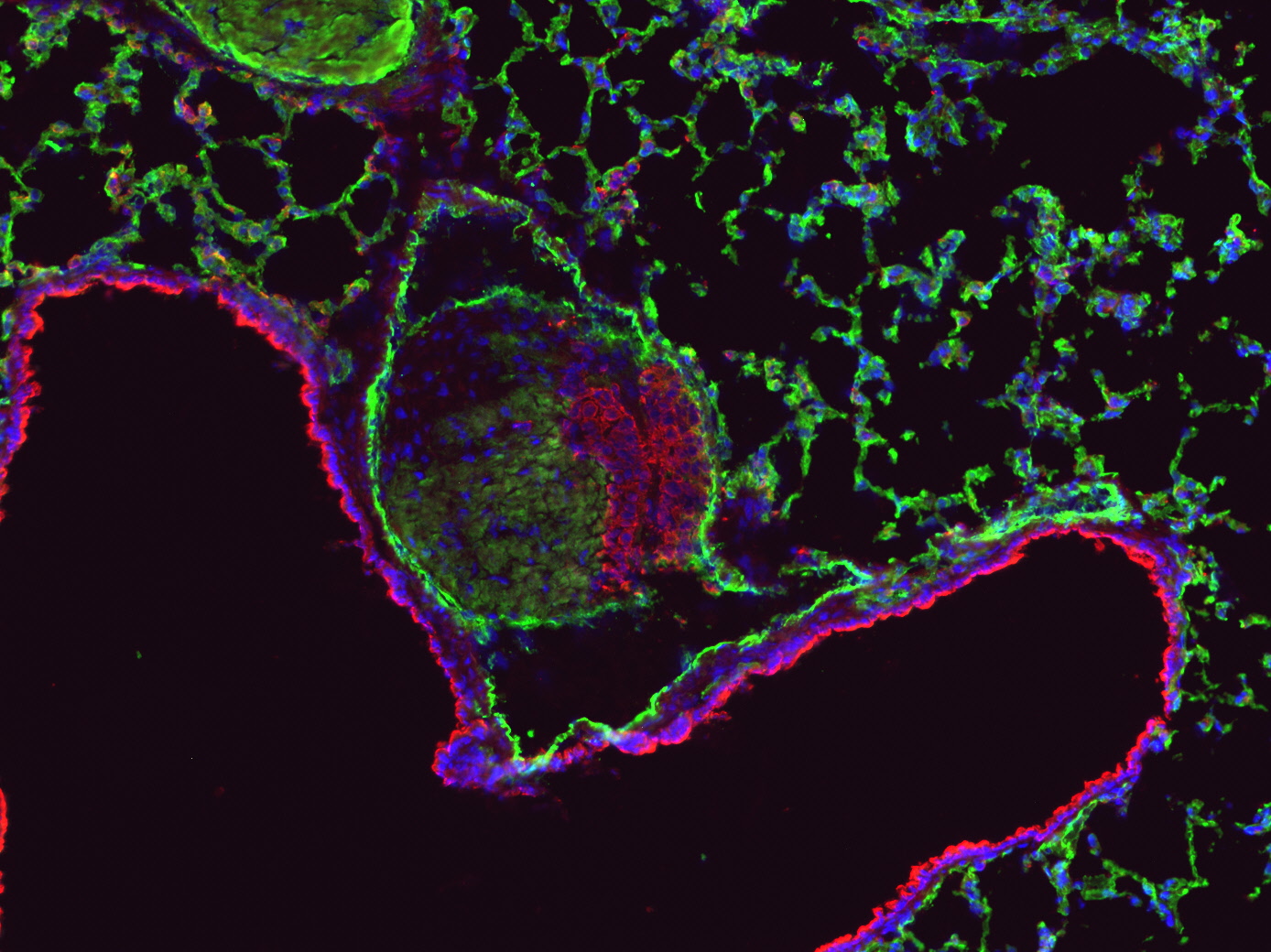

Supplement: Supplementary file 9 — Source Data for Figure 6 [file EMMM-13-e13270-s004.zip › Fig6/Fig6B/879 B lame 5 b Rouge CK8 18 vert CD31 blanc CC3_(DAPI+GFP+Cy3+Cy5).JPG]

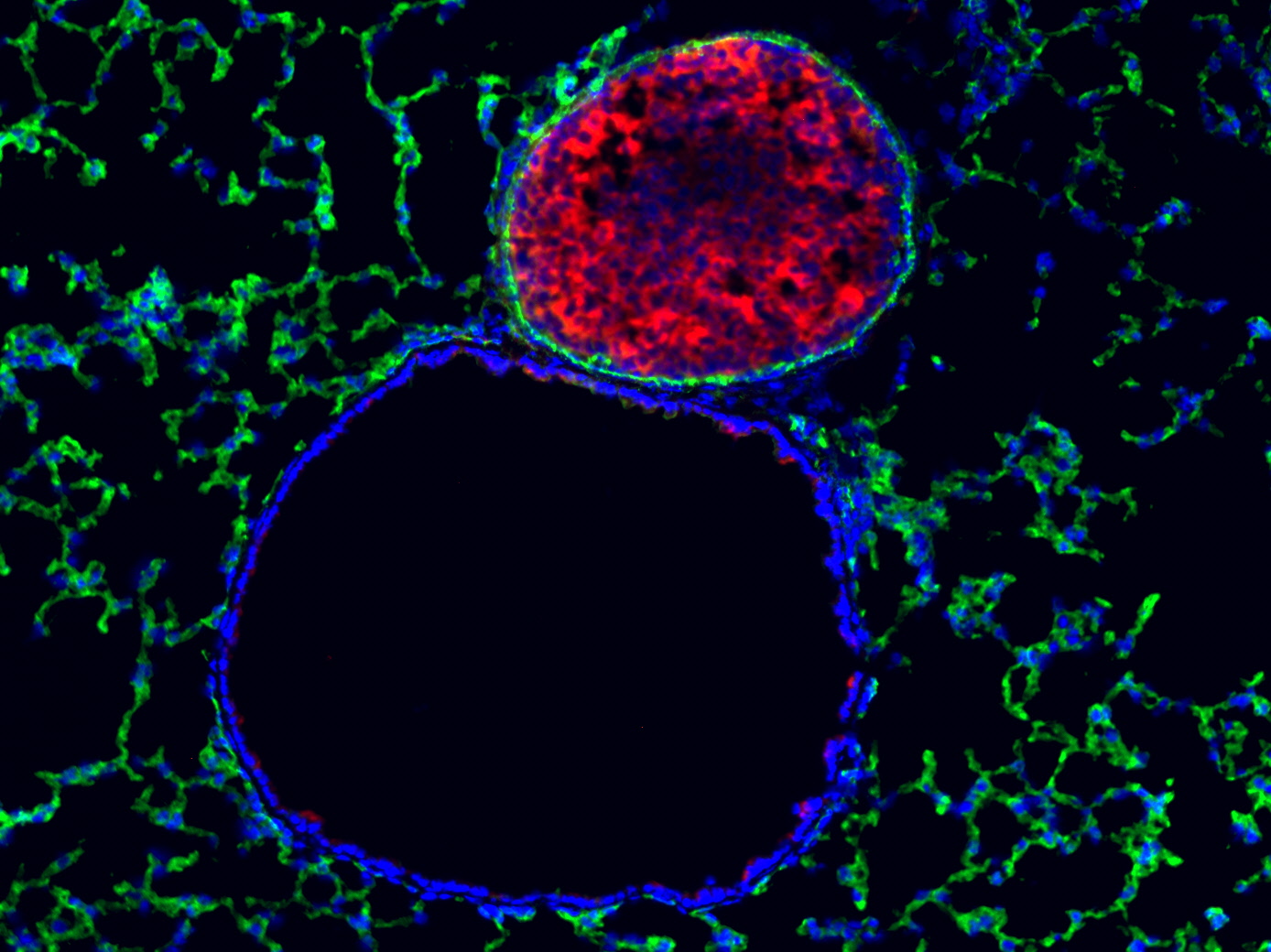

Supplement: Supplementary file 9 — Source Data for Figure 6 [file EMMM-13-e13270-s004.zip › Fig6/Fig6B/930 lung B f vert CD31 rouge HER2 29112018_(DAPI+Cy3+Cy5).JPG]

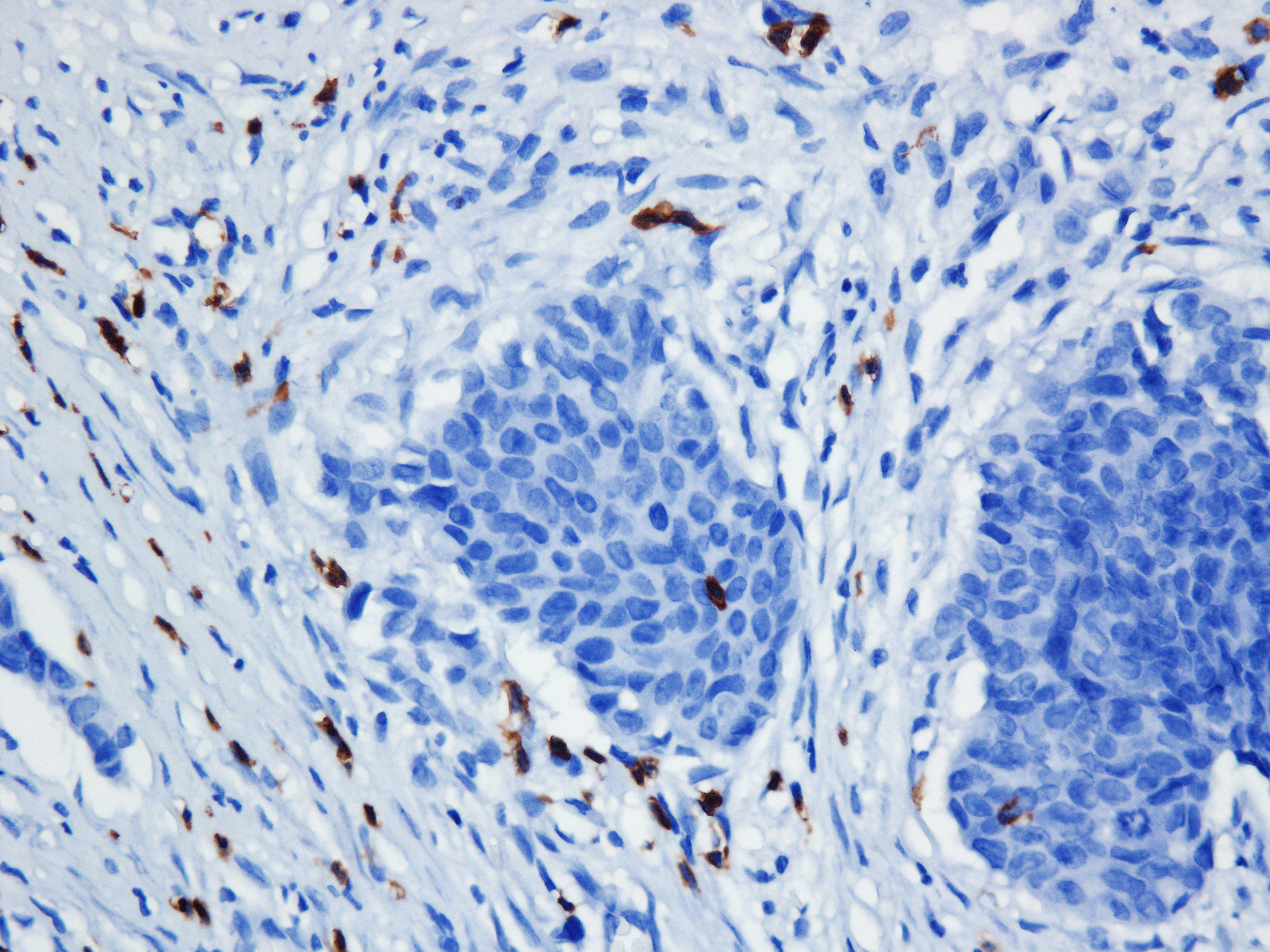

Supplement: Supplementary file 10 — Source Data for Figure 7 [file EMMM-13-e13270-s008.zip › Fig7/fig 7 A/1D3 CD8 400.jpg]

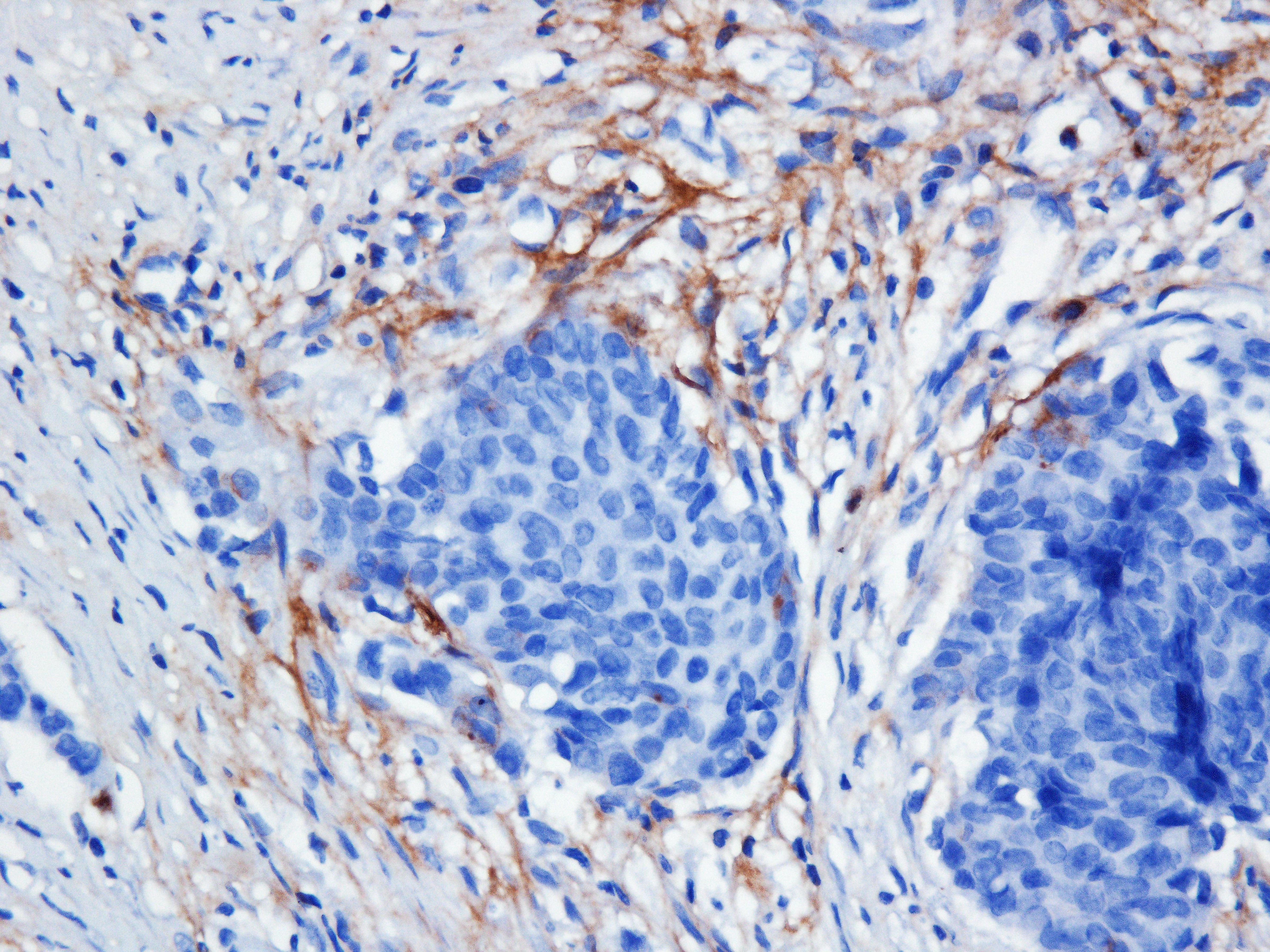

Supplement: Supplementary file 10 — Source Data for Figure 7 [file EMMM-13-e13270-s008.zip › Fig7/fig 7 A/1D3 TNC 400.jpg]

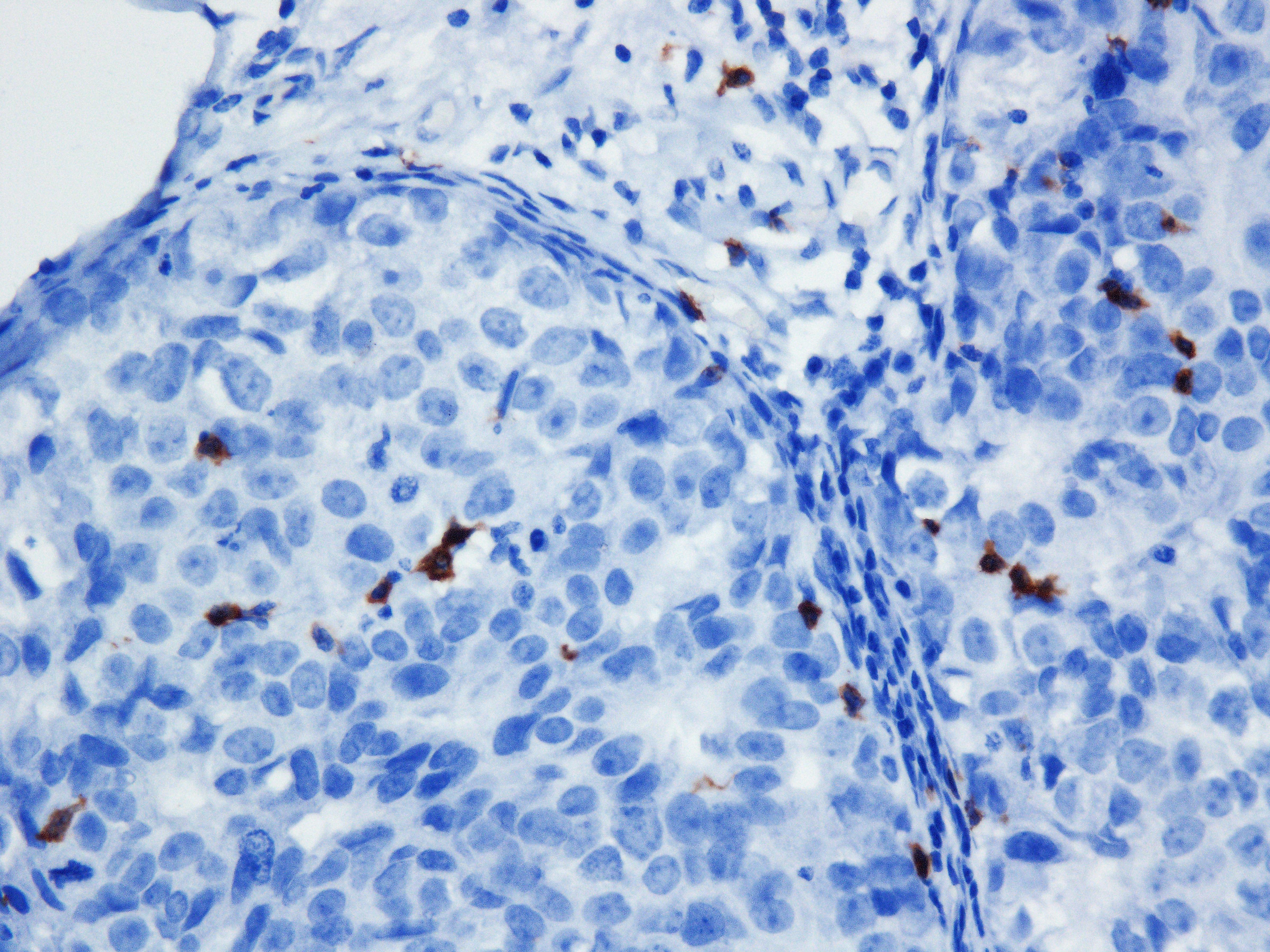

Supplement: Supplementary file 10 — Source Data for Figure 7 [file EMMM-13-e13270-s008.zip › Fig7/fig 7 A/201840000_5.jpg]

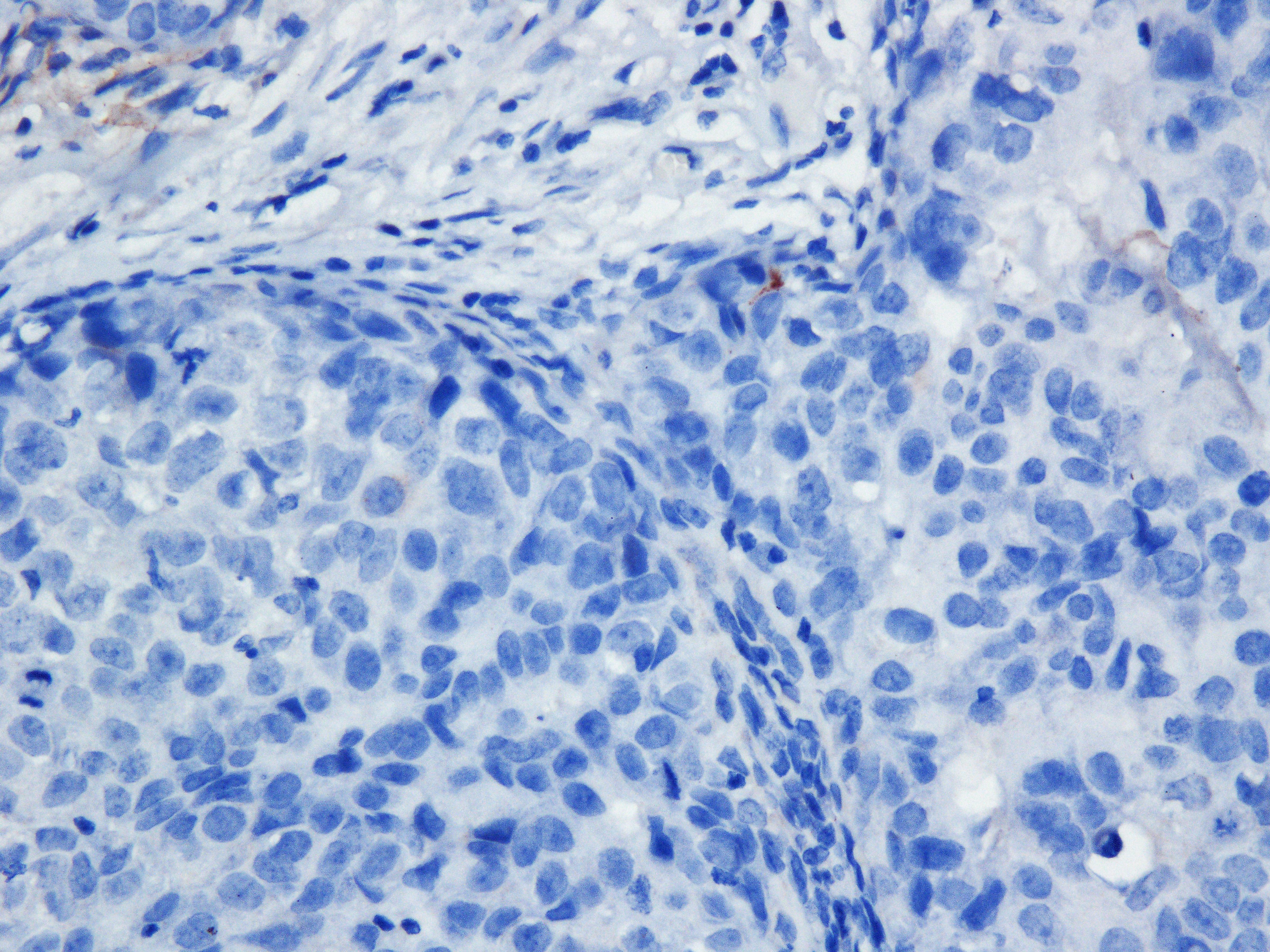

Supplement: Supplementary file 10 — Source Data for Figure 7 [file EMMM-13-e13270-s008.zip › Fig7/fig 7 A/201840000_8.jpg]

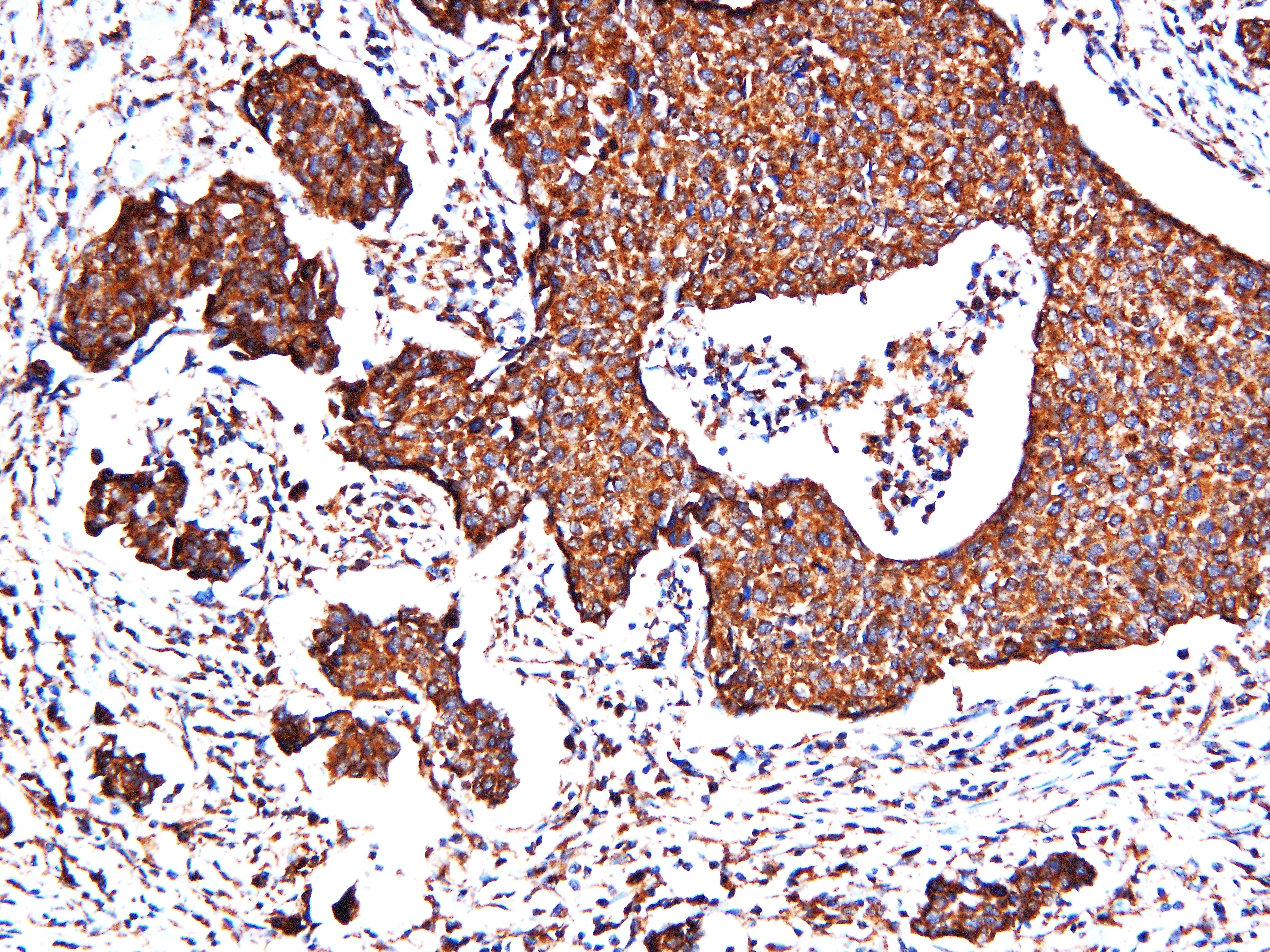

Supplement: Supplementary file 11 — Source Data for Figure 8 [file EMMM-13-e13270-s005.zip › Fig8/fig 8 A/1A7 200.jpg]

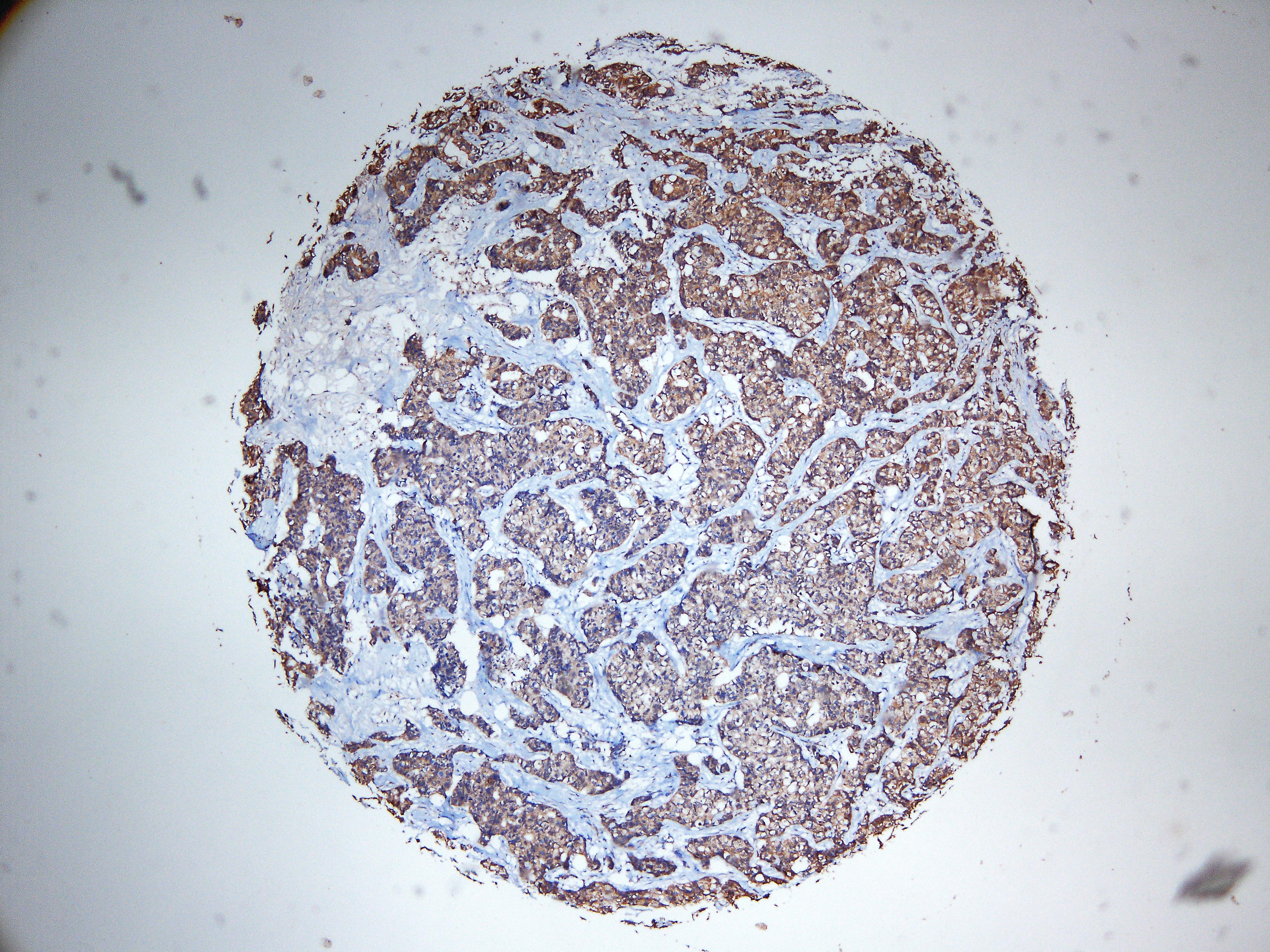

Supplement: Supplementary file 11 — Source Data for Figure 8 [file EMMM-13-e13270-s005.zip › Fig8/fig 8 A/201900001_265.jpg]

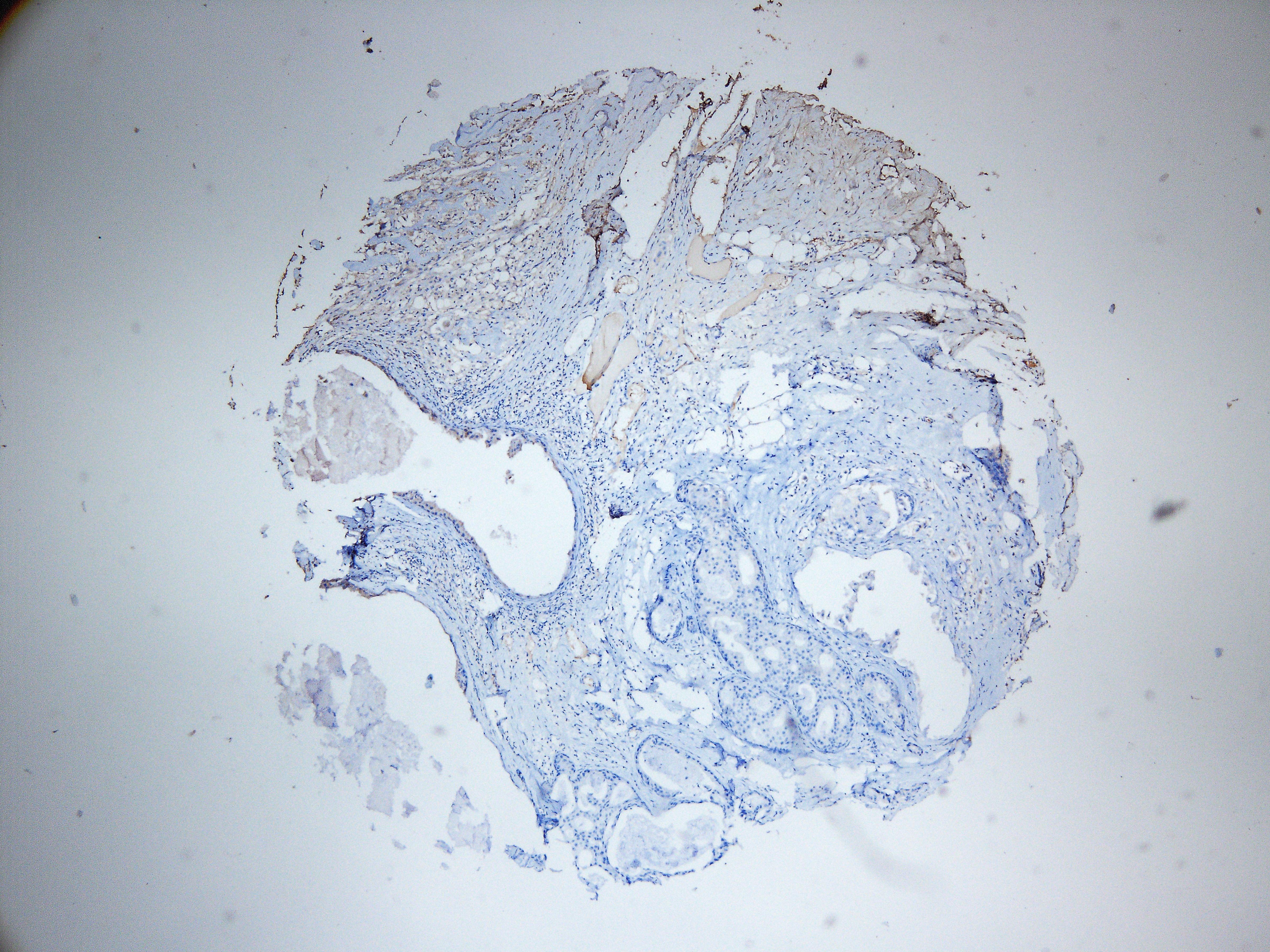

Supplement: Supplementary file 11 — Source Data for Figure 8 [file EMMM-13-e13270-s005.zip › Fig8/fig 8 A/201900001_354.jpg]

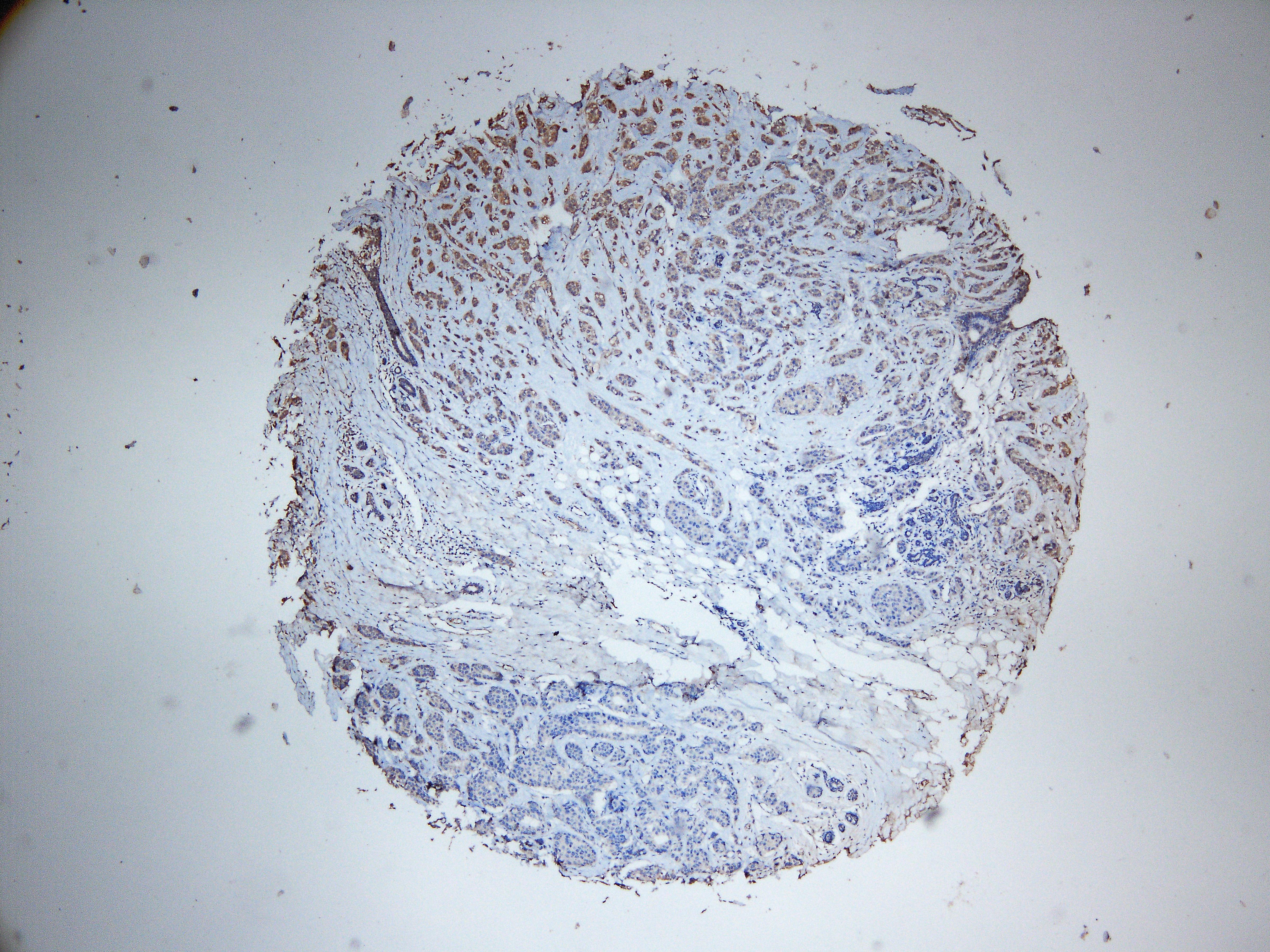

Supplement: Supplementary file 11 — Source Data for Figure 8 [file EMMM-13-e13270-s005.zip › Fig8/fig 8 A/201900001_364.jpg]

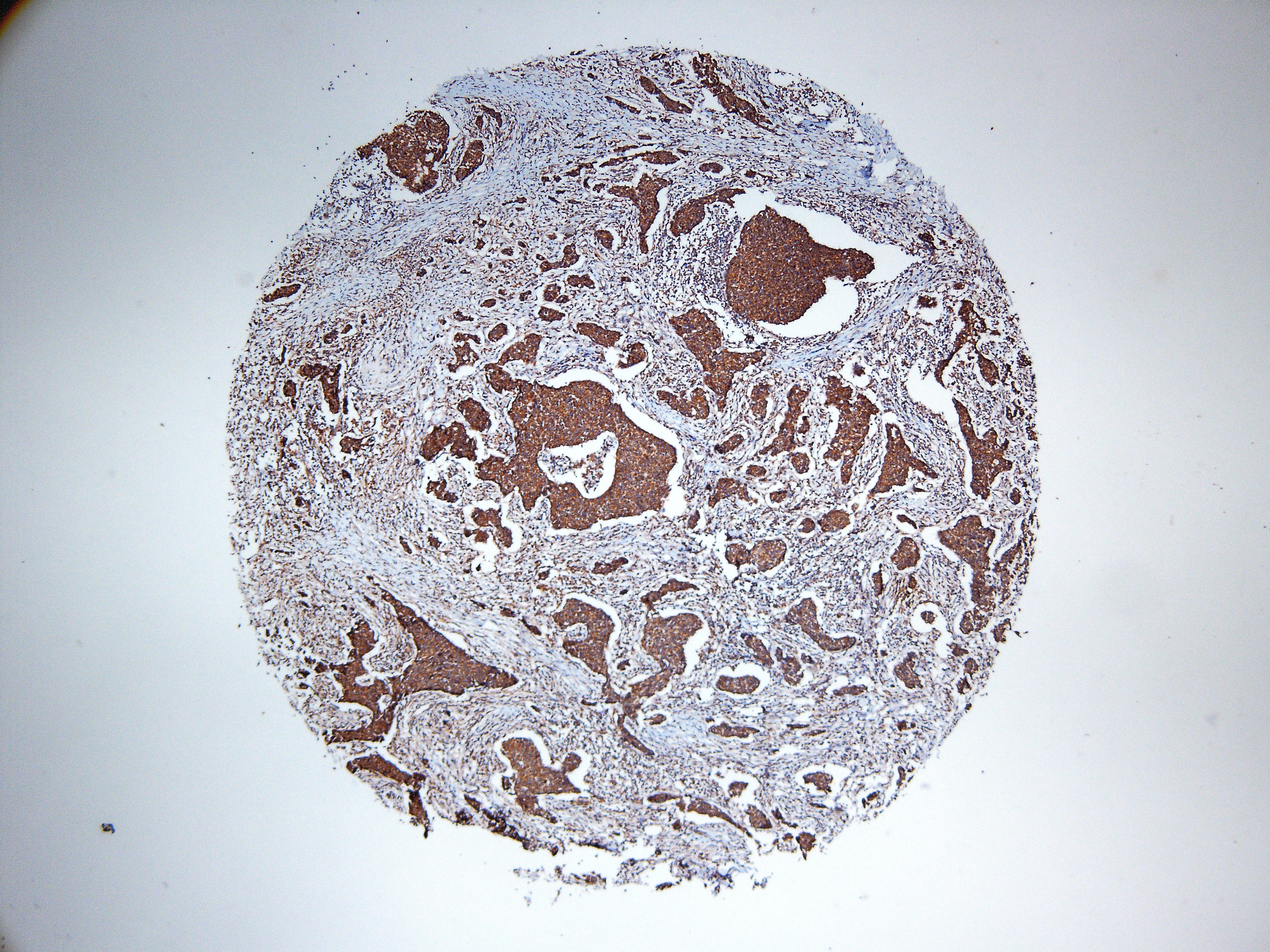

Supplement: Supplementary file 11 — Source Data for Figure 8 [file EMMM-13-e13270-s005.zip › Fig8/fig 8 A/201900001_9.jpg]

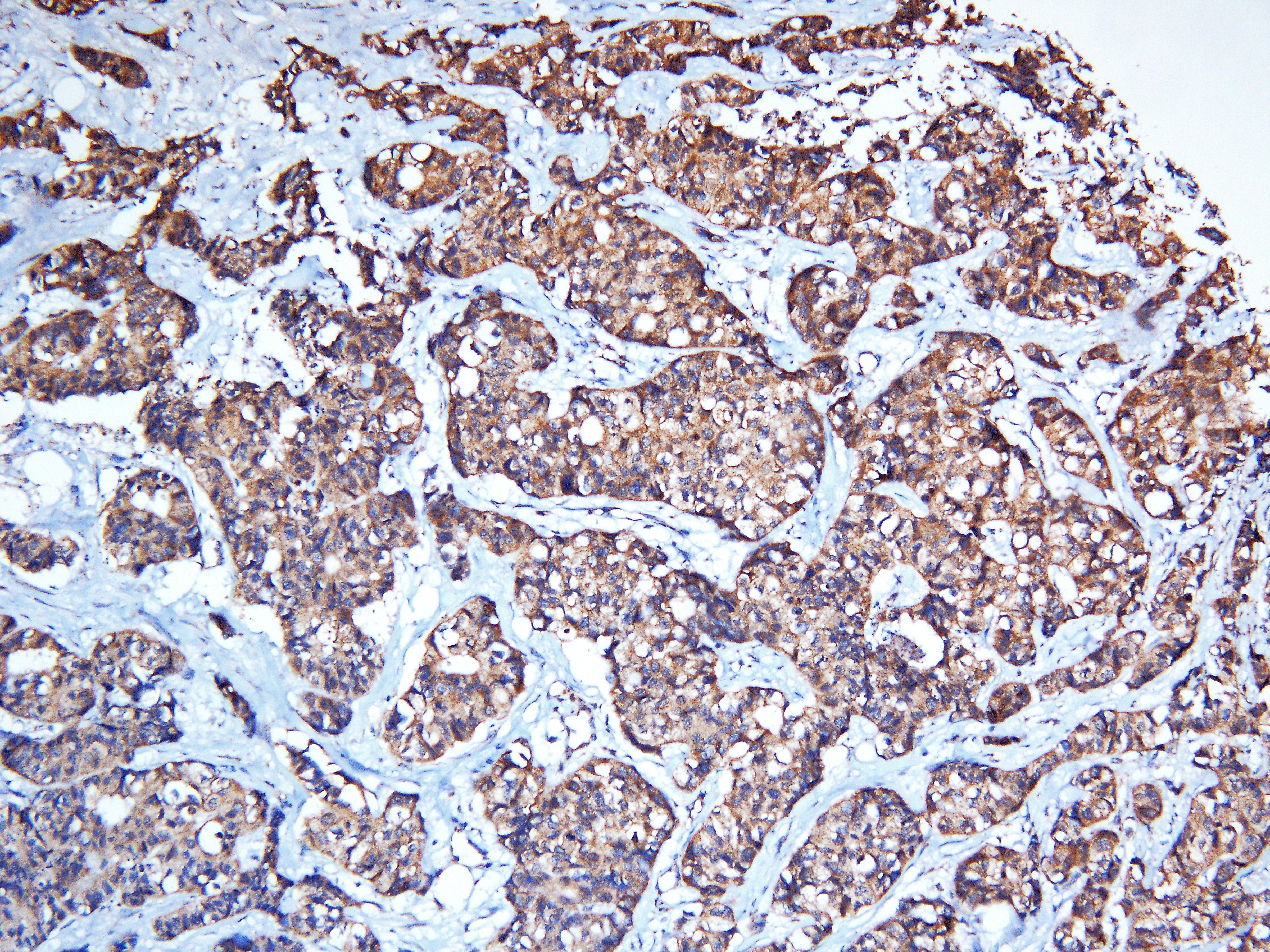

Supplement: Supplementary file 11 — Source Data for Figure 8 [file EMMM-13-e13270-s005.zip › Fig8/fig 8 A/4F2 200.jpg]

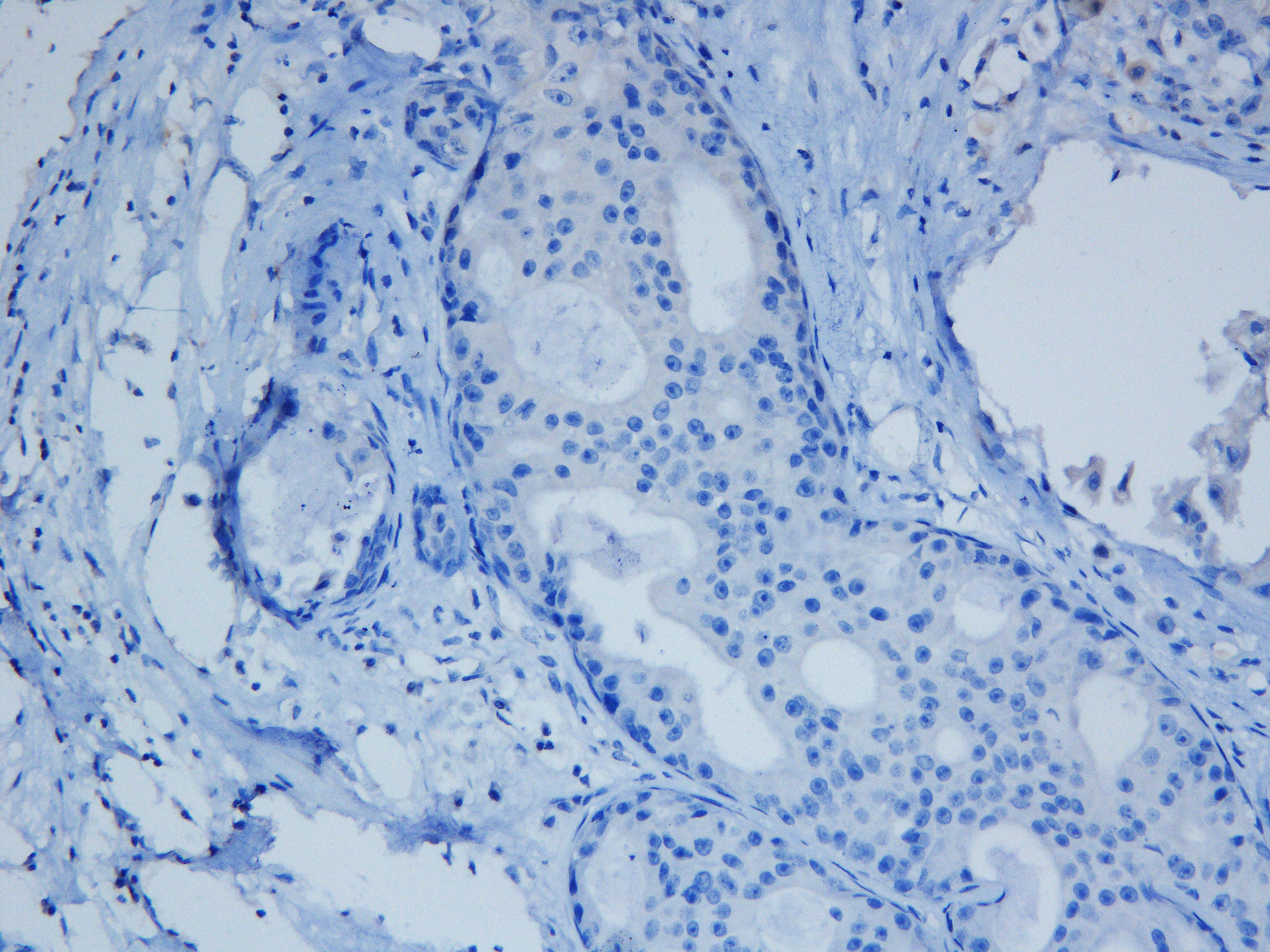

Supplement: Supplementary file 11 — Source Data for Figure 8 [file EMMM-13-e13270-s005.zip › Fig8/fig 8 A/6A1 200.jpg]

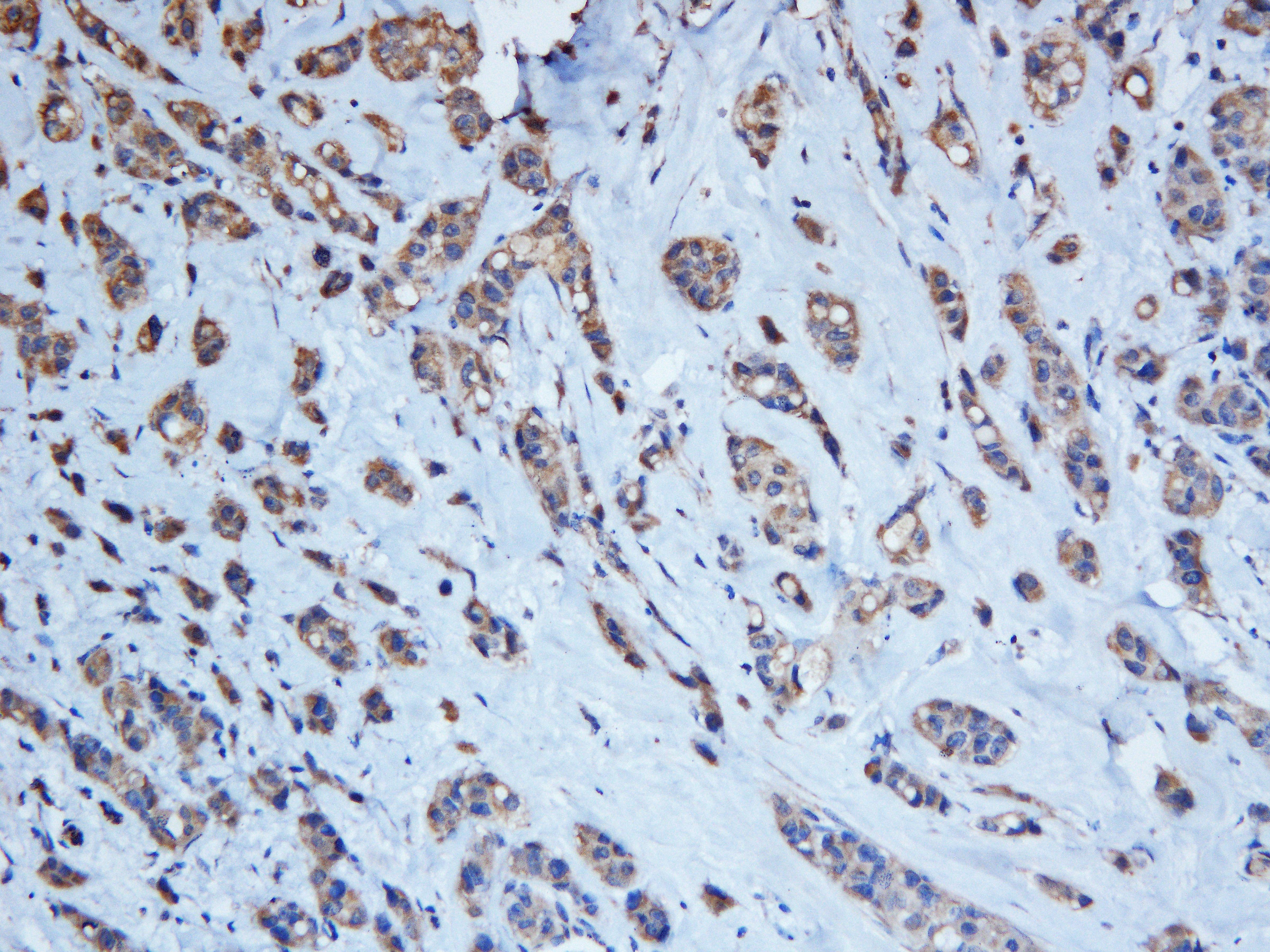

Supplement: Supplementary file 11 — Source Data for Figure 8 [file EMMM-13-e13270-s005.zip › Fig8/fig 8 A/6B1 200.jpg]
