# Supplementary material for: Tenascin‐C immobilizes infiltrating T lymphocytes through CXCL12 promoting breast cancer progression
Source: EMBO Mol Med. 2021 May 14;13(6):e13270. doi: 10.15252/emmm.202013270 (PMC8185552; doi:10.15252/emmm.202013270)
Supplement: Supplementary file 3 — Review Process File [file EMMM-13-e13270-s006.pdf]

EMBO Molecular Medicine

Tenascin-C immobilizes infiltrating T lymphocytes through CXCL12 promoting breast cancer progression

Devadarssen Murdamoothoo, Gertraud Orend, Zhen Sun, Alev Yilmaz, Claire Deligne, Ines Velazquez-Quesada, Chérine Abou-Faycal, William Erne, Matthias Mörgelin, Gérard Crémel, Nicodeme Paul, Raphael Carapito, Romain Veber, Hélène Dumortier, Jingping Yuan, Kim Midwood, Thomas Loustau, Gilles Riegel, and Marine Nascimento
DOI: 10.15252/emmm.202013270

Corresponding author(s): Gertraud Orend (gertraud.orend@inserm.fr)

Review Timeline:

Submission Date:	11th Aug 20
Editorial Decision:	7th Sep 20
Revision Received:	9th Feb 21
Editorial Decision:	2nd Mar 21
Revision Received:	12th Apr 21
Accepted:	12th Apr 21

Editor: Jingyi Hou

Transaction Report:

No Peer Review Process File is available with this article, as the authors have chosen not to make the review process public in this case.

EMBO PRESS

YOU MUST COMPLETE ALL CELLS WITH A PINK BACKGROUND ↓

PLEASE NOTE THAT THIS CHECKLIST WILL BE PUBLISHED ALONGSIDE YOUR PAPER

Corresponding Author Name: Gertraud Orend

Journal Submitted to: EMBO Molecular Medicine

Manuscript Number: EMM-2020-13270

Reporting Checklist For Life Sciences Articles (Rev. June 2017)

This checklist is used to ensure good reporting standards and to improve the reproducibility of published results. These guidelines are consistent with the Principles and Guidelines for Reporting Preclinical Research issued by the NIH in 2014. Please follow the journal's authorship guidelines in preparing your manuscript.

A- Figures

1. Data

The data shown in figures should satisfy the following conditions:

- the data were obtained and processed according to the field's best practice and are presented to reflect the results of the experiments in an accurate and unbiased manner.
- figure panels include only data points, measurements or observations that can be compared to each other in a scientifically meaningful way.
- graphs include clearly labeled error bars for independent experiments and sample sizes. Unless justified, error bars should not be shown for technical replicates.
- if n< 5, the individual data points from each experiment should be plotted and any statistical test employed should be justified
- Source Data should be included to report the data underlying graphs. Please follow the guidelines set out in the author ship guidelines on Data Presentation.

2. Captions

Each figure caption should contain the following information, for each panel where they are relevant:

- a specification of the experimental system investigated (eg cell line, species name).
- the assay(s) and method(s) used to carry out the reported observations and measurements
- an explicit mention of the biological and chemical entity(ies) that are being measured.
- an explicit mention of the biological and chemical entity(ies) that are altered/varied/perturbed in a controlled manner.

- the exact sample size (n) for each experimental group/condition, given as a number, not a range;
- a description of the sample collection allowing the reader to understand whether the samples represent technical or biological replicates (including how many animals, litters, cultures, etc.).
- a statement of how many times the experiment shown was independently replicated in the laboratory.
- definitions of statistical methods and measures:
  - common tests, such as t-test (please specify whether paired vs. unpaired), simple χ^2 tests, Wilcoxon and Mann-Whitney tests, can be unambiguously identified by name only, but more complex techniques should be described in the methods section;
  - are tests one-sided or two-sided?
  - are there adjustments for multiple comparisons?
  - exact statistical test results, e.g., P values = x but not P values < x;
  - definition of 'center values' as median or average;
  - definition of error bars as s.d. or s.e.m.

Any descriptions too long for the figure legend should be included in the methods section and/or with the source data.

In the pink boxes below, please ensure that the answers to the following questions are reported in the manuscript itself. Every question should be answered. If the question is not relevant to your research, please write NA (non applicable). We encourage you to include a specific subsection in the methods section for statistics, reagents, animal models and human subjects.

USEFUL LINKS FOR COMPLETING THIS FORM

http://www.antibodypedia.com
http://1degreebio.org
http://www.equator-network.org/reporting-guidelines/improving-bioscience-research-repor
http://grants.nih.gov/grants/olaw/olaw.htm
http://www.mrc.ac.uk/Ourresearch/Ethicsresearchguidance/Useofanimals/index.htm
http://ClinicalTrials.gov
http://www.consort-statement.org
http://www.consort-statement.org/checklists/view/32-consort/66-title
http://www.equator-network.org/reporting-guidelines/reporting-recommendations-for-tumo
http://datadryad.org
http://figshare.com
http://www.ncbi.nlm.nih.gov/gap
http://www.ebi.ac.uk/ega
http://biomodels.net/
http://biomodels.net/miriam/
http://jjj.biochem.sun.ac.za
http://oba.od.nih.gov/biosecurity/biosecurity_documents.html
http://www.selectagents.gov/

B- Statistics and general methods

Please fill out these boxes ↓ (Do not worry if you cannot see all your text once you press return)

1.a. How was the sample size chosen to ensure adequate power to detect a pre-specified effect size?	Cell culture: at least 3 independent experiments with one or more replicates.
1.b. For animal studies, include a statement about sample size estimate even if no statistical methods were used.	Mouse experiments: at least 5 per condition.
2. Describe inclusion/exclusion criteria if samples or animals were excluded from the analysis. Were the criteria pre-established?	No animals were excluded. Concerning the in vitro samples we used the Grubbs's test to exclude the outliers.
3. Were any steps taken to minimize the effects of subjective bias when allocating animals/samples to treatment (e.g. randomization procedure)? If yes, please describe.	Collection of mouse material (numbered) was blinded and performed by a second person. Analysis of the samples was blinded by using the numbers. Human sample analysis was done by 2 independent physicians and compiled.
For animal studies, include a statement about randomization even if no randomization was used.	Mice were randomized.
4.a. Were any steps taken to minimize the effects of subjective bias during group allocation or/and when assessing results (e.g. blinding of the investigator)? If yes please describe.	Studies were done blinded.
4.b. For animal studies, include a statement about blinding even if no blinding was done	Analysis of the tumor mice was done blinded.
5. For every figure, are statistical tests justified as appropriate?	Appropriate statistical tests were used, taking into account e.g how many conditions are compared and whether the data have a normal distribution or not.
Do the data meet the assumptions of the tests (e.g., normal distribution)? Describe any methods used to assess it.	For each graph we tested whether the values come from a Gaussian distribution using D'Agostino-Pearson omnibus normality test and Shapiro-Wilk normality test.
Is there an estimate of variation within each group of data?	We used the standard error of the mean to estimate the variation within each group of data

Is the variance similar between the groups that are being statistically compared?	When unpaired t test was used to compare groups having a normal distribution, the F-test of equality of variances confirmed a similar variance between those groups.

C- Reagents

6. To show that antibodies were profiled for use in the system under study (assay and species), provide a citation, catalog number and/or clone number, supplementary information or reference to an antibody validation profile. e.g., Antibodypedia (see link list at top right), 1DegreeBio (see link list at top right).	Information about manufacturer, order number and dilution are provided in Supplemental tables.
7. Identify the source of cell lines and report if they were recently authenticated (e.g., by STR profiling) and tested for mycoplasma contamination.	The NT193 cell line was previously published in Arpel et al., 2014, Cell Reports 8: 1714–1721; Sun et al., 2019, Mat Bio83, 26-47; Deligne et al., 2020, Cancer Immun Res 8, 368 - 382. Cells were regularly (every two months) checked by a mycoplasm test (Venor®GeM OneStep, catalog number 11-8050, Clinisciences)

* for all hyperlinks, please see the table at the top right of the document

D- Animal Models

8. Report species, strain, gender, age of animals and genetic modification status where applicable. Please detail housing and husbandry conditions and the source of animals.	Information about the mouse strain, source and husbandry has been provided.
9. For experiments involving live vertebrates, include a statement of compliance with ethical regulations and identify the committee(s) approving the experiments.	The statement of compliance with ethical regulations and authorization has been included.
10. We recommend consulting the ARRIVE guidelines (see link list at top right) (PLoS Biol. 8(6), e1000412, 2010) to ensure that other relevant aspects of animal studies are adequately reported. See author guidelines, under 'Reporting Guidelines'. See also: NIH (see link list at top right) and MRC (see link list at top right) recommendations. Please confirm compliance.	We confirm compliance after having read the guidelines

E- Human Subjects

11. Identify the committee(s) approving the study protocol.	Ethics committe of the Hubei Cancer Hospital, China. INSERM and ethical committee of Alsace, France (CREMEAS)
12. Include a statement confirming that informed consent was obtained from all subjects and that the experiments conformed to the principles set out in the WMA Declaration of Helsinki and the Department of Health and Human Services Belmont Report.	This statement has been included. The experiments conform with the WMA Declaration of Helsinkini and the Department of Health and Human Services Belmont Report.
13. For publication of patient photos, include a statement confirming that consent to publish was obtained.	Not applicable.
14. Report any restrictions on the availability (and/or on the use) of human data or samples.	Not applicable.
15. Report the clinical trial registration number (at ClinicalTrials.gov or equivalent), where applicable.	Not applicable.
16. For phase II and III randomized controlled trials, please refer to the CONSORT flow diagram (see link list at top right) and submit the CONSORT checklist (see link list at top right) with your submission. See author guidelines, under 'Reporting Guidelines'. Please confirm you have submitted this list.	Not applicable.
17. For tumor marker prognostic studies, we recommend that you follow the REMARK reporting guidelines (see link list at top right). See author guidelines, under 'Reporting Guidelines'. Please confirm you have followed these guidelines.	Not applicable.

F- Data Accessibility

18: Provide a "Data Availability" section at the end of the Materials & Methods, listing the accession codes for data generated in this study and deposited in a public database (e.g. RNA-Seq data: Gene Expression Omnibus GSE39462, Proteomics data: PRIDE PXD000208 etc.) Please refer to our author guidelines for 'Data Deposition'.<br><br>Data deposition in a public repository is mandatory for:<br>a. Protein, DNA and RNA sequences<br>b. Macromolecular structures<br>c. Crystallographic data for small molecules<br>d. Functional genomics data<br>e. Proteomics and molecular interactions	The upload of data was done.
19. Deposition is strongly recommended for any datasets that are central and integral to the study; please consider the journal's data policy. If no structured public repository exists for a given data type, we encourage the provision of datasets in the manuscript as a Supplementary Document (see author guidelines under 'Expanded View' or in unstructured repositories such as Dryad (see link list at top right) or Figshare (see link list at top right).	All profiling results have been provided in the supplemental information part.
20. Access to human clinical and genomic datasets should be provided with as few restrictions as possible while respecting ethical obligations to the patients and relevant medical and legal issues. If practically possible and compatible with the individual consent agreement used in the study, such data should be deposited in one of the major public access-controlled repositories such as dbGAP (see link list at top right) or EGA (see link list at top right).	We used publicly available human data sets and provided the information. GSE 19783-GPL6580 and Chen C, Xia H-S, Gong Y-P, Peng J, Peng C-W, Hu M-B, Zhu X-B, Pang D-W, Sun S-R, Li Y (2010) The quantitative detection of total HER2 load by quantum dots and the identification of a new subtype of breat cancer with different 5-year pronosis. Biomaterials 31: 8818-8825
21. Computational models that are central and integral to a study should be shared without restrictions and provided in a machine-readable form. The relevant accession numbers or links should be provided. When possible, standardized format (SBML, CellML) should be used instead of scripts (e.g. MATLAB). Authors are strongly encouraged to follow the MIRIAM guidelines (see link list at top right) and deposit their model in a public database such as Biomodels (see link list at top right) or JWS Online (see link list at top right). If computer source code is provided with the paper, it should be deposited in a public repository or included in supplementary information.	Not applicable.

G- Dual use research of concern

22. Could your study fall under dual use research restrictions? Please check biosecurity documents (see link list at top right) and list of select agents and toxins (APHIS/CDC) (see link list at top right). According to our biosecurity guidelines, provide a statement only if it could.	
